# Oral small-molecule GLP-1 receptor agonist safiglipron in early type 2 diabetes: a randomized, double-blind, placebo-controlled trial

**DOI:** 10.1038/s41591-026-04651-9

**Published:** 2026-09-29

**Authors:** Miao Yu, Liang Peng, Chengyan Jiang, Xianghua Zhang, Qifu Li, Lihui Zhang, Xiuhai Su, Zhaoyang Zeng, Shuang Yan, Jinyang Wang, Debin Huang, Sihong Wang, Na Liu, Yimei Xu, Guo Tang, Fangli Dong, Yanlin Ma, Zi Ye, Xinhua Xiao

**Affiliations:** 1 Department of Endocrinology, Peking Union Medical College Hospital, Chinese Academy of Medical Sciences and Peking Union Medical College, Beijing, China; 2 Clinical Research and Development, Jiangsu Hengrui Pharmaceuticals Co. Ltd., Shanghai, China; 3 Department of Endocrinology, The First People’s Hospital of Zunyi, Zunyi, China; 4 Department of General Medicine, Yueyang Central Hospital, Yueyang, China; 5 Department of Endocrinology, The First Affiliated Hospital of Chongqing Medical University, Chongqing, China; 6 Department of Endocrinology, The Second Hospital of Hebei Medical University, Shijiazhuang, China; 7 Endocrinology Diabetes Ward 1, Cangzhou Hospital of Integrated TCM-WM Hebei, Cangzhou, China; 8 Department of Endocrinology, Yichang Central People’s Hospital, Yichang, China; 9 Department of Endocrinology, The Fourth Hospital Affiliated to Harbin Medical University, Harbin, China; 10 Department of Endocrinology, Gansu Provincial Hospital, Lanzhou, China; 11 Department of Endocrinology, The Third Hospital of Changsha, Changsha, China; 12 Department of Endocrinology, Xuancheng People’s Hospital, Xuancheng, China; 13 Department of Endocrinology, Liuzhou People’s Hospital, Liuzhou, China

**Keywords:** Type 2 diabetes, Phase III trials

## Abstract

Safiglipron is an oral small-molecule glucagon-like peptide-1 (GLP-1) receptor agonist administered without fasting or dietary restrictions. We evaluated its efficacy and safety in OUTSTAND-1, a phase 3, multicenter, randomized, double-blind, placebo-controlled trial at 46 sites in China. We randomized 284 adults with type 2 diabetes managed with diet and exercise alone (mean baseline HbA1c 7.95%, median diabetes duration 1.6 years, 33.1% women) to once-daily safiglipron 30 mg (*n* = 70), 60 mg (*n* = 70), 90 mg (*n* = 72) or placebo (*n* = 72) for 32 weeks, followed by a 20-week active-treatment extension. For the primary endpoint, placebo-adjusted treatment differences in HbA1c change from baseline to week 32 were −1.22% (95% CI, −1.53 to −0.91), −1.20% (95% CI, −1.52 to −0.89) and −1.45% (95% CI, −1.75 to −1.14) for 30 mg, 60 mg and 90 mg, respectively (all *P* < 0.0001; treatment policy estimand). Secondary outcomes showed HbA1c < 7.0% in 71.4−77.8% versus 25.0%, HbA1c ≤ 6.5% in 58.6−68.1% versus 16.7% and placebo-adjusted fasting plasma glucose differences of −1.58, −1.68 and −2.08 mmol l^−^^1^, respectively (all *P* < 0.0001). Body weight differences were modest (−0.65%, −2.23% and −3.56% versus placebo). Other secondary outcomes generally favored safiglipron for HbA1c < 5.7% attainment, postprandial glycemia, homeostatic model assessment of β cell function (HOMA-β), homeostatic model assessment of insulin resistance (HOMA-IR), disposition index and waist circumference, with less rescue therapy use. Insulin and C-peptide responses varied by dose, and changes in treatment satisfaction were limited. Gastrointestinal adverse events were most common and mostly mild or moderate. Adverse events led to treatment discontinuation in 1.4%, 2.9%, 6.9% and 0% of participants receiving safiglipron 30 mg, 60 mg, 90 mg and placebo, respectively. These findings support once-daily oral safiglipron as an effective treatment option for type 2 diabetes. ClinicalTrials.gov identifier: NCT06672172.

## Abstract (web summary)

The OUTSTAND-1 trial showed that once-daily oral small-molecule GLP-1 receptor agonist safiglipron reduced HbA1c by up to 1.45% versus placebo over 32 weeks in people with type 2 diabetes without glucose-lowering medication while body weight effects were modest.

## Main

Diabetes affects an estimated 589 million adults worldwide, more than 90% of whom have type 2 diabetes^1^. β cell dysfunction and insulin resistance drive progressive loss of glycemic control, underscoring the importance of effective treatment early in the disease course^2^. GLP-1 receptor agonists provide effective glucose lowering and weight reduction, with cardiorenal benefits established across multiple large outcome trials^3^. Current guidelines recommend their use regardless of metformin in people with established or high-risk cardiovascular disease or chronic kidney disease, reflecting the expanding role of this class beyond conventional stepwise glucose-lowering intensification^4^.

Despite these benefits, most approved GLP-1 receptor agonists require subcutaneous injection, which can represent a practical barrier for some patients^5^. Oral semaglutide partly addressed this barrier but, as a peptide-based formulation, requires fasting administration with restricted fluid intake^6,7^. Oral non-peptide small-molecule GLP-1 receptor agonists may avoid these peptide-related dosing constraints. Orforglipron has demonstrated glycemic and weight benefits across phase 3 trials in diverse clinical settings^8–11^.

Safiglipron (proposed International Nonproprietary Name (INN)^12^, HRS-7535) is a structurally distinct, once-daily oral small-molecule GLP-1 receptor agonist. Across three phase 2 proof-of-concept and dose-finding trials, safiglipron reduced HbA1c in patients with type 2 diabetes inadequately controlled with metformin^13^, body weight in adults with obesity^14^ and albuminuria in patients with diabetic kidney disease^15^. On the basis of these phase 2 findings, three safiglipron doses were advanced into the phase 3 OUTSTAND clinical development program. We conducted the OUTSTAND-1 trial to assess the efficacy and safety of safiglipron as monotherapy in Chinese adults with type 2 diabetes managed with diet and exercise alone.

## Results

### Trial design and participants

**Extended Data Fig. 1:**
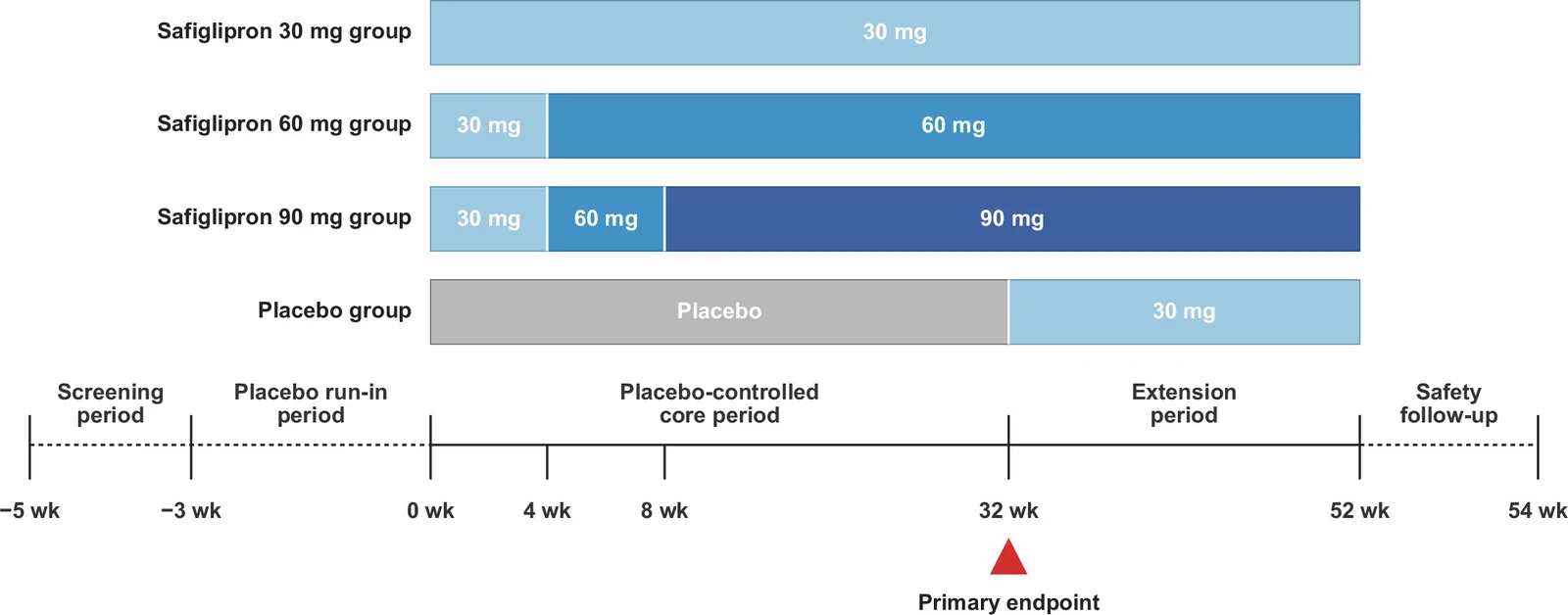
Study design. Participants assigned to safiglipron 30 mg received the target dose from baseline. Participants assigned to safiglipron 60 mg or 90 mg started at 30 mg once daily, with dose escalation in 30 mg increments every 4 weeks until the assigned target dose was reached. Participants assigned to placebo received placebo during the 32-week placebo-controlled core period and switched to safiglipron 30 mg at week 33 for the 20-week extension period.

OUTSTAND-1 was a multicenter, randomized, double-blind, placebo-controlled trial evaluating once-daily oral safiglipron (30 mg, 60 mg and 90 mg, with dose titration in 30-mg increments every 4 weeks) versus placebo in adults aged 18−75 years with type 2 diabetes (HbA1c 7.0−10.0%, body mass index (BMI) 19.0−40.0 kg m^−^^2^) managed with diet and exercise without glucose-lowering medication for at least 2 months. The trial comprised a 32-week placebo-controlled core period followed by a 20-week extension period (Extended Data Fig. 1).

**Fig. 1:**
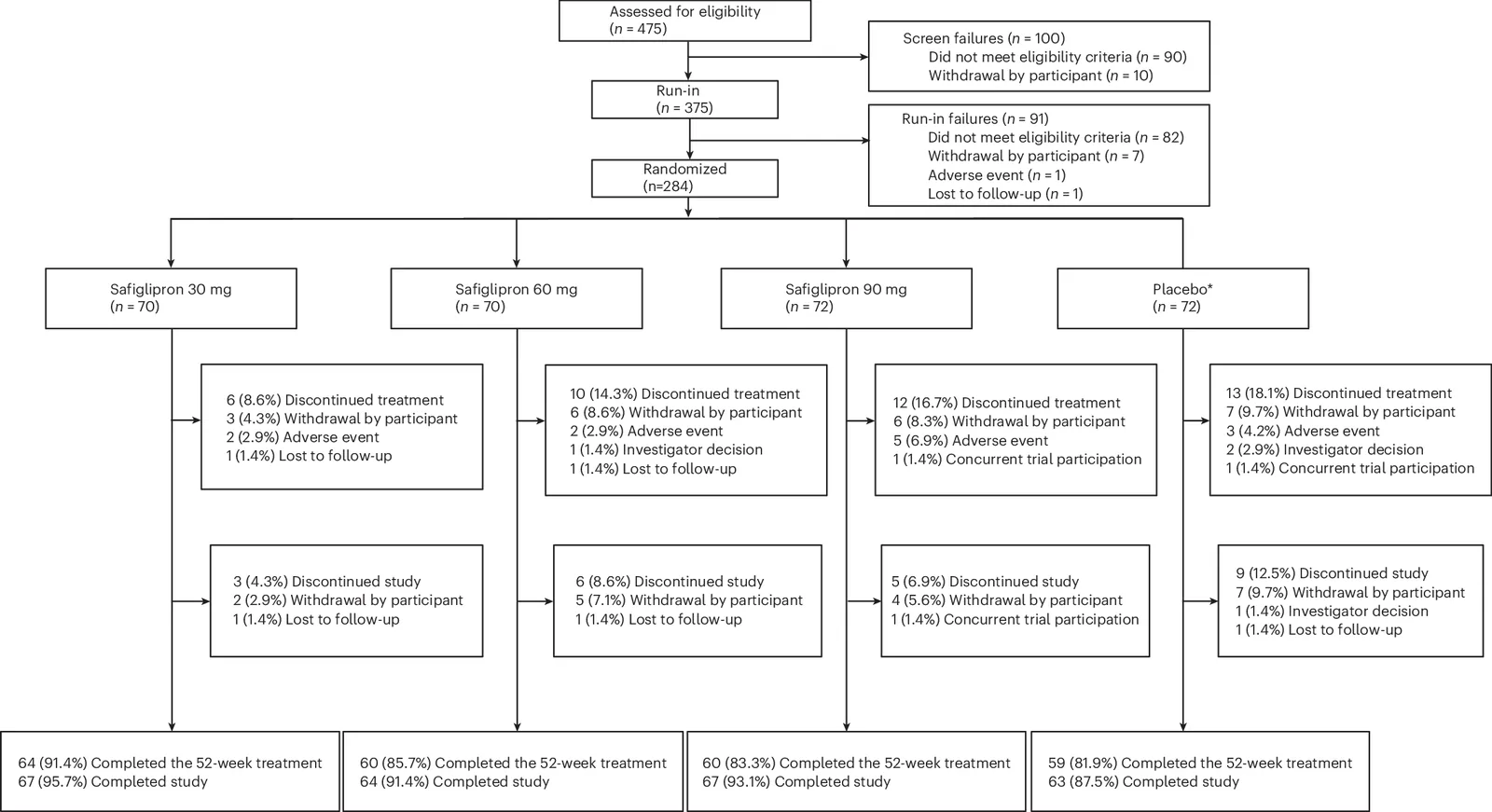
CONSORT diagram. Reasons for treatment discontinuation were classified according to the single primary reason recorded in the trial database. ‘Adverse event’ denotes discontinuation determined by the investigator to be caused by an adverse event, whereas ‘withdrawal by participant’ denotes voluntary discontinuation for personal reasons that did not involve an adverse event. Percentages were calculated using the number of participants randomly assigned to each group as the denominator. The asterisk indicates participants initially assigned to placebo received placebo during the 32-week placebo-controlled core period and switched to safiglipron 30 mg at week 33 for the 20-week extension.

Participants were enrolled between 21 October 2024 and 31 March 2025. Overall, 475 individuals were screened at 46 sites in China, of whom 375 entered the single-blind placebo run-in period. In total, 284 participants were randomly assigned to safiglipron 30 mg (*n* = 70), safiglipron 60 mg (*n* = 70), safiglipron 90 mg (*n* = 72) or placebo (*n* = 72). A total of 243 (85.6%) participants completed the 52-week treatment period: 64 (91.4%) in the safiglipron 30 mg group, 60 (85.7%) in the safiglipron 60 mg group, 60 (83.3%) in the safiglipron 90 mg group and 59 (81.9%) in the placebo group. Overall, 261 (91.9%) participants completed trial follow-up: 67 (95.7%), 64 (91.4%), 67 (93.1%) and 63 (87.5%), respectively. Over the 52-week treatment period, the most common reasons for treatment discontinuation were participant withdrawal (22 (7.7%)) and adverse events (12 (4.2%)) (Fig. 1).

Demographic and baseline clinical characteristics were generally balanced across treatment groups (Table 1). All participants were Asian. The mean age was 49.4 years (s.d. 9.9), 94 (33.1%) of 284 participants were female and the median duration of type 2 diabetes was 1.6 years (Q1−Q3 0.5−3.0). At baseline, the mean HbA1c was 7.95% (s.d. 0.71), body weight was 77.5 kg (s.d. 14.0) and BMI was 27.8 kg m^−^^2^ (s.d. 3.7). A total of 189 (66.5%) participants had not previously received glucose-lowering medication; the remaining 95 (33.5%) had previously received glucose-lowering medication but had received none for at least 2 months before screening.

**Table 1:** Baseline demographic and clinical characteristics

Characteristic	Safiglipron 30 mg (*N* = 70)	Safiglipron 60 mg (*N* = 70)	Safiglipron 90 mg (*N* = 72)	Placebo (*N* = 72)	Total (*N* = 284)
Age, years	48.7 (8.9)	48.9 (10.2)	50.3 (10.2)	49.7 (10.2)	49.4 (9.9)
Sex, *n* (%)					
Male	41 (58.6)	49 (70.0)	52 (72.2)	48 (66.7)	190 (66.9)
Female	29 (41.4)	21 (30.0)	20 (27.8)	24 (33.3)	94 (33.1)
HbA1c, %	7.87 (0.73)	7.97 (0.68)	7.98 (0.63)	7.99 (0.79)	7.95 (0.71)
HbA1c category, *n* (%)^a^					
≤8.5%	56 (80.0)	56 (80.0)	59 (81.9)	57 (79.2)	228 (80.3)
>8.5%	14 (20.0)	14 (20.0)	13 (18.1)	15 (20.8)	56 (19.7)
FPG, mmol l^−1^	9.2 (1.7)	9.5 (1.9)	9.7 (2.1)	9.3 (2.0)	9.4 (1.9)
HOMA-β, %	45.5 (32.2−68.7)	47.6 (32.6−64.7)	38.7 (27.1−66.2)	47.3 (29.2−80.8)	44.8 (31.2−69.3)
HOMA-IR	4.9 (3.4−8.4)	5.7 (4.0−8.3)	5.6 (3.8−7.6)	5.8 (3.1−8.2)	5.5 (3.5−8.3)
Disposition index^b^	266.1 (200.0−356.7)	227.2 (173.0−301.0)	238.4 (171.5−315.7)	286.3 (210.8−350.1)	254.0 (178.8−328.5)
Body weight, kg	76.3 (11.2)	77.9 (14.8)	77.6 (16.2)	78.0 (13.5)	77.5 (14.0)
BMI, kg m^−^^2^	27.7 (3.4)	27.9 (3.6)	27.4 (3.9)	28.3 (3.9)	27.8 (3.7)
BMI category, *n* (%)^c^					
<24.0 kg m^−^^2^	7 (10.0)	10 (14.3)	14 (19.4)	9 (12.5)	40 (14.1)
24.0 to <28.0 kg m^−^^2^	36 (51.4)	31 (44.3)	29 (40.3)	27 (37.5)	123 (43.3)
≥28.0 kg m^−^^2^	27 (38.6)	29 (41.4)	29 (40.3)	36 (50.0)	121 (42.6)
Waist circumference, cm	95.7 (9.1)	97.5 (9.8)	96.7 (11.1)	97.9 (9.5)	97.0 (9.9)
Duration of type 2 diabetes, years	1.8 (0.6−3.2)	1.3 (0.5−2.7)	1.7 (0.6−3.1)	1.3 (0.4−3.1)	1.6 (0.5−3.0)
Previous glucose-lowering medication, *n* (%)					
Yes	22 (31.4)	26 (37.1)	24 (33.3)	23 (31.9)	95 (33.5)
No	48 (68.6)	44 (62.9)	48 (66.7)	49 (68.1)	189 (66.5)
Systolic blood pressure, mmHg	122.9 (11.1)	124.0 (11.7)	124.1 (11.9)	123.7 (11.1)	123.7 (11.4)
Diastolic blood pressure, mmHg	83.7 (7.9)	82.5 (8.3)	82.2 (8.1)	83.2 (6.3)	82.9 (7.7)
Antihypertensive medication use at baseline, *n* (%)					
Yes	22 (31.4)	20 (28.6)	25 (34.7)	27 (37.5)	94 (33.1)
No	48 (68.6)	50 (71.4)	47 (65.3)	45 (62.5)	190 (66.9)
Total cholesterol, mmol l^−^^1^	5.0 (1.1)	5.3 (1.1)	4.9 (1.1)	5.0 (1.0)	5.0 (1.1)
Triglycerides, mmol l^−^^1^	2.2 (1.6)	2.4 (1.6)	2.2 (1.7)	2.4 (1.5)	2.3 (1.6)
LDL cholesterol, mmol l^−^^1^	3.1 (0.9)	3.3 (1.0)	3.0 (0.9)	3.0 (0.8)	3.1 (0.9)
HDL cholesterol, mmol l^−^^1^	1.2 (0.2)	1.2 (0.3)	1.2 (0.3)	1.2 (0.3)	1.2 (0.3)

Data are mean (s.d.), median (Q1−Q3) or *n* (%), unless otherwise specified. The intention-to-treat population included all randomly assigned participants. Percentages were calculated using the number of participants in each treatment group as the denominator and might not sum to 100 because of rounding.

^a^HbA1c categories were based on the randomization stratification cutoff.

^b^The disposition index (ISSI-2) was derived from MMTT measures and used as a composite measure of insulin secretion adjusted for insulin sensitivity.

^c^BMI categories were based on Chinese criteria for normal weight, overweight and obesity.

The primary endpoint was change from baseline in HbA1c at week 32. Confirmatory secondary endpoints were the proportions achieving HbA1c < 7.0% and HbA1c ≤ 6.5%, change in fasting plasma glucose (FPG) and percentage change in body weight, all at week 32. Prespecified supportive secondary endpoints included changes in indices of β cell function and insulin sensitivity (HOMA-β, HOMA-IR and disposition index), postprandial glucose and insulin responses during a mixed-meal tolerance test (MMTT), waist circumference, seven-point self-monitored blood glucose (SMBG) profiles and the proportion receiving rescue therapy. Prespecified exploratory endpoints included changes in blood pressure, lipid parameters and urinary albumin-to-creatinine ratio (UACR). Patient-reported treatment satisfaction was assessed using the Diabetes Treatment Satisfaction Questionnaire, status version (DTSQs). Safety endpoints included adverse events, hypoglycemic episodes, laboratory parameters, electrocardiograms and diabetic retinopathy assessments.

### Primary outcome

**Fig. 2:**
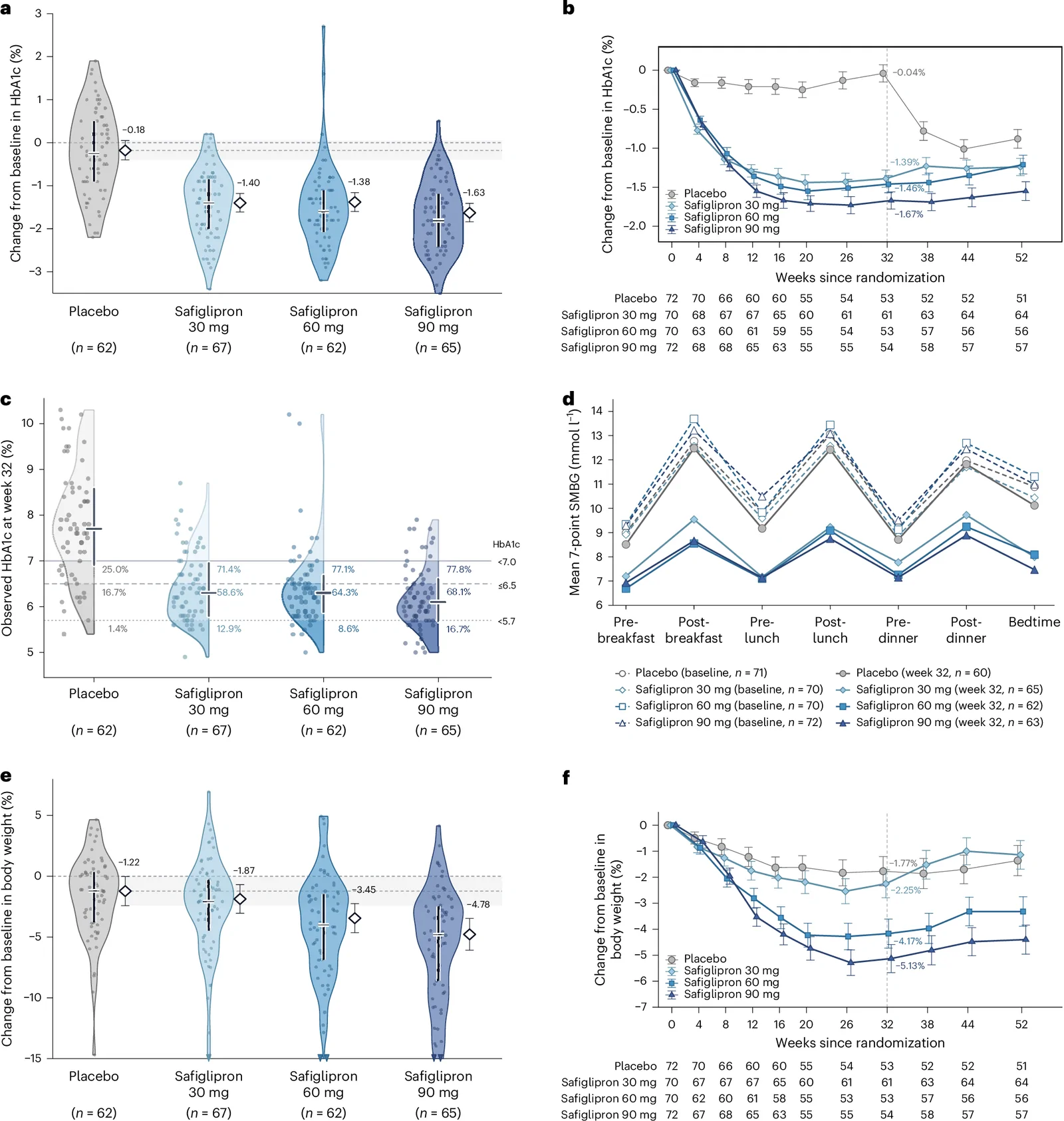
Glycemic and body weight outcomes. **a**, Change from baseline in HbA1c at week 32. Violins and points show observed data, boxes show medians and interquartile ranges and diamonds show treatment policy least squares means with 95% CIs. **b**, Least squares mean changes in HbA1c through week 52 under the supportive efficacy estimand; error bars indicate standard errors. **c**, Observed HbA1c distributions and target attainment at week 32. Half-violins and points show observed data, vertical lines indicate interquartile ranges, short horizontal lines indicate medians and percentages indicate treatment policy model-estimated target attainment rates. Horizontal lines mark the three HbA1c targets. **d**, Observed mean seven-point SMBG profiles at baseline and week 32, measured before and 2 hours after each meal and at bedtime. **e**, Percentage change from baseline in body weight at week 32, presented as in **a**. **f**, Least squares mean percentage changes in body weight through week 52 under the supportive efficacy estimand; error bars indicate standard errors. In **a** and **e**, the dashed line and shaded band indicate the placebo least squares mean and 95% CI. Vertical dashed lines in **b** and **f** mark week 32, after which participants assigned to placebo switched to safiglipron 30 mg. Treatment policy estimates in **a**, **c** and **e** used all randomized participants. Exact *n* values are shown in each panel and denote participants with available observed data at the indicated visit. Analyses used the intention-to-treat population. ANCOVA was used in **a** and **e**, GEE in **c** and MMRM in **b** and **f**. Source data

At week 32, all three safiglipron doses produced greater reductions in HbA1c than placebo, with the numerically greatest reduction observed in the safiglipron 90 mg group (Fig. 2a). Based on the treatment policy estimand, least squares mean changes from baseline in HbA1c were −1.40% (95% CI, −1.61 to −1.18) with safiglipron 30 mg, −1.38% (95% CI, −1.60 to −1.16) with safiglipron 60 mg and −1.63% (95% CI, −1.84 to −1.41) with safiglipron 90 mg compared to −0.18% (95% CI, −0.40 to 0.05) with placebo. Placebo-adjusted treatment differences were −1.22% (95% CI, −1.53 to −0.91) for safiglipron 30 mg, −1.20% (95% CI, −1.52 to −0.89) for safiglipron 60 mg and −1.45% (95% CI, −1.75 to −1.14) for safiglipron 90 mg (all *P* < 0.0001). Findings were consistent under the supportive efficacy estimand (hypothetical strategy estimand; Fig. 2b and Supplementary Table 3).

**Extended Data Fig. 2:**
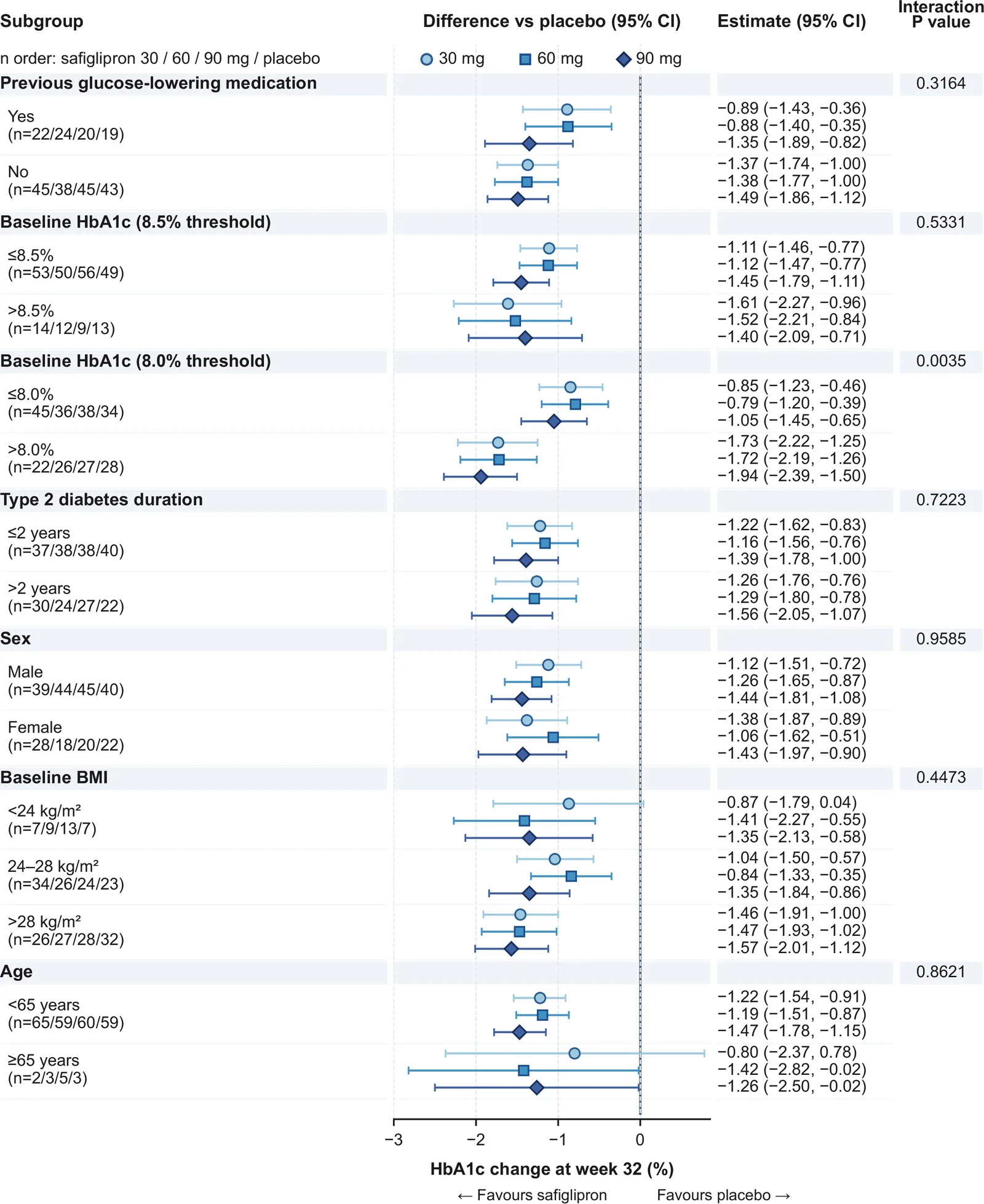
Subgroup analysis of HbA1c change from baseline at week 32. Treatment differences versus placebo in HbA1c change from baseline to week 32 are shown across prespecified subgroups. Data are least-squares mean differences versus placebo with two-sided 95% CIs, estimated using ANCOVA in the intention-to-treat population. Interaction P values were calculated using two-sided F tests of the treatment-by-subgroup interaction. All interaction P values were nominal and were not adjusted for multiplicity. ANCOVA=analysis of covariance. BMI=body mass index. CI=confidence interval. HbA1c=glycated hemoglobin. LS=least-squares. Source data

Prespecified subgroup analyses showed consistent HbA1c reductions with safiglipron versus placebo across subgroups defined by previous glucose-lowering medication use, type 2 diabetes duration, sex, baseline BMI and age (Extended Data Fig. 2). A treatment-by-subgroup interaction was observed for baseline HbA1c (*P*_interaction_ = 0.0035), with greater reductions in participants with baseline HbA1c above 8.0% than in those at or below 8.0%.

### Confirmatory secondary outcomes

#### HbA1c target attainment

**Extended Data Fig. 3:**
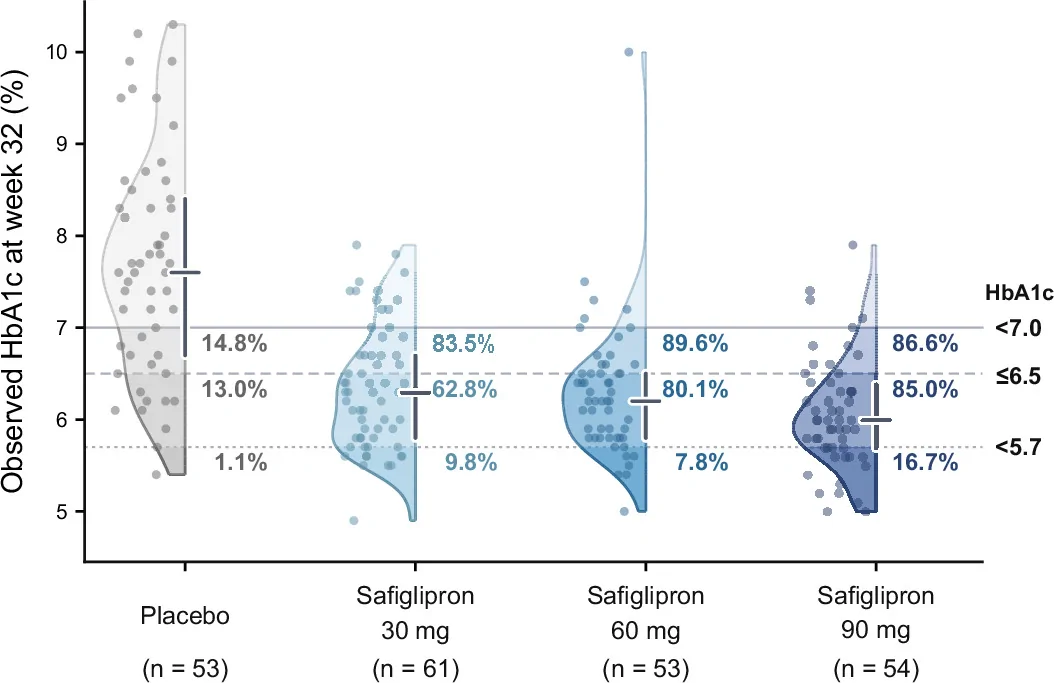
Observed HbA1c distribution and target attainment at week 32 under the supportive efficacy estimand. Points and half-violin plots show observed week-32 HbA1c values and their distributions; vertical lines indicate interquartile ranges, and short horizontal lines indicate medians. Horizontal reference lines mark the HbA1c targets of <7.0%, ≤6.5% and <5.7%. Percentages adjacent to the reference lines are GEE model-estimated marginal target-attainment rates under the supportive efficacy estimand (hypothetical strategy), with post-intercurrent-event data set to missing. The n values shown with the group labels denote participants with available week-32 HbA1c data contributing to the GEE analysis: n = 53, 61, 53 and 54 for placebo and safiglipron 30 mg, 60 mg and 90 mg, respectively. Dose-versus-placebo comparisons used GEE with a logit link, including previous glucose-lowering medication use, treatment, visit and the treatment-by-visit interaction as fixed effects and baseline HbA1c as a covariate, with participant as the repeated unit. The individual randomized participant was the independent unit of study; no technical replicates were used. All comparisons were nominal and unadjusted for multiplicity. For the <7.0% and ≤6.5% targets, all dose-versus-placebo comparisons had P < 0.0001. For the <5.7% target, P values for safiglipron 30 mg, 60 mg and 90 mg versus placebo were 0.0203, 0.0384 and 0.0045, respectively. GEE=generalized estimating equation. HbA1c=glycated hemoglobin. Source data

At week 32, under the treatment policy estimand, the HbA1c target of less than 7.0% was reached in 71.4%, 77.1% and 77.8% of participants in the safiglipron 30 mg, safiglipron 60 mg and safiglipron 90 mg groups, respectively, compared to 25.0% in the placebo group (all *P* < 0.0001; Fig. 2c). An HbA1c level of 6.5% or less was reached in 58.6%, 64.3% and 68.1% of participants, respectively, compared to 16.7% in the placebo group (all *P* < 0.0001; Fig. 2c and Supplementary Fig. 2a). In prespecified supportive analyses under the efficacy estimand, model-estimated responder rates for the corresponding thresholds were 83.5−89.6% and 62.8−85.0%, respectively, across safiglipron dose groups (Extended Data Fig. 3 and Supplementary Fig. 2b).

#### FPG

Under the treatment policy estimand, FPG was reduced to a greater extent with all safiglipron doses than with placebo at week 32. The estimated treatment differences versus placebo were −1.58 mmol l^−1^ (95% CI, −2.15 to −1.00) for safiglipron 30 mg, −1.68 mmol l^−1^ (95% CI, −2.27 to −1.09) for safiglipron 60 mg and −2.08 mmol l^−1^ (95% CI, −2.66 to −1.50) for safiglipron 90 mg (all *P* < 0.0001; Table 2). Findings under the supportive efficacy estimand using a hypothetical strategy for intercurrent events were consistent (Supplementary Table 4).

**Table 2:** Primary and confirmatory secondary efficacy endpoints at week 32 (treatment policy estimand)

Endpoint	Safiglipron 30 mg (*N* = 70)	Safiglipron 60 mg (*N* = 70)	Safiglipron 90 mg (*N* = 72)	Placebo (*N* = 72)
HbA1c, %				
LS mean change from baseline (95% CI)	−1.40 (−1.61 to −1.18)	−1.38 (−1.60 to −1.16)	−1.63 (−1.84 to −1.41)	−0.18 (−0.40 to 0.05)
Difference versus placebo (95% CI); *P* value	−1.22 (−1.53 to −0.91); *P* < 0.0001	−1.20 (−1.52 to −0.89); *P* < 0.0001	−1.45 (−1.75 to −1.14); *P* < 0.0001	NA
Participants achieving HbA1c < 7.0%				
Proportion of participants, % (95% CI)	71.4 (59.38−81.60)	77.1 (65.55−86.33)	77.8 (66.44−86.73)	25.0 (15.54−36.60)
Absolute difference versus placebo, percentage points (95% CI)	46.4 (31.87−60.99)	52.1 (38.11−66.17)	52.8 (38.91−66.64)	NA
Odds ratio versus placebo (95% CI); *P* value	17.17 (5.96 to 49.50); *P* < 0.0001	29.51 (9.85 to 88.39); *P* < 0.0001	27.76 (10.01 to 77.04); *P* < 0.0001	NA
Participants achieving HbA1c ≤ 6.5%				
Proportion of participants, % (95% CI)	58.6 (46.17−70.23)	64.3 (51.93−75.39)	68.1 (56.01−78.56)	16.7 (8.92−27.30)
Absolute difference versus placebo, percentage points (95% CI)	41.9 (27.51−56.30)	47.6 (33.47−61.76)	51.4 (37.60−65.18)	NA
Odds ratio versus placebo (95% CI); *P* value	9.70 (3.82−24.61); *P* < 0.0001	16.75 (6.28−44.66); *P* < 0.0001	21.52 (8.18−56.61); *P* < 0.0001	NA
FPG, mmol l^−1^				
LS mean change from baseline (95% CI)	−1.90 (−2.32 to −1.47)	−1.99 (−2.44 to −1.55)	−2.40 (−2.84 to −1.96)	−0.32 (−0.77 to 0.13)
Difference versus placebo (95% CI); *P* value	−1.58 (−2.15 to −1.00); *P* < 0.0001	−1.68 (−2.27 to −1.09); *P* < 0.0001	−2.08 (−2.66 to −1.50); *P* < 0.0001	NA
Body weight, % change from baseline				
LS mean change from baseline (95% CI)	−1.87 (−3.04 to −0.69)	−3.45 (−4.64 to −2.25)	−4.78 (−6.08 to −3.47)	−1.22 (−2.42 to −0.02)
Difference versus placebo (95% CI); *P* value	−0.65 (−2.21 to 0.91); *P* = 0.21	−2.23 (−3.80 to −0.65); *P* = 0.0028	−3.56 (−5.22 to −1.89); *P* < 0.0001	NA

Analyses were based on the treatment policy estimand. *N* denotes the number of participants in the intention-to-treat analysis population. The numbers of participants with available week 32 HbA1c, FPG and body weight measurements were *n* = 67, 62, 65 and 62 in the safiglipron 30 mg, safiglipron 60 mg, safiglipron 90 mg and placebo groups, respectively. For continuous endpoints, differences versus placebo are least squares mean differences. Continuous endpoints were analyzed using ANCOVA with retrieved dropout data incorporated. For binary endpoints, response rates and exact 95% CIs were calculated using the imputed data. Absolute differences versus placebo are percentage point differences with 95% CIs based on the normal approximation. Odds ratios and corresponding *P* values were estimated using GEE. Data are least squares mean changes from baseline (95% CI), differences versus placebo (95% CI), response rates (95% CI), absolute percentage point differences versus placebo (95% CI) or odds ratios versus placebo (95% CI), as applicable. All 95% CIs are two-sided. All *P* values are one-sided and compare each safiglipron dose to placebo. The prespecified fixed-sequence testing procedure controlled the family-wise type I error rate at a one-sided *α* of 0.025.

LS, least squares;

NA, not applicable.

#### Body weight change

Body weight decreased from baseline in all groups, with larger reductions observed in the safiglipron 60 mg and safiglipron 90 mg groups than in the placebo group (Fig. 2e). Under the treatment policy estimand, the least squares mean percentage changes from baseline to week 32 were −1.87% (95% CI, −3.04 to −0.69) with safiglipron 30 mg, −3.45% (95% CI, −4.64 to −2.25) with safiglipron 60 mg and −4.78% (95% CI, −6.08 to −3.47) with safiglipron 90 mg compared to −1.22% (95% CI, −2.42 to −0.02) with placebo. The estimated treatment differences versus placebo were −0.65% (95% CI, −2.21 to 0.91, *P* = 0.21), −2.23% (95% CI, −3.80 to −0.65, *P* = 0.0028) and −3.56% (95% CI, −5.22 to −1.89, *P* < 0.0001), respectively. Within the prespecified fixed-sequence testing procedure, superiority to placebo was confirmed for the safiglipron 90 mg and safiglipron 60 mg doses; the procedure stopped at the safiglipron 30 mg comparison, which was not statistically significant. Findings were similar in prespecified supportive analyses under the efficacy estimand (Fig. 2f).

### Supportive secondary outcomes

#### Indices of β cell function and insulin sensitivity

**Fig. 3:**
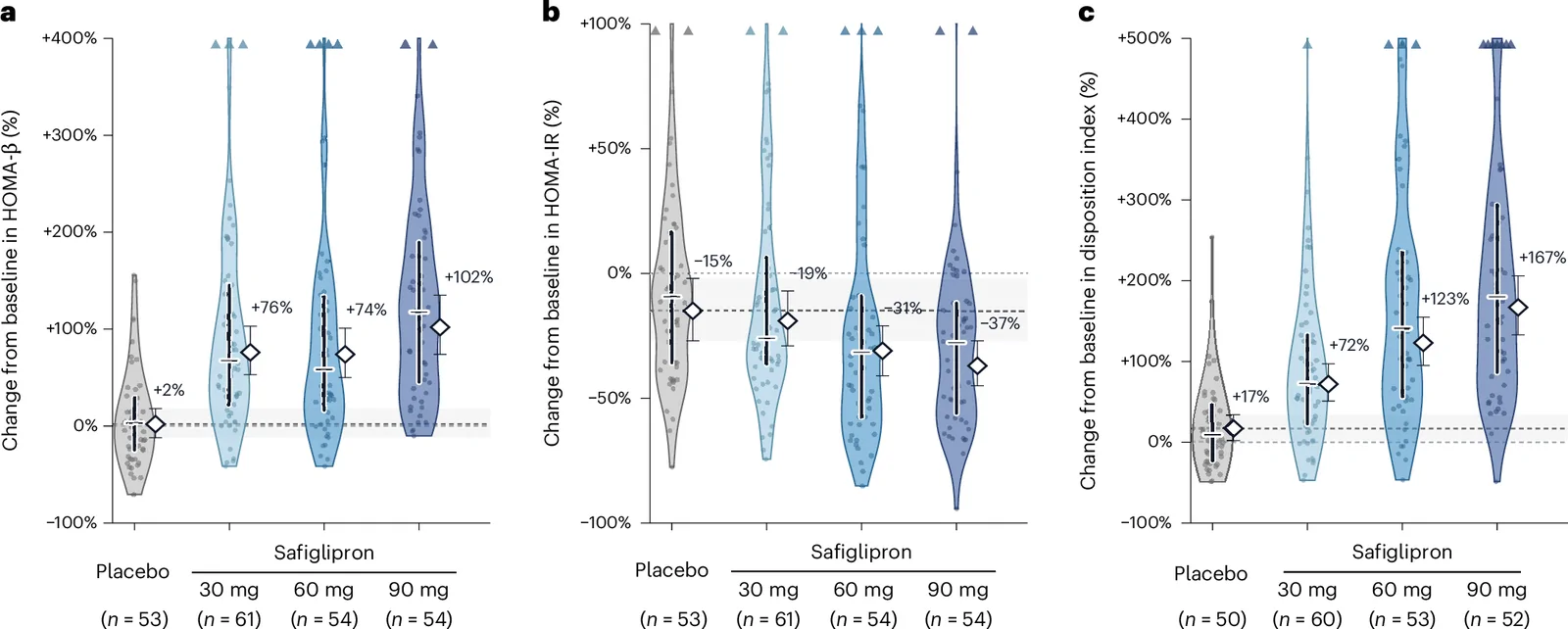
Indices of β cell function and insulin sensitivity at week 32. **a**, Geometric mean percentage change from baseline to week 32 in HOMA-β. **b**, Geometric mean percentage change from baseline to week 32 in HOMA-IR. **c**, Geometric mean percentage change from baseline to week 32 in disposition index (ISSI-2). For **a**−**c**, analyses were done in the intention-to-treat population under the supportive efficacy estimand; error bars indicate 95% CIs. HOMA-β and HOMA-IR were calculated from fasting glucose and fasting insulin measured before the MMTT. Disposition index was calculated as the C-peptide-based ISSI-2 defined as the ratio of the C-peptide to glucose areas under the curve during the MMTT multiplied by the Matsuda insulin sensitivity index. Source data

Postprandial glucose decreased dose dependently, and insulin secretory markers increased with safiglipron relative to placebo during the MMTT (Supplementary Table 4). At week 32, HOMA-β increased by 76%, 74% and 102% from baseline in the safiglipron 30 mg, safiglipron 60 mg and safiglipron 90 mg groups, respectively, compared to 2% in the placebo group. The corresponding geometric mean ratios versus placebo were 1.73 (95% CI, 1.43−2.09), 1.71 (95% CI, 1.41−2.08) and 1.99 (95% CI, 1.63−2.41) (Fig. 3a). HOMA-IR decreased by 19%, 31% and 37% from baseline with safiglipron 30 mg, safiglipron 60 mg and safiglipron 90 mg, respectively, compared to 15% in the placebo group, with geometric mean ratios versus placebo of 0.96 (95% CI, 0.79−1.16), 0.81 (95% CI, 0.67−0.98) and 0.75 (95% CI, 0.61−0.91), respectively (Fig. 3b). The disposition index (insulin secretion-sensitivity index-2 (ISSI-2)) increased by 72%, 123% and 167% from baseline with safiglipron 30 mg, safiglipron 60 mg and safiglipron 90 mg, respectively, versus 17% with placebo (Fig. 3c).

#### Other supportive endpoints

Seven-point SMBG profiles showed greater reductions in mean daily, pre-meal and 2-hour post-meal glucose values with safiglipron than with placebo at week 32 (Fig. 2d). An HbA1c level of less than 5.7% was reached in 12.9%, 8.6% and 16.7% of participants, respectively, compared to 1.4% in the placebo group (Fig. 2c and Supplementary Fig. 2a). Under the supportive efficacy estimand, model-estimated responder rates for the corresponding thresholds were 7.8−16.7% across safiglipron dose groups (Extended Data Fig. 3 and Supplementary Fig. 2b). No participant in any safiglipron group received rescue therapy for persistent hyperglycemia during the 32-week placebo-controlled period compared to seven (9.7%) participants in the placebo group. Under the treatment policy estimand, the least squares mean differences versus placebo in waist circumference at week 32 were 0.08 cm (95% CI, −1.17 to 1.33), −1.26 cm (95% CI, −2.52 to 0.01) and −1.78 cm (95% CI, −3.08 to −0.48) with safiglipron 30 mg, safiglipron 60 mg and safiglipron 90 mg, respectively, with consistent findings under the supportive efficacy estimand (Supplementary Table 4).

#### Treatment satisfaction

At week 32, DTSQs scores increased from baseline in all groups, with numerically greater improvements in safiglipron-treated participants (+1.4 to +2.8 points) than with placebo (+1.0 point). Perceived hyperglycemia scores decreased from baseline with safiglipron (−0.7 to −1.4 points) but increased slightly with placebo (+0.2 points); full results are shown in Supplementary Table 9.

#### Week 52 efficacy outcomes

Efficacy outcomes at week 52 are shown in Supplementary Tables 6–8 and Supplementary Fig. 1. These included model-based and observed-case analyses of glycemic measures, body weight and waist circumference, HbA1c target achievement, seven-point SMBG profiles and indices of β cell function and insulin sensitivity in the continuous safiglipron groups and in participants initially assigned to placebo who switched to safiglipron 30 mg from week 33.

### Safety

During the 32-week core treatment period, treatment-emergent adverse events (TEAEs) were reported in 59 (84.3%) participants in the safiglipron 30 mg group, in 60 (85.7%) participants in the safiglipron 60 mg group, in 65 (90.3%) participants in the safiglipron 90 mg group and in 56 (77.8%) participants in the placebo group (Table 3). Most adverse events were mild or moderate in severity. Serious adverse events occurred in seven (10.0%), two (2.9%) and five (6.9%) participants in the safiglipron 30 mg, safiglipron 60 mg and safiglipron 90 mg groups, respectively, compared to one (1.4%) participant in the placebo group (Supplementary Table 10). TEAEs led to discontinuation of study treatment in one (1.4%), two (2.9%) and five (6.9%) participants in the safiglipron 30 mg, safiglipron 60 mg and safiglipron 90 mg groups, respectively, and in no participants in the placebo group; most discontinuations in the safiglipron groups were due to gastrointestinal events (zero, two (2.9%) and three (4.2%), respectively) (Extended Data Table 1). No deaths occurred during the trial.

**Table 3:** TEAEs during the 32-week placebo-controlled period in the safety population

	Safiglipron 30 mg (*N* = 70)	Safiglipron 60 mg (*N* = 70)	Safiglipron 90 mg (*N* = 72)	Placebo (*N* = 72)
Overview of adverse events				
Any TEAE	59 (84.3)	60 (85.7)	65 (90.3)	56 (77.8)
Maximum severity of TEAE				
Mild	30 (42.9)	38 (54.3)	32 (44.4)	30 (41.7)
Moderate	27 (38.6)	21 (30.0)	31 (43.1)	25 (34.7)
Severe	2 (2.9)	1 (1.4)	2 (2.8)	1 (1.4)
TEAE considered related to study treatment	50 (71.4)	49 (70.0)	62 (86.1)	23 (31.9)
Serious TEAE	7 (10.0)	2 (2.9)	5 (6.9)	1 (1.4)
Death	0	0	0	0
TEAE leading to study treatment discontinuation	1 (1.4)	2 (2.9)	5 (6.9)	0
Gastrointestinal TEAE leading to study drug discontinuation	0	2 (2.9)	3 (4.2)	0
Prespecified adverse events of special interest^a^				
Any prespecified adverse event of special interest	1 (1.4)	0	0	0
Clinically significant hepatic enzyme abnormality^b^	0	0	0	0
Clinically significant calcitonin abnormality^c^	0	0	0	0
Severe hypoglycemia^d^	0	0	0	0
Acute pancreatitis	1 (1.4)	0	0	0
TEAEs reported in ≥5% of participants in any group				
Nausea	18 (25.7)	25 (35.7)	33 (45.8)	5 (6.9)
Diarrhea	15 (21.4)	14 (20.0)	21 (29.2)	3 (4.2)
Decreased appetite	6 (8.6)	14 (20.0)	18 (25.0)	2 (2.8)
Vomiting	9 (12.9)	13 (18.6)	16 (22.2)	0
Hyperuricemia	8 (11.4)	8 (11.4)	12 (16.7)	3 (4.2)
Abdominal distension	3 (4.3)	5 (7.1)	11 (15.3)	0
Asthenia	0	5 (7.1)	9 (12.5)	0
Lipase increased	5 (7.1)	3 (4.3)	7 (9.7)	0
Upper respiratory tract infection	10 (14.3)	7 (10.0)	6 (8.3)	11 (15.3)
Flatulence	2 (2.9)	3 (4.3)	6 (8.3)	0
Urinary tract infection	2 (2.9)	2 (2.9)	6 (8.3)	4 (5.6)
Blood bilirubin increased	5 (7.1)	1 (1.4)	6 (8.3)	3 (4.2)
Dizziness	3 (4.3)	7 (10.0)	4 (5.6)	1 (1.4)
ALT increased	3 (4.3)	3 (4.3)	4 (5.6)	4 (5.6)
Upper abdominal pain	1 (1.4)	3 (4.3)	4 (5.6)	2 (2.8)
Abdominal pain	1 (1.4)	3 (4.3)	4 (5.6)	0
Hyperlipidemia	2 (2.9)	3 (4.3)	3 (4.2)	4 (5.6)
Protein urine present	0	0	3 (4.2)	4 (5.6)
Dyspepsia	4 (5.7)	2 (2.9)	2 (2.8)	1 (1.4)
Blood uric acid increased	1 (1.4)	4 (5.7)	0	1 (1.4)
Proteinuria	1 (1.4)	1 (1.4)	0	4 (5.6)

Data are *n* (%). The safety population included all randomly assigned participants who received at least one dose of study drug. Percentages were calculated using the number of participants in each treatment group as the denominator. TEAEs were adverse events that occurred or worsened after the first dose of study drug and during the 32-week placebo-controlled period. Maximum severity refers to the highest severity reported for each participant. Study treatment-related TEAEs were events considered by the investigator to be related to study treatment. TEAEs reported in ≥5% of participants in any treatment group are listed by Medical Dictionary for Regulatory Activities preferred term.

^a^Prespecified adverse events of special interest were clinically significant hepatic enzyme abnormalities, clinically significant calcitonin abnormalities, acute pancreatitis and severe hypoglycemia.

^b^Clinically significant hepatic enzyme abnormalities were protocol-defined elevations in ALT or AST and total bilirubin meeting prespecified thresholds in the absence of alternative explanations or evidence of cholestasis; full criteria are provided in the study protocol.

^c^Clinically significant calcitonin abnormality was defined as post-baseline calcitonin concentration of at least 50 ng l^−1^ and an increase of more than 50% from baseline.

^d^Severe hypoglycemia was defined according to American Diabetes Association level 3 criteria as severe cognitive or physical impairment requiring external assistance for recovery, without a specific glucose threshold.

**Extended Data Fig. 4:**
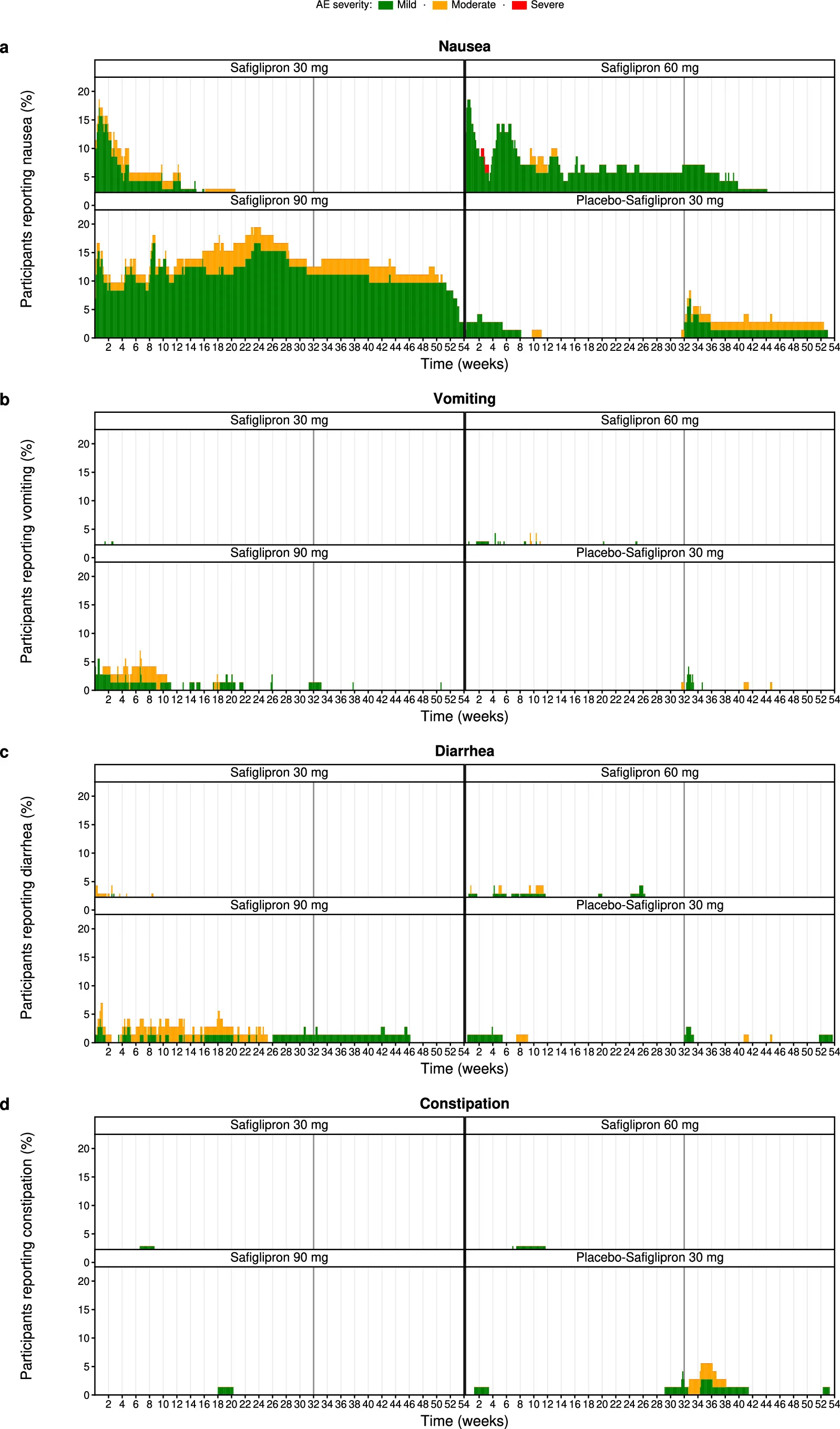
Daily prevalence and severity of selected gastrointestinal treatment-emergent adverse events through week 52. **a**, Nausea. **b**, Vomiting. **c**, Diarrhea. **d**, Constipation. The 52-week treatment period comprised a 32-week placebo-controlled core period and a 20-week extension period; participants initially assigned to placebo switched to safiglipron 30 mg at week 33. In each panel, each vertical bar represents one study day and shows the proportion of participants in the safety population with an ongoing treatment-emergent event of the specified preferred term on that day, with color indicating severity (green, mild; yellow, moderate; red, severe). A participant was counted once per preferred term on each study day. Percentages were calculated using the number of participants in each treatment group as the denominator. Events were counted irrespective of relationship to study treatment. The dashed vertical lines denote week 32, the end of the placebo-controlled core period. Events are listed by Medical Dictionary for Regulatory Activities preferred term. Source data

Gastrointestinal adverse events were the most frequently reported class of events, occurring in 31 (44.3%) participants in the safiglipron 30 mg group, in 42 (60.0%) participants in the safiglipron 60 mg group and in 53 (73.6%) participants in the safiglipron 90 mg group compared to 11 (15.3%) participants in the placebo group. Most gastrointestinal adverse events occurred during the dose titration period. The time courses and severity distributions of nausea, vomiting, diarrhea and constipation are shown in Extended Data Fig. 4 and Supplementary Table 11.

During the 32-week core period, no severe hypoglycemia was reported. Hepatic transaminase levels showed dose-related decreases from baseline (Supplementary Table 17), and no participant met Hy’s law laboratory criteria during the core period (Extended Data Table 2). Acute pancreatitis, a prespecified adverse event of special interest, was reported in one participant, in the safiglipron 30 mg group (one (1.4%)). Diabetic retinopathy stage worsened in six (2.8%) participants receiving safiglipron overall and in five (6.9%) participants receiving placebo, whereas improvement was observed in six (2.8%) and one (1.4%), respectively (Supplementary Table 12).

Cumulative safety findings through week 52, including data from the 20-week extension period after participants initially assigned to placebo switched to safiglipron 30 mg at week 33, are provided in Supplementary Tables 13–19.

### Exploratory outcomes

#### Cardiometabolic and renal risk markers

**Extended Data Fig. 5:**
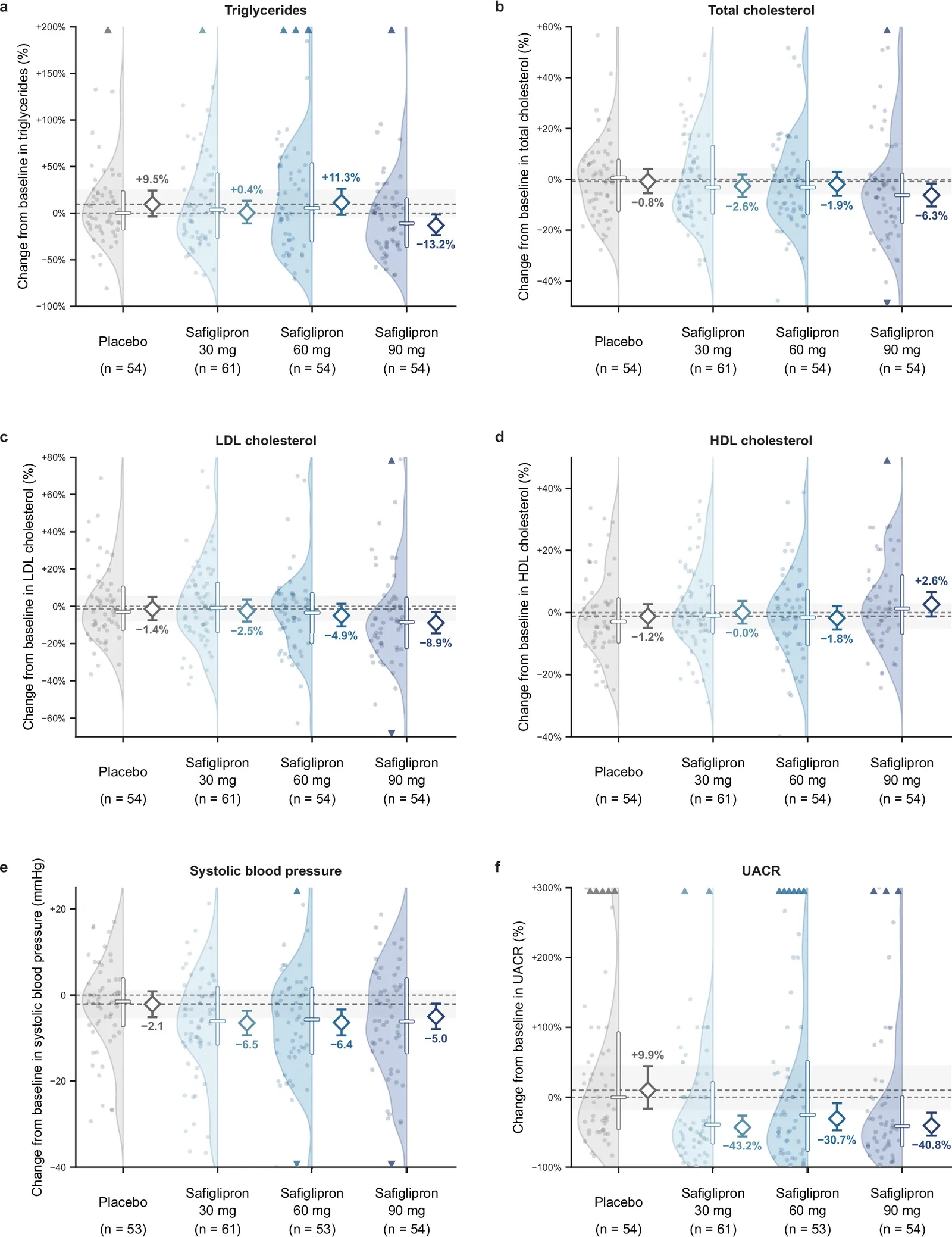
Changes from baseline in cardiometabolic outcomes at week 32. **a**, Geometric mean percentage change from baseline in triglycerides. **b**, Geometric mean percentage change from baseline in total cholesterol. **c**, Geometric mean percentage change from baseline in LDL cholesterol. **d**, Geometric mean percentage change from baseline in HDL cholesterol. **e**, Least-squares mean change from baseline in systolic blood pressure. **f**, Geometric mean percentage change from baseline in urinary albumin-to-creatinine ratio. Dots show individual observed values among participants with available week-32 measurements; half-violin plots show their distributions, and the internal boxplots show the medians and interquartile ranges. Triangles at the upper or lower plot boundaries indicate observed values outside the displayed y-axis range. Diamonds and error bars show the model-estimated changes from baseline and their 95% CIs. The horizontal dashed lines indicate no change from baseline and the corresponding placebo estimate. Values beneath the treatment-group labels are the numbers of participants with available week-32 observations. Analyses were done in the intention-to-treat population under the supportive efficacy estimand using MMRM. Lipid parameters and urinary albumin-to-creatinine ratio were analyzed after natural-log transformation and are shown as geometric mean percentage changes from baseline. Systolic blood pressure is shown as least-squares mean change from baseline. Error bars indicate 95% CIs. CI=confidence interval. HDL=high-density lipoprotein. LDL=low-density lipoprotein. MMRM=mixed model for repeated measures. UACR=urinary albumin-to-creatinine ratio. Source data

In the safiglipron 90 mg group, triglycerides decreased by 13.2%, low-density lipoprotein (LDL) cholesterol by 8.9% and total cholesterol by 6.3% from baseline; effects at lower doses were less consistent (Extended Data Fig. 5). Systolic blood pressure decreased with safiglipron, with differences versus placebo of −4.37 mmHg (95% CI, −8.35 to −0.38) for 30 mg, −4.24 mmHg (95% CI, −8.34 to −0.14) for 60 mg and −2.85 mmHg (95% CI, −6.91 to 1.22) for 90 mg (Extended Data Fig. 5 and Supplementary Table 5). UACR decreased with all doses, with percentage differences versus placebo of −48.3% (95% CI, −64.0 to −25.9) for 30 mg, −36.9% (95% CI, −56.5 to −8.5) for 60 mg and −46.1% (95% CI, −62.8 to −22.1) for 90 mg (Extended Data Fig. 5 and Supplementary Table 5).

#### Mediation analyses

**Extended Data Fig. 6:**
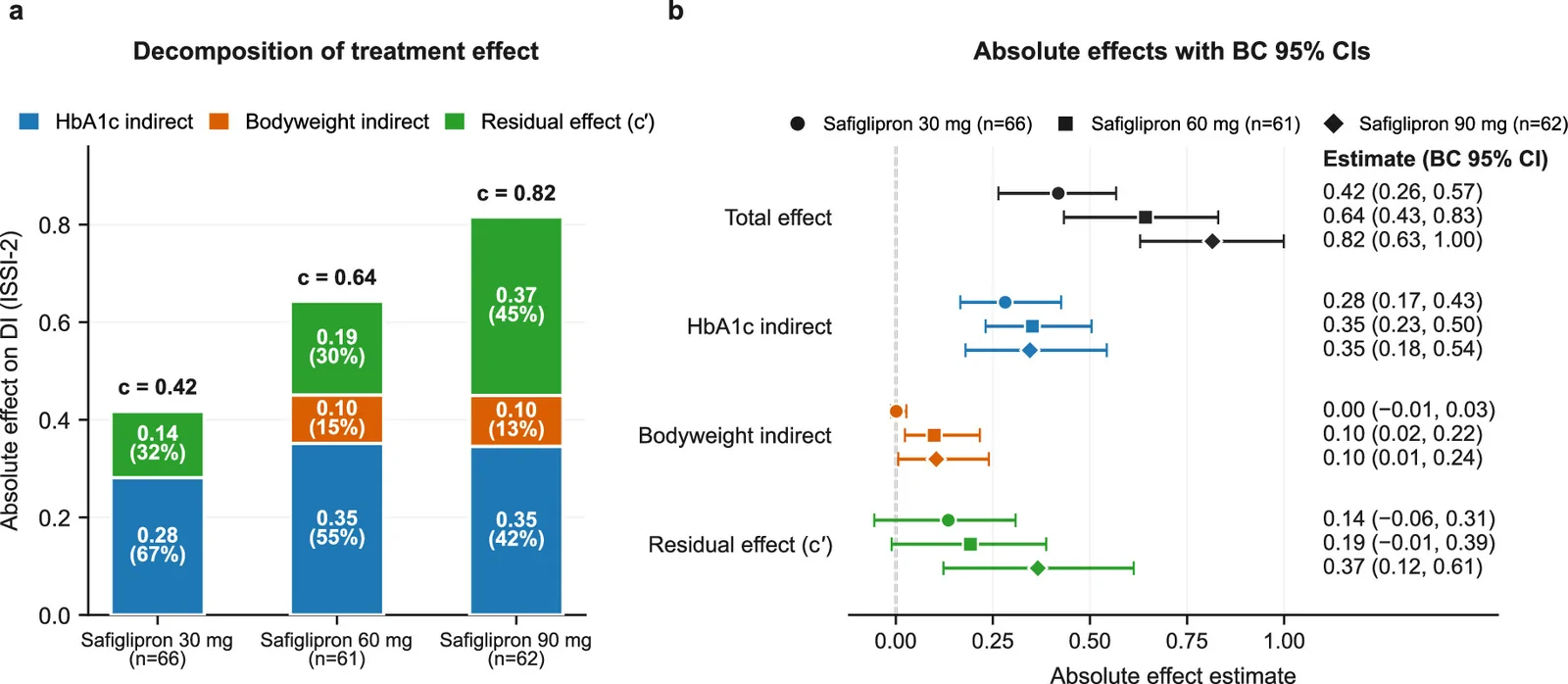
Mediation analysis of the effects of safiglipron on disposition index at week 32. **a**, Decomposition of the treatment effect of each safiglipron dose versus placebo on DI (ISSI-2) into the indirect effect mediated through HbA1c, the indirect effect mediated through bodyweight and the residual effect (c′). Values within the bars show the estimated absolute effects and, in parentheses, the percentages of the total treatment effect mediated through the corresponding pathways. Values above the bars show the total treatment effect (c). **b**, Symbols show point estimates of the total, HbA1c-mediated indirect, bodyweight-mediated indirect and residual effects; horizontal error bars indicate two-sided bias-corrected 95% bootstrap CIs. The dashed vertical line indicates no effect. The n values shown below the dose labels in a and in the key in b denote complete-case participants in the corresponding safiglipron groups; the same 58 placebo participants contributed to all three comparisons. CIs were derived from 10,000 participant-level bootstrap resamples per comparison. The individual randomized participant was the unit of analysis. Inference was based on bootstrap CIs; no P values were calculated. Results are from the prespecified exploratory mediation analysis. BC=bias-corrected. CI=confidence interval. DI=disposition index. HbA1c=glycated hemoglobin. ISSI-2=insulin secretion-sensitivity index-2. Source data

In prespecified exploratory mediation analyses conducted separately for each dose, the total effect of safiglipron on disposition index increased across dose groups on the log-ratio scale: 0.42 (95% bias-corrected bootstrap CI, 0.26−0.57) with 30 mg, 0.64 (0.43−0.83) with 60 mg and 0.82 (0.63−1.00) with 90 mg. The estimated indirect effects through HbA1c reduction accounted for 67%, 55% and 42% of the total effect, whereas those through body weight reduction accounted for less than 1%, 15% and 13%, respectively. The residual component was 0.14 (32%; 95% bias-corrected bootstrap CI, −0.06 to 0.31), 0.19 (30%; −0.01 to 0.39) and 0.37 (45%; 0.12−0.61), respectively (Extended Data Fig. 6).

### Sensitivity analyses

The primary endpoint result was consistent across all prespecified sensitivity analyses, including last-observation-carried-forward imputation and tipping point analysis (Supplementary Table 3).

## Discussion

In OUTSTAND-1, once-daily oral safiglipron produced clinically meaningful improvements in glycemic control across all three evaluated doses in Chinese adults with type 2 diabetes managed with diet and exercise alone. Confirmatory testing showed that all three doses produced greater reductions in HbA1c and FPG and higher HbA1c target achievement rates than placebo; the 60 mg and 90 mg doses also produced significant reductions in body weight relative to placebo. Together, these findings demonstrate clinically meaningful glycemic efficacy across the evaluated dose range, with incremental body weight effects at higher doses.

The magnitude of glycemic lowering places safiglipron within the range reported for oral GLP-1 receptor agonist monotherapy. In similar monotherapy populations with a mean baseline HbA1c of approximately 8.0%, placebo-adjusted HbA1c reductions were 0.6−1.1% at week 26 in PIONEER 1 across oral semaglutide doses, whereas those in ACHIEVE-1 were 0.83−1.07% at week 40 across orforglipron doses; safiglipron produced reductions of 1.20−1.45% at week 32 (refs. ^6,8^). These cross-trial comparisons provide clinical context rather than evidence of comparative efficacy, because differences in study design, population, dosing strategy and placebo response preclude formal ranking. ACHIEVE-3 subsequently demonstrated that efficacy can differ between individual oral GLP-1 receptor agonists in a direct randomized comparison, underscoring the need for molecule-specific and dose-specific comparisons rather than assumptions based on route of administration or molecular class alone^10^.

At the 90-mg dose, safiglipron produced body weight reductions broadly similar to those reported with injectable semaglutide 1 mg, oral semaglutide 14 mg and orforglipron 12 mg in the respective monotherapy trials but smaller than that reported with orforglipron 36 mg, although differences in baseline BMI and ethnic composition across trials limit direct comparison^6,8,16^. The 30−90-mg dose range in OUTSTAND-1 was selected on the basis of phase 2 findings^13^ to characterize glycemic efficacy in type 2 diabetes rather than to maximize weight reduction; higher doses of safiglipron (120 mg and 180 mg) have been evaluated for weight management in the HARBOR-1 phase 3 trial (NCT06904105). At the lower end of this range, safiglipron 30 mg produced glycemic improvements broadly similar to those at higher doses, whereas the body weight difference versus placebo was small and not statistically significant. This profile may be relevant to patients for whom marked weight loss is not a treatment priority, including some patients in East Asian populations, where type 2 diabetes often occurs at lower BMI, and β cell dysfunction contributes prominently to pathophysiology^17–19^. For such patients, effective glycemic control without concomitant weight loss might be desirable. The differing dose−response relationships for glycemia and body weight could, therefore, support dose selection according to individual glycemic and weight management goals, although this application requires prospective evaluation.

Reductions from baseline in HbA1c and body weight remained evident at week 52, although some attenuation in HbA1c reduction and partial body weight regain occurred during the extension period. Several factors may have contributed to these trajectories. Physiological counter-regulation or metabolic adaptation during sustained treatment may have attenuated the initial response. In addition, study visits were less frequent during the extension period, potentially reducing adherence support, and the extension coincided with the winter months for most participants, when seasonal changes in diet and physical activity may have affected body weight. However, because participants initially assigned to placebo switched to safiglipron 30 mg at week 33, no concurrent placebo comparison was available during the extension, and the relative contributions of these potential factors cannot be determined. Longer-term controlled follow-up is needed to further characterize the durability of treatment effects.

In this trial, the study population had a median diabetes duration of 1.6 years, with two-thirds of participants having no history of glucose-lowering medication use. Safiglipron was evaluated as monotherapy without a metformin-first requirement, providing a setting in which treatment-related metabolic changes could be assessed with limited interference from concomitant therapy. Prespecified supportive on-treatment assessments showed progressively greater increases in the disposition index (ISSI-2) across safiglipron dose groups. Prespecified exploratory mediation analyses estimated a residual component of the ISSI-2 effect not statistically explained by concurrent changes in HbA1c or body weight; this component increased numerically across dose groups, paralleling the dose-related pattern in the total effect on ISSI-2. This pattern contrasts with the more limited dose differentiation in HbA1c reduction and is compatible with dose-related on-treatment pharmacodynamic effects, including insulinotropic stimulation. However, because ISSI-2 was measured during treatment with an insulinotropic agent, these findings cannot distinguish direct on-treatment pharmacological effects from changes in intrinsic β cell function. Whether these changes persist after treatment withdrawal or translate into durable preservation or improvement of β cell function requires off-treatment follow-up.

Prespecified exploratory analyses suggested favorable changes in selected cardiometabolic and renal risk markers. Lipid parameters improved primarily with safiglipron 90 mg, including reductions in triglycerides, total cholesterol and LDL cholesterol. Placebo-adjusted reductions were approximately 3−4 mmHg for systolic blood pressure and 37−48% for UACR across dose groups, with no clear dose gradient for either marker. The absence of dose−response relationships for both parameters might reflect floor effects in this population with predominantly normal baseline blood pressure and low baseline UACR. By contrast, a clear dose-dependent reduction in UACR was observed in the phase 2 SOLID-DKD trial of safiglipron in patients with diabetic kidney disease and macroalbuminuria^15^, consistent with a post hoc analysis of SUSTAIN 6, suggesting that UACR dose differentiation may be more apparent at higher baseline albuminuria levels^20^. The clinical significance of these UACR changes at low baseline levels remains to be established^21^. Whether safiglipron confers long-term kidney protection is being evaluated in the phase 3 RENEW-CKD outcome trial (NCT07625774).

Safiglipron was generally well tolerated at all three doses. Gastrointestinal adverse events were the most common events and increased with dose. Most gastrointestinal events were mild or moderate. Adverse-event-related treatment discontinuations also increased with dose. Nausea peaked after treatment initiation and at each dose-escalation step, and, although its prevalence declined within each titration interval, a residual level of nausea persisted after the target dose was reached and was more prevalent at higher doses. This pattern was broadly similar to the time courses reported with orforglipron and aleniglipron^8,22^. Vomiting showed a clearer decline after titration. Diarrhea was associated with a modest increase in prevalence at higher doses but a more apparent increase in severity. Constipation was infrequent and showed no consistent temporal or dose-related pattern. Collectively, these time courses may differ from those typically described with peptide-based GLP-1 receptor agonists; whether these apparent differences reflect variation in molecular properties, trial populations, dosing or event ascertainment cannot be determined without head-to-head comparisons.

Beyond these gastrointestinal findings, no severe hypoglycemia or deaths occurred. No hepatic safety signal was identified, and no participant met Hy’s law laboratory criteria. Mean transaminase levels decreased from baseline across safiglipron groups, with larger reductions at higher doses. Whether these changes reflect a direct hepatic effect or are secondary to improvements in metabolic health requires further investigation in studies incorporating dedicated hepatic assessments.

This trial has several limitations. First, all participants were Chinese, and the findings might not be directly generalizable to other ethnic groups or to populations with higher BMI, longer diabetes duration or more advanced complications. Second, the sample size was powered for glycemic efficacy, not for rare adverse events or cardiovascular and renal outcomes. Third, the trial was not designed with a predefined treatment withdrawal period, and whether the observed changes in on-treatment β cell function indices persist after discontinuation of safiglipron cannot be determined from the present data. Strengths include the monotherapy design in participants managed with diet and exercise alone, which minimized interference from background glucose-lowering therapy. The high overall trial follow-up completion rate (91.9%) also minimized concerns about attrition.

In conclusion, once-daily oral safiglipron produced clinically meaningful glycemic control and body weight reductions that were greater at higher doses, alongside favorable trends in selected cardiometabolic and renal risk markers, in Chinese adults early in the course of type 2 diabetes managed with diet and exercise alone. Overall safety and tolerability were acceptable. These findings support safiglipron as an effective oral treatment option in this early-disease population and motivate further mechanistic studies with longer-term follow-up.

## Methods

### Study design and participants

The OUTSTAND-1 trial complied with all relevant ethical regulations and was conducted in accordance with the Declaration of Helsinki and Good Clinical Practice guidelines; its protocol, amendment and informed consent materials were approved by the Ethics Committee of Peking Union Medical College Hospital (the coordinating center) and by the institutional review board or independent ethics committee of each of the 46 participating hospitals named in Supplementary Table 1.

The OUTSTAND-1 trial was a multicenter, randomized, double-blind, placebo-controlled, phase 3 trial conducted at 46 hospitals in China. The protocol was amended once before database lock, primarily to extend the double-blind treatment period from 32 weeks to 52 weeks at the request of the regulatory authority, with consequential updates to endpoints, dosing and rescue treatment provisions. The HbA1c inclusion criterion was also relaxed from 7.5% to 7.0%. The full protocol and amendment history are provided with the submission. The trial was prospectively registered with Chinadrugtrials.org.cn (CTR20243559) in accordance with Chinese regulatory requirements before first participant enrollment; ClinicalTrials.gov registration (NCT06672172) was subsequently completed after enrollment had begun to improve the international accessibility of the trial record.

Potential participants were recruited by investigators at the 46 participating hospitals and assessed against protocol-defined eligibility criteria. Screening began on 25 September 2024; randomization occurred from 21 October 2024 to 31 March 2025; and the last participant completed trial follow-up on 17 April 2026. All participants provided written informed consent before any trial procedures. Participants received transportation reimbursement and compensation for blood collection, as specified in the informed consent materials approved by the ethics committee. Participants were enrolled regardless of sex. Sex was recorded from the national identification document used for trial registration; gender identity was not assessed separately. Sex was a prespecified subgroup variable for the primary endpoint, although the trial was not powered for sex-specific treatment effect comparisons. Sex-disaggregated participant numbers are reported in Table 1.

### Inclusion criteria

Participants must meet all the following criteria to be enrolled in this trial:

1. Male or female, aged 18−75 years (inclusive) on the day of signing the informed consent form
2. Diagnosed with type 2 diabetes mellitus according to the Chinese Guidelines for the Prevention and Treatment of Type 2 Diabetes (2020 Edition), with a disease duration of ≥2 months at screening
3. Blood glucose was managed through consistent dietary control and exercise within 2 months prior to screening, without the use of any glucose-lowering agents.
4. HbA1c levels between 7.0% and 10.0% (inclusive) at screening (as tested by a local laboratory)
5. BMI of 19.0−40.0 kg m^−^^2^ (inclusive) at screening
6. Having voluntarily signed the informed consent form prior to trial initiation, participants must fully understand the study content, procedures and possible adverse reactions and must be able and willing to complete the study in accordance with protocol requirements (for example, self-monitoring of blood glucose and maintaining participant diaries).
7. From signing the informed consent form until 2 weeks after the last dose, participants have no plans for pregnancy and are willing to use highly effective contraception as specified in the protocol.

### Exclusion criteria

Participants who meet any of the following criteria shall not be enrolled in this trial:

Those with the following abnormalities at screening or randomization:

1. Presence of the following laboratory test abnormalities at screening or randomization:
  Fasting venous glucose >15 mmol l^−1^
  Alanine aminotransferase (ALT) or aspartate aminotransferase (AST) > 2× upper limit of normal (ULN)
  Total bilirubin > 1.5× ULN
  Amylase and/or lipase > 3× ULN
  Fasting triglycerides > 11.3 mmol l^−1^
  Calcitonin ≥ 50 ng l^−1^
  Estimated glomerular filtration rate (eGFR) < 30 ml min^−1^ 1.73 m^−2^ (calculated using the Chronic Kidney Disease Epidemiology Collaboration (CKD-EPI) equation; Supplementary Information, section 13.5)
  UACR ≥ 1,000 mg g^−1^
  Positive hepatitis B virus surface antigen (HBsAg) and HBV-DNA > ULN
2. Clinically significant abnormalities in electrocardiogram results at screening or randomization that may affect participant safety, including, but not limited to, supraventricular tachycardia, atrial fibrillation, atrial flutter and second-degree or third-degree atrioventricular block
3. During screening or randomization, the electrocardiogram examination results indicated a heart rate > 100 beats per minute or Fridericia-corrected QT interval (QTcF) > 480 ms. If the heart rate or QTcF is abnormal, two additional electrocardiograms may be performed to obtain the mean of three values.
4. Uncontrolled severe hypertension (systolic blood pressure ≥ 160 mmHg or diastolic blood pressure ≥ 100 mmHg) at screening or randomization
  Those with the following diseases or medical history prior to screening:
5. Diagnosed or suspected type 1 diabetes mellitus, special type of diabetes mellitus or secondary diabetes mellitus
6. Acute complications of diabetes (diabetic ketoacidosis, lactic acidosis, hyperglycemic hyperosmolar state, etc.) within 6 months prior to screening;
7. Severe hypoglycemic episodes or recurrent hypoglycemic episodes within 6 months prior to screening (≥3 hypoglycemic episodes within 1 week or other hypoglycemic episodes as determined by the investigator)
8. Proliferative retinopathy or maculopathy, painful diabetic neuropathy, diabetic foot ulcer or intermittent claudication requiring acute treatment at screening
9. Participants with known clinically significant diseases within 6 months prior to screening, including, but not limited to, neurological, psychiatric, cardiovascular, endocrine, gastrointestinal, respiratory, urinary, hematological, immune system and other diseases (except for type 2 diabetes-related conditions) that may interfere with the study results or pose additional risks to the administration of the study drug, as determined by the investigator
10. Past or known history of medullary thyroid carcinoma (MTC) or multiple endocrine neoplasia syndrome type 2 (MEN2) or family history thereof
11. Past or known medical history of acute or chronic pancreatitis, pancreatic injury, acute cholecystitis or symptomatic/treatment-requiring gallbladder disease (excluding participants who have undergone cholecystectomy and are determined by the investigator to be eligible for enrollment)
12. Past history of significant gastrointestinal diseases (such as gastric outlet obstruction, inflammatory bowel disease and active ulcer) or those who have undergone gastrointestinal surgery (except for gastrointestinal polypectomy and appendectomy) or those with a history of long-term use of medications that directly affect gastrointestinal motility
13. History of hyperthyroidism prior to screening or hyperthyroidism at screening or other thyroid disorders not adequately controlled (except for participants with thyroid hormone replacement treatment regimen stable for at least 8 weeks and no plan to change the treatment regimen during the study)
14. History of severe cardiovascular and cerebrovascular diseases within 6 months prior to screening, including, but not limited to, congestive heart failure (New York Heart Association (NYHA) class III−IV), unstable angina, stroke, myocardial infarction, severe arrhythmia or coronary revascularization or planned to undergo coronary, carotid or peripheral artery revascularization at screening
15. Participants with known or suspected depression, bipolar disorder, suicidal tendency, schizophrenia or other serious mental diseases or those with cognitive impairment or language barriers who are unable to fully understand the protocol or unwilling to cooperate with the study site staff
16. History of malignant neoplasm within 5 years prior to screening, except for cured local cancer, such as localized cutaneous basal cell carcinoma, cervical carcinoma in situ and carcinoma in situ of the prostate
  Those who have received any of the following drugs or treatments prior to screening:
17. Use of GLP-1 receptor agonists (including multi-target GLP-1 receptor agonists) within 3 months before screening
18. Use of systemic glucocorticoids (excluding those with cumulative use of <14 days or administration via topical, inhaled, intraocular or intranasal routes) within 3 months prior to the screening
19. Use of tricyclic antidepressants, atypical antipsychotics and mood stabilizers (such as imipramine, chlorpromazine, amitriptyline, mirtazapine, clozapine, olanzapine, paroxetine, phenelzine, thioridazine, valproic acid and its derivatives and lithium salts) within 3 months before screening
20. Weight loss therapy within 3 months prior to screening, including intensive diet or exercise therapy for weight loss, pharmacological therapy, surgical treatment (except for liposuction history more than 1 year), dietary supplements or other special therapies
  Those with any of the following conditions:
21. At screening or randomization, self-reported weight change exceeding ±5% within the previous 3 months or planned intensive diet or exercise therapy for weight loss during the trial (except for diabetes diet and exercise control measures)
22. Those who have participated in any clinical trial of drugs or medical devices within 3 months prior to screening or still within 5 half-lives of the study drug (whichever is longer). Participation in a clinical trial is defined as signing informed consent and having used the investigational product (including placebo) or investigational medical device.
23. Known or suspected hypersensitivity to the study drug or its components
24. Known or suspected history of drug or alcohol abuse and judged by the investigator as unsuitable for participation in this clinical trial
25. Participants with a history of blood donation or blood loss of ≥400 ml within 3 months prior to screening or who have received blood transfusion
26. Female participants who are pregnant, breastfeeding or planning to become pregnant during the trial
27. Intolerance to venipuncture or needle phobia
28. Any condition that, in the investigator’s judgment, makes the individual unsuitable for participation in this clinical trial

### Randomization and masking

Participants were randomly assigned in a 1:1:1:1 ratio to receive safiglipron 30 mg, safiglipron 60 mg, safiglipron 90 mg or placebo. Randomization was done centrally through an interactive web response system, using a block design stratified by baseline HbA1c (≤8.5% or >8.5%) and previous use of glucose-lowering medication (yes or no). Safiglipron and matching placebo tablets were identical in appearance to maintain masking. Participants, investigators, site staff, outcome assessors and sponsor personnel involved in trial conduct remained masked to treatment assignment during the double-blind treatment period. After completion of the 32-week placebo-controlled core period, participants initially assigned to placebo were switched to safiglipron 30 mg from week 33 for the extension period while maintaining the double-blind.

### Procedures

The trial included a screening period of up to 2 weeks, a 3-week single-blind placebo run-in period, a 52-week treatment period comprising a 32-week placebo-controlled core period and a 20-week extension period and a 2-week safety follow-up (Extended Data Fig. 1). Scheduled study visits occurred at baseline and at weeks 2, 4, 8, 12, 16, 20, 26, 32, 42 and 52.

Safiglipron was administered orally once daily, with no restrictions regarding food intake or dosing time. Participants assigned to safiglipron 60 mg or safiglipron 90 mg started at safiglipron 30 mg once daily, with dose escalation in 30-mg increments every 4 weeks until the assigned target dose was reached. Participants assigned to safiglipron 30 mg received their target dose without titration. Dose reduction or temporary treatment interruption was permitted for persistent gastrointestinal symptoms according to the protocol. Rescue therapy was permitted for persistent hyperglycemia according to prespecified FPG thresholds; metformin was the preferred rescue medication, whereas GLP-1 receptor agonists and DPP-4 inhibitors were not permitted.

At each visit, vital signs were measured; blood and urine samples were collected; and adverse events, concomitant medications and treatment adherence were recorded. A standardized MMTT was done at baseline, week 32 and week 52. After an overnight fast, participants consumed a standardized mixed meal within 10 minutes, and venous blood samples were collected at 0 minutes (fasting), 30 minutes, 60 minutes and 120 minutes for measurement of plasma glucose, serum insulin and serum C-peptide.

### Outcomes

The primary endpoint was change in HbA1c from baseline to week 32. The confirmatory secondary endpoints, all assessed at week 32, were the proportions of participants achieving HbA1c less than 7.0% and HbA1c of 6.5% or less, change from baseline in FPG and percentage change from baseline in body weight.

Prespecified supportive secondary endpoints assessed at both weeks 32 and 52 included changes from baseline in body weight, waist circumference, fasting serum insulin, fasting serum C-peptide, HOMA-β, HOMA-IR, MMTT-derived postprandial plasma glucose, serum insulin and serum C-peptide, disposition index and seven-point SMBG profiles as well as the proportions of participants achieving HbA1c less than 5.7% and receiving rescue therapy. Additional supportive secondary endpoints at week 52 included change from baseline in HbA1c, the proportions of participants achieving HbA1c less than 7.0% and HbA1c of 6.5% or less, change from baseline in FPG and percentage change from baseline in body weight. Prespecified exploratory endpoints assessed at weeks 32 and 52 included changes from baseline in systolic and diastolic blood pressure and percentage changes from baseline in triglycerides, total cholesterol, LDL cholesterol, high-density lipoprotein (HDL) cholesterol and UACR. Treatment satisfaction was assessed at baseline and at weeks 32 and 52 using the DTSQs.

Safety assessments included adverse events, hypoglycemic episodes, laboratory tests, vital signs, 12-lead electrocardiograms and diabetic retinopathy screening by fundus photography at weeks 32 and 52. Prespecified adverse events of special interest included clinically significant hepatic enzyme abnormalities, clinically significant calcitonin abnormalities, acute pancreatitis and severe hypoglycemia.

### Statistical analysis

The planned sample size was 272 participants, with 68 participants per group. Based on the phase 2 results, the treatment difference was conservatively assumed to be −0.85 percentage points among participants without intercurrent events and 0 percentage points among those with intercurrent events; assuming an intercurrent event rate of 15% yielded an overall treatment difference of −0.72 percentage points in HbA1c change from baseline to week 32 between each safiglipron dose and placebo. Assuming a standard deviation of 1.1 percentage points and a one-sided *α* of 0.025, this sample size provided approximately 96.8% power for each dose−placebo comparison and, assuming independence among the three comparisons, 90% overall power to show superiority of all three safiglipron doses versus placebo for the primary endpoint. Calculations used a one-sided two-sample *z*-test for a parallel-group difference in means in East version 6.5.4 software.

Efficacy analyses were done in the intention-to-treat population, defined as all randomized participants. In accordance with the International Council for Harmonisation (ICH) E9(R1) estimand framework^23^, two estimands were prespecified for the primary endpoint and confirmatory secondary endpoints. The primary treatment policy estimand assessed treatment effect regardless of intercurrent events, defined as premature study treatment discontinuation, insufficient exposure or initiation of supplementary glucose-lowering medication for more than 14 cumulative days; all available data were included irrespective of intercurrent event status. Missing data related to intercurrent events were imputed using a retrieved dropout approach or a copy reference approach if retrieved dropout imputation was not supported; remaining missing data were assumed missing at random. The supportive efficacy estimand applied a hypothetical strategy to all intercurrent events: post-intercurrent-event data were treated as missing, thereby estimating the treatment effect that would have been observed had these events not occurred. Complete prespecified estimand definitions and analysis procedures are provided in the accompanying trial protocol and statistical analysis plan.

Under the treatment policy estimand, the primary endpoint was analyzed using analysis of covariance (ANCOVA), with treatment group and previous use of glucose-lowering medication as fixed effects and baseline HbA1c as a covariate. Binary confirmatory secondary endpoints were analyzed using generalized estimating equations (GEE), whereas continuous confirmatory secondary endpoints were analyzed using ANCOVA with treatment group and previous use of glucose-lowering medication as fixed effects and the corresponding baseline value as a covariate.

Under the supportive efficacy estimand, binary endpoints were analyzed using GEE and continuous endpoints using mixed models for repeated measures (MMRM), with treatment group, visit, treatment-by-visit interaction and previous use of glucose-lowering medication as fixed effects and the corresponding baseline value as a covariate. An unstructured covariance matrix was used for within-participant errors in the MMRM; an alternative covariance structure was used if the model did not converge.

The family-wise type I error rate for the primary and confirmatory secondary endpoints was controlled with a fixed-sequence testing procedure at a one-sided significance level of 0.025. Superiority versus placebo was tested sequentially for safiglipron 90 mg, 60 mg and 30 mg for the primary endpoint, followed by the same dose order for HbA1c less than 7.0%, HbA1c of 6.5% or less, FPG and percentage change in body weight. Non-significant results rendered subsequent tests descriptive (Supplementary Table 2).

Other supportive and exploratory efficacy endpoints were analyzed in the intention-to-treat population using prespecified models appropriate to the endpoint type and measurement schedule. Skewed endpoints, including selected metabolic, lipid, hormonal and UACR variables, were analyzed after natural log transformation when appropriate and are presented as geometric mean percentage changes from baseline or percentage differences versus placebo.

Prespecified exploratory joint mediation analyses were conducted separately for each safiglipron dose versus placebo at week 32. The outcome was the natural logarithm of the week-32-to-baseline ratio in the C-peptide-based disposition index (ISSI-2); change from baseline in HbA1c (percentage points) and percentage change from baseline in body weight were entered simultaneously as mediators. Ordinary least squares models adjusted for log-transformed baseline ISSI-2, baseline HbA1c, age, sex, duration of type 2 diabetes and previous use of glucose-lowering medication. Complete-case analyses included 124, 119 and 120 participants for the 30 mg, 60 mg and 90 mg comparisons, respectively. Total effects were estimated from models excluding the mediators, indirect effects using the product-of-coefficients method and residual components from joint outcome models including both mediators. Bias-corrected 95% CIs were obtained from 10,000 non-parametric participant-level bootstrap resamples with replacement.

Safety analyses were done in the safety population, defined as all randomly assigned participants who received at least one dose of study treatment and analyzed according to randomized treatment assignment. Adverse events were coded using the Medical Dictionary for Regulatory Activities (version 28.0) and summarized by treatment group as TEAEs, treatment-related TEAEs, serious adverse events, adverse events of special interest and adverse events leading to treatment discontinuation. Hypoglycemic events, vital signs, physical examination findings, laboratory variables, 12-lead electrocardiograms and diabetic retinopathy assessments were summarized descriptively.

Analyses were done using SAS version 9.4 software (SAS Institute), except for the mediation analyses, which were performed using Python version 3.12 (Python Software Foundation). Details of the statistical analysis are provided in the Supplementary Methods.

### Reporting summary

Further information on research design is available in the Nature Portfolio Reporting Summary linked to this article.

## Online content

Any methods, additional references, Nature Portfolio reporting summaries, source data, extended data, supplementary information, acknowledgements, peer review information; details of author contributions and competing interests; and statements of data and code availability are available at https://doi.org/10.1038/s41591-026-04651-9.

## Supplementary information

Supplementary Information Supplementary Methods, Supplementary Figs. 1 and 2, Supplementary Tables 1−19, Supplementary References, protocol, statistical analysis plan and publication statistical analysis plan.

Reporting Summary

Supplementary Software 1 Python software package for the prespecified exploratory mediation analysis and plotting of Extended Data Fig. 6, including analysis and plotting scripts, the README, controlled input schema, package requirements and provenance/quality control documentation; no participant-level data are included.

## Source data

Source Data Fig. 2 Statistical source data for the six-panel glycemic and body weight outcomes figure.

Source Data Fig. 3 Statistical source data for indices of β cell function and insulin sensitivity.

Source Data Extended Data Fig. 2 Statistical source data for the subgroup analysis.

Source Data Extended Data Fig. 3 Observed HbA1c distribution and target attainment at week 32 under the supportive efficacy estimand.

Source Data Extended Data Fig. 4 Daily statistical source data for the prevalence and severity of selected gastrointestinal TEAEs.

Source Data Extended Data Fig. 5 Observed participant-level values and model estimates for cardiometabolic outcomes.

Source Data Extended Data Fig. 6 Statistical source data for the mediation analysis.

## Data Availability

Qualified researchers may request access to anonymized individual participant data that underlie the results reported in this article. Data will be made available beginning 24 months after primary publication of the trial results. Access will be provided to researchers who provide a methodologically sound proposal for a specific purpose. Proposals should be directed to the corresponding authors: X.X (xiaoxh2014@vip.163.com) or Z.Y. (zi.ye@hengrui.com). Requests will be evaluated by the corresponding authors and sponsor on the basis of scientific merit, methodological feasibility, consistency with participants’ informed consent and protection of participant confidentiality. Applicants will normally be informed of the decision within 8 weeks. Approved access will be subject to execution of a data use agreement, and data will be shared through a secure data transfer process. Source data are provided with this paper.

## Extended data

**Extended Data Table 1:** Treatment-emergent adverse events leading to study-drug discontinuation during the 32-week placebo-controlled period

	Safiglipron 30 mg (N=70)	Safiglipron 60 mg (N=70)	Safiglipron 90 mg (N=72)	Placebo (N=72)
Any TEAE leading to study treatment discontinuation	1 (1.4)	2 (2.9)	5 (6.9)	0
Nausea	0	2 (2.9)	2 (2.8)	0
Vomiting	0	1 (1.4)	2 (2.8)	0
Pancreatitis acute	1 (1.4)	0	0	0
Duodenitis	0	0	1 (1.4)	0
Asthenia	0	0	2 (2.8)	0
Decreased appetite	0	1 (1.4)	0	0
Tinnitus	0	0	1 (1.4)	0
Lipase increased	0	0	1 (1.4)	0
Dizziness	0	0	1 (1.4)	0

Data are n (%). The safety population included all randomly assigned participants who received at least one dose of study drug. Percentages were calculated using the number of participants in each treatment group as the denominator. TEAEs were adverse events that occurred or worsened after the first dose of study drug and during the 32-week placebo-controlled period. Events were coded using MedDRA version 28.0 and are listed by preferred term. A participant could have more than one TEAE leading to discontinuation of study treatment and could therefore be counted under more than one preferred term. MedDRA=Medical Dictionary for Regulatory Activities. TEAE=treatment-emergent adverse event.

**Extended Data Table 2:** Postbaseline liver-function laboratory abnormalities during the 32-week placebo-controlled period

Laboratory abnormality	Safiglipron 30 mg (N=70)	Safiglipron 60 mg (N=70)	Safiglipron 90 mg (N=72)	Placebo (N=72)
ALT >3× ULN	0	0	2 (2.8)	1 (1.4)
ALT >5× ULN	0	0	0	0
AST >3× ULN	0	0	0	0
AST >5× ULN	0	0	0	0
Total bilirubin >2× ULN	3 (4.3)	0	0	0
ALT or AST >3× ULN	0	0	2 (2.8)	1 (1.4)
ALT or AST >3× ULN with total bilirubin >2× ULN*	0	0	0	0

Data are n (%). The safety population included all randomly assigned participants who received at least one dose of study drug. Percentages were calculated using the number of participants in each treatment group as the denominator. Laboratory abnormalities were counted if they occurred after treatment initiation and during the 32-week placebo-controlled period; participants with more than one qualifying value in the same category were counted once. *Combination consistent with potential Hy’s law laboratory criteria, defined as concurrent ALT or AST > 3× ULN and total bilirubin >2× ULN in the absence of cholestasis or other alternative explanations. ALT=alanine aminotransferase. AST=aspartate aminotransferase. ULN=upper limit of normal.

## Code availability

The Python code used for the prespecified exploratory mediation analyses is provided as Supplementary Software 1.
