# Supplementary material for: Oral small-molecule GLP-1 receptor agonist safiglipron in early type 2 diabetes: a randomized, double-blind, placebo-controlled trial
Source: Nat Med. 2026 Sep 29;32(11):4330–9. doi: 10.1038/s41591-026-04651-9 (PMC13768188; doi:10.1038/s41591-026-04651-9)
Supplement: Supplementary file 1 [file 41591_2026_4651_MOESM1_ESM.pdf]

nature medicine

Supplementary information https://doi.org/10.1038/s41591-026-04651-9

Oral small-molecule GLP-1 receptor agonist safiglipron in early type 2 diabetes: a randomized, double-blind, placebo-controlled trial

In the format provided by the authors and unedited

Contents

Supplementary Methods——Detailed statistical analysis

Supplementary Figure

- Supplementary Fig. 1 | Seven-point self-monitored blood glucose profiles at baseline and week 52
- Supplementary Fig. 2 | HbA1c target attainment at week 32 under two estimands

Supplementary Table

- Supplementary Table 1 | Trial investigators and sites
- Supplementary Table 2 | Prespecified hypotheses and endpoints
- Supplementary Table 3 | Sensitivity analyses for the primary endpoint
- Supplementary Table 4 | Other efficacy endpoints at week 32 under the supportive efficacy estimand
- Supplementary Table 5 | Systolic blood pressure and urinary albumin-to-creatinine ratio at week 32 under the supportive efficacy estimand
- Supplementary Table 6 | Efficacy outcomes through week 52 (treatment-policy estimand)
- Supplementary Table 7 | Lipid parameters through week 52
- Supplementary Table 8 | Urinary albumin-to-creatinine ratio through week 52, observed-case analysis
- Supplementary Table 9 | Change from baseline in DTSQs scores during the 52-week treatment period
- Supplementary Table 10 | Serious treatment-emergent adverse events during the 32-week placebo-controlled period
- Supplementary Table 11 | Gastrointestinal-related treatment-emergent adverse events by maximum severity during the 32-week placebo-controlled period
- Supplementary Table 12 | Change from baseline in the worst diabetic retinopathy stage during the 32-week placebo-controlled period
- Supplementary Table 13 | Treatment-emergent adverse events during the 52-week treatment period
- Supplementary Table 14 | Serious treatment-emergent adverse events during the 52-week treatment period
- Supplementary Table 15 | Treatment-emergent adverse events leading to study treatment discontinuation during the 52-week treatment period
- Supplementary Table 16 | Postbaseline liver-function laboratory abnormalities during the 52-week treatment period
- Supplementary Table 17 | Selected hepatic, pancreatic and thyroid-related laboratory parameters through week 52
- Supplementary Table 18 | Vital signs through week 52
- Supplementary Table 19 | Electrocardiographic parameters through week 52

Supplementary References

Trial Protocol

Statistical Analysis Plan

Publication Statistical Analysis Plan

Supplementary Methods

Detailed statistical analysis

1. Estimands

In accordance with the ICH E9(R1) addendum, two estimands were defined to address distinct clinical questions regarding the efficacy of safiglipron. The primary estimand (treatment-policy estimand) was constructed using a treatment-policy strategy for all intercurrent events, reflecting the treatment effect under real-world conditions in which all post-event data were retained.
A supportive efficacy estimand was used to assess the specific pharmacological effect of safiglipron under conditions where intercurrent events had not occurred, applying a hypothetical strategy to remove the confounding influence of treatment discontinuation and rescue medication. Two supportive estimands were pre-specified within the efficacy estimand framework, differing only in their handling of ICE3 (insufficient drug exposure): supportive estimand 1 applied the hypothetical strategy to all three ICEs, whereas supportive estimand 2 retained the treatment-policy strategy for ICE3 while applying the hypothetical strategy to ICE1 and ICE2.

Three intercurrent events (ICEs) were defined:

- **ICE1**: premature discontinuation of study treatment, defined as any subject whose reason for treatment discontinuation is not "completed"
- **ICE2**: initiation of additional hypoglycaemic drugs used for more than 14 cumulative days from randomisation
- **ICE3**: insufficient drug exposure before a scheduled visit, defined as actual cumulative drug exposure within 90 days prior to the visit being less than 50% of the target total exposure, or the number of PK values below the lower limit of quantification (BLQ) within that 90-day window exceeding 50% of the planned PK assessments (applicable from the Week 20 visit during the core period)

Primary estimand (treatment-policy estimand) applied a treatment-policy strategy to all three ICEs; data after ICE occurrence were included. Missing data attributable to ICEs were imputed using the retrieved dropout (RDO) method; if RDO was not supported, the copy-reference (CR) method was used; remaining missing data unrelated to ICEs were assumed missing at random (MAR).

Supportive efficacy estimand 1 applied a hypothetical strategy to all three ICEs; data after any ICE were considered missing and excluded. All remaining missing data were assumed MAR.

Supportive estimand 2 applied a hypothetical strategy to ICE1 and ICE2 and a treatment-policy strategy to ICE3; all other attributes were the same as supportive estimand 1.

For key secondary efficacy endpoints, the treatment-policy estimand was the primary estimand for confirmatory analyses; the supportive efficacy estimand was used for supportive analyses.

2. Analysis Populations

- **Intention-to-treat (ITT) set:** All randomised participants, analysed according to randomised treatment assignment. Used for all efficacy analyses.
- **Safety set (SS):** All participants who received at least one dose of the investigational product after randomisation, analysed according to randomised treatment assignment.
- **PK concentration analysis set (PKCS):** All participants randomised to receive safiglipron with at least one evaluable post-dose PK concentration measurement.

3. Statistical Analyses

3.1 Primary analysis

For the primary estimand, the ITT set was analysed using an ANCOVA model for HbA1c change from baseline at Week 32. Fixed effects were treatment group and prior use of hypoglycaemic drugs (yes, no); baseline HbA1c was the covariate. Missing data were handled as described in Section 1 (RDO/CR for ICE-related; MAR for others); multiply-imputed datasets were combined using Rubin's rules. LS means, standard errors, 95% CIs, and one-sided p-values for each safiglipron dose group versus placebo were reported.

3.2 Supportive analysis

For supportive estimand 1, the primary endpoint was analysed in the ITT set using MMRM (REML). Fixed effects were prior use of hypoglycaemic drugs (yes, no), treatment, visit, and treatment-by-visit interaction; baseline HbA1c was the covariate. Within-subject variability was modelled using an unstructured (UN) or compound symmetry (CS) covariance structure. Data after any ICE were excluded; remaining missing data were assumed MAR. Supportive estimand 2 was analysed using the same MMRM approach.

3.3 Sensitivity analyses

Sensitivity analysis 1: LOCF

LOCF imputation for all missing HbA1c measurements from Week 0 to Week 32, followed by ANCOVA with the same model as the primary analysis.

Sensitivity analysis 2: Tipping point analysis

delta adjustments were applied to the imputed values of each safiglipron and placebo group to progressively reduce the between-group difference; the threshold delta at which statistical significance was lost was identified. Under each delta, MAR-based multiple imputation was applied followed by ANCOVA consistent with the primary analysis; results were combined using Rubin's rules.[1]

3

3.4 Key secondary and exploratory analyses

Binary key secondary endpoints (proportion achieving HbA1c <7.0% or ≤6.5% at Week 32):

- Treatment-policy estimand: missing HbA1c values were first imputed per primary analysis rules; the binary outcome was then analysed using GEE with a logit link, including prior use of hypoglycaemic drugs (yes, no), treatment, visit, and treatment-by-visit interaction as fixed effects, baseline HbA1c as a covariate, with participant specified as the clustering variable and an unstructured working correlation structure (compound symmetry if required for model convergence). Odds ratios, 95% CIs, and one-sided P values were reported. Results across imputed datasets were combined using Rubin's rules.
- Supportive efficacy estimand: the same GEE model was applied without prior imputation after post-intercurrent-event data were set to missing; available observations contributed to the GEE analysis.

Continuous key secondary endpoints (change in FPG and percentage change in body weight at Week 32):

- Treatment-policy estimand: ANCOVA with the same model and imputation rules as the primary analysis.
- Supportive efficacy estimand: MMRM (REML) with two stratification factors — prior use of hypoglycaemic drugs (yes, no) and baseline HbA1c (≤8.5%, >8.5%) — treatment, visit, and treatment-by-visit interaction as fixed effects, the corresponding baseline as a covariate, and UN or CS covariance. Missing data handled by MMRM under MAR.

Exploratory endpoints:

Changes from baseline in systolic and diastolic blood pressure were analysed using MMRM (REML) with both stratification factors (prior use of hypoglycaemic drugs [yes, no] and baseline HbA1c [≤8.5%, >8.5%]), treatment, visit, and treatment-by-visit interaction as fixed effects, and the corresponding baseline as a covariate. For TG, TC, LDL-C, HDL-C, and UACR, the same MMRM was applied after natural log transformation; LS means and differences were back-transformed by exponentiation to yield percentage changes in geometric means and between-group geometric mean ratios. Week-52 HbA1c and selected glycaemic and anthropometric endpoints were analysed using prespecified ANCOVA or MMRM models, as applicable; remaining week-52 supportive and exploratory endpoints were summarised descriptively using observed-case data.

Prespecified exploratory mediation analyses were conducted separately for each safiglipron dose versus placebo at week 32. The outcome was the natural logarithm of the week-32-to-baseline ratio in the C-peptide-based disposition index (ISSI-2). A joint mediation model included change from baseline in HbA1c and percentage change from baseline in bodyweight as simultaneous

mediators and adjusted for log-transformed baseline ISSI-2, baseline HbA1c, age, sex, duration of type 2 diabetes, and previous use of glucose-lowering medication. Analyses included participants with complete data for the outcome, both mediators, and all covariates (n=124, 119, and 120 for the 30 mg, 60 mg, and 90 mg comparisons, respectively). Indirect effects were estimated using the product-of-coefficients method. The total effect, the two indirect effects, and the residual component not statistically explained by the measured mediators were estimated on the log-ratio scale; 95% CIs were obtained from 10,000 nonparametric bias-corrected bootstrap resamples.

4. Statistical Hypothesis Testing

Each safiglipron dose group was compared with placebo on the primary and key secondary endpoints at a one-sided significance level of 0.025. Hypothesis notation: first subscript 1, 2, 3 = safiglipron 90 mg, 60 mg, 30 mg; second subscript 1–5 = endpoint.

Supplementary Table 2 | Prespecified hypotheses and endpoints

Hypothesis	Endpoint
H_{11}, H_{21}, H_{31}	HbA1c change from baseline at Week 32
H_{12}, H_{22}, H_{32}	Proportion with HbA1c <7.0% at Week 32
H_{13}, H_{23}, H_{33}	Proportion with HbA1c ≤6.5% at Week 32
H_{14}, H_{24}, H_{34}	FPG change from baseline at Week 32
H_{15}, H_{25}, H_{35}	Percentage body weight change from baseline at Week 32

Fixed-sequence testing order: $H_{11} \rightarrow H_{21} \rightarrow H_{31} \rightarrow H_{12} \rightarrow H_{22} \rightarrow H_{32} \rightarrow H_{13} \rightarrow H_{23} \rightarrow H_{33} \rightarrow H_{14} \rightarrow H_{24} \rightarrow H_{34} \rightarrow H_{15} \rightarrow H_{25} \rightarrow H_{35}$. Testing stops at the first hypothesis with a one-sided p-value ≥ 0.025.

5. Sample Size and Power

The sample size was based on the primary endpoint. Assuming a treatment difference of −0.85% for participants without ICEs and 0% for participants with ICEs (anticipated ICE rate: 15%), the expected overall difference was −0.72%, with a standard deviation of 1.1%. Randomising 68 participants per group (272 total, 1:1:1:1 ratio) provides 90% overall power to demonstrate superiority of all three safiglipron dose groups versus placebo at a one-sided significance level of 0.025.

5

6. Control of Multiplicity

A fixed-sequence testing procedure controlled the family-wise type I error rate at a one-sided 0.025 level across the 15 pre-specified hypotheses (H11 through H35). All statistical analyses were performed using SAS® 9.4 or later.

6

Supplementary Fig. 1 | Seven-point self-monitored blood glucose profiles at baseline and week 52

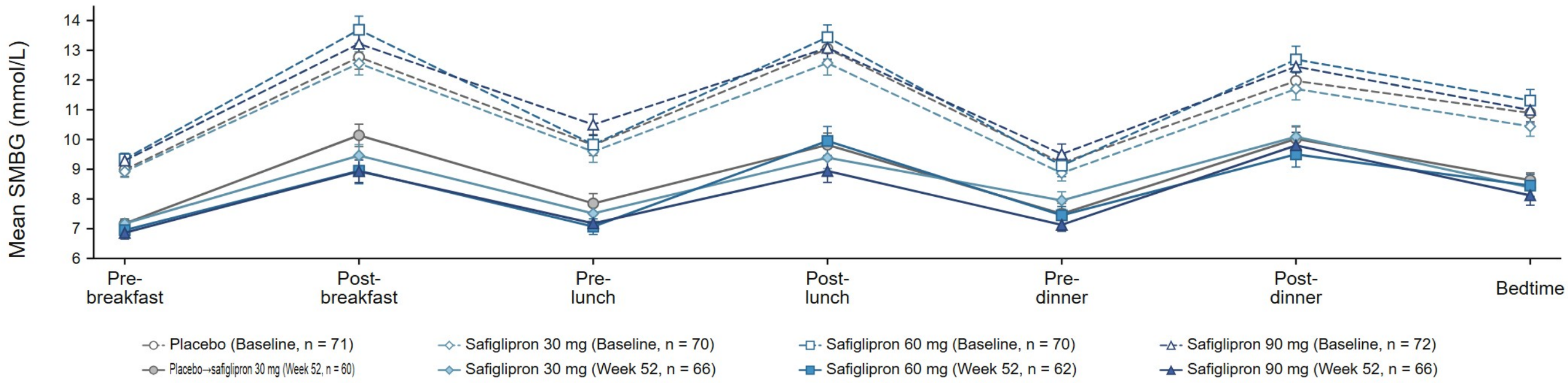

Points and lines show observed mean seven-point self-monitored blood glucose profiles; error bars indicate SE. Baseline profiles are shown by dashed lines and open symbols, and week-52 profiles by solid lines and filled symbols. Gray circles, light-blue diamonds, medium-blue squares and dark-blue triangles denote the placebo-randomized, safiglipron 30 mg, safiglipron 60 mg and safiglipron 90 mg groups, respectively. Exact available n values are shown in the figure key; n is the number of individual randomized participants contributing observed data at the corresponding visit, and the same n applies to all seven time points within each visit profile. Participants randomized to placebo received placebo through week 32 and switched to safiglipron 30 mg at week 33 as prespecified; therefore, week-52 values are displayed by randomized group and do not constitute a concurrent placebo-controlled comparison. Data are from observed cases in the intention-to-treat population. No formal between-group comparisons were performed.

SE=standard error. SMBG=self-monitored blood glucose.

Supplementary Fig. 2 | HbA1c target attainment at week 32 under two estimands

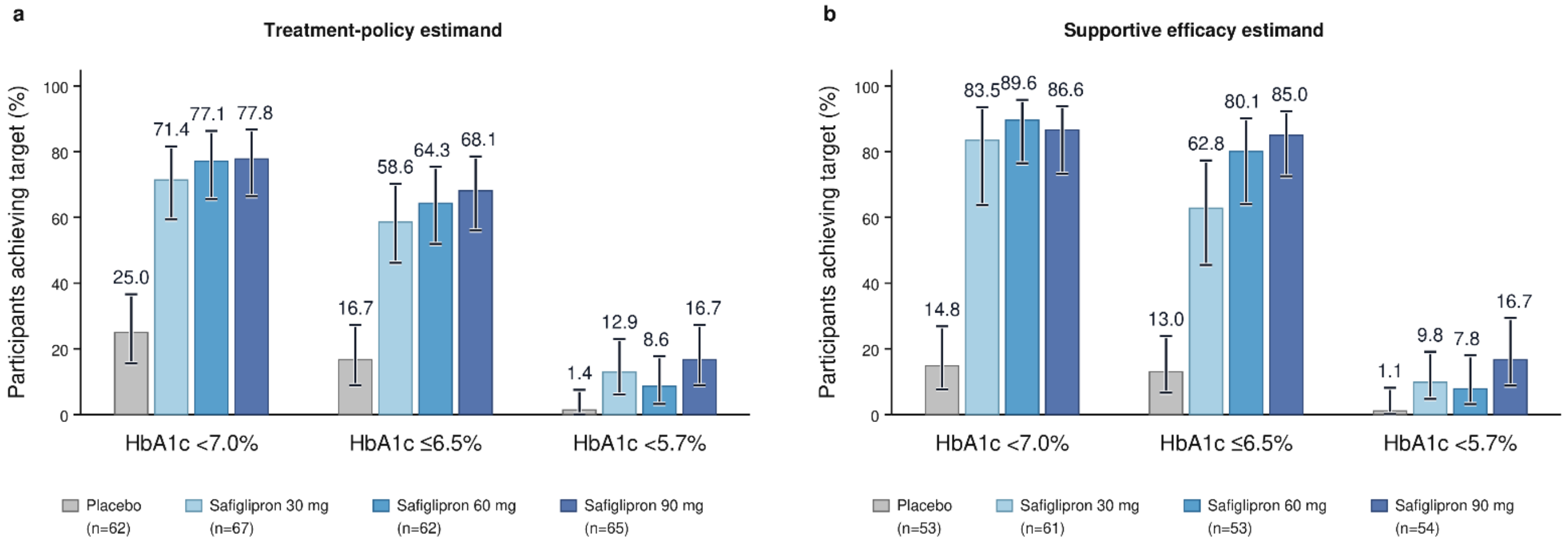

a, Treatment-policy estimand. Bars show proportions estimated after prespecified retrieved-dropout imputation, values above the bars show the corresponding percentages, and error bars indicate two-sided Clopper–Pearson exact 95% CIs. The intention-to-treat population comprised N=72, 70, 70 and 72 randomized participants for placebo and safiglipron 30 mg, 60 mg and 90 mg, respectively. Observed week-32 HbA1c data were available for n=62, 67, 62 and 65 participants, respectively, as shown with the group labels. b, Supportive efficacy estimand (hypothetical strategy), with post-intercurrent-event data set to missing. Bars show GEE model-estimated marginal proportions, values above the bars show the corresponding percentages, and error bars indicate two-sided 95% CIs. The corresponding n values denote participants with week-32 HbA1c data contributing to the GEE analysis: n=53, 61, 53 and 54 for placebo and safiglipron 30 mg, 60 mg and 90 mg, respectively. Dose-versus-placebo comparisons used GEE with a logit link, including previous glucose-lowering medication use, treatment, visit and the treatment-by-visit interaction as fixed effects and baseline HbA1c as a covariate, with participant as the repeated unit. The individual randomized participant was the independent unit of study; no technical replicates were used. All P values were from one-sided superiority tests. In a, comparisons for the <7.0% and ≤6.5% targets formed part of the prespecified fixed-sequence procedure controlling the family-wise type I error rate at α=0.025; comparisons for the <5.7% target were nominal and unadjusted for multiplicity. In b, all comparisons were nominal and unadjusted for multiplicity. For the <7.0% and ≤6.5% targets, all dose-versus-placebo comparisons had P<0.0001 in both panels. For the <5.7% target, P values for 30 mg, 60 mg and 90 mg versus placebo were 0.0217, 0.0481 and 0.0070 in a, and 0.0203, 0.0384 and 0.0045 in b, respectively. CI=confidence interval. GEE=generalized estimating equation. HbA1c=glycated hemoglobin.

8

Supplementary Table 1 | Trial investigators and sites

A total of 46 sites participated in the study, and each site screened and obtained informed consent from at least one participant.

Site ID	Institution	Principal Investigator
CN001	Peking Union Medical College Hospital, Chinese Academy of Medical Sciences	Xinhua Xiao
CN002	Beijing Aerospace General Hospital	Wei Zhu
CN003	Gansu Provincial People's Hospital	Jinyang Wang
CN004	Second Hospital of Hebei Medical University	Lihui Zhang
CN005	First Affiliated Hospital of Henan University of Science and Technology	Yujin Ma
CN006	Third People's Hospital of Luoyang	Junhang Tian
CN007	Zhumadian Central Hospital	Lianwei Wang
CN008	Heilongjiang Provincial Hospital	Binhong Duan
CN009	Fifth People's Hospital of Shenyang	Yuzhu Zhao
CN010	Baotou Central Hospital	Yan Zhu
CN011	First Affiliated Hospital of Baotou Medical College, Inner Mongolia University of Science and Technology	Feng Wei
CN012	The First Hospital of Shanxi Medical University	Yan Wang
CN013	Shaanxi Provincial People's Hospital	Xiaofeng Li
CN014	Jinshan Hospital, Fudan University	Xiaohong Shi
CN015	Qingpu Branch of Zhongshan Hospital, Fudan University	Min Zhang
CN016	The 2nd School of Medicine, WMU / The 2nd Affiliated Hospital and Yuying Children's Hospital of WMU	Zhaoming Wu
CN017	Zhongshan Hospital Xiamen University	Xiaomin Chen
CN018	Huizhou Central People's Hospital	Shu Li
CN019	Liuzhou People's Hospital	Na Liu
CN020	The First Affiliated Hospital of Hainan Medical University	Liling Tan
CN022	Cangzhou Hospital of Integrated TCM-WM, Hebei	Xiuhai Su

9

Site ID	Institution	Principal Investigator
CN023	The First Affiliated Hospital of University of Science and Technology of China (Anhui Provincial Hospital)	Wei Wang
CN025	The First Affiliated Hospital of Soochow University	Bimin Shi
CN026	The Affiliated Hospital of Xuzhou Medical University	Hongwei Ling
CN027	The First People's Hospital of Zunyi	Chengyan Jiang
CN028	Xiangyang Central Hospital	Shaoyong Xu
CN029	Yichang Central People's Hospital	Zhaoyang Zeng
CN030	The Third Hospital of Changsha	Debin Huang
CN031	The First People's Hospital of Chenzhou	Weihong Song
CN032	Yueyang Central Hospital	Xianghua Zhang
CN033	Pingxiang People's Hospital	Yawei Zhang
CN034	Affiliated Hospital of Southwest Medical University	Qin Wan
CN035	First Affiliated Hospital of Chongqing Medical University	Qifu Li
CN036	First Affiliated Hospital of Kunming Medical University	Huifang Li
CN037	General Hospital of Ningxia Medical University	Xiaoying Dong
CN038	Fuzhou University Affiliated Provincial Hospital	Huibin Huang
CN039	Longhua District People's Hospital of Shenzhen	Zhiming Liu
CN040	Baoding No.1 Central Hospital	Yunliang Zhang
CN041	Huzhou Central Hospital	Jianping Yao
CN042	Weifang People's Hospital	Haixia Liu
CN043	Lianyungang Hospital of Traditional Chinese Medicine	Dongji Wang
CN044	Affiliated Hospital of Nantong University	Yunjuan Gu
CN045	Jilin Provincial People's Hospital	Kexin Li
CN046	The Second Affiliated Hospital of Hainan Medical University	Zongcun Chen
CN047	Fourth Affiliated Hospital of Harbin Medical University	Shuang Yan
CN048	Xuancheng People's Hospital	Sihong Wang

10

Site ID	Institution	Principal Investigator

11

Supplementary Table 3 | Sensitivity analyses for the primary endpoint

Analysis Method	Safiglipron 30 mg (N=70)	Safiglipron 60 mg (N=70)	Safiglipron 90 mg (N=72)	Placebo (N=72)
**Primary Analysis***	−1.40 (−1.61 to −1.18) n=67	−1.38 (−1.60 to −1.16) n=62	−1.63 (−1.84 to −1.41) n=65	−0.18 (−0.40 to 0.05) n=62
Difference vs placebo, % (95% CI); p value	−1.22 (−1.53 to −0.91); p<0.0001	−1.20 (−1.52 to −0.89); p<0.0001	−1.45 (−1.75 to −1.14); p<0.0001	NA
**Supplementary Analysis**				
**†Supportive efficacy estimand 1 (MMRM)**	−1.40 (−1.61 to −1.19) n=61	−1.48 (−1.70 to −1.26) n=53	−1.68 (−1.90 to −1.47) n=54	−0.06 (−0.28 to 0.16) n=53
Difference vs placebo, % (95% CI); p value	−1.34 (−1.65 to −1.04); p<0.0001	−1.42 (−1.73 to −1.11); p<0.0001	−1.62 (−1.93 to −1.32); p<0.0001	NA
**‡Supportive efficacy estimand 2 (MMRM)**	−1.39 (−1.61 to −1.17) n=64	−1.46 (−1.69 to −1.23) n=58	−1.66 (−1.88 to −1.43) n=58	−0.05 (−0.28 to 0.18) n=53
Difference vs placebo, % (95% CI); p value	−1.34 (−1.66 to −1.03); p<0.0001	−1.41 (−1.73 to −1.09); p<0.0001	−1.60 (−1.92 to −1.29); p<0.0001	NA
**Sensitivity Analyses**				
**§1. LOCF imputation; ANCOVA**	−1.39 (−1.61 to −1.18) n=67	−1.37 (−1.58 to −1.16) n=62	−1.56 (−1.77 to −1.35) n=65	−0.18 (−0.39 to 0.03) n=62
Difference vs placebo, % (95% CI); p value	−1.22 (−1.51 to −0.93);	−1.19 (−1.48 to −0.90);	−1.38 (−1.67 to −1.10);	NA

12

	p<0.0001	p<0.0001	p<0.0001	
**¶2. Tipping point analysis**	See footnote			

Data are least-squares mean change from baseline in HbA1c at week 32 (95% CI). Second line of each row shows placebo-corrected difference (95% CI). All p values are nominal and not adjusted for multiplicity. Analyses are based on the intention-to-treat population. N denotes the number of participants in the intention-to-treat population; n denotes participants with an observed week-32 HbA1c value. For MMRM analyses, n is the week-32 available-observation count and is not the total number of participants contributing longitudinal data to the model.

* Treatment-policy estimand; ANCOVA with treatment group and prior use of hypoglycaemic drugs as fixed effects, baseline HbA1c as covariate; missing data imputed using RDO or CR method; remaining missing data assumed MAR; Rubin's rules.

† Supportive efficacy estimand 1: hypothetical strategy for all three ICEs; data after any ICE excluded; all remaining missing data assumed MAR; MMRM (REML) with prior use of hypoglycaemic drugs, treatment, visit, and treatment×visit as fixed effects, baseline HbA1c as covariate; UN or CS covariance.

‡ Supportive efficacy estimand 2 (hybrid intercurrent-event strategy): hypothetical strategy for ICE1 and ICE2; treatment-policy strategy for ICE3; same MMRM specification as supportive efficacy estimand 1.

§ All missing HbA1c values imputed using last observation carried forward; same ANCOVA model as primary analysis.

¶ Results remained statistically significant (one-sided p<0.025) under all delta combinations tested: for safiglipron 30 mg, significance was maintained until penalty exceeded −1.2% applied to the experimental group simultaneously with +1.0% applied to the placebo group; for 60 mg, until −0.9% and +0.8% respectively; for 90 mg, until −1.2% and +1.0% respectively.

13

Supplementary Table 4 | Other efficacy endpoints at week 32 under the supportive efficacy estimand

Endpoint*	Safiglipron 30 mg (N=70)	Safiglipron 60 mg (N=70)	Safiglipron 90 mg (N=72)	Placebo (N=72)
**Participants achieving HbA1c <7.0%**	n=61	n=53	n=54	n=53
Model-estimated responder rate, % (95% CI)	83.5 (63.74 to 93.59)	89.6 (76.47 to 95.79)	86.6 (73.28 to 93.84)	14.8 (7.64 to 26.85)
Odds ratio vs placebo (95% CI); p value	29.09 (7.76 to 109.07)	49.38 (14.08 to 173.11)	37.10 (11.65 to 118.17)	NA
	p<0.0001	p<0.0001	p<0.0001	
**Participants achieving HbA1c ≤6.5%**	n=61	n=53	n=54	n=53
Model-estimated responder rate, % (95% CI)	62.8 (45.51 to 77.34)	80.1 (64.00 to 90.14)	85.0 (72.51 to 92.37)	13.0 (6.61 to 23.90)
Odds ratio vs placebo (95% CI); p value	11.32 (4.17 to 30.73)	27.04 (8.85 to 82.60)	37.89 (12.84 to 111.85)	NA
	p<0.0001	p<0.0001	p<0.0001	
**Fasting plasma glucose, mmol/L**	n=61	n=53	n=54	n=53
LS mean change from baseline (95% CI)	−1.99 (−2.39 to −1.60)	−2.23 (−2.65 to −1.80)	−2.59 (−3.01 to −2.17)	−0.23 (−0.64 to 0.19)
Difference vs placebo (95% CI); p value	−1.77 (−2.31 to −1.22);	−2.00 (−2.56 to −1.44);	−2.36 (−2.92 to −1.81);	NA
	p<0.0001	p<0.0001	p<0.0001	
**Bodyweight, kg**	n=61	n=53	n=54	n=53
LS mean change from baseline (95% CI)	−1.80 (−2.62 to −0.97)	−3.29 (−4.15 to −2.43)	−3.99 (−4.83 to −3.15)	−1.41 (−2.26 to −0.56)

14

Difference vs placebo (95% CI); p value	−0.39 (−1.56 to 0.78); p=0.2561	−1.88 (−3.08 to −0.68); p=0.0011	−2.58 (−3.76 to −1.40); p<0.0001	NA
**Waist circumference, cm**	n=61	n=53	n=54	n=53
LS mean change from baseline (95% CI)	−1.75 (−2.69 to −0.80)	−3.28 (−4.27 to −2.29)	−3.93 (−4.90 to −2.96)	−1.92 (−2.91 to −0.94)
Difference vs placebo (95% CI); p value	0.18 (−1.17 to 1.52); p=0.3976	−1.35 (−2.73 to 0.02); p=0.0270	−2.00 (−3.36 to −0.64); p=0.0020	NA
**Fasting serum insulin, μIU/mL†**	n=61	n=54	n=54	n=53
Geometric mean ratio to baseline (95% CI)	1.08 (0.96 to 1.21)	0.95 (0.84 to 1.07)	0.96 (0.85 to 1.09)	0.91 (0.80 to 1.03)
Ratio vs placebo (95% CI); p value	1.18 (1.01 to 1.39); p=0.0212	1.04 (0.88 to 1.24); p=0.3070	1.05 (0.89 to 1.25); p=0.2671	NA
**Fasting serum C-peptide, ng/mL†**	n=61	n=54	n=54	n=54
Geometric mean ratio to baseline (95% CI)	1.09 (1.00 to 1.19)	1.05 (0.96 to 1.15)	1.04 (0.95 to 1.15)	0.98 (0.90 to 1.08)
Ratio vs placebo (95% CI); p value	1.11 (0.99 to 1.25); p=0.0388	1.07 (0.95 to 1.21); p=0.1304	1.06 (0.94 to 1.20); p=0.1618	NA
**HOMA-β, %†**	n=61	n=54	n=54	n=53
Geometric mean ratio to baseline (95% CI)	1.76 (1.53 to 2.03)	1.74 (1.50 to 2.01)	2.02 (1.74 to 2.35)	1.02 (0.88 to 1.18)
Ratio vs placebo (95% CI); p value	1.73 (1.43 to 2.09); p<0.0001	1.71 (1.41 to 2.08); p<0.0001	1.99 (1.63 to 2.41); p<0.0001	NA
**HOMA-IR†**	n=61	n=54	n=54	n=53

15

Geometric mean ratio to baseline (95% CI)	0.81 (0.71 to 0.93)	0.69 (0.59 to 0.79)	0.63 (0.55 to 0.73)	0.85 (0.73 to 0.98)
Ratio vs placebo (95% CI); p value	0.96 (0.79 to 1.16);	0.81 (0.67 to 0.98);	0.75 (0.61 to 0.91);	NA
	p=0.3340	p=0.0174	p=0.0018	
**1-h postprandial plasma glucose, mmol/L‡**	n=61	n=54	n=53	n=54
LS mean change from baseline (95% CI)	−3.03 (−3.91 to −2.16)	−3.77 (−4.70 to −2.83)	−4.90 (−5.84 to −3.96)	0.13 (−0.74 to 1.00)
Difference vs placebo (95% CI); p value	−3.17 (−4.33 to −2.01);	−3.90 (−5.07 to −2.73);	−5.04 (−6.19 to −3.89);	NA
	p<0.0001	p<0.0001	p<0.0001	
**2-h postprandial plasma glucose, mmol/L‡**	n=61	n=54	n=53	n=54
LS mean change from baseline (95% CI)	−4.26 (−5.26 to −3.26)	−6.03 (−7.10 to −4.97)	−6.50 (−7.60 to −5.40)	0.24 (−0.82 to 1.30)
Difference vs placebo (95% CI); p value	−4.50 (−5.81 to −3.19);	−6.27 (−7.60 to −4.94);	−6.74 (−8.12 to −5.36);	NA
	p<0.0001	p<0.0001	p<0.0001	
**2-h postprandial serum insulin, μIU/mL†‡**	n=60	n=54	n=53	n=53
Geometric mean ratio to baseline (95% CI)	1.38 (1.09 to 1.76)	1.21 (0.94 to 1.55)	1.07 (0.81 to 1.42)	0.96 (0.75 to 1.23)
Ratio vs placebo (95% CI); p value	1.45 (1.06 to 1.98);	1.26 (0.89 to 1.78);	1.12 (0.79 to 1.59);	NA
	p=0.0105	p=0.0942	p=0.2562	
**2-h postprandial serum C-peptide, ng/mL†‡**	n=61	n=54	n=54	n=54
Geometric mean ratio to baseline (95% CI)	1.29 (1.13 to 1.47)	1.22 (1.07 to 1.40)	1.20 (1.04 to 1.38)	1.03 (0.90 to 1.17)
Ratio vs placebo (95% CI);	1.26 (1.06 to	1.19 (1.00 to	1.17 (0.98 to	NA

16

p value	1.50); p=0.0047	1.42); p=0.0239	1.39); p=0.0457	
**Disposition index†‡**	n=60	n=53	n=52	n=50
Geometric mean ratio to baseline (95% CI)	1.72 (1.51 to 1.97)	2.23 (1.95 to 2.55)	2.67 (2.33 to 3.06)	1.17 (1.02 to 1.34)
Ratio vs placebo (95% CI); p value	1.47 (1.25 to 1.74); p<0.0001	1.91 (1.60 to 2.27); p<0.0001	2.28 (1.91 to 2.73); p<0.0001	NA
**Participants achieving HbA1c <5.7%§**	n=61	n=53	n=54	n=53
Model-estimated responder rate, % (95% CI)	9.8 (4.75 to 19.11)	7.8 (3.17 to 17.99)	16.7 (8.78 to 29.36)	1.1 (0.14 to 8.13)
Odds ratio vs placebo (95% CI); p value	9.91 (1.10 to 88.91); p=0.0203	7.74 (0.80 to 74.68); p=0.0384	18.26 (2.06 to 161.90); p=0.0045	NA
**Participants receiving rescue therapy during weeks 0–32§**	0	0	0	7 (9.7)

*Analyses are based on the efficacy estimand. Data are least-squares mean change from baseline (95% CI), difference versus placebo (95% CI), geometric mean ratio to baseline (95% CI), ratio versus placebo (95% CI), or n (%; 95% CI), unless otherwise specified. The efficacy estimand was used to estimate treatment effects assuming that participants remained on study treatment without intercurrent events. N denotes the number of participants in the intention-to-treat population; n denotes participants with an observed week-32 value contributing to the analysis of the specified endpoint.

†Endpoints analysed after natural-log transformation are shown as geometric mean ratios to baseline; comparisons versus placebo are shown as ratios of geometric mean ratios.

‡Postprandial outcomes were derived from the mixed-meal tolerance test.

Continuous endpoints were analysed using MMRM or ANCOVA as prespecified.

§HbA1c target achievement was analysed using GEE; model-estimated responder rates, 95% CIs, odds ratios versus placebo, and nominal p values are reported. Rescue therapy is reported descriptively as n (%) without formal between-group comparison.

P values are for comparisons with placebo and are nominal, without adjustment for multiplicity.

17

ANCOVA=analysis of covariance. GEE=generalised estimating equation. HbA1c=glycated haemoglobin. HOMA-β=homeostatic model assessment of β-cell function. HOMA-IR=homeostatic model assessment of insulin resistance. LS=least-squares. MMRM=mixed model for repeated measures. NA=not applicable.

18

Supplementary Table 5 | Systolic blood pressure and urinary albumin-to-creatinine ratio at week 32 under the supportive efficacy estimand

Endpoint	Safiglipron 30 mg (N=70)	Safiglipron 60 mg (N=70)	Safiglipron 90 mg (N=72)	Placebo (N=72)
**Systolic blood pressure, mm Hg**	n=61	n=53	n=54	n=53
LS mean change from baseline (95% CI)	−6.48 (−9.32 to −3.64)	−6.36 (−9.35 to −3.36)	−4.96 (−7.92 to −2.00)	−2.11 (−5.11 to 0.89)
Difference vs placebo (95% CI); p value	−4.37 (−8.35 to −0.38); p=0.0159	−4.24 (−8.34 to −0.14);p=0.0214	−2.85 (−6.91 to 1.22); p=0.0844	NA
**Urinary albumin-to-creatinine ratio, mg/g**	n=61	n=53	n=54	n=54
Geometric mean percentage change from baseline (95% CI), %	−43.21 (−56.20 to −26.35)	−30.68 (−47.35 to −8.71)	−40.78 (−54.96 to −22.13)	9.92 (−16.37 to 44.47)
Percentage difference vs placebo (95% CI); p value	−48.33 (−63.97 to −25.91); p=0.0002	−36.93 (−56.53 to −8.49); p=0.0077	−46.12 (−62.76 to −22.06); p=0.0006	NA

Data are from the intention-to-treat population under the supportive efficacy estimand and were estimated using MMRM. N denotes the number of participants in the intention-to-treat population; n denotes participants with an observed week-32 value for the specified endpoint.

Systolic blood pressure is shown as least-squares mean change from baseline and least-squares mean difference versus placebo.

Urinary albumin-to-creatinine ratio was analysed after natural-log transformation and is shown as geometric mean percentage change from baseline; percentage differences versus placebo were derived from the log-transformed model.

P values are nominal and were not adjusted for multiplicity.

LS=least-squares. MMRM=mixed model for repeated measures. NA=not applicable.

UACR=urinary albumin-to-creatinine ratio.

19

Supplementary Table 6 | Efficacy outcomes through week 52 (treatment-policy estimand)

Endpoint	Safiglipron 30 mg (N=70)	Safiglipron 60 mg (N=70)	Safiglipron 90 mg (N=72)	Placebo–safiglipron 30 mg (N=72)*
**Endpoints analysed using ANCOVA with retrieved-dropout data†**				
**HbA1c, %**	n=67	n=64	n=66	n=63
LS mean change from baseline (95% CI)	−1.30 (−1.50 to −1.09)	−1.24 (−1.44 to −1.04)	−1.52 (−1.72 to −1.32)	−1.01 (−1.22 to −0.80)
Difference vs placebo–safiglipron 30 mg (95% CI); p value	−0.29 (−0.57 to −0.00); p=0.0246	−0.23 (−0.52 to 0.06); p=0.0613	−0.51 (−0.79 to −0.23); p=0.0002	NA
**Fasting plasma glucose, mmol/L**	n=67	n=64	n=66	n=63
LS mean change from baseline (95% CI)	−1.64 (−2.02 to −1.26)	−1.94 (−2.33 to −1.55)	−2.31 (−2.71 to −1.91)	−1.68 (−2.06 to −1.30)
Difference vs placebo–safiglipron 30 mg (95% CI); p value	0.04 (−0.46 to 0.54); p=0.4378	−0.26 (−0.76 to 0.24); p=0.1556	−0.63 (−1.13 to −0.13); p=0.0065	NA
**Bodyweight, kg**	n=67	n=64	n=66	n=63
LS mean change from baseline (95% CI)	−0.63 (−1.54 to 0.28)	−2.16 (−3.08 to −1.24)	−2.82 (−3.77 to −1.88)	−0.85 (−1.77 to 0.07)
Difference vs placebo–safiglipron 30 mg (95% CI); p value	0.22 (−0.99 to 1.43); p=0.3606	−1.31 (−2.53 to −0.10); p=0.0169	−1.98 (−3.20 to −0.76); p=0.0008	NA
**Bodyweight, % change from baseline**	n=67	n=64	n=66	n=63
LS mean change from baseline (95% CI)	−0.88 (−2.04 to 0.28)	−2.74 (−3.92 to −1.56)	−3.61 (−4.82 to −2.39)	−1.14 (−2.32 to 0.03)
Difference vs placebo–safiglipron 30 mg (95% CI); p value	0.27 (−1.27 to 1.81); p=0.3667	−1.59 (−3.15 to −0.04); p=0.0224	−2.46 (−4.03 to −0.89); p=0.0010	NA
**Waist circumference, cm**	n=67	n=64	n=66	n=63

20

LS mean change from baseline (95% CI)	−0.83 (−1.90 to 0.25)	−2.30 (−3.40 to −1.21)	−3.02 (−4.13 to −1.91)	−1.50 (−2.61 to −0.39)
Difference vs placebo–safiglipron 30 mg (95% CI); p value	0.67 (−0.77 to 2.12); p=0.1809	−0.81 (−2.26 to 0.64); p=0.1380	−1.52 (−2.96 to −0.09); p=0.0187	NA
**Observed-case descriptive endpoints‡**				
Fasting serum insulin, geometric mean ratio to baseline (geometric SD)	1.02 (1.56) n=67	0.93 (1.59) n=64	0.97 (1.54) n=66	1.04 (1.51) n=63
Fasting serum C-peptide, geometric mean ratio to baseline (geometric SD)	1.08 (1.46) n=67	1.10 (1.44) n=64	1.09 (1.36) n=66	1.16 (1.33) n=63
HOMA-β, geometric mean ratio to baseline (geometric SD)	1.43 (1.74) n=67	1.51 (1.77) n=64	1.69 (1.69) n=65	1.45 (1.61) n=63
HOMA-IR, geometric mean ratio to baseline (geometric SD)	0.84 (1.64) n=67	0.72 (1.76) n=64	0.70 (1.69) n=65	0.86 (1.62) n=63
1-h postprandial plasma glucose, mean percentage change from baseline (SD), %	−9.05 (21.20) n=66	−23.50 (21.51) n=63	−21.52 (36.86) n=66	−12.65 (23.28) n=62
2-h postprandial plasma glucose, mean percentage change from baseline (SD), %	−15.42 (25.68) n=66	−28.82 (26.13) n=64	−31.71 (26.23) n=66	−17.02 (23.56) n=62
2-h postprandial serum insulin, geometric mean ratio to baseline (geometric SD)	1.47 (1.60) n=66	1.07 (2.45) n=64	1.14 (1.94) n=66	1.27 (1.87) n=60
2-h postprandial serum C-peptide, geometric mean	1.38 (1.38)	1.16 (1.69)	1.24 (1.46)	1.27 (1.42)

21

ratio to baseline (geometric SD)	n=66	n=64	n=66	n=62
Disposition index, geometric mean ratio to baseline (geometric SD)§	1.55 (1.62) n=66	2.18 (1.79) n=62	2.33 (1.80) n=65	1.64 (1.62) n=58
Mean 7-point self-monitored blood glucose, mean percentage change from baseline (SD), %	−18.75 (18.77) n=66	−24.13 (19.31) n=62	−26.17 (17.31) n=66	−17.84 (14.56) n=60
**Glycaemic target achievement and rescue therapy**				
Participants achieving HbA1c <7.0%, n (%; 95% CI)	44 (62.9; 50.48 to 74.11)	41 (58.6; 46.17 to 70.23)	54 (75.0; 63.40 to 84.46)	33 (45.8; 34.02 to 58.00)
Participants achieving HbA1c ≤6.5%, n (%; 95% CI)	35 (50.0; 37.80 to 62.20)	34 (48.6; 36.44 to 60.83)	41 (56.9; 44.73 to 68.57)	25 (34.7; 23.88 to 46.86)
Participants achieving HbA1c <5.7%, n (%; 95% CI)	8 (11.4; 5.07 to 21.28)	6 (8.6; 3.21 to 17.73)	14 (19.4; 11.06 to 30.47)	2 (2.8; 0.34 to 9.68)
Participants receiving rescue therapy during weeks 0–52, n (%)	0	2 (2.9)	0	7 (9.7)

*Participants initially assigned to placebo received placebo during the 32-week placebo-controlled core period and switched to safiglipron 30 mg from week 33. Therefore, week 52 results are descriptive and do not represent a continuing placebo-controlled comparison.
†Analyses were done in the intention-to-treat population using ANCOVA with retrieved-dropout data incorporated. Data are least-squares mean change from baseline (95% CI) and least-squares mean difference versus the placebo–safiglipron 30 mg group (95% CI). N denotes the number of participants in the intention-to-treat population. n denotes participants with an observed week-52 value; for change-from-baseline and ratio summaries, n denotes participants with both baseline and week-52 values. For ANCOVA with retrieved-dropout data, n describes observed week-52 availability and is not the total number contributing after imputation.
‡Observed-case descriptive endpoints are based on participants with available week 52 data and

22

are presented without formal between-group comparisons.

§Postprandial outcomes and disposition index were derived from the mixed-meal tolerance test.

P values are nominal and were not adjusted for multiplicity.

ANCOVA=analysis of covariance. HbA1c=glycated haemoglobin. HOMA-β=homeostatic model assessment of β-cell function. HOMA-IR=homeostatic model assessment of insulin resistance. LS=least-squares. NA=not applicable.

 23

Supplementary Table 7 | Lipid parameters through week 52

Lipid parameter	Timepoint and measure	Safiglipron 30 mg (N=70)	Safiglipron 60 mg (N=70)	Safiglipron 90 mg (N=72)	Placebo–safiglipron 30 mg (N=72)*
Triglycerides, mmol/L	Baseline, mean (SD)	2.17 (1.63) n=70	2.35 (1.62) n=70	2.21 (1.70) n=72	2.40 (1.51) n=72
	Week 32, geometric mean ratio to baseline (geometric SD)	1.01 (1.64) n=67	1.10 (1.79) n=64	0.91 (1.63) n=66	1.04 (1.74) n=63
	Week 32, geometric mean percentage change from baseline, %	0.99	9.77	−9.17	3.85
	Week 52, geometric mean ratio to baseline (geometric SD)	0.97 (1.59) n=67	0.97 (1.60) n=64	0.82 (1.60) n=66	0.94 (1.52) n=63
	Week 52, geometric mean percentage change from baseline, %	−2.96	−3.42	−17.51	−5.87
Total cholesterol, mmol/L	Baseline, mean (SD)	4.99 (1.08) n=70	5.27 (1.13) n=70	4.88 (1.09) n=72	4.96 (0.96) n=72
	Week 32, geometric mean ratio to baseline (geometric SD)	0.98 (1.19) n=67	0.98 (1.20) n=64	0.94 (1.29) n=66	1.00 (1.17) n=63
	Week 32, geometric mean	−1.78	−2.24	−6.35	−0.29

24

	percentage change from baseline, %				
	Week 52, geometric mean ratio to baseline (geometric SD)	1.03 (1.20) n=67	1.00 (1.21) n=64	0.95 (1.23) n=66	1.03 (1.20) n=63
	Week 52, geometric mean percentage change from baseline, %	2.78	−0.08	−4.86	3.25
LDL cholesterol, mmol/L	Baseline, mean (SD)	3.05 (0.89) n=70	3.29 (1.01) n=70	3.03 (0.92) n=72	3.02 (0.78) n=72
	Week 32, geometric mean ratio to baseline (geometric SD)	0.99 (1.24) n=67	0.93 (1.31) n=64	0.88 (1.54) n=66	1.00 (1.22) n=63
	Week 32, geometric mean percentage change from baseline, %	−0.70	−6.85	−11.70	−0.32
	Week 52, geometric mean ratio to baseline (geometric SD)	1.05 (1.26) n=67	0.99 (1.32) n=64	0.93 (1.34) n=66	1.06 (1.28) n=63
	Week 52, geometric mean percentage change from baseline, %	5.39	−1.50	−7.48	5.75
HDL	Baseline, mean	1.16 (0.23)	1.15 (0.25)	1.17 (0.34)	1.15 (0.26)

25

cholesterol, mmol/L	(SD)	n=70	n=70	n=72	n=72
	Week 32, geometric mean ratio to baseline (geometric SD)	1.00 (1.15) n=67	0.98 (1.18) n=64	1.00 (1.17) n=66	1.00 (1.15) n=63
	Week 32, geometric mean percentage change from baseline, %	0.35	−2.04	−0.32	−0.02
	Week 52, geometric mean ratio to baseline (geometric SD)	1.04 (1.14) n=67	1.03 (1.16) n=64	1.06 (1.16) n=66	1.02 (1.17) n=63
	Week 52, geometric mean percentage change from baseline, %	3.69	3.13	5.95	1.8

Data are observed-case descriptive data from the intention-to-treat population. Baseline values are mean (SD). Postbaseline values are geometric mean ratio to baseline (geometric SD) and geometric mean percentage change from baseline. No formal between-group comparisons were done for this observed-case analysis. N denotes the number of participants in the analysis population. n denotes participants with non-missing data for the specified parameter and visit. For postbaseline ratios and percentage changes, n denotes participants with both baseline and the specified postbaseline value; the same n applies to both summary rows. Missing data were not imputed.

*Participants initially assigned to placebo received placebo during the 32-week placebo-controlled core period and switched to safiglipron 30 mg from week 33; therefore, week 52 data in this group should not be interpreted as continuing placebo-controlled comparisons.

HDL=high-density lipoprotein. LDL=low-density lipoprotein.

26

Supplementary Table 8 | Urinary albumin-to-creatinine ratio through week 52, observed-case analysis

**Timepoint and measure**	**Safiglipron 30 mg (N=70)**	**Safiglipron 60 mg (N=70)**	**Safiglipron 90 mg (N=72)**	**Placebo–safiglipron 30 mg (N=72)***
Baseline, mean (SD), mg/g	23.71 (33.51) n=70	31.74 (92.56) n=70	32.33 (84.90) n=72	26.57 (64.46) n=72
Week 32, geometric mean ratio to baseline (geometric SD)	0.57 (2.86) n=67	0.75 (3.82) n=63	0.64 (3.95) n=66	1.21 (2.80) n=63
Week 32, geometric mean percentage change from baseline, %	−42.72	−24.96	−36.08	20.64
Week 52, geometric mean ratio to baseline (geometric SD)	1.01 (3.08) n=67	1.04 (3.24) n=64	0.85 (2.98) n=66	1.20 (2.73) n=63
Week 52, geometric mean percentage change from baseline, %	1.03	3.71	−14.62	20.50

Data are observed-case descriptive data from the intention-to-treat population. Baseline values are mean (SD). Postbaseline values are geometric mean ratios to baseline (geometric SD) and geometric mean percentage changes from baseline. No formal between-group comparisons were done for this observed-case analysis. N denotes the number of participants in the analysis population. n denotes participants with non-missing data at the specified visit. For postbaseline ratios and percentage changes, n denotes participants with both baseline and the specified postbaseline value; the same n applies to both summary rows. Missing data were not imputed. *Participants initially assigned to placebo received placebo during the 32-week placebo-controlled core period and switched to safiglipron 30 mg from week 33; therefore, week 52 data in this group should not be interpreted as continuing placebo-controlled comparisons. UACR=urinary albumin-to-creatinine ratio.

Supplementary Table 9 | Change from baseline in DTSQs scores during the 52-week treatment period

DTSQs domain	Visit	Safiglipron 30 mg (N=70)	Safiglipron 60 mg (N=70)	Safiglipron 90 mg (N=72)	Placebo-safiglipron 30 mg (N=72)
Satisfaction with current treatment	Baseline	5.1 (1.4) n=70	5.0 (1.4) n=70	5.4 (1.0) n=72	5.2 (1.4) n=72
	Change from baseline at week 32	0.4 (1.5) n=67	0.5 (1.1) n=64	0.2 (1.1) n=65	0.0 (1.7) n=63
	Change from baseline at week 52	0.5 (1.4) n=67	0.7 (1.2) n=64	0.4 (1.0) n=67	0.2 (1.5) n=63
Perceived hyperglycaemia	Baseline	2.0 (1.9) n=70	2.0 (1.8) n=70	2.3 (2.1) n=72	1.8 (1.8) n=72
	Change from baseline at week 32	−0.7 (2.5) n=67	−1.4 (1.8) n=64	−1.4 (2.6) n=65	0.2 (2.1) n=63
	Change from baseline at week 52	−0.6 (2.1) n=67	−1.3 (1.5) n=64	−1.3 (2.6) n=67	−0.3 (2.1) n=63
Perceived hypoglycaemia	Baseline	0.5 (1.2) n=70	0.8 (1.5) n=70	0.7 (1.5) n=72	0.8 (1.4) n=72
	Change from baseline at week 32	0.3 (1.7) n=67	−0.4 (1.5) n=64	−0.2 (2.0) n=65	0.0 (1.7) n=63
	Change from	0.3 (1.6) n=67	−0.3 (1.4) n=64	−0.4 (1.7) n=67	−0.1 (1.8) n=63

28

	baseline at week 52				
Convenience of treatment	Baseline	4.9 (1.4) n=70	4.9 (1.2) n=70	5.1 (1.0) n=72	5.0 (1.3) n=72
	Change from baseline at week 32	0.5 (1.3) n=67	0.5 (1.1) n=64	0.4 (1.2) n=65	0.5 (1.4) n=63
	Change from baseline at week 52	0.5 (1.5) n=67	0.5 (1.4) n=64	0.5 (1.2) n=67	0.5 (1.3) n=63
Flexibility of treatment	Baseline	4.9 (1.3) n=70	5.1 (1.1) n=70	5.3 (0.9) n=72	5.1 (1.2) n=72
	Change from baseline at week 32	0.5 (1.4) n=67	0.3 (1.1) n=64	0.1 (1.1) n=65	0.3 (1.2) n=63
	Change from baseline at week 52	0.5 (1.4) n=67	0.4 (1.0) n=64	0.2 (1.2) n=67	0.4 (1.2) n=63
Satisfaction with understanding of diabetes	Baseline	4.7 (1.3) n=70	4.5 (1.4) n=70	4.8 (1.2) n=72	5.0 (1.3) n=72
	Change from baseline at week 32	0.4 (1.6) n=67	0.7 (1.4) n=64	0.3 (1.4) n=65	0.2 (1.2) n=63
	Change from baseline at week 52	0.7 (1.4) n=67	0.8 (1.4) n=64	0.6 (1.3) n=67	0.3 (1.1) n=63

29

Willingness to recommend treatment	Baseline	4.8 (1.5) n=70	4.9 (1.3) n=70	5.2 (1.0) n=72	5.1 (1.4) n=72
	Change from baseline at week 32	0.2 (1.7) n=67	0.4 (1.0) n=64	0.3 (1.2) n=65	0.0 (1.3) n=63
	Change from baseline at week 52	0.4 (1.6) n=67	0.5 (1.1) n=64	0.3 (1.2) n=67	0.3 (1.2) n=63
Satisfaction with continuing current treatment	Baseline	5.1 (1.2) n=70	5.2 (1.0) n=70	5.3 (1.0) n=72	5.3 (1.2) n=72
	Change from baseline at week 32	0.3 (1.3) n=67	0.4 (0.9) n=64	0.2 (1.4) n=65	0.0 (1.1) n=63
	Change from baseline at week 52	0.2 (1.4) n=67	0.3 (1.0) n=64	0.3 (0.9) n=67	0.0 (1.1) n=63
Total treatment satisfaction score	Baseline	29.6 (6.3) n=70	29.6 (5.7) n=70	31.0 (4.5) n=72	30.7 (6.0) n=72
	Change from baseline at week 32	2.3 (6.5) n=67	2.8 (4.6) n=64	1.4 (5.4) n=65	1.0 (5.5) n=63
	Change from baseline at week 52	2.7 (6.5) n=67	3.3 (5.0) n=64	2.3 (4.9) n=67	1.7 (5.1) n=63

Data are mean (SD). Analyses were based on observed cases in the intention-to-treat population. Participants initially assigned to placebo received placebo during the 32-week

30

placebo-controlled core period and switched to safiglipron 30 mg from week 33 during the extension period. Individual DTSQs item scores range from 0 to 6. For satisfaction with current treatment, convenience of treatment, flexibility of treatment, satisfaction with understanding of diabetes, willingness to recommend treatment, and satisfaction with continuing current treatment, higher scores indicate greater satisfaction. For perceived hyperglycaemia and perceived hypoglycaemia, higher scores indicate more frequent perceived episodes. The total treatment satisfaction score ranges from 0 to 36, with higher scores indicating greater treatment satisfaction. DTSQs=Diabetes Treatment Satisfaction Questionnaire status version. N denotes the number of participants in the intention-to-treat population. n denotes participants contributing observed data to the specified summary; for change-from-baseline summaries, n denotes participants with both baseline and the corresponding postbaseline score. Missing data were not imputed.

31

Supplementary Table 10 | Serious treatment-emergent adverse events during the 32-week placebo-controlled period

	**Safiglipron 30 mg (N=70)**	**Safiglipron 60 mg (N=70)**	**Safiglipron 90 mg (N=72)**	**Placebo (N=72)**
Any serious TEAE	7 (10.0)	2 (2.9)	5 (6.9)	1 (1.4)
Pneumonia	1 (1.4)	0	0	0
Skin infection	0	1 (1.4)	0	0
Gastroenteritis	0	1 (1.4)	0	0
Intercostal neuralgia	0	0	1 (1.4)	0
Facial palsy	0	0	1 (1.4)	0
Headache	1 (1.4)	0	0	0
Arteriosclerosis coronary artery	2 (2.9)	0	0	0
Acute coronary syndrome	0	0	1 (1.4)	0
Intervertebral disc protrusion	0	0	0	1 (1.4)
Arthropathy	1 (1.4)	0	0	0
Nephrolithiasis	0	0	1 (1.4)	0
Ureterolithiasis	0	0	1 (1.4)	0
Lipase increased	0	0	1 (1.4)	0
Foot fracture	1 (1.4)	0	0	0
Localised oedema	1 (1.4)	0	0	0
Pancreatitis acute	1 (1.4)	0	0	0
Lymphoedema	0	1 (1.4)	0	0

Data are n (%). The safety population included all randomly assigned participants who received at least one dose of study drug. Percentages were calculated using the number of participants in each treatment group as the denominator. TEAEs were adverse events that occurred or worsened after the first dose of study drug and during the 32-week placebo-controlled period. Events are listed by Medical Dictionary for Regulatory Activities preferred term. Participants could have more than one serious TEAE.

TEAE=treatment-emergent adverse event.

33

Supplementary Table 11 | Gastrointestinal-related treatment-emergent adverse events by maximum severity during the 32-week placebo-controlled period

	Safiglipron 30 mg (N=70)	Safiglipron 60 mg (N=70)	Safiglipron 90 mg (N=72)	Placebo (N=72)
Any gastrointestinal-related TEAE	31 (44.3)	42 (60.0)	53 (73.6)	11 (15.3)
Mild	21 (30.0)	33 (47.1)	36 (50.0)	9 (12.5)
Moderate	10 (14.3)	8 (11.4)	17 (23.6)	2 (2.8)
Severe	0	1 (1.4)	0	0
Nausea	18 (25.7)	25 (35.7)	33 (45.8)	5 (6.9)
Mild	15 (21.4)	21 (30.0)	25 (34.7)	4 (5.6)
Moderate	3 (4.3)	3 (4.3)	8 (11.1)	1 (1.4)
Severe	0	1 (1.4)	0	0
Diarrhoea	15 (21.4)	14 (20.0)	21 (29.2)	3 (4.2)
Mild	8 (11.4)	9 (12.9)	13 (18.1)	2 (2.8)
Moderate	7 (10.0)	5 (7.1)	8 (11.1)	1 (1.4)
Severe	0	0	0	0
Decreased appetite	6 (8.6)	14 (20.0)	18 (25.0)	2 (2.8)
Mild	6 (8.6)	13 (18.6)	14 (19.4)	2 (2.8)
Moderate	0	0	4 (5.6)	0
Severe	0	1 (1.4)	0	0
Vomiting	9 (12.9)	13 (18.6)	16 (22.2)	0
Mild	8 (11.4)	10 (14.3)	13 (18.1)	0
Moderate	1 (1.4)	3 (4.3)	3 (4.2)	0
Severe	0	0	0	0
Abdominal distension	3 (4.3)	5 (7.1)	11 (15.3)	0
Mild	2 (2.9)	5 (7.1)	10 (13.9)	0
Moderate	1 (1.4)	0	1 (1.4)	0
Severe	0	0	0	0

34

	**Safiglipron 30 mg (N=70)**	**Safiglipron 60 mg (N=70)**	**Safiglipron 90 mg (N=72)**	**Placebo (N=72)**
Constipation	3 (4.3)	2 (2.9)	1 (1.4)	2 (2.8)
Mild	3 (4.3)	2 (2.9)	1 (1.4)	2 (2.8)
Moderate	0	0	0	0
Severe	0	0	0	0

Data are n (%). The safety population included all randomly assigned participants who received at least one dose of study drug. Percentages were calculated using the number of participants in each treatment group as the denominator. TEAEs were adverse events that occurred or worsened after the first dose of study drug and during the 32-week placebo-controlled period. Severity categories show the maximum severity reported for each participant for the corresponding event. Participants could have more than one gastrointestinal or appetite-related TEAE. Events are listed by Medical Dictionary for Regulatory Activities preferred term. TEAE=treatment-emergent adverse event.

35

Supplementary Table 12 | Change from baseline in the worst diabetic retinopathy stage during the 32-week placebo-controlled period

**Change in diabetic retinopathy stage**	**Safiglipron 30 mg (N=70)**	**Safiglipron 60 mg (N=70)**	**Safiglipron 90 mg (N=72)**	**Overall safiglipron (N=212)**	**Placebo (N=72)**
Worsened	1 (1.4)	1 (1.4)	4 (5.6)	6 (2.8)	5 (6.9)
Unchanged	62 (88.6)	61 (87.1)	58 (80.6)	181 (85.4)	59 (81.9)
Improved	0	1 (1.4)	5 (6.9)	6 (2.8)	1 (1.4)
Indeterminate	0	0	0	0	0

Data are n (%) from the safety population. Percentages were calculated using the number of participants in each treatment group as the denominator. The numbers of participants with an evaluable baseline-to-postbaseline change category were n=63, n=63, n=67, n=193 and n=65 for the safiglipron 30 mg, 60 mg and 90 mg groups, overall safiglipron group and placebo group, respectively. For each participant, the worse stage in the left or right eye was used, and the worst postbaseline stage during the 32-week placebo-controlled period was compared with baseline. No formal between-group comparisons were done.

36

Supplementary Table 13 | Treatment-emergent adverse events during the 52-week treatment period

	**Safiglipron 30 mg (N=70)**	**Safiglipron 60 mg (N=70)**	**Safiglipron 90 mg (N=72)**	**Placebo-safiglipron 30 mg (N=72)**
**Overview of adverse events**				
Any TEAE	60 (85.7)	62 (88.6)	68 (94.4)	63 (87.5)
Maximum severity of TEAE				
Mild	28 (40.0)	37 (52.9)	28 (38.9)	27 (37.5)
Moderate	30 (42.9)	24 (34.3)	38 (52.8)	34 (47.2)
Severe	2 (2.9)	1 (1.4)	2 (2.8)	2 (2.8)
TEAE considered related to study treatment	53 (75.7)	49 (70.0)	64 (88.9)	41 (56.9)
Serious TEAE	8 (11.4)	2 (2.9)	8 (11.1)	5 (6.9)
Death	0	0	0	0
TEAE leading to study-drug discontinuation	1 (1.4)	2 (2.9)	5 (6.9)	3 (4.2)
Any gastrointestinal TEAE	32 (45.7)	43 (61.4)	54 (75.0)	26 (36.1)
**Prespecified adverse events of special interest†**				
Any prespecified adverse event of special interest	1 (1.4)	0	0	1 (1.4)
Clinically significant hepatic enzyme abnormality‡	0	0	0	0
Clinically significant calcitonin abnormality§	0	0	0	0
Severe hypoglycaemia¶	0	0	0	0
Acute pancreatitis	1 (1.4)	0	0	1 (1.4)

37

**TEAEs reported in ≥5% of participants in any group**				
Nausea	18 (25.7)	26 (37.1)	33 (45.8)	14 (19.4)
Diarrhoea	15 (21.4)	15 (21.4)	22 (30.6)	7 (9.7)
Decreased appetite	7 (10.0)	14 (20.0)	18 (25.0)	6 (8.3)
Vomiting	9 (12.9)	14 (20.0)	17 (23.6)	5 (6.9)
Hyperuricaemia	11 (15.7)	8 (11.4)	14 (19.4)	5 (6.9)
Abdominal distension	4 (5.7)	5 (7.1)	11 (15.3)	0
Upper respiratory tract infection	10 (14.3)	11 (15.7)	10 (13.9)	13 (18.1)
Lipase increased	7 (10.0)	4 (5.7)	9 (12.5)	5 (6.9)
Asthenia	0	5 (7.1)	9 (12.5)	0
Urinary tract infection	3 (4.3)	3 (4.3)	7 (9.7)	6 (8.3)
Flatulence	2 (2.9)	3 (4.3)	6 (8.3)	1 (1.4)
Alanine aminotransferase increased	4 (5.7)	5 (7.1)	6 (8.3)	6 (8.3)
Blood bilirubin increased	7 (10.0)	1 (1.4)	6 (8.3)	3 (4.2)
Abdominal pain	2 (2.9)	3 (4.3)	5 (6.9)	1 (1.4)
Hyperlipidaemia	3 (4.3)	5 (7.1)	5 (6.9)	5 (6.9)
Diabetic retinopathy	3 (4.3)	1 (1.4)	5 (6.9)	5 (6.9)
Upper abdominal pain	2 (2.9)	3 (4.3)	4 (5.6)	4 (5.6)
Protein urine present	1 (1.4)	0	4 (5.6)	6 (8.3)
Amylase increased	1 (1.4)	3 (4.3)	4 (5.6)	1 (1.4)
Blood calcitonin increased	0	2 (2.9)	4 (5.6)	2 (2.8)
Dizziness	3 (4.3)	7 (10.0)	4 (5.6)	4 (5.6)
Sinus tachycardia	1 (1.4)	1 (1.4)	4 (5.6)	0
Arteriosclerosis coronary	2 (2.9)	0	4 (5.6)	0

38

artery				
Aspartate aminotransferase increased	3 (4.3)	2 (2.9)	3 (4.2)	5 (6.9)
Dyslipidaemia	2 (2.9)	4 (5.7)	3 (4.2)	3 (4.2)
Dyspepsia	4 (5.7)	2 (2.9)	2 (2.8)	2 (2.8)
Gamma-glutamyltransferase increased	6 (8.6)	3 (4.3)	2 (2.8)	3 (4.2)
Anaemia	4 (5.7)	2 (2.9)	2 (2.8)	1 (1.4)
Proteinuria	1 (1.4)	1 (1.4)	1 (1.4)	5 (6.9)
Blood uric acid increased	1 (1.4)	4 (5.7)	0	2 (2.8)
Headache	4 (5.7)	2 (2.9)	0	1 (1.4)

Data are n (%). The safety population included all randomly assigned participants who received at least one dose of study drug. Participants initially assigned to placebo received placebo during the 32-week placebo-controlled core period and switched to safiglipron 30 mg from week 33 during the extension period. Percentages were calculated using the number of participants in each treatment group as the denominator. TEAEs were adverse events that occurred or worsened after the first dose of study drug and during the 52-week treatment period. Maximum severity refers to the highest severity reported for each participant. Study treatment-related TEAEs were events considered by the investigator to be related to study treatment. TEAEs reported in ≥5% of participants in any treatment group are listed by Medical Dictionary for Regulatory Activities preferred term.

†Prespecified adverse events of special interest were clinically significant hepatic enzyme abnormalities, clinically significant calcitonin abnormalities, acute pancreatitis, and severe hypoglycaemia.

‡Clinically significant hepatic enzyme abnormalities were protocol-defined elevations in ALT or AST and total bilirubin meeting prespecified thresholds in the absence of alternative explanations or evidence of cholestasis; full criteria are provided in the study protocol.

§Clinically significant calcitonin abnormality was defined as postbaseline calcitonin concentration of at least 50 ng/L and an increase of more than 50% from baseline.

¶Severe hypoglycaemia was defined according to American Diabetes Association level 3 criteria as a severe event characterised by altered mental or physical status requiring assistance from another person, without a specific glucose threshold.

39

ALT=alanine aminotransferase. AST=aspartate aminotransferase. TEAE=treatment-emergent adverse event.

40

Supplementary Table 14 | Serious treatment-emergent adverse events during the 52-week treatment period

	Safiglipron 30 mg (N=70)	Safiglipron 60 mg (N=70)	Safiglipron 90 mg (N=72)	Placebo-safiglipron 30 mg (N=72)
Any serious TEAE	8 (11.4)	2 (2.9)	8 (11.1)	5 (6.9)
Arteriosclerosis coronary artery	2 (2.9)	0	1 (1.4)	0
Atrial fibrillation	0	0	1 (1.4)	0
Acute coronary syndrome	0	0	1 (1.4)	0
Pneumonia	1 (1.4)	0	0	0
Skin infection	0	1 (1.4)	0	0
Gastroenteritis	0	1 (1.4)	0	0
Lower respiratory tract infection	1 (1.4)	0	0	0
Cervical radiculopathy	0	0	1 (1.4)	0
Intercostal neuralgia	0	0	1 (1.4)	0
Facial palsy	0	0	1 (1.4)	0
Headache	1 (1.4)	0	0	0
Intervertebral disc protrusion	0	0	1 (1.4)	1 (1.4)
Osteoarthritis	0	0	0	1 (1.4)
Arthropathy	1 (1.4)	0	0	0
Cervical spinal stenosis	0	0	1 (1.4)	0
Ureterolithiasis	0	0	1 (1.4)	1 (1.4)
Nephrolithiasis	0	0	1 (1.4)	0
Pancreatitis acute	1 (1.4)	0	0	1 (1.4)

41

Vestibular disorder	0	0	0	1 (1.4)
Lipase increased	0	0	1 (1.4)	0
Foot fracture	1 (1.4)	0	0	0
Chronic obstructive pulmonary disease	1 (1.4)	0	0	0
Localised oedema	1 (1.4)	0	0	0
Lymphoedema	0	1 (1.4)	0	0

Data are n (%). The safety population included all randomly assigned participants who received at least one dose of study drug. Participants initially assigned to placebo received placebo during the 32-week placebo-controlled core period and switched to safiglipron 30 mg from week 33 during the extension period. Percentages were calculated using the number of participants in each treatment group as the denominator. TEAEs were adverse events that occurred or worsened after the first dose of study drug and during the 52-week treatment period. Events are listed by Medical Dictionary for Regulatory Activities preferred term. Participants could have more than one serious TEAE. TEAE=treatment-emergent adverse event.

42

Supplementary Table 15 | Treatment-emergent adverse events leading to study treatment discontinuation during the 52-week treatment period

	**Safiglipron 30 mg (N=70)**	**Safiglipron 60 mg (N=70)**	**Safiglipron 90 mg (N=72)**	**Placebo-safiglipron 30 mg (N=72)**
Any TEAE leading to study treatment discontinuation	1 (1.4)	2 (2.9)	5 (6.9)	3 (4.2)
Nausea	0	2 (2.9)	2 (2.8)	1 (1.4)
Vomiting	0	1 (1.4)	2 (2.8)	1 (1.4)
Pancreatitis acute	1 (1.4)	0	0	1 (1.4)
Constipation	0	0	0	1 (1.4)
Diarrhoea	0	0	0	1 (1.4)
Duodenitis	0	0	1 (1.4)	0
Dyspepsia	0	0	0	1 (1.4)
Asthenia	0	0	2 (2.8)	0
Decreased appetite	0	1 (1.4)	0	0
Tinnitus	0	0	1 (1.4)	0
Lipase increased	0	0	1 (1.4)	0
Dizziness	0	0	1 (1.4)	0

Data are n (%). The safety population included all randomly assigned participants who received at least one dose of study drug. Participants initially assigned to placebo received placebo during the 32-week placebo-controlled core period and switched to safiglipron 30 mg from week 33 during the extension period. Percentages were calculated using the number of participants in each treatment group as the denominator. TEAEs were adverse events that occurred or worsened after the first dose of study drug and during the 52-week treatment period. Events are listed by Medical Dictionary for Regulatory Activities preferred term. Participants could have more than one TEAE leading to study-drug discontinuation. TEAE=treatment-emergent adverse event.

43

Supplementary Table 16 | Postbaseline liver-function laboratory abnormalities during the 52-week treatment period

**Laboratory abnormality**	**Safiglipron 30 mg (N=70)**	**Safiglipron 60 mg (N=70)**	**Safiglipron 90 mg (N=72)**	**Placebo–safiglipron 30 mg (N=72)**
ALT >3 × ULN	0	0	2 (2.8%)	2 (2.8%)
ALT >5 × ULN	0	0	0	1 (1.4%)
AST >3 × ULN	0	0	0	1 (1.4%)
AST >5 × ULN	0	0	0	1 (1.4%)
Total bilirubin >2 × ULN	3 (4.3%)	0	1 (1.4%)	0
ALT >3 × ULN or AST >3 × ULN	0	0	2 (2.8%)	2 (2.8%)
ALT >3 × ULN or AST >3 × ULN, and total bilirubin >2 × ULN	0	0	0	0

Data are n (%) of participants with at least one postbaseline liver-function laboratory abnormality meeting the specified threshold during the 52-week treatment period. Percentages were calculated using the safety population as the denominator. No formal between-group comparisons were done.

ALT=alanine aminotransferase. AST=aspartate aminotransferase. ULN=upper limit of normal.

44

Supplementary Table 17 | Selected hepatic, pancreatic and thyroid-related laboratory parameters through week 52

Laboratory parameter	Timepoint and measure	Safiglipron 30 mg (N=70)	Safiglipron 60 mg (N=70)	Safiglipron 90 mg (N=72)	Placebo–safiglipron 30 mg (N=72)*
Alanine aminotransferase, U/L	Baseline, mean (SD)	29.73 (14.72) n=70	31.46 (17.99) n=70	31.63 (16.01) n=72	33.01 (19.20) n=72
	Week 32, geometric mean ratio to baseline (geometric SD)	0.91 (1.44) n=67	0.80 (1.58) n=64	0.75 (1.76) n=65	0.94 (1.49) n=63
	Week 32, geometric mean percentage change from baseline, %	−9.07	−19.68	−24.85	−5.52
	Week 52, geometric mean ratio to baseline (geometric SD)	0.90 (1.40) n=67	0.83 (1.51) n=64	0.75 (1.68) n=67	0.94 (1.56) n=63
	Week 52, geometric mean percentage change from baseline, %	−9.59	−16.82	−24.67	−6.03
Aspartate	Baseline,	23.41 (7.10)	23.19 (9.43)	24.89	25.62

45

aminotransferase, U/L	mean (SD)	n=70	n=70	(10.74) n=72	(10.96) n=72
	Week 32, geometric mean ratio to baseline (geometric SD)	0.98 (1.29) n=67	0.93 (1.34) n=64	0.93 (1.52) n=65	0.98 (1.40) n=63
	Week 32, geometric mean percentage change from baseline, %	−1.84	−6.67	−7.24	−1.61
	Week 52, geometric mean ratio to baseline (geometric SD)	0.98 (1.28) n=67	0.96 (1.33) n=64	0.92 (1.44) n=67	0.98 (1.39) n=63
	Week 52, geometric mean percentage change from baseline, %	−1.68	−4.39	−8.24	−2.15
Amylase, U/L	Baseline, mean (SD)	56.78 (21.12) n=70	66.20 (22.67) n=70	57.38 (21.64) n=72	58.95 (18.83) n=72
	Week 32, geometric mean ratio to baseline (geometric	1.08 (1.18) n=67	1.07 (1.29) n=64	1.12 (1.23) n=65	1.01 (1.29) n=63

46

	SD)				
	Week 32, geometric mean percentage change from baseline, %	8.32	6.66	11.75	1.32
	Week 52, geometric mean ratio to baseline (geometric SD)	1.11 (1.17) n=67	1.05 (1.28) n=64	1.19 (1.27) n=67	1.09 (1.24) n=63
	Week 52, geometric mean percentage change from baseline, %	11.38	5.09	19.06	9.15
Lipase, U/L	Baseline, mean (SD)	47.76 (38.95) n=70	60.40 (50.88) n=70	46.90 (36.36) n=72	36.80 (21.86) n=72
	Week 32, geometric mean ratio to baseline (geometric SD)	1.13 (1.35) n=67	1.11 (1.65) n=64	1.17 (1.44) n=65	1.04 (1.47) n=63
	Week 32, geometric mean percentage change from baseline, %	12.94	11.29	17.39	4.29

47

	Week 52, geometric mean ratio to baseline (geometric SD)	1.22 (1.44) n=67	1.17 (1.56) n=64	1.31 (1.53) n=67	1.21 (1.44) n=63
	Week 52, geometric mean percentage change from baseline, %	21.9	17.31	31.49	21.39
Calcitonin, ng/L	Baseline, mean (SD)	2.09 (2.32) n=70	2.98 (3.10) n=70	3.00 (3.51) n=72	3.29 (3.84) n=72
	Week 32, geometric mean ratio to baseline (geometric SD)	1.01 (2.41) n=67	0.99 (1.93) n=64	1.02 (1.67) n=65	0.83 (1.92) n=62
	Week 32, geometric mean percentage change from baseline, %	0.57	−0.79	1.8	−17.17
	Week 52, geometric mean ratio to baseline (geometric SD)	0.99 (2.50) n=66	1.02 (1.95) n=64	0.98 (1.73) n=67	0.93 (2.04) n=62
	Week 52, geometric	−0.67	1.6	−1.93	−7.24

48

	mean percentage change from baseline, %				

Data are observed-case descriptive data from the safety population. Baseline values are mean (SD). Postbaseline values are geometric mean ratio to baseline (geometric SD) and geometric mean percentage change from baseline. No formal between-group comparisons were done for this observed-case analysis. N denotes the number of participants in the analysis population. n denotes participants with non-missing data for the specified parameter and visit. For postbaseline ratios and percentage changes, n denotes participants with both baseline and the specified postbaseline value; the same n applies to both summary rows. Missing data were not imputed.
*Participants initially assigned to placebo received placebo during the 32-week placebo-controlled core period and switched to safiglipron 30 mg from week 33; therefore, week 52 data in this group should not be interpreted as continuing placebo-controlled comparisons.
ALT=alanine aminotransferase. AST=aspartate aminotransferase.

49

Supplementary Table 18 | Vital signs through week 52

Vital sign	Timepoint and measure	Safiglipron 30 mg (N=70)	Safiglipron 60 mg (N=70)	Safiglipron 90 mg (N=72)	Placebo–safiglipron 30 mg (N=72)*
Systolic blood pressure, mm Hg	Baseline, mean (SD)	122.93 (11.05) n=70	123.97 (11.73) n=70	124.06 (11.94) n=72	123.71 (11.05) n=72
	Week 32, mean change from baseline (SD), mm Hg	−6.18 (12.26) n=67	−5.94 (12.33) n=64	−5.80 (12.98) n=65	−2.52 (10.44) n=63
	Week 32, mean percentage change from baseline (SD), %	−4.62 (9.68)	−4.56 (9.64)	−4.11 (10.18)	−1.69 (8.25)
	Week 52, mean change from baseline (SD), mm Hg	−3.32 (11.54) n=67	−1.74 (14.14) n=64	−4.32 (11.41) n=67	−1.42 (9.82) n=63
	Week 52, mean percentage change from baseline (SD), %	−2.27 (9.29)	−0.87 (11.48)	−3.08 (9.03)	−0.78 (7.80)
Diastolic blood pressure, mm Hg	Baseline, mean (SD)	83.74 (7.89) n=70	82.50 (8.26) n=70	82.22 (8.07) n=72	83.15 (6.32) n=72
	Week 32, mean change from baseline (SD), mm Hg	−3.50 (7.69) n=67	−2.17 (8.38) n=64	−2.46 (8.25) n=65	−2.35 (6.62) n=63

50

Vital sign	Timepoint and measure	Safiglipron 30 mg (N=70)	Safiglipron 60 mg (N=70)	Safiglipron 90 mg (N=72)	Placebo–safiglipron 30 mg (N=72)*
	Week 32, mean percentage change from baseline (SD), %	−3.98 (9.19)	−2.19 (10.36)	−2.47 (10.00)	−2.59 (8.00)
	Week 52, mean change from baseline (SD), mm Hg	−2.87 (7.57) n=67	−0.21 (9.78) n=64	−2.39 (7.76) n=67	−0.57 (7.12) n=63
	Week 52, mean percentage change from baseline (SD), %	−3.05 (9.02)	0.31 (12.03)	−2.45 (9.33)	−0.47 (8.59)
Pulse rate, beats/min	Baseline, mean (SD)	80.67 (11.16) n=70	78.48 (9.35) n=70	80.14 (8.93) n=72	78.38 (9.62) n=72
	Week 32, mean change from baseline (SD), beats/min	−1.87 (10.43) n=67	2.60 (9.56) n=64	1.47 (10.04) n=65	−1.88 (8.57) n=63
	Week 52, mean change from baseline (SD), beats/min	−2.06 (10.82) n=67	1.66 (9.13) n=64	0.21 (8.88) n=67	1.13 (7.96) n=63

Data are observed-case descriptive data from the safety population. Baseline values are mean (SD). Postbaseline values are mean change from baseline (SD) and, for blood pressure, mean percentage change from baseline (SD). No formal between-group comparisons were done for this observed-case analysis. N denotes the number of participants in the safety population. n denotes participants contributing observed data to the specified summary; for postbaseline changes and percentage changes, n denotes participants with both baseline and the specified postbaseline value, and the same n applies to both blood-pressure summary rows. Missing data were not imputed.

 51

*Participants initially assigned to placebo received placebo during the 32-week placebo-controlled core period and switched to safiglipron 30 mg from week 33; therefore, week 52 data in this group should not be interpreted as continuing placebo-controlled comparisons.

52

Supplementary Table 19 | Electrocardiographic parameters through week 52

Electrocardiographic parameter	Timepoint and measure	Safiglipron 30 mg (N=70)	Safiglipron 60 mg (N=70)	Safiglipron 90 mg (N=72)	Placebo–safiglipron 30 mg (N=72)*
Heart rate, beats/min	Baseline, mean (SD)	75.9 (10.22) n=70	75.2 (10.52) n=70	76.6 (10.92) n=72	73.8 (9.73) n=72
	Week 32, mean change from baseline (SD), beats/min	1.4 (9.54) n=67	2.5 (10.42) n=64	3.0 (12.19) n=65	1.6 (9.77) n=63
	Week 52, mean change from baseline (SD), beats/min	2.6 (9.85) n=67	3.5 (9.91) n=64	2.3 (10.85) n=67	5.8 (9.13) n=63
PR interval, ms	Baseline, mean (SD)	159.5 (20.48) n=70	162.7 (20.88) n=70	157.4 (17.87) n=72	157.5 (19.29) n=72
	Week 32, mean change from baseline (SD), ms	2.6 (13.35) n=67	3.0 (15.83) n=64	1.5 (11.78) n=65	1.6 (10.60) n=63
	Week 52, mean change from	0.7 (12.28) n=67	1.0 (14.16) n=64	1.5 (10.65) n=67	−0.4 (9.92) n=63

53

Electrocardiographic parameter	Timepoint and measure	Safiglipron 30 mg (N=70)	Safiglipron 60 mg (N=70)	Safiglipron 90 mg (N=72)	Placebo–safiglipron 30 mg (N=72)*
	baseline (SD), ms				
QT interval, ms	Baseline, mean (SD)	376.6 (27.25) n=70	375.8 (28.55) n=70	375.6 (26.56) n=72	384.7 (27.85) n=72
	Week 32, mean change from baseline (SD), ms	−1.3 (27.22) n=67	−1.7 (23.61) n=64	−4.3 (24.85) n=65	−4.4 (26.78) n=63
	Week 52, mean change from baseline (SD), ms	0.6 (25.21) n=67	−4.5 (24.72) n=64	−2.7 (23.06) n=67	−13.2 (23.43) n=63
QTcF interval, ms	Baseline, mean (SD)	405.17 (21.53) n=70	403.19 (24.78) n=70	405.30 (22.59) n=72	410.28 (20.16) n=72
	Week 32, mean change from baseline (SD), ms	1.10 (22.77) n=67	2.18 (22.31) n=64	−0.05 (21.11) n=65	−1.81 (22.15) n=63
	Week 52, mean change from	5.18 (22.09) n=67	1.27 (24.89) n=64	0.90 (24.78) n=67	−3.86 (18.09) n=63

54

Electrocardiographic parameter	Timepoint and measure	Safigliptron 30 mg (N=70)	Safiglipron 60 mg (N=70)	Safiglipron 90 mg (N=72)	Placebo–safiglipron 30 mg (N=72)*
	baseline (SD), ms				
RR interval, ms	Baseline, mean (SD)	806.0 (118.87) n=70	812.7 (117.28) n=70	800.9 (119.80) n=72	826.6 (109.73) n=72
	Week 32, mean change from baseline (SD), ms	−14.4 (102.80) n=67	−12.7 (98.40) n=64	−28.8 (126.64) n=65	−20.7 (103.16) n=63
	Week 52, mean change from baseline (SD), ms	−25.8 (104.98) n=67	−36.4 (100.14) n=64	−24.9 (120.51) n=67	−64.9 (107.93) n=63

Data are observed-case descriptive data from scheduled 12-lead electrocardiograms in the safety population. Baseline values are mean (SD), and postbaseline values are mean change from baseline (SD). No formal between-group comparisons were done. N denotes the number of participants in the safety population. n denotes participants contributing observed data to the specified summary; for postbaseline changes, n denotes participants with both baseline and the specified postbaseline value. Missing data were not imputed.

*Participants initially assigned to placebo received placebo during the 32-week placebo-controlled core period and switched to safiglipron 30 mg from week 33; therefore, week 52 data in this group should not be interpreted as continuing placebo-controlled comparisons.

QTcF=QT interval corrected for heart rate using Fridericia's formula.

Supplementary References

1. Rubin, D. B. Multiple Imputation for Nonresponse in Surveys (Wiley, 1987). https://doi.org/10.1002/9780470316696

56

A MULTICENTER, RANDOMIZED, DOUBLE-BLIND, PLACEBO-CONTROLLED, PARALLEL-GROUP, PHASE III STUDY TO EVALUATE THE EFFICACY AND SAFETY OF HRS-7535 IN ADULT SUBJECTS WITH TYPE 2 DIABETES MELLITUS INADEQUATELY CONTROLLED BY DIET AND EXERCISE ALONE

Protocol No.: HRS-7535-301
Study Phase: Phase III
Compound No.: HRS-7535
Protocol Lead: Zi Ye
Protocol Version No. and Date: 2.0, 09 Sep 2024

Sponsor: Shandong Suncadia Medicine Co., Ltd.
Biomedical Park, 1777 Dazheng Road, High-tech Zone, Jinan, Shandong
Building 5, Room 102, Industrialization Base for Small and Medium Enterprises

Confidentiality Statement

The information contained in this protocol is confidential and is for use by clinical investigators only, and shall not be disclosed unless required by current laws or regulations. Its copyright is owned by Jiangsu Hengrui Pharmaceuticals Co., Ltd. or its subsidiaries, and it shall not be duplicated or distributed to any person who has not participated in this clinical trial, except for those who have signed a confidentiality agreement with Jiangsu Hengrui Pharmaceuticals Co., Ltd. or its subsidiaries.

PROTOCOL AMENDMENT HISTORY AND CURRENT VERSION AMENDMENT

This study protocol is version 2.0.

Protocol Amendment History

Version No.	Version Date	Enrollment rate estimates
2.0	September 09, 2024	Not applicable
1.0	03 Jul 2024	Not applicable

Overview of Protocol Amendments in Current Version

**Version No.**	**2.0**	
**Enrollment rate estimates**	Not applicable	
**Reason for Revision**	Main reason for amendment:<br>☒ Revision requested by the drug regulatory authority<br>☐New regulatory guidance requirements<br>☐Institutional Review Board Feedback<br>☐New safety data<br>☐Manufacturing Changes<br>☐New Investigational Product (in case of adaptive trial)<br>☐Change in strategy<br>☐Changes in Standard of Care<br>☐New non-safety data<br>☐Investigator/Site Feedback<br>☐Difficulty in recruiting<br>☐Protocol inconsistencies and/or errors<br>☐Protocol design error<br>☐Others	Other reason for amendment:<br>☐ Revision requested by the drug regulatory authority<br>☐New regulatory guidance requirements<br>☐Institutional Review Board Feedback<br>☐New safety data<br>☐Manufacturing Changes<br>☐New Investigational Product (in case of adaptive trial)<br>☐Change in strategy<br>☐Changes in Standard of Care<br>☐New non-safety data<br>☐Investigator/Site Feedback<br>☐Difficulty in recruiting<br>☒Protocol inconsistencies and/or errors<br>☐Protocol design error<br>☐Others
**Summary of Revisions**	According to the requirements of the drug regulatory authority, the duration of the double-blind treatment period was extended to 52 weeks, with additional Visits and procedures added, and the corresponding study endpoints were modified.	

**Is the current revision likely to have a significant impact on any of the following:**<br>• Safety of clinical trial subjects;<br>• The scientific nature of the clinical trial;<br>• The reliability of the generation of clinical trial data.	☐Yes No☒

Summary of Changes to the Current Version of the Protocol

Section Number and Name	Description of Change	Brief Rationale for Changes
Figure 1 Study Schematic Diagram	• Extend the double-blind treatment period to 52 weeks, with additional Visits and procedures.	• Revision requested by the drug regulatory authority

1.3. Schedule of Activities		
Table 1. Schedule of Activities<br>8.3.3. 12-lead ECG	• 12-lead electrocardiogram increased PR interval data measurement.	• The 12-lead electrocardiogram should measure and collect PR interval data to obtain more complete ECG-related safety data.
3.1. Objectives and Endpoints	• Extend the double-blind treatment period to 52 weeks, and modify and supplement the corresponding endpoints.	• Revision requested by the drug regulatory authority
4.1. Study Design Description	• Extend the double-blind treatment period to 52 weeks, with corresponding modifications to the trial design.	• Revision requested by the drug regulatory authority
5.3. Inclusion Criteria	• The lower limit of HbA1c for inclusion was relaxed from 7.5% to 7.0%.	• The inclusion criteria were relaxed to align with clinical practice.
Dosing Regimen	• Extend the double-blind treatment period to 52 weeks, with corresponding modifications to the dosing regimen.	• Revision requested by the drug regulatory authority
6.8.4. Rescue Treatment	• Extend the double-blind treatment period to 52 weeks, with corresponding modifications to the rescue treatment.	• As required by the drug regulatory authority, the double-blind treatment period was extended to 52 weeks, and the description of rescue treatment from 33 to 52 weeks was added.
8.3.2. Laboratory Determinations	• The term “urea nitrogen” was modified to “urea nitrogen or serum urea”.	• Some local laboratories at the centers did not have Urea nitrogen test, only Urea test item, and the test item description was improved.
8.6. Pharmacokinetics	• Extend the double-blind treatment period to 52 weeks, with corresponding increases in PK blood sampling procedures.	• Revision requested by the drug regulatory authority
9.8. Sample size determination	• Improve the description of the basis for overall efficacy prediction.	• Revision requested by the drug regulatory authority
/	• Minor corrections	• Corrected clerical errors or inconsistencies in the protocol.

Information on previous protocol amendments is summarized in Section *13.4*

TABLE OF CONTENTS

Table of Contents ... 3
LIST OF TABLES ... 6
List of Figures ... 6
1. Protocol Summary ... 7
1.1. Protocol Synopsis ... 7
1.2. Schematic Diagram of Trial ... 20
1.3. Schedule of Activities ... 21
2. Introduction ... 28
2.1. Study Background ... 28
2.1.1. Disease Background ... 28
2.1.2. HRS-7535 ... 28
2.1.3. Non-Clinical Experience ... 29
2.1.4. Clinical Experience ... 31
2.2. Summary of Benefits and Risks ... 33
2.2.1. Benefit Summary ... 33
2.2.2. Risk Summary and Management Measures ... 33
2.2.3. Overall Benefit-Risk Conclusion ... 34
3. Study Objectives, Endpoints, and Estimands ... 34
3.1. Objectives and Endpoints ... 34
3.2. Estimands ... 36
3.2.1. Primary Estimand ... 36
3.2.2. Supplementary Estimand ... 36
4. Study Design ... 37
4.1. Study Design Description ... 37
4.2. Trial Design Basis ... 37
4.3. Access to Study Intervention After End of Study ... 38
4.4. Definition of Trial Initiation and Completion ... 38
5. Trial Population ... 39
5.1. Selection of Trial Population ... 39
5.2. Rationale for Selection of Trial Population ... 39
5.3. Inclusion Criteria ... 39
5.4. Exclusion Criteria ... 40
5.5. Lifestyle Requirements ... 43
5.6. Screen failure ... 43
6. Trial Intervention and Concomitant Treatment ... 44
6.1. Information of Investigational Intervention ... 44
6.2. Selection of Trial Intervention and Basis ... 45
6.2.1. Rationale for Dose Selection ... 45
6.3. Dosage and Administration ... 45

6.3.1. Dosing Regimen....45
6.3.2. Medication Guidance....47
6.3.3. Dose Adjustment....47
6.3.3.1. Gastrointestinal System Symptom Management....47
6.3.3.2. Temporary Dose Interruption....49
6.4. Overdose Treatment....49
6.5. Medicinal Product Preparation, Handling, Storage and Accountability....50
6.5.1. Preparation of medicinal product....50
6.5.2. Medicinal Product Handling and Storage....50
6.5.3. Management of Medicinal Products....51
6.5.3.1. Medicinal Product Destruction....51
6.6. Subject Assignment, Randomization, and Blinding....51
6.6.1. Subject Assignment....51
6.6.2. Randomization....52
6.6.2.1. Randomization Criteria....52
6.6.3. Blinding and Unblinding....53
6.7. Compliance with Trial Intervention....53
6.8. Concomitant treatment....54
6.8.1. Medications for Gastrointestinal Symptoms....54
6.8.2. Prohibited Concomitant Treatment....55
6.8.3. Permitted Concomitant Treatment....56
6.8.4. Rescue Treatment....56
7. Termination of Trial Intervention or Withdrawal from Trial....57
7.1. Termination of Trial Intervention for Subject....57
7.1.1. Discontinuation of Trial Intervention....57
7.2. Withdrawal of Subject from the Trial....58
7.3. Lost to Follow-up....58
7.4. Specific Discontinuation Criteria (Not Applicable)....59
8. Trial Evaluation and Procedure....59
8.1. Screening and Run-in phase Assessments and Procedures....60
8.2. Assessment of Efficacy and Procedures....60
8.2.1. Glycated hemoglobin (HbA1c)....60
8.2.2. Fasting Plasma Glucose (FPG)....60
8.2.3. Self-measured blood glucose (SMBG)....60
8.2.4. Mixed meal test....61
8.2.5. Body weight and waist circumference under fasting conditions....62
8.3. Safety Evaluation and Procedures....63
8.3.1. Vital signs and physical examination....63
8.3.2. Laboratory Tests....64
8.3.3. 12-Lead ECG....64
8.3.4. Screening and Assessment of Diabetic Retinopathy....65

8.4. Adverse Events (AEs) and Serious Adverse Events (SAEs) 66
8.4.1. Definition of AEs and SAEs 66
8.4.2. Collection of AEs and SAEs 67
8.4.3. Identification of AEs and SAEs 67
8.4.4. Recording of AEs and SAEs 67
8.4.5. Follow-up of AEs/SAEs 68
8.4.6. SAE Reporting System 68
8.4.7. Regulatory Reporting Requirements for SAEs 68
8.4.8. Suspected Unexpected Serious Adverse Reaction Report 68
8.4.9. Adverse events of special interest (AESI) 69
8.4.9.1. Abnormal Liver Function Tests 69
8.4.9.2. Hypoglycemic episodes 70
8.5. Pregnancy 71
8.6. Pharmacokinetics 71
8.7. Genetics (Not Applicable) 72
8.8. Biomarkers (Not Applicable) 72
8.9. Immunogenicity (Not applicable) 72
8.10. Medical Resource Utilization and Health Economics (Not Applicable) 72
9. Statistical Considerations 72
9.1. Analysis Population 72
9.2. Supportive analysis for primary objective 72
9.2.1. Statistical Hypothesis and Decision Rules 72
9.2.2. Primary Estimand Analysis 73
9.2.3. Supplementary Estimand Analysis 74
9.3. Analysis supporting secondary objectives 74
9.3.1. Analysis of secondary efficacy endpoints 74
9.3.2. Analysis of Other Secondary Endpoints 74
9.4. Analyses to Support Exploratory Objectives 75
9.5. Safety Analysis 75
9.6. Other Analyses 75
9.6.1. Pharmacokinetic analysis 75
9.6.2. Subgroup analysis 75
9.7. Interim analysis (not Applicable) 76
9.8. Sample size determination 76
9.9. Protocol deviations 76
10. GENERAL CONSIDERATIONS: REGULATORY, ETHICAL, AND STUDY OVERSIGHT 76
10.1. Regulatory and Ethical Considerations 76
10.2. Committees (Not Applicable) 77
10.3. Informed consent process 77
10.4. Data Protection 77
10.4.1. Confidentiality of Subject Information 77
10.4.2. Data Protection 78
10.5. Early termination or suspension of trial 79

11. General Considerations: Risk Management and Quality Assurance 79
11.1. Data Quality Assurance 79
11.2. Source Data 80
12. APPENDIX: ADVERSE EVENTS AND SERIOUS ADVERSE EVENTS - DEFINITIONS, SEVERITY, AND CAUSALITY 81
12.1. Definition and Supplement of AE...81
12.1.1. Drug Overdose 81
12.2. Description and Supplement of SAE Definition..81
12.2.1. Hospitalization 81
12.3. Severity 82
12.4. Causality 82
13. APPENDIX: DEFINITIONS AND SUPPORTING OPERATIONAL DETAILS..........83
13.1. Contraception and Pregnancy Test 83
13.1.1. Definitions related to fertility...83
13.1.2. Contraception..83
13.1.3. Pregnancy test and FSH test 84
13.2. Laboratory Tests 85
13.3. Country-Specific Variations (not Applicable) 85
13.4. Protocol Amendment History 85
13.5. CKD-EPI Formula 86
14. APPENDIX: ABBREVIATIONS 87
15. APPENDIX: REFERENCES...89

LIST OF TABLES

Table 1. Study Flowchart...21
Table 2. Study Objectives and Endpoints 34
Table 3. Definition of Primary Estimand and Handling Strategies 36
Table 4. Investigational Product Information 44
Table 5. Drug Dosage and Corresponding Number of Tablets During the Trial 46
Table 6. Rescue Treatment Criteria for Fasting Plasma Glucose Levels 56
Table 7. Laboratory Test Items..64
Table 8. Collection of Clinical Trial Safety Events 67
Table 9. Criteria for Determining Abnormal Liver Function Tests..........................69
Table 10. 2024 ADA-Recommended Classification of Hypoglycemic Episodes 70

LIST OF FIGURES

Figure 1. Overview of study design...20

1. Protocol Summary

1.1. Protocol Synopsis

**PROTOCOL TITLE**	A multicenter, randomized, double-blind, placebo-controlled, parallel-group Phase III study to evaluate the efficacy and safety of HRS-7535 in adult subjects with type 2 diabetes mellitus with inadequate glycemic control by diet and exercise alone
**Protocol No.:**	HRS-7535-301
**Version No. and Date**	Version 2.0, Sep. 09, 2024
**Study phase: phase**	Phase III
**Compound number**	HRS-7535
**Sponsor**	Shandong Suncadia Medicine Co., Ltd.
**Participating Site(s)**	Multiple hospitals including Peking Union Medical College Hospital
**Objectives**	**Primary Objective**<br>To demonstrate that the efficacy of HRS-7535 in glycemic control is superior to placebo in adult subjects with type 2 diabetes mellitus inadequately controlled by diet and exercise alone.<br>**Secondary Objective**<br>In adult subjects with type 2 diabetes who have inadequate glycemic control with diet and exercise alone:<br>• To evaluate the efficacy of HRS-7535 in other aspects of glycemic control;<br>• To evaluate the efficacy of HRS-7535 in weight management;<br>• To evaluate the safety and tolerability of HRS-7535;<br>• To evaluate the pharmacokinetics (PK) of HRS-7535;<br>• To assess patient reported outcomes in each treatment group.

**Endpoints and Estimands**	**Primary Endpoint**<br>• Compared with the placebo group, the change in Glycated Hemoglobin (HbA1c) from baseline at week 32 of treatment in the HRS-7535 group.<br>**Primary Estimand**<br>• Treatment: HRS-7535 30 mg, 60 mg, 90 mg or placebo;<br>• Population: adult subjects with type 2 diabetes with inadequate glycemic control by diet and exercise alone<br>• Variable (Endpoint): change in HbA1c from baseline at week 32 of treatment;<br>• Intercurrent events and handling strategies:<br><table><tr><th>Intercurrent events</th><th>Management Strategy</th></tr><tr><td>Early discontinuation of study treatment</td><td>Treatment policy strategy: if a subject prematurely discontinued study treatment prior to the primary endpoint at Week 32, the subsequently collected data were included in the statistical analysis.</td></tr><tr><td>Start additional glucose-lowering agents*</td><td>Treatment policy strategy: if the subject starts using additional glucose-lowering agents before the primary endpoint at Week 32, the subsequently collected data will be included in the statistical analysis.</td></tr></table>*Additional glucose-lowering agents are defined as any glucose-lowering agents used for more than 14 days cumulatively.<br>• Summary at the population level: the mean difference between groups in the change of HbA1c from baseline after 32 weeks of treatment.<br>**Secondary endpoints:**<br>At week 32 of treatment, the HRS-7535 group showed the following compared with the placebo group:<br>• Proportion of subjects reaching HbA1c < 7.0%;<br>• Proportion of subjects reaching HbA1c ≤6.5%;<br>• Change from baseline in fasting plasma glucose (FPG);<br>• Percentage change in weight from baseline.<br>**Secondary endpoints:**<br><u>Efficacy endpoint</u>

	At week 32 of treatment, the HRS-7535 group showed the following compared with the placebo group:<br>• Change in weight and waist circumference from baseline;<br>• Proportion of subjects reaching HbA1c < 5.7%;<br>• Changes from baseline in fasting serum insulin, fasting serum C-peptide, homeostasis model assessment of β-cell function (HOMA-β), and insulin resistance index (HOMA-IR);<br>• Changes from baseline in 1-hour postprandial plasma glucose, 2-hour postprandial plasma glucose, 2-hour postprandial insulin, serum 2-hour postprandial C-peptide, and disposition index (DI);<br>• Changes from baseline in 7-point self-monitored blood glucose (SMBG) profile;<br>• Proportion of subjects requiring rescue treatment in each group<br>At week 52 of treatment, the HRS-7535 group showed the following compared with the placebo group:<br>• Change in HbA1c from baseline;<br>• Change in FPG relative to baseline;<br>• Percentage change in weight from baseline;<br>• Change in weight and waist circumference from baseline;<br>• Proportion of subjects reaching HbA1c < 7.0%;<br>• Proportion of subjects reaching HbA1c ≤6.5%;<br>• Proportion of subjects reaching HbA1c < 5.7%;<br>• Changes from baseline in fasting serum insulin, fasting serum C-peptide, homeostasis model assessment of β-cell function (HOMA-β), and insulin resistance index (HOMA-IR);<br>• Changes from baseline in 1-hour postprandial plasma glucose, 2-hour postprandial plasma glucose, 2-hour postprandial insulin, serum 2-hour postprandial C-peptide, and disposition index (DI);<br>• Changes from baseline in 7-point self-monitored blood glucose (SMBG) profile;<br>• Proportion of subjects requiring rescue treatment in each group;<br>Safety endpoints

	Week 32 and Week 52 of Treatment<br>• Adverse events (AEs), hypoglycemic events, laboratory tests, vital signs, physical examination, 12-lead electrocardiogram, diabetic retinopathy screening and evaluation, etc.<br><u>Pharmacokinetic Endpoints</u><br>• Plasma HRS-7535 concentration.<br><u>Patient-Reported Outcome endpoint</u><br>• Change from baseline to Week 32 and Week 52 in Diabetes Treatment Satisfaction Questionnaire status version (DTSQs).
**Trial Population**	Adult subjects with type 2 diabetes with inadequate glycemic control by diet and exercise alone.
**Study Design**	This trial is a multi-center, randomized, double-blind, placebo-controlled, parallel-group Phase III clinical trial, including a Screening Phase of up to 2 weeks, a 3-week run-in Phase, a 52-week double-blind treatment period, and a 2-week Safety follow-up Phase. The trial population consists of adult subjects with type 2 diabetes inadequately controlled by diet and exercise alone, with a planned enrollment of 272 subjects.<br>**Screening phase (W-5 ~ W-3, up to 2 weeks)**<br>After signing the Informed Consent Form, subjects will undergo screening assessments, and those who meet the inclusion/exclusion criteria may enter the run-in phase.<br>**Run-in period (W-3 ~ W0)**<br>Subjects who pass the screening will start continuous oral administration of single-blind run-in drug for 3 weeks at V2, D-21 (once daily [QD]).<br>Subjects undergo baseline assessment at V3 (D-4), and the assessment at V4 (D1) will be performed to assess whether the subject meets the randomization criteria. Subjects who met the randomization criteria entered the 52-week double-blind treatment period.<br>**Double-Blind Treatment Period (W1-W52)**

	Subjects are randomized in a 1:1:1:1 ratio to the HRS-7535 30 mg group, 60 mg group, 90 mg group, and placebo group, with stratification based on baseline HbA1c values (≤8.5%, >8.5%) measured by the central laboratory and prior use of glucose-lowering agents (yes, no). Double-blind treatment period medication was initiated after randomization. From week 33 of the double-blind treatment period to the end of this period, the placebo group switched to HRS-7535 30 mg treatment. All subjects are required to undergo assessments of efficacy and safety-related parameters at protocol-specified visit timepoints during the double-blind treatment period.<br>**Safety follow-up phase (W53 ~ W54)**<br>After discontinuation of the double-blind treatment period medication, subjects will complete the safety follow-up via telephone visit at Week 54.
**Dosing Regimen**	Run-in period (single-blind)<br>All subjects received HRS-7535 placebo (1 tablet each of 30 mg and 60 mg specifications), QD. The Investigational Product is allowed to be taken on the day of V2 or the next day, with the medication day as day 1 of the run-in phase.<br>Double-blind treatment period<br>Approximately 272 subjects will be randomized in a 1:1:1:1 ratio to HRS-7535 30 mg group, 60 mg group, 90 mg group, and placebo group, with stratification by baseline HbA1c value (≤8.5%, >8.5%) measured by central laboratory and prior use of glucose-lowering agents (yes, no). The double-blind treatment period medication will be initiated after randomization. The Investigational Product is allowed to be administered on the day of randomization or the next day, with the administration day considered as Day 1 of the double-blind treatment period.<br>The dosing regimens of each group are as follows:<br>• HRS-7535 30 mg group: HRS-7535 30 mg, administered orally, QD.

- The HRS-7535 60 mg group: dose titration is adopted. The subjects receive HRS-7535 30 mg, orally administered, QD for the first 4 weeks (D1 to D28); starting from Week 5 (after D29), they receive HRS-7535 60 mg, orally administered, QD.
- HRS-7535 90 mg group: dose titration is adopted. For the first 4 weeks (D1 to D28), HRS-7535 30 mg is administered orally, QD; from Week 5 to Week 8 (D29 to D56), HRS-7535 60 mg is administered orally, QD; starting from Week 9 (after D57), HRS-7535 90 mg is administered orally, QD.
- Placebo group: from Week 1 to Week 32 (D1 to D224), receive HRS-7535 Placebo (1 tablet each of 30 mg and 60 mg specifications), QD; starting from Week 33 (after D225), receive HRS-7535 30 mg, orally administered, QD.

To ensure the implementation of blinding, the drug dosage and corresponding number of tablets for each dose level during the run-in phase and double-blind treatment phase are as follows:

Dose Level / Investigational Product	Run-in period:	Double-blind treatment period			
	All Subjects	HRS-7535 30 mg	HRS-7535 60 mg	HRS-7535 90 mg	Placebo
HRS-7535 30 mg	0	1	0	1	0
HRS-7535 30 mg placebo	1	0	1	0	1
HRS-7535 60 mg	0	0	1	1	0
HRS-7535 60 mg placebo	1	1	0	0	1

Inclusion Criteria

Subjects must meet all the following criteria to be enrolled in this trial:

1. Male or female, aged between 18 and 75 years (inclusive) on the day of signing the ICF;
2. Diagnosed with type 2 diabetes mellitus according to the Chinese Guidelines for the Prevention and Treatment of Type 2 Diabetes (2020 Edition), with a disease duration of ≥2 months at screening;
3. Blood glucose was managed through consistent dietary control and exercise within 2 months prior to screening, without the use of any glucose-lowering agents;

	4. HbA1c levels between 7.0% and 10.0% (inclusive) at screening (as tested by a local laboratory);<br>5. Body mass index (BMI) of 19.0 – 40.0 kg/m^2 (inclusive) at screening;<br>6. Having voluntarily signed the ICF prior to trial initiation, subjects must fully understand the study content, procedures, and possible adverse reactions, and must be able and willing to complete the study in accordance with protocol requirements (e.g., self-monitoring of blood glucose and maintaining subject diaries);<br>7. From signing the ICF until 2 weeks after the last dose, subjects have no plans for pregnancy and are willing to use highly effective contraception as specified in the protocol.
**Exclusion criteria**	Subjects who meet any of the following criteria shall not be enrolled in this trial:<br>**Those with the following abnormalities at screening or randomization:**<br>1. Presence of the following laboratory test abnormalities at screening or randomization:<br>• Fasting venous glucose > 15 mmol/L;<br>• Alanine aminotransferase (ALT) or aspartate aminotransferase (AST) > 2 × upper limit of normal (ULN);<br>• Total bilirubin (TBIL) > 1.5 × ULN;<br>• Amylase and/or lipase > 3 × ULN;<br>• Fasting triglycerides (TG) > 11.3 mmol/L;<br>• Calcitonin ≥50 ng/L;<br>• Estimated glomerular filtration rate (eGFR) < 30 mL/min/1.73 m^2 (calculated according to the chronic kidney disease epidemiology collaboration [CKD-EPI] formula);<br>• Urinary albumin-to-creatinine ratio (UACR) ≥1000 mg/g;<br>• Positive Hepatitis B Virus Surface Antigen (HBsAg) and HBV DNA > ULN;

	2. Clinically significant abnormalities in ECG results at screening or randomization that may affect subject safety, including but not limited to supraventricular tachycardia, atrial fibrillation, atrial flutter, second- or third-degree atrioventricular block, etc.;<br>3. The electrocardiogram examination results during screening or randomization indicated a heart rate > 100 bpm or QTcF > 480 ms. If the heart rate or QTcF is abnormal, two additional electrocardiograms may be performed to obtain the mean of three values;<br>4. Uncontrolled severe hypertension (systolic blood pressure ≥160 mmHg or diastolic blood pressure ≥100 mmHg) at screening or randomization;<br>**Those with the following diseases or medical history prior to screening:**<br>5. Diagnosed or suspected type 1 diabetes mellitus, special type of diabetes mellitus or secondary diabetes mellitus;<br>6. Acute complications of diabetes (diabetic ketoacidosis, lactic acidosis, hyperglycemic hyperosmolar state, etc.) within 6 months prior to screening;<br>7. Severe hypoglycemic episodes or recurrent hypoglycemic episodes within 6 months prior to screening (≥ 3 hypoglycemic episodes within a week, or other hypoglycemic episodes as determined by the investigator);<br>8. Proliferative retinopathy or maculopathy, painful diabetic neuropathy, diabetic foot ulcer, or intermittent claudication requiring acute treatment at screening;<br>9. Within 6 months prior to screening, subjects with known clinically significant diseases including but not limited to neurological, psychiatric, cardiovascular, endocrine, gastrointestinal, respiratory, urinary, hematological, immune system and other diseases (except for type 2 diabetes related conditions) that may interfere with the study results or pose additional risks to the administration of the study drug as determined by the investigator;

	10. Past or known history of medullary thyroid carcinoma (MTC) or multiple endocrine neoplasia syndrome type 2 (MEN2), or family history thereof;<br>11. Past or known medical history of acute or chronic pancreatitis, pancreatic injury, acute cholecystitis, or symptomatic/treatment-requiring gallbladder disease (excluding subjects who have undergone cholecystectomy and are determined by the investigator to be eligible for enrollment);<br>12. Past history of significant gastrointestinal diseases (such as gastric outlet obstruction, inflammatory bowel disease, active ulcer, etc.), or those who have undergone gastrointestinal surgery (except for gastrointestinal polypectomy and appendectomy), or those with a history of long-term use of medications that directly affect gastrointestinal motility;<br>13. History of hyperthyroidism prior to screening, or hyperthyroidism at screening, or other thyroid disorders not adequately controlled (except for subjects with thyroid hormone replacement treatment regimen stable for at least 8 weeks and no plan to change the treatment regimen during the study);<br>14. History of severe cardiovascular and cerebrovascular diseases within 6 months prior to screening, including but not limited to: congestive heart failure (New York Heart Association [NYHA] Class III–IV), unstable angina, stroke, myocardial infarction, severe arrhythmia, or coronary revascularization; or planned to undergo coronary, carotid or peripheral artery revascularization at screening;<br>15. Subjects with known or suspected depression, bipolar disorder, suicidal tendency, schizophrenia or other serious mental diseases; or those with cognitive impairment or language barriers who are unable to fully understand the protocol or unwilling to cooperate with the study site staff;<br>16. History of malignant neoplasm within 5 years prior to screening, except for cured local cancer, such as: localized cutaneous basal

cell carcinoma, cervical carcinoma in situ, and carcinoma in situ of the prostate;

Those who have received any of the following drugs or treatments prior to screening:

17. Use of glucagon-like peptide-1 (GLP-1) receptor agonists (including multi-target GLP-1 receptor agonists) within 3 months before screening;
18. Use of systemic glucocorticoids (excluding those with cumulative use of <14 days or administration via topical, inhaled, intraocular, or intranasal routes) within 3 months prior to the screening;
19. Use of tricyclic antidepressants, atypical antipsychotics, and mood stabilizers (such as imipramine, chlorpromazine, amitriptyline, mirtazapine, clozapine, olanzapine, paroxetine, phenelzine, thioridazine, valproic acid and its derivatives, lithium salts, etc.) within 3 months before screening;
20. Weight loss therapy within 3 months prior to screening, including intensive diet or exercise therapy for weight loss, pharmacological therapy, surgical treatment (except for liposuction history more than 1 year), dietary supplements or other special therapies;

Those with any of the following conditions:

21. At screening or randomization, self-reported weight change exceeding ±5% within the previous 3 months, or planned intensive diet or exercise therapy for weight loss during the trial (except for diabetes diet and exercise control measures);
22. Those who have participated in any clinical trial of drugs or medical devices within 3 months prior to screening or still within 5 half-lives of the study drug (whichever is longer). Participation in a clinical trial is defined as signing informed consent, and having used the Investigational Product (including placebo) or investigational medical device;
23. Known or suspected hypersensitivity to the study drug or its components;

	24. Known or suspected history of drug or alcohol abuse, and judged by the investigator as unsuitable for participation in this clinical trial;<br>25. Subjects with a history of blood donation or blood loss of ≥ 400 mL within 3 months prior to screening, or who have received blood transfusion;<br>26. Female subjects who are pregnant, breastfeeding, or planning to become pregnant during the trial;<br>27. Intolerance to venipuncture or needle phobia;<br>28. Any condition that, in the investigator's judgment, makes the subject unsuitable for participation in this clinical trial.
**Randomization Criteria**	Based on the V3 assessment results, the subject must be reconfirmed to meet the following randomization criteria before randomization. For subjects who failed randomization in this study, there is no eligibility for rescreening.<br>1. HbA1c levels between 7.0% and 10.0% (inclusive) (as tested by a central laboratory);<br>2. Glucose under fasting conditions ≤15.0 mmol/L (central laboratory results);<br>3. Medication compliance ≥ 80% and ≤ 120% during the run-in phase;<br>4. Laboratory tests, vital signs, body weight, BMI, electrocardiogram, pregnancy status, etc. met the inclusion criteria and did not meet the exclusion criteria;<br>5. No glucose-lowering agents were used;<br>6. No condition considered by the investigator to be inappropriate for participation in this clinical trial occurred during the run-in phase.
**Efficacy evaluation**	Assessment of Efficacy measures include HbA1c, FPG, 1-hour postprandial plasma glucose, 2-hour postprandial plasma glucose, fasting serum insulin and C-peptide, 2-hour postprandial serum insulin and C-peptide, HOMA-β, HOMA-IR, disposition index, 7-point SMBG profile, body weight, and waist circumference.

**PK Evaluation**	PK blood samples will be collected from the subject at V4, V6, V7, V8, V9, V10, V11, V12, and V15, and plasma HRS-7535 concentrations will be measured.
**Safety evaluation**	Safety evaluation include collection of AEs, hypoglycemic episodes, vital signs and physical examination, 12-lead ECG, laboratory assessment, diabetic retinopathy screening and assessment.
**Sample size determination**	The sample size calculation of this study is based on the primary endpoint, i.e., the change in HbA1c from baseline at Week 32 of treatment. Based on the results of the Phase II study, assuming that the efficacy difference in HbA1c between each dosage group of HRS-7535 and the placebo group is -0.85% for subjects without intercurrent events, and 0 for subjects with intercurrent events, the overall efficacy would be -0.72% (considering an intercurrent events incidence of 15%), with a standard deviation (SD) = 1.1%. A total of 272 subjects (68 per group) will be randomly assigned in a 1:1:1:1 ratio to the HRS-7535 30 mg group, 60 mg group, 90 mg group, and placebo group, providing an overall power of 90% to detect the superiority of all three dosage groups of HRS-7535 over the placebo group at a one-sided significance level of 0.025.
**Statistical Analysis**	**Definition of Analysis Set**<br>Intention-to-treat (ITT) analysis set: includes all randomized subjects. Subjects will be analyzed according to the group assigned at randomization, and ITT is the primary analysis population for efficacy.<br>Safety set (SS): includes all subjects who received at least one dose of the Investigational Product after randomization. Subjects will be analyzed according to the group they are randomly assigned to.<br>PK concentration analysis set (PKCS): includes all subjects who have been randomized to receive HRS-7535 treatment and have at least one evaluable PK concentration data post-dose.<br>**Analysis of primary efficacy endpoints**

	For the primary estimand, the primary study endpoint will be analyzed based on the ITT set. HbA1c will be analyzed using analysis of covariance (ANCOVA) model with treatment, stratification factors (prior use of glucose-lowering agents [yes, no]) as fixed effects, and baseline HbA1c as covariate. The least squares mean, standard error, 95% confidence interval (CI) of the change from baseline in HbA1c at week 32 for each group and between groups, as well as the p-value for the difference between each dosage group of HRS-7535 and the placebo group will be estimated and listed.<br>A treatment-policy strategy is applied for all intercurrent events, data after intercurrent events will be included in the analysis. Missing data due to intercurrent events will be imputed using the RDO (Retrieved Dropout) method. If the data do not support the RDO method, the CR (Copy Reference) method will be used for imputation. Missing data unrelated to Intercurrent events are assumed to be missing at random.<br>A sensitivity analysis will be performed on the primary estimand. See the Statistical Analysis Plan (SAP) for the detailed method.<br>**Analysis of secondary efficacy endpoints**<br>The secondary efficacy endpoints will be analyzed based on the ITT.<br>The generalized estimating equation (GEE) will be used to analyze the binary endpoint (response/no response) at week 32 of treatment.<br>Continuous endpoints at week 32 of treatment will be analyzed using the mixed-effects model for repeated measures (MMRM) method.<br>The secondary efficacy endpoints at Week 52 will be analyzed descriptively.<br>**Analysis of other secondary endpoints**<br>The change from baseline in DTSQs at week 32 will be analyzed using ANCOVA method based on the ITT. The change from baseline in DTSQs at week 52 will be summarized descriptively.<br>**Safety Analysis**<br>All safety analyses will be based on the SS.

	Descriptive statistics will be provided for safety endpoints, which include AEs, hypoglycemia events, laboratory assessment, vital signs, physical examination, 12-lead electrocardiogram, and diabetic retinopathy screening and assessment.<br>**Pharmacokinetic Endpoint Analysis**<br>The plasma concentrations of HRS-7535 will be summarized using descriptive statistics (if applicable).

1.2. Schematic Diagram of Trial

The schematic diagram of this trial is shown in Figure 1:Figure 1. Overview of study design1

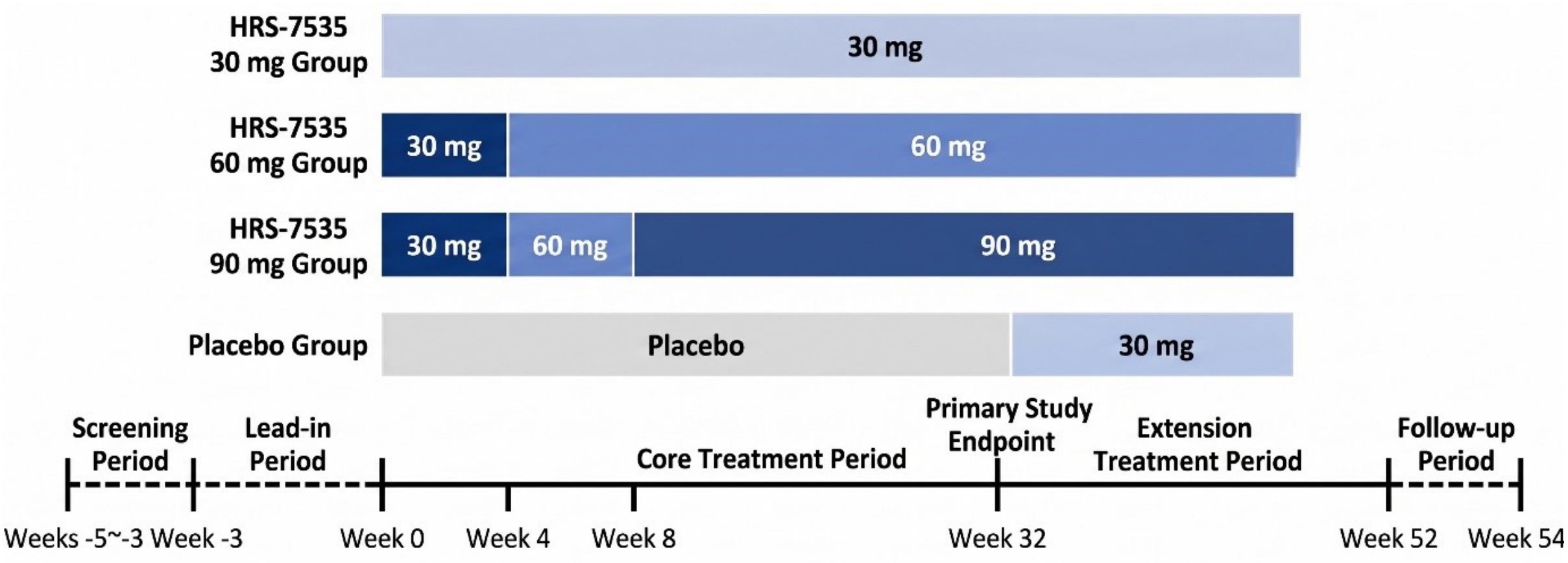

Figure 1. Overview of study design1

1.3. Schedule of Activities

Table 1. Study Flowchart

In order to protect the health of subjects, the investigator may arrange unscheduled visits in addition to the visits in the table below.

Table 1. Study Flowchart

Item	Screening Period[1]	Run-in period:		Treatment Period													Safety Follow-up
**Visit**	**V1**	**V2**	**V3**	**V4**	**V5[14]**	**V6**	**V7**	**V8**	**V9**	**V10**	**V11**	**V12**	**V13**	**V14**	**V15**	**Early Withdrawal[15]**	**V16**
**Visit Time (weeks)**	**W-5 ~ W-3**	**W-3**	**W-1 ~ W0**	**W1**	**W2**	**W4**	**W8**	**W12**	**W16**	**W20**	**W26**	**W32**	**W38**	**W44**	**W52**	**/**	**W54/ 14 days after withdrawal**
**Visit Time (days)**	**D-35 ~ D-22**	**D-21**	**D-4**	**D1**	**D15**	**D29**	**D57**	**D85**	**D113**	**D141**	**D183**	**D225**	**D267**	**D309**	**D364**	**/**	
**Time Window (days)**	**/**	**/**	**-3**	**0**	**±3**	**±3**	**±3**	**±3**	**±3**	**±3**	**±5**	**±5**	**±5**	**±5**	**±5**	**/**	**±3**
**Visit under fasting conditions**	X	X	X	X	☎[14]	X	X	X	X	X	X	**X**	**X**	**X**	**X**	X	☎[14]
**Subject information and clinical evaluation**																	
Signing of ICF	X																
Assignment of screening number	X																
Inclusion and exclusion criteria	X																
Randomization Criteria[2]				X													
Assigning of randomization number				X													

Item	Screening Period[1]	Run-in period:		Treatment Period													Safety Follow-up
**Visit**	**V1**	**V2**	**V3**	**V4**	**V5[14]**	**V6**	**V7**	**V8**	**V9**	**V10**	**V11**	**V12**	**V13**	**V14**	**V15**	**Early Withdrawal[15]**	**V16**
**Visit Time (weeks)**	**W-5 ~ W-3**	**W-3**	**W-1 ~ W0**	**W1**	**W2**	**W4**	**W8**	**W12**	**W16**	**W20**	**W26**	**W32**	**W38**	**W44**	**W52**	**/**	**W54/ 14 days after withdrawal**
**Visit Time (days)**	**D-35 ~ D-22**	**D-21**	**D-4**	**D1**	**D15**	**D29**	**D57**	**D85**	**D113**	**D141**	**D183**	**D225**	**D267**	**D309**	**D364**	**/**	
**Time Window (days)**	**/**	**/**	**-3**	**0**	**±3**	**±3**	**±3**	**±3**	**±3**	**±3**	**±5**	**±5**	**±5**	**±5**	**±5**	**/**	**±3**
Demographic data	X																
Past medical history	X																
Prior and Concomitant Treatment	X	X	X	X	X	X	X	X	X	X	X	X	X	X	X	X	X
History of tobacco and alcohol use	X																
Body height	X																
BMI	X		X														
Body weight and waist circumference under fasting conditions	X		X			X	X	X	X	X	X	X	X	X	X	X	
**Safety evaluation**																	
Adverse event	X	X	X	X	X	X	X	X	X	X	X	X	X	X	X	X	X
Hypoglycemic episodes		X	X	X	X	X	X	X	X	X	X	X	X	X	X	X	X

**Item**	**Screening Period**[1]	**Run-in period:**		**Treatment Period**													**Safety Follow-up**
**Visit**	**V1**	**V2**	**V3**	**V4**	**V5**[14]	**V6**	**V7**	**V8**	**V9**	**V10**	**V11**	**V12**	**V13**	**V14**	**V15**	**Early Withdrawal**[15]	**V16**
**Visit Time (weeks)**	**W-5 ~ W-3**	**W-3**	**W-1 ~ W0**	**W1**	**W2**	**W4**	**W8**	**W12**	**W16**	**W20**	**W26**	**W32**	**W38**	**W44**	**W52**	**/**	**W54/ 14 days after withdrawal**
**Visit Time (days)**	**D-35 ~ D-22**	**D-21**	**D-4**	**D1**	**D15**	**D29**	**D57**	**D85**	**D113**	**D141**	**D183**	**D225**	**D267**	**D309**	**D364**	**/**	
**Time Window (days)**	**/**	**/**	**-3**	**0**	**±3**	**±3**	**±3**	**±3**	**±3**	**±3**	**±5**	**±5**	**±5**	**±5**	**±5**	**/**	**±3**
Vital Signs [3]	X		X			X	X	X	X	X	X	X	X	X	X	X	
Physical Examination [4]	X		X									X			X	X	
Routine Blood Test	X		X			X		X		X		X			X	X	
Routine Urine Test	X		X			X		X		X		X			X	X	
UACR	X		▲			▲	▲		▲		▲	▲			▲	▲	
Blood biochemistry	X		X			X	X	X	X	X	X	X	X	X	X	X	
Blood lipids	X		X				X			X		X			X	X	
Serum amylase	X		X				X			X		X			X	X	
Serum lipase [5]	X		X				X			X		X			X	X	
Coagulation test	X																
Thyroid Function Test	X																
Serum calcitonin 5	X						X					X			X	X	

Item	Screening Period[1]	Run-in period:		Treatment Period													Safety Follow-up
**Visit**	**V1**	**V2**	**V3**	**V4**	**V5[14]**	**V6**	**V7**	**V8**	**V9**	**V10**	**V11**	**V12**	**V13**	**V14**	**V15**	**Early Withdrawal[15]**	**V16**
**Visit Time (weeks)**	**W-5 ~ W-3**	**W-3**	**W-1 ~ W0**	**W1**	**W2**	**W4**	**W8**	**W12**	**W16**	**W20**	**W26**	**W32**	**W38**	**W44**	**W52**	**/**	**W54/ 14 days after withdrawal**
**Visit Time (days)**	**D-35 ~ D-22**	**D-21**	**D-4**	**D1**	**D15**	**D29**	**D57**	**D85**	**D113**	**D141**	**D183**	**D225**	**D267**	**D309**	**D364**	**/**	
**Time Window (days)**	**/**	**/**	**-3**	**0**	**±3**	**±3**	**±3**	**±3**	**±3**	**±3**	**±5**	**±5**	**±5**	**±5**	**±5**	**/**	**±3**
12-lead electrocardiogram [6]	X		X				X			X		X			X	X	
Pregnancy test[7]	X		X				X			X		X			X	X	
Follicle-stimulating hormone (menopausal women only)[7]	X																
Hepatitis B Test	X																
Screening and assessment of diabetic retinopathy [8]	X											X			X	X	
**EFFICACY EVALUATION**																	
Glycated Hemoglobin 9	X		▲			▲	▲	▲	▲	▲	▲	▲	▲	▲	▲	▲	
Fasting plasma glucose[9]	X		▲			▲	▲	▲	▲	▲	▲	▲	▲	▲	▲	▲	
Fasting insulin, C-peptide			▲						▲			▲			▲	▲	
MMTT[10]			▲									▲			▲	▲	

Item	Screening Period[1]	Run-in period:		Treatment Period													Safety Follow-up
**Visit**	**V1**	**V2**	**V3**	**V4**	**V5[14]**	**V6**	**V7**	**V8**	**V9**	**V10**	**V11**	**V12**	**V13**	**V14**	**V15**	**Early Withdrawal[15]**	**V16**
**Visit Time (weeks)**	**W-5 ~ W-3**	**W-3**	**W-1 ~ W0**	**W1**	**W2**	**W4**	**W8**	**W12**	**W16**	**W20**	**W26**	**W32**	**W38**	**W44**	**W52**	**/**	**W54/ 14 days after withdrawal**
**Visit Time (days)**	**D-35 ~ D-22**	**D-21**	**D-4**	**D1**	**D15**	**D29**	**D57**	**D85**	**D113**	**D141**	**D183**	**D225**	**D267**	**D309**	**D364**	**/**	
**Time Window (days)**	**/**	**/**	**-3**	**0**	**±3**	**±3**	**±3**	**±3**	**±3**	**±3**	**±5**	**±5**	**±5**	**±5**	**±5**	**/**	**±3**
Pre-breakfast SMBG[11]		X	→	→	→	→	→	→	→	→	→	→	→	→	X		
7-point SMBG[12]			X						X			X			X	X	
DTSQs	X		X									X			X	X	
PK Blood Collection[13]				▲		▲	▲	▲	▲	▲	▲	▲			▲	▲	
**Subject Guidance**																	
Diet and lifestyle education	X	X	X	X	X	X	X	X	X	X	X	X	X	X	X	X	X
Contraceptive guidance	X	X	X	X	X	X	X	X	X	X	X	X	X	X	X	X	
Glucose meter instructions		X	X	X	X	X	X	X	X	X	X	X	X	X			
Subject diary usage guidance		X	X	X	X	X	X	X	X	X	X	X	X	X			
Dispensation of Investigational Product		☆		★		★	★	★	★	★	★	★	★	★			

Item	Screening Period[1]	Run-in period:		Treatment Period													Safety Follow-up
**Visit**	**V1**	**V2**	**V3**	**V4**	**V5[14]**	**V6**	**V7**	**V8**	**V9**	**V10**	**V11**	**V12**	**V13**	**V14**	**V15**	**Early Withdrawal[15]**	**V16**
**Visit Time (weeks)**	**W-5 ~ W-3**	**W-3**	**W-1 ~ W0**	**W1**	**W2**	**W4**	**W8**	**W12**	**W16**	**W20**	**W26**	**W32**	**W38**	**W44**	**W52**	**/**	**W54/ 14 days after withdrawal**
**Visit Time (days)**	**D-35 ~ D-22**	**D-21**	**D-4**	**D1**	**D15**	**D29**	**D57**	**D85**	**D113**	**D141**	**D183**	**D225**	**D267**	**D309**	**D364**	**/**	
**Time Window (days)**	**/**	**/**	**-3**	**0**	**±3**	**±3**	**±3**	**±3**	**±3**	**±3**	**±5**	**±5**	**±5**	**±5**	**±5**	**/**	**±3**
Accountability of returned Investigational Product				☆		★	★	★	★	★	★	★	★	★	★	★	
Compliance assessment			X	X	X	X	X	X	X	X	X	X	X	X	X	X	
Telephone inquiry [16]						X	X										

Abbreviations: ☎=telephone visit; ☆=Phase induction drug; ★=double-blind period drug; ▲=central laboratory test; BMI=body mass index; DTSQs=Diabetes Treatment Satisfaction Questionnaire current version; PK=Pharmacokinetics; SMBG=self-monitoring of blood glucose; UACR=Albumin_U/Creatinine ratio; MMTT=mixed meal tolerance test; →=ongoing.

1. Screening phase: subjects may proceed with the screening process only after signing the Informed Consent Form. If an assessment during the screening Phase is abnormal and there is a valid reason for retesting, it may be retested once within 1 week (HbA1c may only be retested in cases of objective circumstances such as sample collection errors, sample contamination, or unqualified samples). Subjects who experience screen failure may undergo re-evaluation for screening after 1 month if there is sufficient justification. During re-screening, a new screening number should be assigned, and the Informed Consent Form should be re-signed. Only one re-screening is allowed.
2. Randomization criteria: the complete run-in period is from D-21 to D-1, and the baseline assessment should be completed at V3 (D-4). Retesting of the baseline V3 assessment is not allowed (except for objective circumstances such as sample collection error, sample contamination, or unqualified samples). Subjects who pass the screening but do not continue to participate in the trial, fail to meet the randomization criteria, or meet the randomization criteria but do not participate in the randomization are considered randomization failures. Subjects who fail randomization are not eligible for re-screening.
3. Vital signs: including systolic blood pressure, diastolic blood pressure, pulse, etc.

4. Physical examination: including general condition, skin, lymph nodes, head and neck, chest (including heart and lungs), abdomen, the musculoskeletal system, and nervous system.

5. Lipase, calcitonin: if the local laboratory cannot test for lipase and calcitonin, they should be tested at the central laboratory.

6. 12-lead ECG: including heart rate, PR, RR, QT, and QTcF. Subjects are monitored after at least 10 min of rest in sitting position; if cardiac AEs occur during the study, ECG frequency may be increased at the discretion of the investigator. For details, see Section 8.3.3 of the protocol.8.3.3

7. Pregnancy test: for female subjects of childbearing potential, serum pregnancy tests will be performed at V1, V3, V12, and V15. For subsequent visits, urine or serum pregnancy tests may be conducted if the subject is not in the menstrual phase. The investigator may perform additional pregnancy tests during the study based on the subject's actual condition. Postmenopausal women: for those > 45 years and ≤65 years old, both Follicle Stimulating Hormone (FSH) and serum pregnancy tests are required during the screening phase. The decision on pregnancy testing during subsequent visits will be based on the FSH results. For those > 65 years old, FSH and pregnancy tests are not required. Detailed regulations are provided in Section 13.1.3.13.1.3

8. Diabetic retinopathy screening and assessment: all subjects will undergo fundus photography at Week 32 (±2 weeks) and Week 52 Visit (-4 weeks).

9. HbA1c, fasting plasma glucose: HbA1c and fasting plasma glucose are tested at the local laboratory during screening and at the central laboratory at other times. If some sites cannot test plasma glucose during screening phase, serum glucose results are acceptable.

10. MMTT: under fasting conditions in the morning, venous blood samples will be collected at 0.5 h ± 5 min, 1 h ± 5 min, and 2 h ± 5 min (timing started from the first bite) after mixed meal consumption for the test of plasma glucose, serum insulin, and serum C-peptide. Fasting status should be maintained until the 2 h postprandial blood collection, as detailed in Section 8.2.4.

11. Pre-breakfast SMBG: it is recommended to measure fasting fingertip blood glucose at least once weekly between the visit. If hypoglycemia is suspected, fingertip blood glucose should be tested promptly.

12. 7-point SMBG: conduct continuous 7-point SMBG monitoring on any one day within 3 days before the visit (starting from pre-breakfast on that day and completing 7-point blood glucose collection within 24 hours), including measurements before (recommended within 30 minutes before meals) and after (120 ± 10 minutes after the start of each meal) breakfast, lunch, and dinner, as well as bedtime measurement. If one complete 7-point SMBG test is not completed, a second 7-point SMBG monitoring should be performed on a different day.

13. PK sampling: PK blood collection is performed at the scheduled visits, and the time of the last dose before blood collection should be accurately recorded. If a subject withdraws early, a PK blood sample should also be collected at the time of withdrawal (except for those whose withdrawal visit is more than 48 hours after the last dose).

14. Telephone visit: V5 and V16 are telephone visits, and the investigator may change them to site visits if necessary.

15. Early withdrawal: randomized subjects who withdraw early from the study should complete the early withdrawal visit as soon as possible. If the early withdrawal visit occurs ≤ 1 week from the last visit (except for V4), only a blood pregnancy test for females is required without repeating other safety assessments, unless deemed necessary by the investigator.

16. Telephone inquiry: telephone inquiries will be conducted 1~2 weeks after the V6 and V7 visits to inquire about the general condition of the subject and provide medication guidance.

2. Introduction

2.1. Study Background

2.1.1. Disease Background

Diabetes is a group of metabolic diseases characterized by chronic hyperglycemia caused by multiple etiologies. Long-term hyperglycemia can cause multi-system damage, leading to chronic, progressive damage, functional decline, and failure of organs and tissues, including the eyes, kidneys, nerves, heart, and blood vessels. According to the IDF Diabetes Atlas (10th edition) released by the International Diabetes Federation in 2021, there were already 537 million diabetic patients worldwide in 2021 (prevalence: 10.5%). China ranked first with about 140.9 million diabetic patients. Diabetes and its complications impose a significant economic burden on healthcare systems worldwide. Type 2 diabetes accounts for more than 90% of all diabetes cases.[12]

There is still a lack of etiological treatment for type 2 diabetes, and comprehensive management models are mainly adopted, including dietary control, reasonable exercise, blood glucose monitoring, diabetes education, and the use of glucose-lowering agents. The glucose-lowering agents currently marketed in China mainly include biguanides, sulfonylureas, glinides, α-glucosidase inhibitors, thiazolidinediones, dipeptidyl peptidase-4 (DPP-4) inhibitors, sodium-glucose cotransporter-2 (SGLT2) inhibitors, glucagon-like peptide-1 (GLP-1) receptor agonists, and insulin. [3,4] Although there are numerous therapeutic drugs, the treatment rate and control rate of diabetes in China still need improvement. The 2013 epidemiological survey results showed rates of 32.2% and 49.2%, respectively.[3]

2.1.2. HRS-7535

GLP-1 receptor agonists are novel glucose-lowering drugs that have been widely used in clinical practice in recent years. GLP-1 receptor agonists can increase glucose-dependent insulin secretion, inhibit glucagon secretion, increase glucose uptake in muscle tissue, reduce hepatic glucose output, augment the incretin effect, and improve neurotransmitter dysfunction. Their mechanisms of action cover six of the " ominous octet" in the pathogenesis of diabetes.[5]

HRS-7535 is a small molecule oral GLP-1 receptor agonist independently developed by our company, intended for development in the treatment of type 2 diabetes and weight loss. This study plans to evaluate the glucose-lowering efficacy and safety of HRS-7535 compared with Placebo in adults with type 2 diabetes who have inadequate glycemic control with diet and exercise alone.

2.1.3. Non-Clinical Experience

Preclinical toxicology studies of HRS-7535 included dose range-finding studies in rats and cynomolgus monkeys, 4-week and 26-week repeated dose toxicity study in rats and 4-week and 39-week repeated dose toxicity study in cynomolgus monkeys, fertility and early embryonic developmental toxicity study in rats, embryo-fetal developmental toxicity study in transgenic mice and rabbits expressing human GLP-1 receptor, and genotoxicity study.

In the dose range determination test of HRS-7535 with 7-day repeated oral administration at 30, 100 and 300 mg/kg in rats, the Maximum Tolerated Dose (MTD) was 300 mg/kg, and the No Observed Adverse Effect Level (NOAEL) was 100 mg/kg. The primary toxicities were decreased reticulocyte count and increased platelet count. In the 2-week repeated nasogastric administration of 5, 100 and 300 mg/kg HRS-7535 dose range determination test in cynomolgus monkeys, animal deaths were observed in the 300 mg/kg group, with primary toxicities observed in the kidneys and gastrointestinal tract. The MTD was 100 mg/kg and the NOAEL was 5 mg/kg.

The NOAEL for the 4-week repeated dose toxicity study of HRS-7535 in SD rats at doses of 10, 50 and 250 mg/kg was 50 mg/kg; the main toxicity in the 250 mg/kg group was manifested as increased blood phosphorus in animals at the end of administration (mean change level: female: ↑22.7%; male: ↑14.2%). The NOAEL for the 26-week repeated dose toxicity study of HRS-7535 in SD rats at doses of 15, 50 and 150 mg/kg/day was 150 mg/kg/day.

The NOAEL for the 4-week repeated administration of 2, 8 and 32 mg/kg HRS-7535 toxicity study in cynomolgus monkeys was 8 mg/kg; the main toxicity manifested as increased WBC and LYMP in male animals during the recovery period (mean change level: WBC: ↑263.3%; LYMP: ↑444.5%), and decreased RET# and RET% were observed in females (mean change level: RET%: ↓52.6%; RET#: ↓47.6%). The NOAEL for the 39-week repeated administration of 2, 10 and 50 mg/kg HRS-7535 toxicity study in cynomolgus monkeys was 10 mg/kg, the main toxicity manifested as sustained decrease of LDH in high-dose (50 mg/kg) group of both genders from D61 (mean change level: ↓49%-63%), without recovery during the recovery period.

HRS-7535 is a small molecule agonist of the GLP-1 receptor, which promotes pancreatic insulin secretion, reduces glucagon secretion, inhibits gastric emptying, and affects the central nervous system to enhance satiety and suppress appetite, thereby directly reducing energy intake. Cynomolgus monkeys, as a non-rodent toxicological species relevant to pharmacological effects, showed a decrease in the hematological parameter LDH only in the high-Dosage group (50 mg/kg) after 39 weeks of Repeated administration. Typically, a

decrease in LDH may be observed in conditions such as malnutrition or chronic wasting disease. No histopathological changes were observed in the liver or related tissues, suggesting that the decrease was mediated by the activation of the GLP-1 receptor, leading to reduced food intake and body weight. The exposure ratio of this dosage at the last administration was 4 times that of the clinical daily oral dose of 120 mg, indicating a low clinical risk.

In the genotoxicity studies of HRS-7535, the Ames test result was negative, indicating a low risk of DNA point mutation for HRS-7535. In addition, the results of chromosomal aberration test and rat bone marrow micronucleus test were both negative. Therefore, the genotoxicity risk of HRS-7535 in humans is expected to be low.

In the fertility and early embryonic development toxicity study in SD rats, the NOAEL for HRS-7535 in parental male and female animals was 75 mg/kg. In the 150 mg/kg dose group, decreased/average weight gain was observed in male animals, and decreased absolute body weight during pregnancy was observed in female animals. The NOAEL for HRS-7535 on fertility and early embryonic development in SD rats was 150 mg/kg. In the embryo-fetal developmental toxicity study in transgenic mice expressing human GLP-1 receptors (pharmacologically relevant rodents), the NOAEL for maternal toxicity of HRS-7535 was 60 mg/kg, and the NOAEL for embryo-fetal development was 20 mg/kg (based on exposure, 0.7 times the exposure ratio of the clinical daily oral dose of 120 mg). In 6 high-dose (180 mg/kg) pregnant animals, test article-related histopathological changes were observed, including increased thymic cortex-to-medulla ratio, lymphocyte depletion predominantly in the cortex, focal erosion/ulcer/inflammation in the non-glandular stomach, focal squamous epithelial hyperplasia, and increased extramedullary hematopoiesis in the spleen. Additionally, individual animals in the high-dose group showed emaciation and reduced activity after administration. Three animals had complete resorption of all implantation sites, with a high post-implantation loss rate. In the dose groups ≥60 mg/kg, the average weight and length of fetuses per litter were significantly reduced, the incidence of skeletal abnormalities was increased, and the number of ossification sites was significantly reduced. In the embryo-fetal developmental toxicity study in rabbits (non-pharmacologically relevant non-rodents), the NOAEL for parental pregnant rabbits was 150 mg/kg (based on exposure, 0.8 times the exposure ratio of the clinical daily oral dose of 120 mg), and the NOAEL for embryo-fetal development was the high dose of 300 mg/kg.

It is speculated that the toxicity to embryo-fetal development in transgenic mice expressing human GLP-1 receptors exposed to HRS-7535 at doses below the maximum recommended human dose (MRHD) may be secondary to maternal effects mediated by GLP-1 receptor

activation and impaired inverted yolk sac placenta function in rodents (in primates, the yolk sac does not invert to form a placenta). Food restriction has been confirmed to lead to increased pregnancy loss and fetal abnormalities, which is consistent with the toxicity profile of marketed GLP-1 analogs. The above results suggest that subjects in the HRS-7535 clinical study should use strict contraceptive measures.

The nonclinical study results of HRS-7535 are detailed in the Investigator's Brochure (IB) of HRS-7535.

2.1.4. Clinical Experience

As of May 2024, seven clinical studies of HRS-7535 have been conducted, namely HRS-7535-101 study, HRS-7535-102 study, HRS-7535-103 study, HRS-7535-201 study, HRS-7535-202 study, HRS-7535-203 study, and HRS-7535-205 study. The HRS-7535-101 study and HRS-7535-201 study have been completed, and the remaining studies are ongoing.

HRS-7535-101 is a randomized, double-blind, dose-escalation, placebo-controlled Phase I study in healthy subjects. Pharmacokinetics (PK) results showed that: at doses of 15 mg to 120 mg, the Tmax of oral HRS-7535 was approximately 6 hours under fasting conditions and about 12 h postprandially; the terminal elimination half-life was 5.28 to 9.08 h; exposure increased correspondingly with dose escalation, exhibiting approximately linear PK characteristics; after multiple oral administrations of HRS-7535, steady state was achieved in approximately 3 days, with mild accumulation of systemic exposure compared to single-dose administration; food intake slowed the time to peak absorption (Tmax) of HRS-7535, reduced peak concentration (Cmax), and slightly decreased the area under the curve (AUC), presenting PK characteristics similar to sustained-release formulations, which may not significantly affect the relative bioavailability of HRS-7535. In the HRS-7535-101 study, a total of 44 healthy subjects received doses ranging from 15 mg to 120 mg, and the incidence of treatment-emergent adverse events (TEAEs) was 86.4% (38/44), with all TEAEs being mild. No serious adverse events (SAEs), fatal TEAEs, or adverse events of special interest (AESIs) occurred. No subjects permanently discontinued treatment due to TEAEs. For detailed safety information, please refer to the HRS-7535 IB.

Study HRS-7535-102 was a single-dose, open-label study conducted in subjects with moderate renal impairment and subjects with normal renal function, aiming to compare the PK characteristics of HRS-7535 between these two groups. By Mar., 2024, a total of 16 subjects had been enrolled, including 8 subjects with moderate renal impairment and 8 subjects with normal renal function. All 16 subjects received a single dose of HRS-7535 30 mg. Three

subjects (37.5%) with moderate renal impairment experienced at least one TEAE, all of which were mild. No subjects experienced AESI, SAE, or TEAE leading to death or study withdrawal.
Study HRS-7535-103 is a bioavailability and food-effect study conducted in healthy subjects, aiming to evaluate the relative bioavailability of HRS-7535 with different formulation processes in healthy subjects and the effect of a high-fat meal on the PK characteristics of HRS-7535. The trial is ongoing and no relevant clinical data are available.
The HRS-7535-201 study is a multicenter, randomized, double-blind, placebo-controlled, parallel-group Phase II clinical study conducted in subjects with type 2 diabetes, aiming to evaluate the efficacy and safety of HRS-7535 in patients with type 2 diabetes. As of May 2024, preliminary results have been obtained. A total of 194 adult subjects with type 2 diabetes received doses ranging from 15 mg to 90 mg. In the HRS-7535 15 mg group, 30 mg group, 60 mg group, 90 mg group, and placebo group, 31 subjects (79.5%), 28 subjects (71.8%), 29 subjects (76.3%), 33 subjects (84.6%), and 28 subjects (71.8%) experienced TEAEs, respectively, with the majority being mild or moderate in severity. In the HRS-7535 15 mg group, 30 mg group, and 90 mg group, 3 subjects (7.7%), 2 subjects (5.1%), and 2 subjects (5.1%) experienced TEAEs leading to treatment discontinuation, respectively. In the HRS-7535 15 mg group, 30 mg group, and 90 mg group, 3 subjects (7.7%), 2 subjects (5.1%), and 1 subject (2.6%) experienced TEAEs leading to withdrawal from the study, respectively. After 16 weeks of treatment, significant reductions in glycated hemoglobin (HbA1c) were observed in all HRS-7535 dose groups. The mean changes in HbA1c from baseline in the HRS-7535 15 mg group, 30 mg group, 60 mg group, 90 mg group, and placebo group were -1.19%, -1.59%, -1.82%, -1.64%, and -0.25%, respectively.
HRS-7535-202 is a multicenter, randomized, double-blind, placebo-controlled, parallel-group Phase II clinical study conducted in obese subjects to evaluate the efficacy and safety of HRS-7535 in obese patients. The trial is ongoing and no relevant clinical data are available.
The HRS-7535-203 study is a multicenter, randomized, double-blind, placebo-controlled, parallel-group Phase II clinical study in subjects with type 2 diabetic kidney disease, aiming to evaluate the efficacy and safety of HRS-7535 in subjects with type 2 diabetic kidney disease. The trial is ongoing and no relevant clinical data are available.
Study HRS-7535-205 is a multicenter, randomized, double-blind, placebo-controlled, parallel-group Phase II clinical study conducted in subjects with type 2 diabetes inadequately controlled on metformin and SGLT2 inhibitors, aiming to evaluate the efficacy and safety of HRS-7535 in these subjects. The trial is ongoing and no relevant clinical data are available.

2.2. Summary of Benefits and Risks

2.2.1. Benefit Summary

Based on the clinical data of other GLP-1 receptor agonists and the preclinical data of HRS-7535, the known potential benefits for subjects with type 2 diabetes in the proposed study include: reduction in HbA1c, improvement in fasting plasma glucose (FPG), and weight loss. Additionally, all subjects are expected to benefit from the investigator's customized health guidance, dietary and exercise management, and comprehensive health assessments during the study.

2.2.2. Risk Summary and Management Measures

Based on the safety data as of Mar., 2024, a total of 52 healthy subjects were exposed to single or multiple doses of HRS-7535, among which 38 subjects (73.1%, 38/52) experienced HRS-7535-related adverse events (AEs). HRS-7535-related AEs with incidence ≥10% included nausea (57.7%), vomiting (48.1%), dizziness (26.9%), abdominal distension (21.2%), headache and proteinuria (19.2% each), and abdominal pain (13.5%). Among subjects with renal impairment, 8 subjects were exposed to a single dose of HRS-7535 30 mg, and no subjects experienced HRS-7535-related AEs.

Based on the safety data as of May 2024, a total of 155 subjects with type 2 diabetes (HRS-7535-201 study) received HRS-7535 treatment, among which 83 subjects (50.0%, 83/155) experienced HRS-7535-related AEs. HRS-7535-related AEs with incidence ≥5% included nausea (17.4%), decreased appetite (16.8%), lipase increased (9.7%), vomiting (9.0%), diarrhea (8.4%) and abdominal distension (5.2%).

Based on the clinical data of same-class products with the same target, combined with the existing clinical and preclinical data of HRS-7535, the known potential risks may include but are not limited to the following:

（1） Gastrointestinal reactions, including: nausea, vomiting, loss of appetite, abdominal pain, diarrhea, etc.;

（2） Occurrence of hypoglycemic episodes;

（3） Elevations in amylase and lipase;

（4） Cholelithiasis;

（5） Heart rate elevation.

2.2.3. Overall Benefit-Risk Conclusion

In conclusion, HRS-7535 has a positive benefit-risk relationship, and the potential risks to subjects participating in this trial are considered low and acceptable.

No important known risks or important potential risks have been identified for HRS-7535. Important risks will continue to be monitored in future studies, and risk minimization measures will be developed.

3. Study Objectives, Endpoints, and Estimands

3.1. Objectives and Endpoints

Table 2. Study Objectives and Endpoints

**Primary Objective**	**Primary Endpoint**
In adult subjects with type 2 diabetes who have inadequate glycemic control with diet and exercise alone, to demonstrate that the efficacy of HRS-7535 in controlling blood glucose is superior to that of placebo.	The change in HbA1c from baseline at week 32 in the HRS-7535 group compared with the placebo group.
**Secondary Objective**	**Secondary Endpoints**
In adult subjects with type 2 diabetes who have inadequate glycemic control with diet and exercise alone:<br>Evaluate the efficacy of HRS-7535 in other aspects of glycemic control;<br>Evaluate the efficacy of HRS-7535 in weight management.	Key Secondary efficacy endpoints:<br>At week 32 of treatment, the HRS-7535 group showed the following compared with the placebo group:<br>• Proportion of subjects reaching HbA1c < 7.0%<br>• Proportion of subjects reaching HbA1c ≤6.5%<br>• Change in FPG from baseline;<br>• Percentage change in weight from baseline;<br>Supportive Secondary Efficacy Endpoint:<br>At week 32 of treatment, the HRS-7535 group showed the following compared with the placebo group:<br>• Change in weight and waist circumference from baseline;<br>• Proportion of subjects reaching HbA1c < 5.7%<br>• Changes from baseline in fasting serum insulin, fasting serum C-peptide, homeostasis model assessment of β-cell function (HOMA-β), and insulin resistance index (HOMA-IR);<br>• Changes from baseline in 1-hour postprandial plasma glucose, 2-hour postprandial plasma glucose, 2-hour postprandial insulin, 2-hour postprandial C-peptide, and disposition index (DI).<br>• Change from baseline in 7-point SMBG profile;

	• Proportion of subjects requiring rescue treatment in each group<br>At week 52 of treatment, the HRS-7535 group showed the following compared with the placebo group:<br>• Change in HbA1c from baseline;<br>• Change in FPG relative to baseline;<br>• Percentage change in weight from baseline;<br>• Change in weight and waist circumference from baseline;<br>• Proportion of subjects reaching HbA1c < 7.0%<br>• Proportion of subjects reaching HbA1c ≤6.5%<br>• Proportion of subjects reaching HbA1c < 5.7%<br>• Changes from baseline in fasting serum insulin, fasting serum C-peptide, homeostasis model assessment of β-cell function (HOMA-β), and insulin resistance index (HOMA-IR);<br>• Changes from baseline in 1-hour postprandial plasma glucose, 2-hour postprandial plasma glucose, 2-hour postprandial insulin, 2-hour postprandial C-peptide, and disposition index (DI).<br>• Change from baseline in 7-point SMBG profile;<br>• Proportion of subjects requiring rescue treatment in each group
In adult subjects with type 2 diabetes with inadequate glycemic control by diet and exercise alone:<br>To evaluate the safety and tolerability of HRS-7535.	Week 32 and Week 52 of Treatment<br>• Adverse events, hypoglycemic events, laboratory assessment, vital signs, physical examination, 12-lead electrocardiogram, diabetic retinopathy screening and assessment.
In adult subjects with type 2 diabetes with inadequate glycemic control by diet and exercise alone:<br>To evaluate the pharmacokinetics (PK) of HRS-7535.	• Plasma HRS-7535 concentration.
In adult subjects with type 2 diabetes with inadequate glycemic control by diet and exercise alone:<br>To assess patient reported outcomes in each treatment group.	• Change from baseline to Week 32 and Week 52 in Diabetes Treatment Satisfaction Questionnaire status version (DTSQs).
**Exploratory Objective**	**Exploratory endpoints**
In adult subjects with type 2 diabetes with inadequate glycemic control by diet and exercise alone:	Compared with the placebo group, at week 32 and week 52 of treatment in the HRS-7535 group:<br>• Change from baseline in systolic blood pressure and diastolic blood pressure

To evaluate the efficacy of HRS-7535 in improving blood pressure, lipid metabolism, and renal function.	• Percentage change in TG, TC, LDL-C and HDL-C from baseline;<br>• Percentage change in UACR relative to baseline.

Abbreviations: DTSQ = Diabetes Treatment Satisfaction Questionnaire; FPG = Fasting Plasma Glucose; HbA1c = Glycated Hemoglobin; HDL-C = High-Density Lipoprotein Cholesterol; HOMA-IR = Homeostasis Model Assessment of Insulin Resistance; HOMA-β = Homeostasis Model Assessment of β-cell function; LDL-C = Low-Density Lipoprotein Cholesterol; PK = Pharmacokinetics; SMBG = Self-Monitoring of Blood Glucose; TC = Total Cholesterol; TG = Triglycerides; UACR = Urine Albumin/Creatinine Ratio.

3.2. Estimands

3.2.1. Primary Estimand

The primary estimand is for the primary efficacy endpoint (change in HbA1c from baseline at week 32 of treatment), with 5 attributes as follows:

Table 3. Definition of Primary Estimand and Handling Strategies

**Attribute:**	**Definitions and Strategies**
Treatment	HRS-7535 30 mg, 60 mg, 90 mg or placebo, orally administered, QD
Population	Adult subjects with type 2 diabetes with inadequate glycemic control by diet and exercise alone
Variable (endpoint)	Change from Baseline in HbA1c at Week 32
Intercurrent events and handling strategies	
Intercurrent event 1: premature discontinuation of study treatment	Treatment-policy strategy: if the subject prematurely discontinues study treatment before the primary endpoint at week 32, the subsequently collected data will be included in the statistical analysis
Intercurrent events 2: initiation of additional glucose-lowering agents*	Treatment-policy strategy: if the subject starts using additional glucose-lowering agents before the primary endpoint at week 32, the subsequently collected data will be included in the statistical analysis.
Summary at the Population Level	Difference in Mean Change from Baseline in HbA1c Between Groups at Week 32

Abbreviation: HbA1c=glycated hemoglobin; QD=once daily.
*Additional glucose-lowering agents are defined as any glucose-lowering agents used cumulatively for more than 14 days.

3.2.2. Supplementary Estimand

The handling strategies for intercurrent events 1-2 of the supplementary estimand both adopt hypothetical strategies, i.e., data collected after the occurrence of intercurrent events 1-2 are not included in the statistical analysis, while other attributes and settings of Intercurrent events are consistent with those of the primary estimand.

4. Study Design

4.1. Study Design Description

This trial is a multicenter, randomized, double-blind, placebo-controlled, parallel-group Phase III clinical trial, including a screening period of up to 2 weeks, a 3-week run-in period, a 52-week double-blind treatment period, and a 2-week safety follow-up period. The trial population consists of adult subjects with type 2 diabetes inadequately controlled by diet and exercise alone, with a planned enrollment of 272 subjects.

Screening phase (W-5 ~ W-3, up to 2 weeks)

After signing the Informed Consent Form, subjects will undergo screening assessment, and those who meet the inclusion/exclusion criteria may enter the run-in phase.

Run-in period (W-3 ~ W0)

Subjects who pass the screening will start continuous oral administration of single-blind run-in drug for 3 weeks at V2, D-21 (once daily).

Subjects undergo baseline assessment at V3 (D-4), and the assessment at V4 (D1) determines whether the subject meets the randomization criteria. Subjects who meet the randomization criteria enter the 52-week double-blind treatment period.

Double-Blind Treatment Period (W1-W52)

Subjects are randomized in a 1:1:1:1 ratio to the HRS-7535 30 mg group, 60 mg group, 90 mg group, and placebo group, with stratification based on baseline HbA1c values (≤8.5%, > 8.5%) measured by the central laboratory and prior use of glucose-lowering agents (yes, no). After randomization, subjects begin taking the double-blind treatment period medication.

From week 33 of the double-blind treatment period to the end of the double-blind treatment period, the placebo group is switched to HRS-7535 30 mg treatment. All subjects are required to undergo efficacy and safety assessments at the scheduled visit time points during the double-blind treatment period.

Safety follow-up phase (W53 ~ W54)

After discontinuation of the double-blind treatment period medication, subjects will complete the safety follow-up via telephone visit at Week 54.

4.2. Trial Design Basis

HRS-7535 is an oral small molecule GLP-1 receptor agonist intended for the treatment of type 2 diabetes and weight loss. Preliminary clinical trials suggest that HRS-7535 has good hypoglycemic effects and is expected to become a new treatment option for type 2 diabetes subjects with inadequate glycemic control after diet and exercise alone. The objective of this

trial is to evaluate the efficacy and safety of HRS-7535 compared with placebo in adult subjects with type 2 diabetes who have inadequate glycemic control with diet and exercise alone.

The study design refers to the "Technical Guidelines for Clinical Research and Development of Drugs for Adult Type 2 Diabetes Mellitus" and follows the feedback from CDE on the study design, adopting a multicenter, randomized, double-blind, placebo-controlled, parallel-group design to eliminate selection bias and information bias. The total trial duration is approximately 59 weeks, with a 52-week double-blind treatment period, which can meet the needs for observing efficacy and safety. After Week 33 of the double-blind treatment period, all subjects in the placebo group will begin receiving HRS-7535 30 mg treatment to reduce the ethical concerns that may arise from prolonged exposure to placebo. This trial included a run-in period before randomization to exclude subjects with poor compliance, placebo responders, or inadequate glycemic control before randomization. The primary endpoint is the change in HbA1c from baseline at Week 32. The change in HbA1c from baseline remains a recognized surrogate endpoint in current clinical trials of glucose-lowering drugs and is used as the primary efficacy endpoint in key studies to evaluate the glucose-lowering effect of the drug.

In addition, to better ensure the safety of subjects, this trial has set up rescue treatment, allowing subjects who meet the criteria for rescue treatment to receive other antidiabetic drugs. All subjects entering the double-blind treatment period are provided with a blood glucose meter for convenient self-monitoring of blood glucose, thereby minimizing the risk of worsening glycemic control. Subjects may withdraw from the trial at any time, and the investigator may also discontinue a subject's participation in the trial.

4.3. Access to Study Intervention After End of Study

The Investigational Product will only be provided to the subjects during this trial and will no longer be supplied after the trial ends.

4.4. Definition of Trial Initiation and Completion

The start of the trial is defined as follows:

First subject first visit (FSFV) (ie, when the first subject signs the informed consent form [ICF]).

The definition of the end of the trial is as follows:

Last subject last visit (LSLV) (i.e., the last visit of the last subject).

5. Trial Population

5.1. Selection of Trial Population

Subjects aged ≥18 and ≤75 years with type 2 diabetes mellitus who have inadequate glycemic control with diet and exercise alone.

5.2. Rationale for Selection of Trial Population

Based on the objective of this trial, adult subjects with type 2 diabetes who have inadequate glycemic control with diet and exercise alone are selected as the trial population. Enrolled subjects should be free of glucose-lowering agents at the time of enrollment, to better evaluate the efficacy and safety of HRS-7535 compared to placebo. The HbA1c cutoff of 7.0% to 10.0% is selected to include patients with type 2 diabetes requiring glucose-lowering therapy, while avoiding the inclusion of patients with poorer glycemic control that may increase safety risks. Considering the adverse reactions of HRS-7535 and other similar drugs, subjects with a medical history of pancreatitis, thyroid disorder, or significant gastrointestinal disease are excluded. In addition, subjects with a medical history of severe diseases (such as malignant neoplasm, cardiovascular disorder, or severe renal function) are also excluded to avoid increasing safety risks and affecting safety evaluation.

5.3. Inclusion Criteria

Subjects must meet all the following criteria to be enrolled in this trial:

1. Male or female, aged between 18 and 75 years (inclusive) on the day of signing the ICF;
2. Diagnosed with type 2 diabetes mellitus according to the Chinese Guidelines for the Prevention and Treatment of Type 2 Diabetes (2020 Edition), with a disease duration of ≥2 months at screening;
3. Blood glucose was managed through consistent dietary control and exercise within 2 months prior to screening, without the use of any glucose-lowering agents;
4. HbA1c levels between 7.0% and 10.0% (inclusive) at screening (as tested by a local laboratory);
5. Body mass index (BMI) of 19.0 – 40.0 kg/m^2 (inclusive) at screening;
6. Having voluntarily signed the ICF prior to trial initiation, subjects must fully understand the study content, procedures, and possible adverse reactions, and must be able and willing to complete the study in accordance with protocol requirements (e.g., self-monitoring of blood glucose and maintaining subject diaries);

7. From signing the ICF until 2 weeks after the last dose, subjects have no plans for pregnancy and are willing to use highly effective contraception as specified in the protocol.

5.4. Exclusion Criteria

Subjects who meet any of the following criteria shall not be enrolled in this trial:

Those with the following abnormalities at screening or randomization:

1. Presence of the following laboratory test abnormalities at screening or randomization:
    - Fasting venous glucose > 15 mmol/L;
    - Alanine aminotransferase (ALT) or aspartate aminotransferase (AST) > 2 × upper limit of normal (ULN);
    - Total bilirubin (TBIL) > 1.5 × ULN;
    - Amylase and/or lipase > 3 × ULN;
    - Fasting triglycerides (TG) > 11.3 mmol/L;
    - Calcitonin ≥50 ng/L;
    - Estimated glomerular filtration rate (eGFR) < 30 mL/min/1.73m^2 (calculated using the chronic kidney disease epidemiology collaboration [CKD-EPI] equation, see Section 13.5);13.5
    - Urinary albumin-to-creatinine ratio (UACR) ≥1000 mg/g;
    - Positive Hepatitis B Virus Surface Antigen (HBsAg) and HBV-DNA > ULN;
2. Clinically significant abnormalities in ECG results at screening or randomization that may affect subject safety, including but not limited to supraventricular tachycardia, atrial fibrillation, atrial flutter, second- or third-degree atrioventricular block, etc.;
3. During screening or randomization, the electrocardiogram examination results indicated a heart rate > 100 bpm or QTcF > 480 ms. If the heart rate or QTcF is abnormal, two additional electrocardiograms may be performed to obtain the mean of three values;
4. Uncontrolled severe hypertension (systolic blood pressure ≥160 mmHg or diastolic blood pressure ≥100 mmHg) at screening or randomization;

Those with the following diseases or medical history prior to screening:

5. Diagnosed or suspected type 1 diabetes mellitus, special type of diabetes mellitus or secondary diabetes mellitus;
6. Acute complications of diabetes (diabetic ketoacidosis, lactic acidosis, hyperglycemic hyperosmolar state, etc.) within 6 months prior to screening;
7. Severe hypoglycemic episodes or recurrent hypoglycemic episodes within 6 months prior to screening (≥ 3 hypoglycemic episodes within a week, or other hypoglycemic episodes as determined by the investigator);
8. Proliferative retinopathy or maculopathy, painful diabetic neuropathy, diabetic foot ulcer, or intermittent claudication requiring acute treatment at screening;
9. Subjects with known clinically significant diseases within 6 months prior to screening: including but not limited to neurological, psychiatric, cardiovascular, endocrine, gastrointestinal, respiratory, urinary, hematological, immune system and other diseases (except for type 2 diabetes related conditions) that may interfere with the study results or pose additional risks to the administration of the study drug as determined by the investigator;
10. Past or known history of medullary thyroid carcinoma (MTC) or multiple endocrine neoplasia syndrome type 2 (MEN2), or family history thereof;
11. Past or known medical history of acute or chronic pancreatitis, pancreatic injury, acute cholecystitis, or symptomatic/treatment-requiring gallbladder disease (excluding subjects who have undergone cholecystectomy and are determined by the investigator to be eligible for enrollment);
12. Past history of significant gastrointestinal diseases (such as gastric outlet obstruction, inflammatory bowel disease, active ulcer, etc.), or those who have undergone gastrointestinal surgery (except for gastrointestinal polypectomy and appendectomy), or those with a history of long-term use of medications that directly affect gastrointestinal motility;
13. History of hyperthyroidism prior to screening, or hyperthyroidism at screening, or other thyroid disorders not adequately controlled (except for subjects with thyroid hormone replacement treatment regimen stable for at least 8 weeks and no plan to change the treatment regimen during the study);
14. History of severe cardiovascular and cerebrovascular diseases within 6 months prior to screening, including but not limited to: congestive heart failure (New York Heart Association [NYHA] Class III–IV), unstable angina, stroke, myocardial infarction, severe

arrhythmia, or coronary revascularization; or planned to undergo coronary, carotid or peripheral artery revascularization at screening;

15. Subjects with known or suspected depression, bipolar disorder, suicidal tendency, schizophrenia or other serious mental diseases; or those with cognitive impairment or language barriers who are unable to fully understand the protocol or unwilling to cooperate with the study site staff.

16. History of malignant neoplasm within 5 years prior to screening, except for cured local cancer, such as: localized cutaneous basal cell carcinoma, cervical carcinoma in situ, and carcinoma in situ of the prostate;

Those who have received any of the following drugs or treatments prior to screening:

17. Use of GLP-1 receptor agonists (including multi-target GLP-1 receptor agonists) within 3 months before screening;

18. Use of systemic glucocorticoids (excluding those with cumulative use of <14 days or administration via topical, inhaled, intraocular, or intranasal routes) within 3 months prior to the screening;

19. Use of tricyclic antidepressants, atypical antipsychotics, and mood stabilizers (such as imipramine, chlorpromazine, amitriptyline, mirtazapine, clozapine, olanzapine, paroxetine, phenelzine, thioridazine, valproic acid and its derivatives, lithium salts, etc.) within 3 months before screening;

20. Weight loss therapy within 3 months prior to screening, including intensive diet or exercise therapy for weight loss, pharmacological therapy, surgical treatment (except for liposuction history more than 1 year), dietary supplements or other special therapies;

Those with any of the following conditions:

21. At screening or randomization, self-reported weight change exceeding ±5% within the previous 3 months, or planned intensive diet or exercise therapy for weight loss during the trial (except for diabetes diet and exercise control measures);

22. Those who have participated in any clinical trial of drugs or medical devices within 3 months prior to screening or still within 5 half-lives of the study drug (whichever is longer). Participation in a clinical trial is defined as signing informed consent, and having used the Investigational Product (including placebo) or investigational medical device;

23. Known or suspected hypersensitivity to the study drug or its components;

24. Known or suspected history of drug or alcohol abuse, and judged by the investigator as unsuitable for participation in this clinical trial;

25. Subjects with a history of blood donation or blood loss of ≥ 400 mL within 3 months prior to screening, or who have received blood transfusion;

26. Female subjects who are pregnant, breastfeeding, or planning to become pregnant during the trial;

27. Intolerance to venipuncture or needle phobia.

28. Any condition that, in the investigator's judgment, makes the subject unsuitable for participation in this clinical trial.

5.5. Lifestyle Requirements

During this study, starting from the screening visit, the investigator or designated personnel will provide guidance on healthy diet and lifestyle for diabetes to the subject at each visit. The subject should follow the corresponding guidance to manage their own diet and lifestyle. Any violations of the guidance should be recorded on the subject diary card, but these violations will not be considered as protocol deviations.

In addition, the following matters should be noted before each blood collection or assessment:

- Do not consume products containing caffeine or xanthine (such as coffee, tea, cola drinks, and chocolate) within 24 hours prior to each blood collection.
- Abstain from alcohol for 24 hours before each blood collection.
- Avoid strenuous exercise for 24 hours before each blood sampling for laboratory tests.

Female subjects of childbearing potential and male subjects whose female partners are of childbearing potential must use contraceptive measures (see Section 13.1 for details).13.1

5.6. Screen failure

The following subjects meet the definition of screen failure:

- Subjects who signed the informed consent failed to meet the screening criteria;
- Subjects who signed the informed consent did not continue to participate in the trial before confirming whether they met the screening criteria.

Rescreening of subjects who have previously failed screening is allowed, provided that each subject is allowed only once throughout the course of the trial. They must sign the ICF again, re-register and obtain a new subject number at the re-screening.

The following subjects meet the definition of randomization failure:

- Subjects who passed screening but failed to meet the randomization criteria;

- Subjects who passed the screening did not continue to participate in the trial before confirming whether they met the randomization criteria;
- Subjects who passed the screening and met the randomization criteria but did not participate in randomization.

The investigator should inform the subjects who failed randomization to discontinue the drugs of run-in period, return the packaging and the remaining drugs, and provide the subjects with reasonable clinical treatment measures. Subjects who failed randomization are not eligible for rescreening.

The necessary information of screening/randomization failure should be recorded to ensure the transparency of the report, to meet the requirements of the Consolidated Standards of Reporting Trials (CONSORT), and to respond to the queries of the regulatory authorities. This information includes demographics, informed consent, reason for screening/randomization failure, inclusion and exclusion criteria, and any SAE.

6. Trial Intervention and Concomitant Treatment

6.1. Information of Investigational Intervention

The investigational interventions in this trial are all Investigational Products, including: HRS-7535 and HRS-7535 placebo. The Investigational Products are provided by the sponsor, uniformly packaged and tested to be qualified. The relevant information is shown in Table 4.Table 4. Investigational Product Information

Table 4. Investigational Product Information

**Product Name**	**HRS-7535**	**HRS-7535 placebo**
**Type**	Study Drug	Comparator (product)
**Dosage Form**	Tablet	Tablet
**Strength**	30 mg、60 mg	30 mg、60 mg
**Storage Conditions**	Protected from light and sealed, stored below 25°C	Protected from light and sealed, stored below 25°C
**Packaging**	Hard aluminum-aluminum blister packaging	Hard aluminum-aluminum blister packaging
**Shelf Life**	See the Investigational Product packaging for details.	See the Investigational Product packaging for details.
**Manufacturer**	Shandong Suncadia Medicine Co., Ltd.	Shandong Suncadia Medicine Co., Ltd.

6.2. Selection of Trial Intervention and Basis

GLP-1 receptor agonists are a new class of oral antidiabetic drugs that can effectively reduce blood glucose, partially restore pancreatic β-cell function, reduce body weight, improve lipid profiles and lower blood pressure, with proven protective effects on the cardiovascular system and kidneys. HRS-7535 is an oral small molecule GLP-1 receptor agonist with convenient administration, which is expected to become a new option for the treatment of type 2 diabetes. To evaluate the absolute efficacy and true adverse reactions of the study drug and to reduce trial bias, placebo is selected as the comparator. To address the ethical concern of prolonged exposure to placebo in the placebo group, the placebo group will receive HRS-7535 30 mg after week 33 of the double-blind treatment period.

6.2.1. Rationale for Dose Selection

In this study, 30 mg, 60 mg, and 90 mg are selected as the target doses.

Based on the results of previous clinical studies, HRS-7535 demonstrated good safety and tolerability at doses ranging from 15 - 90 mg.

Based on the existing clinical study data, a population pharmacokinetics (PopPK) model was constructed to simulate and calculate the AUCss of subjects, and to evaluate the relationship between AUCss and HbA1c as well as body weight. The analysis results showed that with the increase of average exposure to HRS-7535, both HbA1c and body weight exhibited a decreasing trend. Among them, HbA1c began to show a more obvious reduction effect at the dosage of 30 mg, and essentially reached a plateau in drug effect at the 60 mg dose. With the increase of exposure, the weight loss effect increased, and a more obvious effect was observed at the dosage of 90 mg.

Based on the safety and tolerability data of the HRS-7535-201 study, the impact of different titration starting doses and dose escalation increments on tolerability is evaluated to develop the dosing regimen. According to the study results, a titration regimen with 30 mg as the starting dose and a 30 mg increase every 4 weeks can achieve sufficient tolerability to gastrointestinal adverse events.

6.3. Dosage and Administration

6.3.1. Dosing Regimen

Run-in period (single-blind)

All subjects received HRS-7535 placebo (1 tablet each of 30 mg and 60 mg specifications), QD. The Investigational Product is allowed to be taken on the day of V2 or the next day, with the dosing day as day 1 of the run-in period. Double-blind treatment period

Approximately 272 subjects will be randomized in a 1:1:1:1 ratio to the HRS-7535 30 mg group, 60 mg group, 90 mg group, and placebo group, with stratification based on baseline HbA1c values (≤8.5%, > 8.5%) measured by the central laboratory and prior use of glucose-lowering agents (yes, no). The double-blind treatment period medication will be initiated after randomization. The Investigational Product may be administered on the day of randomization or the following day, with the dosing day designated as Day 1 of the double-blind treatment period. The dosing regimens for each group are as follows:

- HRS-7535 30 mg group: HRS-7535 30 mg, administered orally, QD.
- The HRS-7535 60 mg group: dose titration is adopted. The subjects received HRS-7535 30 mg, orally administered, QD for the first 4 weeks (D1 to D28); after Week 5 (after D29), they received HRS-7535 60 mg, orally administered, QD.
- HRS-7535 90 mg group: dose titration is adopted. For the first 4 weeks (D1 to D28), HRS-7535 30 mg administered orally, QD; from Week 5 to Week 8 (D29 to D56), HRS-7535 60 mg administered orally, QD; after Week 9 (after D57), HRS-7535 90 mg is administered orally, QD.
- Placebo group: from Week 1 to Week 32 (D1 to D224), receive HRS-7535 Placebo (1 tablet each of 30 mg and 60 mg specifications), QD; after Week 33 (after D225), receive HRS-7535 30 mg, orally administered, QD.

To ensure the implementation of blinding, the drug dosage and corresponding number of tablets for each dose level during the run-in phase and double-blind treatment phase are as follows:

Table 5. Drug Dosage and Corresponding Number of Tablets During the Trial

Dose Level / Investigational Product	Run-in period:	Double-blind treatment period			
	All Subjects	HRS-7535 30 mg	HRS-7535 60 mg	HRS-7535 90 mg	Placebo
HRS-7535 30 mg	0	1	0	1	0
HRS-7535 30 mg placebo	1	0	1	0	1
HRS-7535 60 mg	0	0	1	1	0
HRS-7535 60 mg placebo	1	1	0	0	1

6.3.2. Medication Guidance

The subject should take the Investigational Product once daily with water, swallow it whole, and should not hold or chew the drug before swallowing. It may be taken before or after meals. It is recommended that the subject take the medication at approximately the same time each day and not exceed the dosage specified in the protocol. Missed dose guidance: if the subject misses a dose of the Investigational Product on any day, they should take the prescribed dose on the following day as scheduled and should not make up for the missed dose from the previous day.

Guidance for vomiting after medication: if a subject vomits after taking the Investigational Product, regardless of whether the vomitus contains the Investigational Product, they should not take an additional dose but should continue with the next day's Investigational Product as per the protocol.

Guidance for overdosing: if the subject takes more than the prescribed amount of the Investigational Product, they must inform the Investigator. The Investigator and the Sponsor will jointly decide whether to adjust the dosage of the medication subsequently.

During the single-blind run-in phase, the subject receives the run-in phase Investigational Product HRS-7535 placebo (1 tablet each of 30 mg and 60 mg specifications). In order to not affect the subject's medication compliance, the investigator must comply with the requirements of the single-blind design and not inform the subject of the drug components taken.

6.3.3. Dose Adjustment

Except for the provisions in Section 6.3.3.1 and Section 6.3.3.2, dose adjustment of HRS-7535/placebo is not allowed during the trial.6.3.3.16.3.3.2

6.3.3.1. Gastrointestinal System Symptom Management

During the trial, if intolerable gastrointestinal symptoms occur, the investigator should implement the following steps:

1. It is recommended that the subject take the medication after meals and eat small, frequent meals. e.g., changing from three to four or more meals a day, and to stop eating immediately upon feeling full.
2. If changes in dietary patterns cannot effectively improve gastrointestinal symptoms, symptomatic treatment with antiemetics, gastroprotective agents, or antidiarrheals may be administered based on symptoms and needs.

3. When the subject continues to have intolerable gastrointestinal system symptoms after the above management measures, the investigator may adjust the dosage of the study drug according to the following rules:

- Within 1–4 weeks post-randomization (V4–V6, excluding the day of V6 visit): after 1–2 days of study treatment suspended, continue with the planned study treatment.
- Within 5–8 weeks post-randomization (V6–V7, excluding the day of V7 visit): after study treatment suspended for 1–2 days, continue with the planned study treatment. For those still intolerable, return to the study site for re-dispensing.
- Between 9 to 52 weeks post-randomization (V7 to V15): after study treatment suspended for 1 to 2 days, continue taking partial drugs from the packaging under the investigator's guidance (temporarily discontinue HRS-7535/placebo 30 mg) to complete dose adjustment. Subjects who still cannot tolerate should return to the study site for re-dispensing.

4. When the subject undergoes the above dose adjustment:

- If the investigator determines that the subject has persistent intolerable gastrointestinal system symptoms, and the investigator may terminate the study treatment early.
- For subjects who do not receive re-dispensing, if the gastrointestinal system symptoms are tolerable or resolved, they may attempt to take all the medications in the drug packaging again to re-escalate to the highest maintenance dose at randomization; or they may maintain the current dose-adjusted treatment regimen until the end of the double-blind treatment period.
- Subjects who received re-dispensing will be adjusted to the lowest initial dose at randomization until the end of the double-blind treatment period. The results of dose adjustments at each stage are summarized as follows:

**Subject Group**	**Discontinuation of HRS-7535/placebo 30 mg,<br>The dosage will be adjusted to**	**After returning to the study site for re-dispensing,<br>The dosage will be adjusted to**
Placebo	Placebo	Placebo
30 mg	Placebo	30 mg
60 mg	60 mg	30 mg
90 mg	60 mg	30 mg

The above dose adjustment steps are general principles. The investigator may actively contact the sponsor based on the actual situation to jointly develop an individualized dose adjustment

strategy under the premise of maintaining blinding, in order to fully minimize the risk of gastrointestinal system symptoms for the subject.

6.3.3.2. Temporary Dose Interruption

If the study drug dosage is interrupted for reasons other than those specified in Section 6.3.3.1, the investigator should refer to the table below to guide the subject to resume the corresponding study treatment as soon as possible, provided that the subject's safety and tolerability are acceptable.6.3.3.1

Subject visit	Number of Dose Interruption Days	Dose Adjustment
Within 1 to 8 weeks after randomization (V4 to V7, excluding the day of V7 visit)	Any day	Continue to receive the planned study treatment as per the protocol
Weeks 9 to 52 after randomization (after V7, including the V7 visit day)	< 3 days	Continue to receive the planned study treatment as per the protocol
	3 to 5 days	Select one dose adjustment strategy based on the subject's previous tolerance:<br>（1） Continue to receive the current study treatment according to the protocol;<br>（2） Take part of the drugs in the packaging (suspend HRS-7535/Placebo 30 mg), if the gastrointestinal system symptoms are tolerable or subside, take all the drugs in the packaging to re-escalate to the highest maintenance dosage at randomization.
	≥6 days	Take part of the drugs in the packaging (suspend HRS-7535/placebo 30 mg), if the symptoms of the gastrointestinal system are tolerable or subside, take all the drugs in the packaging. To re-escalate to the highest maintenance dosage at randomization.

If the subject with temporary dose interruption experiences gastrointestinal system symptoms during the above-mentioned dose adjustment and treatment, please refer to Section 6.3.3.1 for gastrointestinal system symptom management and dose adjustment.6.3.3.1

The above dose adjustment steps are general principles. The investigator may actively contact the sponsor according to the actual situation, and jointly develop an individualized dose adjustment strategy under the premise of maintaining blinding, so as to fully ensure the minimization of risk for the subject.

6.4. Overdose Treatment

For the definition of overdose, please refer to Section 12.1. In the event of a drug overdose, it is recommended to monitor the subject for any symptoms or signs of adverse reactions and

immediately provide appropriate symptomatic treatment.12.1

6.5. Medicinal Product Preparation, Handling, Storage and Accountability

6.5.1. Preparation of medicinal product

All Investigational Products will be prepared by qualified personnel according to the drug manual and dispensed to subjects in a blinded form.

6.5.2. Medicinal Product Handling and Storage

The investigator or his/her authorized representative (e.g., a pharmacist) will ensure that all Investigational Products are stored in a secure and access-controlled area conforming to storage conditions, and their storage complies with applicable regulatory requirements.

Site systems must be capable of measuring and recording (e.g. via a log) at least daily minimum and maximum temperatures for all site storage locations (including frozen, refrigerated and/or room temperature products, as applicable). This data must be collected continuously from the time of IMP receipt through the duration of the study. Even with a continuous monitoring system in place, there should be logs or study site procedures to ensure proactive assessment of deviations. This is to ensure that no deviations have occurred since the last assessment by checking the minimum and maximum temperatures each working day, and to ensure that after normal operation is restored, the study site can store or review the minimum/maximum temperatures for all non-working days. Temperature monitoring equipment and storage devices (e.g. refrigerators) shall be checked regularly for operation (if applicable) to ensure they are in proper working condition.

Any deviations from the product label conditions should be reported promptly once detected. The study site should take active measures to place the product under the storage conditions described on the label as soon as possible, and report the temperature deviation and measures taken to the sponsor.

The Investigational Product affected by the temperature deviation should be isolated temporarily in the environment meeting the drug storage conditions, and can only be used after receiving permission from the Sponsor. If the Sponsor allows the continued use of the Investigational Product after the temperature deviation, this deviation will not be recorded as a Protocol deviation. However, continuing to use the Investigational Product before obtaining the Sponsor's permission is considered a Protocol deviation. The Sponsor will provide the Study site with specific steps to report the temperature deviation.

Storage requirements for HRS-7535/Placebo: sealed, stored at no more than 25°C. For details, refer to the Pharmacy Manual.

The study site should store all medicinal products in accordance with the storage requirements from the date of receipt of the Investigational Product to the period of drug recovery. The study institution should keep the Investigational Product in accordance with the relevant regulatory requirements, and should not transfer the Investigational Product to any non-clinical trial participants, unauthorized personnel and third parties.

For the study in which subjects are allowed to take medicinal products home, the staff of the study site will guide the subjects on the correct way to store medicinal products.

6.5.3. Management of Medicinal Products

Qualified study team personnel will dispense the Investigational Product according to the quantity specified in the process each time. Throughout the trial, the subject should store the drugs as instructed and must return all unused drugs and packaging to the investigator at each visit and at the end of the trial.

The study site must maintain records of the Investigational Product supply, including receipt, distribution, use, recovery, loss, or other disposition. Under no circumstances shall the Investigator provide the Investigational Product to a third party, allow its use for purposes other than those specified in the clinical study protocol, or dispose of the Investigational Product in any other manner.

6.5.3.1. Medicinal Product Destruction

If applicable, the sponsor or their authorized personnel will instruct about how to destroy unused and recovered Investigational Products. If the study site is authorized to destroy the Investigational Product, the investigator must ensure that the destruction of the Investigational Product complies with applicable environmental regulations, institutional policies and other provisions, and provide relevant procedures for destruction. All the destructions should be well recorded.

6.6. Subject Assignment, Randomization, and Blinding

6.6.1. Subject Assignment

After the subject signs the written Informed Consent Form, a subject coding will be automatically assigned, which will be used for all electronic Case Report Forms (eCRFs) and study documentation. Subjects who complete all screening procedures and meet the inclusion/exclusion criteria will be formally enrolled in this trial.

Subjects who successfully pass the screening will receive a 3-week run-in phase (single-blind) drug therapy. Baseline assessment will be conducted at V3 (D-4), and V4 (D1) will assess whether the subjects meet the randomization criteria. Subjects who meet the randomization criteria will be randomized, obtain a randomization number, and receive one or more drug numbers according to the randomization-related protocol, followed by the corresponding double-blind Investigational Product during the treatment period.

Randomized subjects who withdraw from the clinical trial for any reason, regardless of whether the Investigational Product is used or not, will retain their randomization number, and the subject will not be allowed to enter the trial again.

Before the start of the trial, the sponsor provides HRS-7535 and HRS-7535 placebo with existing drug numbers to the study center. After subjects pass screening, they are given the run-in phase medication. Upon enrollment, subjects are randomized by the system and provided with the double-blind Investigational Product bearing the corresponding drug number.

6.6.2. Randomization

The study adopts a stratified block randomization method, with baseline HbA1c values (≤8.5%, > 8.5%) measured by the central laboratory and previous use of glucose-lowering agents (yes, no) as stratification factors. Eligible subjects are randomly assigned to different groups in a 1:1:1:1 ratio.

The randomization specialist uses SAS software to complete the generation of the randomization table and Medicinal Product number table, randomizes subjects who met the randomization criteria using the Interactive Web Response System (IWRS), and gives the corresponding group of Medicinal Product treatment according to the enrollment results. A randomized subject is defined as a subject who is registered and assigned with a randomized treatment group from the IWRS, regardless whether the treatment is used or not. A subject cannot be randomized more than once in this study.

6.6.2.1. Randomization Criteria

Based on the V3 assessment results, the subject must be reconfirmed to meet the following randomization criteria before randomization. Subjects who fail randomization in this study are not allowed to re-enter the trial.

1. HbA1c levels between 7.0% and 10.0% (inclusive) (as tested by a central laboratory);
2. Glucose under fasting conditions ≤15.0 mmol/L (central laboratory results);
3. Medication compliance ≥ 80% and ≤ 120% during the run-in phase;

4. Laboratory tests, vital signs, body weight, BMI, electrocardiogram, pregnancy status, etc. met the inclusion criteria and did not meet the exclusion criteria;
5. No glucose-lowering agents were used;
6. No condition considered by the investigator to be unsuitable for participation in this clinical trial occurred during the run-in period.

6.6.3. Blinding and Unblinding

The study adopts the stratified block randomization method to randomize subjects into 4 groups in a 1:1:1:1 ratio. The randomization specialist uses SAS statistical software to complete the generation of the randomization table and drug coding list. Under the guidance of the blinding personnel, the sponsor’s production department packages the medicinal products according to the drug number. The labels of the investigational medicinal product and placebo are completely identical except for the drug number. Blinding must be maintained throughout subject enrollment and administration, documentation and evaluation of study results, assessment of the study process, and data management. The subjects, investigators, sponsor's researchers participating in the clinical evaluation, and the clinical research associate (CRA) participating in the study are not aware of the treatment allocation. The third-party laboratory testing personnel are unblinded and will not perform PK testing for the placebo group. To control any potential study bias, the blind codes will be properly kept as confidential information until the study database is locked so as to prevent disclosure to the investigators and study staff in direct contact with the investigators.

In the event of the following emergencies: (1) SAE; (2) the subject requires urgent rescue, the investigator shall decide whether to perform emergency unblinding. If possible, before unblinding the study drug, the investigator should first contact the sponsor to discuss the need for unblinding. If the sponsor is not informed in advance, the investigator shall notify the sponsor as soon as possible after unblinding.

After data verification in a blinded state and data lock, the statistician requests unblinding with the randomization specialist. This involves disclosing the subject's group assignment and medication code to the statistician for statistical analysis of all data.

6.7. Compliance with Trial Intervention

Compliance with trial intervention = actual number of tablets taken/theoretical number of tablets to be taken × 100%. Compliance during the run-in period, between Visits in the double-

blind treatment period (excluding telephone visits), and overall compliance during the double-blind treatment period should all be ≥ 80% and ≤ 120%.

Throughout the trial, the investigator should remind the subjects to comply with the protocol and requirements to ensure subject compliance. At each study visit or at the end of the trial, subjects should return all partially used and unused Investigational Products and packaging to the study site, where the returned Investigational Products and packaging will be recorded and archived.

At each visit, the investigator should assess the subject's intake of the Investigational Product based on drug accountability. The investigator (or designee) will document in detail the dispensing and recovery of the Investigational Product at each visit and the reason for poor compliance in the source documents (if applicable).

If the subject is found to have poor compliance, the investigator must inform the subject of the importance of using the Investigational Product according to the protocol. Subjects with severely poor compliance, as determined by the investigator, may be withdrawn from the trial early.

6.8. Concomitant treatment

Concomitant treatment refers to the treatment that is given for the interest of the subject as determined by the investigator.

All concomitant medications, blood products, and non-drug interventions administered from the screening phase through the end of trial visits will be documented in the eCRF.

In principle, subjects should avoid receiving any concomitant treatment during the trial. If a subject experiences an AE, the investigator may give appropriate treatment for the benefit of the subject; before using the treatment, if possible, the investigator should discuss with the sponsor to determine the concomitant treatment to be used.

6.8.1. Medications for Gastrointestinal Symptoms

If a subject experiences gastrointestinal symptoms (such as nausea, vomiting, and diarrhea) considered related to the study drug, it is advisable for the subject to manage symptoms by resting, taking the medication after meals, and eating smaller, more frequent meals. If the subject's gastrointestinal symptoms do not improve with the aforementioned treatments, the investigator may decide to use symptomatic treatments such as antiemetics, gastroprotective agents, or antidiarrheals, and must inform the sponsor and record this information in the eCRF, as detailed in Section 6.3.3.1.6.3.3.1

6.8.2. Prohibited Concomitant Treatment

From the screening phase to the end of the safety follow-up phase, subjects may use drugs for the treatment of underlying diseases and AEs, and in principle, no other drugs are recommended.

Subjects are prohibited from using the following drugs from the screening phase to the last dose of Investigational Product.

- Any glucose-lowering medications or treatments other than the study drug and rescue treatment medications;

Note:

- *Rescue treatment is prohibited from using: drugs containing GLP-1 receptor agonist components (including multi-target drugs), DPP-4 inhibitors.*
- *In certain clinical situations (e.g., elective surgery, hospitalization, hyperosmolar state), short-term use of insulin with a cumulative duration of < 14 days is allowed, and when reported in the eCRF, this insulin use must be distinguished from insulin use as rescue treatment.*

- Any medication or treatment that in the investigator's opinion could significantly interfere with glucose metabolism, such as any systemic corticosteroid therapy lasting ≥14 days (excluding topical, inhaled, intraocular, intranasal, or intra-articular administration).
- Drugs approved for weight loss, such as orlistat, liraglutide, benaglutide, etc.;
- Certain other drugs that may cause increased blood sugar, including but not limited to phenothiazine, estrogen, phenytoin, nicotinic acid, and isoniazid.
- Growth hormone or somatostatin analogues;
- Other incretin drugs;
- Any drug or herbal medicine with known common toxic reactions to major organs, or any drug that may interfere with the interpretation of efficacy and safety data.

After the last study treatment, the investigator, based on clinical experience and the subject's glycemic control, formulates a new antidiabetic regimen for the subject. The use of the following antidiabetic medications is strictly restricted within a certain time limit:

- GLP-1 receptor agonist-containing drugs are prohibited from the last study treatment until the end of the safety follow-up phase.
- DPP-4 inhibitors are prohibited within 14 days after the last study treatment.

6.8.3. Permitted Concomitant Treatment

In principle, subjects should avoid receiving any concomitant treatment during the trial. If a subject experiences an AE, the investigator may give appropriate treatment for the benefit of the subject; before using the treatment, if possible, the investigator should discuss with the sponsor to determine the concomitant treatment to be used.

6.8.4. Rescue Treatment

During the double-blind treatment period, if a subject experiences fasting plasma glucose levels that meet the criteria for rescue treatment (see Table 6) without any apparent trigger, they must undergo an unscheduled visit within 7 days (including the 7th day) under normal dietary conditions for fasting venous blood glucose testing at a local laboratory to confirm if the criteria for rescue treatment are met.

Table 6. Rescue Treatment Criteria for Fasting Plasma Glucose Levels

Study Time Period	Rescue Treatment Criteria for Fasting Plasma Glucose Levels
Double-Blind Treatment Period (Weeks 2-8)	>15.0 mmol/L
Double-Blind Treatment Period (Weeks 9-33)	>13.3 mmol/L
Double-Blind Treatment Period (Weeks 34-52)	>11.1 mmol/L

If the fasting venous blood glucose levels tested by the local laboratory (including results from both scheduled and unscheduled visits) meet the criteria for rescue treatment, the investigator may provide rescue treatment based on the subject’s medical needs, with rescue medication options selected from the approved antidiabetic regimens in the protocol. It is recommended to prioritize metformin, while insulin is suitable for severe persistent hyperglycemia or other clinical situations where the investigator deems rapid blood glucose control necessary. During the rescue treatment period, subjects will continue to receive the study treatment according to the protocol.

In addition, during the run-in phase, if a subject's self-measured fasting glucose is > 15 mmol/L, an unscheduled visit must be conducted within 7 days (including the 7th day) under normal dietary conditions after the day of awareness for fasting venous blood glucose testing at a local laboratory. If the test confirms venous fasting glucose > 15 mmol/L, the investigator will inform the subject to discontinue the Investigational Product during the run-in phase and permanently discontinue from the study.

7. Termination of Trial Intervention or Withdrawal from Trial

7.1. Termination of Trial Intervention for Subject

7.1.1. Discontinuation of Trial Intervention

Early discontinuation of study treatment does not represent withdrawal from the study. Subjects who discontinue the study treatment should continue to complete all items in the remaining study visits as required by the protocol to the greatest extent possible.

The subject should permanently discontinue study treatment if any of the following situations occur:

- The subject requested early withdrawal from the study and premature discontinuation of study treatment;
- Any AE, laboratory test abnormality, or other medical condition arises, and the investigator determines that continuing treatment with the Investigational Product is not in the subject's best interest;
- Poor glycemic control in the subject: during the run-in phase, the subject's fasting venous blood glucose was > 15.0 mmol/L (if self-monitored blood glucose [SMBG] > 15.0 mmol/L, the subject is required to return to the study site within 7 days for confirmation by the study site); during the treatment period, the subject still developed progressively worsening hyperglycemia after receiving rescue treatment (refer to the rescue treatment criteria in Table 6).Table 6. Rescue Treatment Criteria for Fasting Plasma Glucose Levels
- Amylase and/or lipase > 3 × ULN, confirmed by repeat testing within 3 days, and confirmed as pancreatitis by clinical symptoms or imaging examination;
- The subject participates in another clinical trial involving an approved or unapproved Investigational Product;
- Poor compliance of the subject, which may significantly affect the efficacy and safety evaluation.
- A female subject becomes pregnant during the study;
- Lost to follow-up.
- Death of the subject.
- Early termination of the trial by the Sponsor;
- Important protocol deviations, e.g., the subject is no longer eligible to continue participating in the trial after enrollment;

- Any other situation where the investigator determines it is necessary to discontinue treatment with the Investigational Product.

When the subject discontinues treatment, the subject will continue to complete the remaining study visits as required by the protocol until the safety visit. During the visits after discontinuation, the investigator will not perform the following: dispense Investigational Product, PK sampling. The investigator may suggest or provide new replacement treatments (except GLP-1 receptor agonists and other prohibited medications, see section 6.8.2) to subjects based on their actual situation, and the new dose administration should be recorded in the subject diary.6.8.2

7.2. Withdrawal of Subject from the Trial

Subjects may voluntarily withdraw from the trial at any time, or be asked to withdraw from the trial at any time by the investigator or the sponsor due to safety or behavioral reasons, or inability to comply with the study visit times or procedures required by the protocol at the study site where they are located.

If a subject fails to return to the site for follow-up as agreed, every effort should be made to contact the subject. In any case, every effort should be made to document the subject's outcome, if possible. The investigator should inquire about the reason for withdrawal, request the subject to return any unused Investigational Product, request the subject to return to the site for a final visit, and follow-up on any unresolved AEs if applicable.

If a subject refuses to go to the study site for further visits, safety information should continue to be followed up unless the subject also refuses to disclose further information or to be contacted. In this case, no study evaluation should be conducted, and no data should be collected.

For subjects who are lost to follow-up or have died, the steps for study withdrawal will no longer be applicable.

7.3. Lost to Follow-up

If a subject misses scheduled visits at the study site more than 3 times and the study site is unable to establish contact with the subject, the subject is considered lost to follow-up.

If a subject fails to return to the study site for necessary study visits, the following actions shall be taken:

- The study site should make efforts to contact the subject, reschedule any missed visits promptly, provide counseling to the subject about the importance of attending visits on time, and determine whether the subject wishes to and/or should continue with the trial.
- Before considering a subject as lost to follow-up, the investigator or designated personnel shall make every possible effort to contact the subject (e.g., attempting to contact the subject at least three times by phone and, if necessary, sending emails to their email address, mailing letters to their postal address, and using social media tools to establish communication). All the attempts to make contact should be recorded in the medical document.
- If the investigator is still unable to contact the subject, the subject is considered as withdrawal from the trial, with the primary reason being lost to follow-up.

7.4. Specific Discontinuation Criteria (Not Applicable)

Not applicable.

8. Trial Evaluation and Procedure

Examinations and procedures specified in the protocol should be performed to the best extent possible in accordance with the protocol. However, unexpected situations beyond the control of the investigators may occur, making examinations difficult. Under such circumstances, the investigator must take all necessary measures to protect the safety and interests of the subjects. When the procedure required by the protocol cannot be performed, the investigator must record the reason. In addition, the study team must be notified by the investigator of unexpected situations in a timely manner.

This clinical trial should be conducted in accordance with the study protocol, the current Good Clinical Practice (GCP) and relevant laws and regulations.

Subjects must be under fasting conditions for all study site visits. Fasting is defined as no intake of food or liquids other than water and medications for at least 8 hours overnight.

Early withdrawal from the study: after randomization, subjects who are unable or unwilling to continue the study for any reason should undergo an early withdrawal visit. If a subject discontinues the study during the visit, the full content of the early withdrawal visit should be completed, and this visit may serve as the early withdrawal visit.

Safety follow-up visit: all subjects who have taken the Investigational Product (including those who entered the run-in phase but were not successfully randomized) are required to undergo safety follow-up. Subjects who withdraw early from the study are also required to undergo safety follow-up visits.

8.1. Screening and Run-in phase Assessments and Procedures

The assessment items during the screening phase are detailed in Section 1.3 Schedule of Activities.1.3

In the run-in phase, eligible subjects who pass the screening uniformly take HRS-7535 placebo. At V3 (D-4), assessments will be conducted again. At V4 (D1), randomization criteria are reviewed. Subjects who meet the randomization criteria are randomized in a 1:1:1:1 ratio to the HRS-7535 30 mg group, 60 mg group, 90 mg group, and placebo group.

8.2. Assessment of Efficacy and Procedures

Assessment of Efficacy is conducted at the time points specified in the Schedule of Activities in Section 1.3. The efficacy assessment measures in this study include HbA1c, fasting plasma glucose (FPG), 1-hour postprandial plasma glucose, 2-hour postprandial plasma glucose, fasting serum insulin and C-peptide, 2-hour postprandial serum insulin and C-peptide, homeostasis model assessment of β-cell function (HOMA-β) and insulin resistance index (HOMA-IR), disposition index (DI), 7-point SMBG profile, body weight, and waist circumference.1.3

8.2.1. Glycated hemoglobin (HbA1c)

For screening, HbA1c will be assessed at the local laboratory. For efficacy assessment, HbA1c will be measured at the central laboratory during subsequent visits.

8.2.2. Fasting Plasma Glucose (FPG)

During the screening phase, FPG will be assessed at local laboratory. For efficacy assessment, FPG will be measured at central laboratory during subsequent visits.

8.2.3. Self-measured blood glucose (SMBG)

After successful screening, a glucose meter should be provided to the subject, and all glucose measurements during the study must be completed using this meter. In addition to 7-point SMBG and pre-breakfast SMBG, the subject may perform SMBG as needed (e.g., when symptoms of hypoglycemia or severe hyperglycemia occur), and it is recommended that the subject monitors pre-breakfast SMBG at least once a week. The subject should be provided

with oral and written instructions on the use of the glucose meter, including regular calibration according to the manufacturer's instructions. At subsequent visits, site staff should repeat the instructions for using the meter to the subject as necessary. The subject should be instructed to bring the glucose meter and subject diary at every site visit and should be trained on meter calibration as needed. The glucose meter uses test strips calibrated to plasma values, so all measurements obtained from capillary blood are automatically converted to plasma glucose equivalents and displayed on the meter, which are then recorded as SMBG values. The subject should be instructed on how to log SMBG values in the provided diary and should only record measurements obtained with the provided glucose meter.

For the 7-point SMBG profile, subjects should be instructed to perform continuous 7-point SMBG monitoring on any one day within 3 days before the visit (starting from before breakfast on that day and completing 7-point blood glucose collection within 24 hours) and record the SMBG values. On the measurement day, subjects should maintain normal diet and exercise levels. If a subject fails to complete one continuous 7-point SMBG test, a second 7-point SMBG monitoring should be performed on a different day.

The SMBG values at the following time points should be recorded in the subject's diary and eCRF (including the date of measurement and actual clock time), always starting with the pre-breakfast measurement:

- Before breakfast
- 120 ± 10 min after the start of breakfast
- Before lunch (recommended within 30 min before meals)
- 120 ± 10 min after the start of lunch
- Before dinner (recommended within 30 min before meals)
- 120 ± 10 min after the start of dinner
- Before bedtime

8.2.4. Mixed meal test

The assessment under fasting conditions must be completed on the scheduled visit day, followed by the mixed-meal test: the subject consumes instant noodles in the morning under fasting conditions, ensuring all noodles are eaten, with the first bite of noodles marking the 0 h time point, and the meal should be completed within 10 min. Venous blood samples are collected at 0.5 h (±5 min), 1 h (±5 min), and 2 h (±5 min) after the meal for the measurement of postprandial plasma glucose, postprandial serum insulin, and postprandial serum C-peptide.

The subject remains under fasting conditions from the end of the mixed-meal consumption until the 2 h postprandial blood collection.

8.2.5. Body weight and waist circumference under fasting conditions

Measurement of body weight under fasting conditions:

1) Perform the measurement in a uniform manner using a calibrated scale (either mechanical or electronic scales) and use the same weighing scale whenever possible. Each weight measurement of a subject should be performed under fasting conditions after the subject has emptied his/her bladder.
2) Before weighing, effort should be made to ensure that the pointer of the weighing scale is pointing at zero and that the weighing scale is placed on a stable and smooth surface;
3) After confirming that the subject has emptied his/her bladder, the subject will be asked to enter the weight measuring area, pull up the curtain (if any), take off the clothing and accessories that increase the weight (coat/outer pants/hat/scarf/necklace/belt, etc., to ensure that the subject does not feel cold after taking off the clothing), wear single clothes only, and take off the shoes.
4) Subjects are required to stand still in the center of the scale pedal, bear weight evenly on both legs, and place both arms on both sides of the body. Then, the body weight should be read in kg, and the value should be recorded and rounded to one decimal place.

Waist circumference measurement under fasting conditions

1) The subject is under fasting conditions, stands upright, breathes naturally and evenly, relaxed the abdomen, hangs both arms naturally down, keeps feet together, and bears weight evenly on both legs.
2) The measurement site between the lower edge of the costal arch and the iliac crest should be fully exposed, and the horizontal position of waist circumference measurement should be the midpoint of the line connecting the lower edge of the costal arch on the bilateral midaxillary line and the iliac crest.
3) A tape should be used to embrace the above horizontal position for one circle. During the measurement, the tape should be close to the skin (not separated by clothes), but it should not be too tight against the skin.
4) During measurement, there should be no intentional abdominal contraction or protrusion. Readings are taken at the end of a calm exhalation. The unit is cm. The value is recorded with one decimal place retained.

5) The mean value of 2 measurements should be taken as the measurement result. If the difference between the 2 measurements is more than 1 cm, the set of measurements will be discarded and additional 2 measurements should be performed.

During the study, measurements should be performed by the same designated person as much as possible.

Note: if a subject is not under fasting conditions, an appointment should be made within the visit time window for the subject to return to the hospital again for measurements of fasting body weight and waist circumference.

8.3. Safety Evaluation and Procedures

See Section 1.3 Schedule of Trial Activities for details.1.3

Safety evaluation includes collection of AEs (see Section 8.4), hypoglycemic episodes, laboratory assessments, vital signs, physical examination, 12-lead ECG, diabetic retinopathy screening and assessments.8.4

8.3.1. Vital signs and physical examination

Subjects will undergo physical examination including height, weight, and vital signs (including pulse, systolic blood pressure, and diastolic blood pressure). Height will be measured at screening only.

The subject's pulse, systolic blood pressure and diastolic blood pressure will be assessed while sitting. Measurements should be performed after 5 minutes of rest. Vital signs should be measured at least 10 hours after caffeine, alcohol, or nicotine intake.

For each blood pressure measurement, the same arm as that at screening is selected for measurement, and the measurement is taken in a sitting position with the elbow positioned at the same level as the heart.

Blood pressure and pulse are required to be measured three times, with an interval of at least one minute each time. The average is calculated based on the three measurements.

If the average of the systolic blood pressure is ≥159.5 mmHg, it will be rounded to 160 mmHg, making the subject not eligible for the study based on exclusion criteria (the same calculation applies to diastolic blood pressure).

Physical examination includes major body organs, referring to general conditions, skin, lymph nodes, head and neck, chest (including heart and lungs), abdomen, musculoskeletal system, and nervous system.

8.3.2. Laboratory Tests

Blood and urine samples involved in laboratory tests will be collected at the time points specified in the Schedule of Trial Activities in Section 1.3 and analyzed at the local/central laboratory. Laboratory tests are specified in Table 7.1.3

Table 7. Laboratory Test Items

**Routine Blood Test**	**Blood biochemistry**	**Urinalysis**
White blood cell count<br>Red blood cell count<br>Platelet count<br>Neutrophil count<br>Lymphocyte count<br>Hemoglobin<br>Hematocrit<br>Lymphocyte percentage<br>Neutrophil percentage<br>Monocyte percentage<br>Absolute eosinophil count<br>Absolute basophil count	Serum bilirubin total<br>Serum direct bilirubin<br>Serum alanine aminotransferase<br>Serum aspartate aminotransferase<br>Serum γ-glutamyl transpeptidase<br>Serum alkaline phosphatase<br>Serum albumin<br>Urea nitrogen or serum urea<br>Serum creatinine<br>eGFR<br>Uric acid<br>Serum calcium total<br>Potassium<br>Sodium<br>Serum chloride<br>Serum inorganic phosphorus	Urine pH<br>Urine protein<br>Urinary occult blood<br>Urine erythrocyte<br>Urine leukocytes<br>Urine glucose<br>Urine ketone body<br>Urine bilirubin<br>Urobilinogen
**Coagulation Function Test**	**Thyroid Function Test**	**Others**
Prothrombin time<br>Activated partial thromboplastin time<br>International normalized ratio<br>Thrombin time	Free triiodothyronine<br>Thyroxine, Free<br>Thyroid stimulating hormone	Serum amylase<br>Serum lipase<br>Serum calcitonin<br>Pregnancy Test<br>FSH<br>UACR<br>Triglycerides<br>Serum cholesterol total<br>Serum high density lipoprotein cholesterol<br>Serum low density lipoprotein cholesterol<br>Hepatitis B Virus Surface Antigen (if positive, HBV DNA will be additionally tested)

Abbreviations: eGFR = estimated glomerular filtration rate; FSH = follicle-stimulating hormone; HBV = hepatitis B virus.

8.3.3. 12-Lead ECG

12-lead ECG should be performed by a qualified trained physician at the time points specified in the Schedule of Trial Activities in Section 1.3.1.3

All scheduled electrocardiogram examinations should be performed in a supine position after the subject has been seated for at least 10 minutes. Electrocardiogram measurements will include heart rate, PR interval, RR interval, QT interval and QTcF. QTcF is calculated by Fridericia formula: QTcF = QT/(RR^0.33). When the electrocardiogram report does not

automatically provide RR interval results: if the subject has regular rhythm, the RR interval can be obtained by dividing 60 by the heart rate; if the subject has irregular rhythm, the RR interval will be provided by the physician or technician evaluating the electrocardiogram report results.

Additional electrocardiogram examination may be performed if clinically indicated.

8.3.4. Screening and Assessment of Diabetic Retinopathy

Baseline fundus photography should be performed for subjects who have successfully passed screening (including laboratory test results). A comprehensive ophthalmic evaluation of baseline fundus photography must be completed prior to randomization to confirm eligibility. Subjects with any degree of diabetic retinopathy or macular edema at baseline assessment, and who require treatment based on the clinical judgment of an ophthalmologist or medical personnel experienced in the evaluation of diabetic retinopathy and macular edema, will be excluded (see Exclusion Criterion 8).

All subjects need to undergo fundus photography according to the trial process table in Section 1.3. All subjects need to undergo fundus photography at screening, V12 (W32) and V15 (W52)/early withdrawal. If necessary, following the screening guidelines for diabetic retinopathy, the frequency of fundus photography may be increased based on the severity of retinal lesions or macular edema at baseline or during the study.1.3

Fundus photography should be performed on both eyes (unless assessment is not possible due to severe cataract, prosthesis, or other diseases), using the widest field of view available at the study site or by relevant qualified ophthalmic care professionals, such as: 2 fields with at least 60 degrees, or 4 wide fields, or 7 standard fields. Referral to an ophthalmologist or qualified medical personnel for assessment should be made whenever possible.

All fundus photographs will be evaluated by a central ophthalmologist or a medical professional experienced in the assessment of diabetic retinopathy and macular edema. The evaluation will include the presence of diabetic retinopathy and/or macular edema and whether treatment is recommended. Fundus photographs deemed unassessable for any reason will be retaken based on the ophthalmologist's recommendation. Throughout the study, all photographs of the subject should be reviewed by the same qualified assessor whenever possible. Any evidence of diabetic retinopathy, macular edema, or other findings of clinical significance on fundus photographs will require additional ophthalmological follow-up according to local standard treatment. Optical coherence tomography is recommended to confirm cases of macular edema.

The results of the comprehensive ophthalmic assessment, including the final diagnosis of non-proliferative retinopathy, proliferative retinopathy or diabetic macular edema, will be provided to the Investigator and recorded in the specific retinopathy eCRF. Any interventions initiated for diabetic retinopathy or macular edema during the study will be recorded in the concomitant medications eCRF.

8.4. Adverse Events (AEs) and Serious Adverse Events (SAEs)

8.4.1. Definition of AEs and SAEs

AE refers to any adverse medical event that occurs after a subject receives an Investigational Product, but it does not necessarily have a causal relationship with the treatment. An AE could therefore be any unfavorable and unintended sign (including an abnormal laboratory finding), symptom, or disease temporally associated with the use of a medicinal product, whether or not related to the medicinal product. AEs include but are not limited to:

1) Exacerbation of a pre-existing (before entering the clinical trial) medical condition/disease (including exacerbation of symptoms, signs, and laboratory test abnormalities);
2) Any new occurrence of AE: any adverse medical conditions newly occurred (including symptoms, signs, newly diagnosed diseases);
3) Clinically significant abnormal laboratory test results.

SAE refers to any adverse medical event that occurs in a subject after receiving any dosage of the investigational drug:

- Events leading to death;
- Life-threatening event (at risk of death at the time of the event);
- Events that require hospitalization (as defined in Section 12.2.1) or prolongation of hospitalization;12.2.1
- An event resulting in persistent or significant disability/incapacity (substantial disruption of the ability to conduct normal life functions);
- Congenital abnormalities or birth defects;
- Important Medical Event
  - Medical and scientific judgments must be used to decide whether it is an Important Medical Event. Important Medical Events may not be immediately life-threatening, result in death, or hospitalization. However, if the event may jeopardize the subject or require intervention to prevent one of the outcomes listed in the SAE definition above, the Important Medical Event should be reported as an SAE.

- Examples include allergic bronchospasm requiring emergency treatment in the emergency room or at home, non-hospitalized cachexia or convulsions, and drug dependence or drug abuse.

Further details and descriptions on AEs and SAEs are provided in Sections 12.1 and 12.2.12.112.2

8.4.2. Collection of AEs and SAEs

Clinical trial safety events, including adverse medical events, AEs/SAEs/AESIs, and pregnancy and drug overdose events prior to the first dose, will be collected from the signing of the ICF by the subjects until the end of the safety follow-up period (2 weeks after the last dose) (table 8).

Table 8. Collection of Clinical Trial Safety Events

Period	Collection Requirements
Starting from the subject's signing of Informed Consent Form until the end of the Safety follow-up phase*	Untoward medical events/AEs/SAEs prior to first dose: collected after signing the Informed Consent Form<br>Pregnancy event/drug overdose: from the first dose<br>AESI: collection started after randomization
After the aforementioned periods	SAEs and AESIs related to the Investigational Product

Abbreviations: AE = adverse event, AESI = adverse event of special interest, SAE = serious adverse event.
*The end of the safety follow-up phase refers to: 2 weeks after the last administration of the Investigational Product.

8.4.3. Identification of AEs and SAEs

At each study visit, the investigator should assess whether the subject has experienced AEs or SAEs by inquiring and conducting physical examinations or laboratory tests. All events, whether observed by the investigator or reported spontaneously by the subject, must be assessed and recorded by the investigator.

8.4.4. Recording of AEs and SAEs

All AEs should be recorded in detail in the AE page of the eCRF, including: event term, start and end dates, severity, seriousness, causality between the event and the Investigational Product, actions taken regarding the Investigational Product, and outcome. If the event meets the SAE criteria, it must be reported according to the SAE reporting procedure.

For more details on the assessment of the severity and causality of AEs and SAEs, please refer to Sections 12.3 and 12.4.12.312.4

8.4.5. Follow-up of AEs/SAEs

All AEs should be followed up until symptoms disappear, or clinically relevant changes in laboratory tests return to baseline and/or reach a stable state, or a reasonable explanation is obtained (such as lost to follow-up and death).

8.4.6. SAE Reporting System

In case of SAE, regardless of whether it is an initial report or a follow-up report, the investigator must immediately complete the "SAE/AESI/Drug Overdose Report Form", sign and date it, notify the sponsor within 24 hours of awareness, and report to relevant organizations in a timely manner as required by local regulations.

For SAEs that occur after the safety follow-up phase, those suspected to be related to the Investigational Product should be collected. SAEs should be documented in detail including symptoms, severity, correlation with the Investigational Product, time of onset, time of treatment, measures taken, follow-up time and method, and outcome. If the investigator considers an SAE unrelated to the Investigational Product but potentially related to study conditions (e.g., discontinuation of original treatment, or complications during the trial), this relationship should be thoroughly explained in the narrative section of the SAE report form.

The email address of the sponsor for receiving reports of SAEs (as well as AESIs, pregnancy and drug overdose) for this project is: hengrui_drug_safety@hengrui.com

8.4.7. Regulatory Reporting Requirements for SAEs

Except for those SAEs explicitly exempted from immediate reporting per the protocol, the investigator must submit a written report of all SAEs to the sponsor within 24 h of becoming aware of the event. A detailed written follow-up report must be submitted in a timely manner thereafter.

For reports involving fatal events, the Investigator shall provide the Sponsor and Institutional Review Board with other required materials, such as autopsy reports and final medical reports.

8.4.8. Suspected Unexpected Serious Adverse Reaction Report

Suspected Unexpected Serious Adverse Reaction refers to a serious adverse reaction whose nature and severity of clinical manifestations are beyond the existing information in the Investigator's Brochure (IB), package insert or Summary of Product Characteristics (SmPC) of the study drug or marketed medicinal product.

After the Sponsor receives safety-related information from any source, it shall immediately analyze and evaluate it, including seriousness, correlation with the study drug, and whether it

is an expected event. The Sponsor shall quickly report Suspected Unexpected Serious Adverse Reactions to the drug regulatory authority, the competent health authority and all Investigators participating in the Clinical trial, and submit them to the Clinical trial institution and Institutional Review Board according to the requirements of the Study site.

8.4.9. Adverse events of special interest (AESI)

For the reporting of AESIs specified in the clinical study protocol, "SAE/AESI/Drug Overdose Report Form" must be filled out within 24 h of the investigator's first knowledge of the event and reported to the sponsor. This procedure applies to all AESIs regardless of whether they meet the SAE criteria. The reporting procedure for AESIs is the same as that for SAEs. If they meet the SAE definition, they must also be reported according to the SAE reporting process.

AESIs in this study include:

- Liver function test abnormal;
- Grade 3 hypoglycemia: see Section 8.4.9.2 for specific definition;8.4.9.2
- Calcitonin abnormal: calcitonin level ≥50 ng/L after treatment administration, and the specific value increases by > 50% from baseline;
- Acute pancreatitis

8.4.9.1. Abnormal Liver Function Tests

AST and/or ALT abnormalities accompanied by abnormally increased TBIL that meet the following criteria (1), (2) and (3) with no other reasons should always be reported as AESIs.

Table 9. Criteria for Determining Abnormal Liver Function Tests

Condition Met	Determination Criteria
1) ALT or AST abnormalities	Normal ALT and AST at baseline: ALT or AST > 3 × ULN during the treatment period;<br>Abnormal ALT or AST at baseline: ALT or AST > 2 × baseline level during the treatment period, and > 3 × ULN; or > 8 × ULN (whichever is lower).
2) TBIL abnormality	Normal TBIL at baseline: TBIL > 2 × ULN during the treatment period;<br>Abnormal TBIL at baseline: TBIL increases by > 1 × ULN during the treatment period or its value is > 3 × ULN (whichever is lower).
(3) No evidence of cholestasis (e.g., elevated serum ALP due to gallbladder or bile duct disease, etc.)	

Abbreviations: ALP=alkaline phosphatase, ALT=alanine aminotransferase, AST=aspartate aminotransferase, TBIL=total bilirubin, ULN=upper limit of normal.

If a subject has abnormal AST and/or ALT levels and abnormally increased TBIL levels during the treatment period or follow-up period, the subject must return to the study site for investigation and assessment as soon as possible (preferably within 48 h) after learning about

the abnormal results. Examination and assessment should include relevant laboratory tests of liver function, thorough inquiry of medical history and physical examination.

In addition to repeat testing of AST and ALT, liver function tests may include albumin, creatine kinase, TBIL, direct and indirect bilirubin, gamma-glutamyl transferase, prothrombin time/international normalized ratio, and alkaline phosphatase. Collection of detailed medical history and relevant information is recommended to include the history of alcohol use, acetaminophen, soft drugs, various supplements, family medical history, history of sexual behavior, travel history, history of contact with patients with jaundice, surgical history, history of blood transfusion, hepatopathy and allergic diseases, or possible occupational chemical exposure. Further examinations may also include tests on acute infections of hepatitis A, B, C, D and E, as well as imaging examination of the liver (such as the biliary tract).

8.4.9.2. Hypoglycemic episodes

8.4.9.2.1. Definition and Classification of Hypoglycemic Episodes

Hypoglycemic episodes are classified according to the 2024 American Diabetes Association (ADA) Standards of Medical Care in Diabetes and the 2020 Chinese Diabetes Society Guidelines for the Prevention and Treatment of Type 2 Diabetes in China (table 10).[6]

Table 10. 2024 ADA-Recommended Classification of Hypoglycemic Episodes

Hypoglycemia Level	Definition
Grade 1	Blood glucose < 3.9 mmol/L and ≥3.0 mmol/L
Grade 2	Blood glucose < 3.0 mmol/L
Grade 3	No specific glucose threshold; hypoglycemia associated with severe cognitive and/or physical impairment requiring external assistance for recovery

Abbreviations: ADA = American Diabetes Association.

Clinical manifestations of hypoglycemia (related to blood glucose levels and glucose-lowering rate) may include: sympathetic nerve excitement (e.g., palpitations, anxiety, sweating, hunger, etc.) and central nervous system symptoms (e.g., altered consciousness, cognitive impairment, convulsions, and coma). When hypoglycemia is suspected, the subject should measure and record glucose levels as soon as possible using the study-provided glucometer.

8.4.9.2.2. Recording and Reporting of Hypoglycemic Episodes

Grade 3 hypoglycemia is defined as AESI.

Hypoglycemic episodes meeting the definition of SAEs or AESIs should be recorded on both the AE page and hypoglycemic episode page of the eCRF, and reported following the procedure for SAE or AESI. All other hypoglycemic episodes are recorded on the

hypoglycemic episode page only, including general information of hypoglycemia (time, SMBG measurement results, symptoms, treatment, possible causes, etc.).

During the study, the subject should record hypoglycemia events in the subject diary.

If blood glucose values are not available at the time of the suspected hypoglycemic episode based on clinical manifestations, the event, i.e., a possible symptomatic hypoglycemic episode, should still be recorded.

8.5. Pregnancy

If a female subject becomes pregnant during the clinical trial, the subject must discontinue the trial intervention; if the partner of a male subject becomes pregnant during the clinical trial, the subject can continue the clinical study. The investigator should fill out the "Pregnancy Report Form" within 24 hours after becoming aware of the pregnancy event to report it to the sponsor, and report to relevant authorities as required by local regulations.

The investigator is required to follow-up on the pregnancy event until the final outcome (including any early termination of pregnancy or delivery), which, in the case of delivery, would be 1 month thereafter, and report the pregnancy outcome to the sponsor or to other relevant authorities according to local requirements. If the pregnancy outcome meets the SAE criteria (such as ectopic pregnancy, spontaneous abortion, intrauterine fetal demise, neonatal death, congenital anomaly, etc.), the investigator should report it according to the SAE procedures.

If a subject experiences any SAE during pregnancy, the SAE should be reported according to the SAE reporting procedure.

8.6. Pharmacokinetics

PK blood samples of the subject will be collected at V4, V6, V7, V8, V9, V10, V11, V12, and V15 as shown in the trial flow chart in Section 1.3. The time of the last dose before blood collection must be accurately recorded, and the plasma concentration of HRS-7535 will be measured.1.3

For subjects with unexpected AEs and/or SAEs, additional blood samples may be collected beyond the planned samples, and the sampling date and specific time, along with the last dosing before sampling, must be recorded in eCRF.

Every effort should be made to ensure that the pharmacokinetic samples are taken according to the planned relative time to administration. The exact sampling times will be indicated in full in the eCRF. If sampling cannot be completed as scheduled or sampling time is missed, the

sampling may be performed at another time after a consensus is reached among Investigator, subject and Sponsor.

PK samples of the study drug will be analyzed using a validated method. The study manual will provide information on the collection, processing, storage and transportation of blood samples.

8.7. Genetics (Not Applicable)

Not applicable.

8.8. Biomarkers (Not Applicable)

Not applicable.

8.9. Immunogenicity (Not applicable)

Not applicable.

8.10. Medical Resource Utilization and Health Economics (Not Applicable)

Not applicable.

9. Statistical Considerations

This section provides an overview of the study data collection and analysis methods as detailed in the Statistical Analysis Plan (SAP) and maintained by the sponsor.

9.1. Analysis Population

Intention-to-treat (ITT) analysis set: this population includes all randomized subjects. Subjects will be analyzed according to the group they are randomly assigned to. ITT is the primary analysis population for efficacy.

Safety set (SS): includes all subjects who received at least one dose of the Investigational Product after randomization. Subjects will be analyzed according to the group they were randomly assigned to.

PK concentration analysis set (PKCS): all subjects who have been randomized to receive HRS-7535 treatment and have at least one evaluable PK concentration data post-dose.

9.2. Supportive analysis for primary objective

9.2.1. Statistical Hypothesis and Decision Rules

This study is designed as a superiority trial, during which each HRS-7535 dose group will be compared with the placebo group for superiority testing on the primary efficacy endpoint and

key secondary endpoints. The corresponding alternative hypotheses (Hxx, where the first subscript 1, 2, 3 represents the HRS-7535 90 mg, 60 mg, and 30 mg groups respectively; the second subscript 1-5 represents different endpoints) are as follows:

- H11, H21, H31: superiority of HRS-7535 90 mg, 60 mg, and 30 mg groups vs. placebo group in HbA1c change from baseline after 32 weeks of treatment;
- H12, H22, H32: superiority in the proportion of subjects with HbA1c< 7.0% after 32 weeks of treatment in the HRS-7535 90 mg, 60 mg, 30 mg groups vs. the placebo group;
- H13, H23, H33: superiority of the proportion of subjects with HbA1c ≤6.5% at 32 weeks post-treatment in the HRS-7535 90 mg, 60 mg, 30 mg groups vs. placebo group;
- H14, H24, H34: superiority of FPG change from baseline at 32 weeks post-treatment for the HRS-7535 90 mg, 60 mg, and 30 mg groups vs. placebo group;
- H15, H25, H35: the superiority of the percentage change in body weight from baseline at Week 32 of treatment for HRS-7535 90 mg, 60 mg, and 30 mg groups versus placebo group.

The sequential testing method sequentially tests the following hypotheses at a significance level of 0.025: H11, H21, H31, H12, H22, H32, H13, H23, H33, H14, H24, H34, H15, H25, and H35. If the one-sided p-value of a test is greater than 0.025, the testing is discontinued.

9.2.2. Primary Estimand Analysis

For the primary estimand, the primary study endpoint will be analyzed based on the ITT set. HbA1c is analyzed using analysis of covariance (ANCOVA) model with treatment, stratification factors (prior use of glucose-lowering agents [yes, no]) as fixed effects, and baseline HbA1c as covariate. The least squares mean, standard error, 95% confidence interval (CI) of the change from baseline in HbA1c at week 32 for each group and between groups, as well as the p-value for the difference between each dosage group of HRS-7535 and the placebo group will be estimated and listed.

A treatment-policy strategy is applied for all intercurrent events, data after intercurrent events will be included in the analysis. Missing data due to intercurrent events will be imputed using the RDO (retrieved Dropout) method. If the data do not support the RDO method, the CR (copy Reference) method will be used for imputation. Missing data unrelated to intercurrent events are assumed to be missing at random.

A sensitivity analysis will be performed on the primary estimand. See the SAP for the detailed method.

9.2.3. Supplementary Estimand Analysis

For the supplementary estimand, the primary study endpoint will be analyzed based on the ITT set. It will be analyzed using a mixed-effect model repeated measure (MMRM) based on restricted maximum likelihood (REML). The model includes stratification factors (previous use of glucose-lowering agents: yes, no), treatment, visit, and the interaction between treatment and visit as fixed effects, baseline HbA1c as the covariate, and subject as the random effect. The UN or alternative CS covariance matrix is employed. The least squares mean, standard error, 95% CI of the change from baseline in HbA1c at week 32 for each group and between groups, as well as the p-value for the difference between each dosage group of HRS-7535 and the placebo group, are estimated and listed. Hypothetical strategy is applied for all intercurrent events, and data after intercurrent events are not included in the analysis.

9.3. Analysis supporting secondary objectives

9.3.1. Analysis of secondary efficacy endpoints

The secondary efficacy endpoints analysis will be based on ITT, with intercurrent events and handling strategies consistent with the primary estimand. For dichotomous endpoints (response/no response) at Week 32 of treatment: the proportion of subjects achieving HbA1c< 7.0%, HbA1c≤6.5% and HbA1c < 5.7%, generalized estimating equations (GEE) will be used for analysis if applicable. For continuous endpoints at Week 32 of treatment: change from baseline in FPG; percentage change from baseline in body weight; change from baseline in body weight and waist circumference; change from baseline in fasting serum insulin, fasting serum C-peptide, HOMA-β, HOMA-IR; change from baseline in 1-hour postprandial plasma glucose, 2-hour postprandial plasma glucose, 2-hour postprandial serum insulin, 2-hour postprandial serum C-peptide, disposition index (DI); change from baseline in 7-point SMBG profile; for endpoints with two or more repeated measurements after randomization, MMRM will be used for analysis if applicable. For endpoints with only one measurement after randomization, ANCOVA will be used for analysis if applicable.

Descriptive summary analysis will be performed for all secondary efficacy endpoints at Week 52 of treatment.

9.3.2. Analysis of Other Secondary Endpoints

Based on ITT, the change from baseline in DTSQs at Week 32 will be analyzed using the ANCOVA method. The change from baseline in DTSQs at Week 52 will be summarized descriptively.

9.4. Analyses to Support Exploratory Objectives

Analysis of exploratory endpoints will be based on ITT.

For continuous endpoints with normal distribution and two or more repeated measurements after randomization, MMRM will be used for analysis where applicable. Specific analysis methods are detailed in the SAP. Exploratory endpoints will be summarized descriptively.

9.5. Safety Analysis

Safety analysis will be performed based on the SS.

AEs are coded according to the Medical Dictionary for Regulatory Activities (MedDRA). All AEs are listed with their severity and relationship to the Investigational Product. The number and percentage of subjects with the following TEAEs are summarized:

- TEAE
- Treatment-Related TEAE
- With TEAEs Leading to Treatment Discontinuation
- AESI
- SAE

AEs, hypoglycemic events, vital signs, physical examinations, laboratory tests, 12-lead ECG, diabetic retinopathy screening and evaluation will be summarized using descriptive statistics and listed.

9.6. Other Analyses

9.6.1. Pharmacokinetic analysis

Descriptive statistical analysis will be performed on the plasma drug concentration by dose group and Visit based on PKCS.

9.6.2. Subgroup analysis

Subgroup analyses will be performed on the primary efficacy endpoints including but not limited to the following, with the same analytical methods as the primary estimand analysis:

- Baseline HbA1c (≤8.5% versus > 8.5%);
- Gender (males versus females);
- Baseline BMI (≥24 kg/m2 vs. < 24 kg/m2);
- Previous use of glucose-lowering agents (yes vs. no);
- Duration of type 2 diabetes (≥median [months] vs. <median [months]).

Refer to the SAP for details on other subgroup analysis.

9.7. Interim analysis (not Applicable)

Not applicable.

9.8. Sample size determination

The sample size calculation is based on the primary endpoint, i.e., the change in HbA1c from baseline to Week 32. Based on the results of the Phase II study, assuming that the efficacy difference in HbA1c between the HRS-7535 dosage groups and the placebo group is -0.85% for subjects without intercurrent events and 0 for subjects with intercurrent events, the overall efficacy is -0.72% (considering a intercurrent events incidence of 15%), with a standard deviation (SD) of 1.1%. A total of 272 subjects (68 per group) will be randomly assigned in a 1:1:1:1 ratio to the HRS-7535 30 mg group, 60 mg group, 90 mg group, and placebo group, providing an overall power of 90% to detect the superiority of all three dosage groups of HRS-7535 over the placebo group at a one-sided significance level of 0.025.

9.9. Protocol deviations

A protocol deviation refers to any change and non-compliance with the clinical study protocol design and relevant process documents involved in the protocol. Different protocol deviations have different effects on the rights and interests of subjects and on the integrity, accuracy and reliability of trial data. Protocol deviations may come from subjects, investigators, or study site staff. Depending on the root cause of the deviation, corrective and preventive actions need to be formulated and implemented promptly.

10. GENERAL CONSIDERATIONS: REGULATORY, ETHICAL, AND STUDY OVERSIGHT

10.1. Regulatory and Ethical Considerations

This clinical trial must be conducted in compliance with the Protocol, ethical principles of the Declaration of Helsinki, current GCP and relevant laws and regulations. Before the trial starts, the Protocol and its amendments, ICF and other relevant documents (such as recruitment materials) should be provided to the Institutional Review Board. This trial must be approved by the relevant regulatory authorities and the Institutional Review Board of the hospital before it can be implemented.

Neither the sponsor nor the investigator shall unilaterally amend the protocol without the consent of both parties. Any amendment to the protocol must be reviewed and approved by the institutional review board before implementation. If necessary, other relevant documents

should be revised accordingly and submitted and/or approved as required by the institutional review board.

If the investigator has to initiate a process that deviates from the protocol to eliminate significant and direct harm to a subject, the Institutional Review Board and the sponsor must be notified in writing immediately after the deviation is implemented, along with an explanation and documentation of any protocol deviations made. In addition, the investigator is responsible for submitting the interim reports of the study on a regular basis in accordance with the relevant requirements of the Institutional Review Board and shall notify the Institutional Review Board of the completion of the study.

10.2. Committees (Not Applicable)

No external Data Monitoring Committee (DMC) or Safety Monitoring Committee (SMC) is established for this trial.

10.3. Informed consent process

Informed consent process: informed consent begins before a subject agrees to participate in the clinical trial and continues throughout the entire clinical trial process. The investigator or designated research personnel must provide the latest version of the Informed Consent Form approved by the Institutional Review Board, discuss in detail and fully with the subject or his/her guardian the risks and potential benefits of participating in the trial, introduce the details of the trial, and answer any related questions raised by the subject or his/her guardian. Subjects may only start participating in the trial after providing written informed consent. During the entire clinical trial process, subjects may voluntarily withdraw from the trial at any time. One copy of the Informed Consent Form will be kept by the subject. Even if subjects refuse to participate in this trial, their rights and interests will be fully protected, and the quality of their medical care will not be affected in any way.

Requirements for re-screening informed consent: subjects who have previously experienced screen failure (as defined in Section 5.6) are allowed to be re-screened, with each subject permitted only one re-screening throughout the trial. During re-screening, the subject must re-sign the informed consent form and re-register to obtain a new subject number.5.6

10.4. Data Protection

10.4.1. Confidentiality of Subject Information

The subject information will be kept strictly confidential by the investigator, participating study personnel, sponsor and its agency. Confidentiality also covers biological samples and genetic

tests in addition to subjects' clinical information. Therefore, the trial protocol, documentation, data and all other information generated therefrom will be kept strictly confidential. Without the prior written approval of the sponsor, all information on trial or data should not be disclosed to any unauthorized third party.

All trial procedures will be performed as confidentially as possible.

The Monitor and other authorized representatives of the Sponsor, Institutional Review Board, and drug regulatory authority may inspect all documents and records that the Investigator is required to maintain, including but not limited to: medical records and medication records of subjects. The Study site shall permit access to these records.

The subject’s contact information will be stored at each study center safely and only provided for internal use during the study. At the end of the trial, all records will continue to be stored in a secure place in accordance with the time limit specified by the local Institutional Review Board and regulations.

10.4.2. Data Protection

All parties will comply with all applicable laws, including those related to the implementation of institutional and technical measures to ensure the protection of subject data.

Subjects’ personal data will be stored at the study site in encrypted electronic and/or paper form, password protected or in a locked storage room to ensure that only authorized study staff have access. The study site will take appropriate technical and organizational measures to ensure that personal data can be restored in the event of a disaster. In the event of a potential personal data breach, the site will be responsible for determining if a personal data breach has occurred and, if so, for providing a breach notification as required by law.

To protect the rights and freedoms of the subjects with respect to data processing, subjects will be assigned a separate specific identification number. Any subject records or datasets transferred to the sponsor will contain this identification number; the subject’s first name will not be transferred. All other identifiable data transferred to the sponsor will be replaced by this separate specific identification number. The study site will maintain a confidential list of subjects participating in the trial that can link each subject’s identification number to their actual identity and medical records. During the data transfer process, the sponsor will protect the confidentiality of the subjects’ personal data in accordance with the trial protocol and applicable privacy laws.

10.5. Early termination or suspension of trial

This trial may be terminated or suspended early if justified. This may be due to the decision of the drug regulatory authority, changes in the comments of the Institutional Review Board, issues with the efficacy or safety of the study drug, or the judgment of the sponsor. In addition, the sponsor reserves the right to stop the development of HRS-7535 at any time.

If the sponsor terminates or suspends the trial prematurely, it shall immediately inform the investigator, the clinical trial institution and the drug regulatory authority, and explain the reasons. Upon receipt of the notification, the investigator shall promptly notify the subjects and give the subjects appropriate treatment and follow-up. In addition, the investigator shall immediately report to the clinical trial institution and the Institutional Review Board, and provide detailed written explanation.

Reason(s) for premature discontinuation or suspension of the trial may include the following:

- Confirmed unexpected, major, or unacceptable risks to the subjects;
- The investigator fails to comply with the protocol, the requirements of the ethics committee or drug regulatory authority, the sponsor's procedures, or the current GCP;
- Other situations, such as enrollment difficulties, no longer complying with medical practice, changes in the sponsor's research and development strategy.

The trial may continue once the drug safety, protocol compliance, and data quality issues, etc. that caused the suspension of the trial are resolved, and the approval of the sponsor, Institutional Review Board, or the drug regulatory authority is obtained.

11. General Considerations: Risk Management and Quality Assurance

11.1. Data Quality Assurance

In order to ensure the quality of the trial, the sponsor and the investigator will jointly discuss and formulate the clinical trial plan before the official start of the trial, and ensure that the study staff participating in the implementation of the trial have received appropriate training on GCP. Each study center shall manage the Investigational Product in accordance with the Standard Operating Procedures, including receipt, storage, dispensing, recovery and destruction.

According to the GCP guidelines, necessary procedures should be taken during the design and implementation stages of the trial to ensure that the collected data are accurate, consistent, complete and credible. All observed results and abnormal findings in the clinical trial must be verified and recorded in a timely manner to ensure data reliability. The instruments, equipment, reagents, standards, etc. used in various assessment items in clinical trials should have strict quality standards and ensure that they work under normal conditions.

The sponsor or designee will conduct monitoring to confirm that the data completed in the eCRF by the study site staff are complete, accurate, and traceable to ensure that the safety and rights of the subjects are protected and that the trial is conducted in accordance with the protocol, the GCP and all relevant laws and regulations. The investigator must ensure the accuracy of the source documents (source data) for the information entered into the eCRF.

The regulatory agencies, Institutional Review Board, Sponsor's Monitors and/or auditors may systematically examine clinical trial-related activities and documents to evaluate whether the trial is conducted in accordance with the requirements of the trial protocol, SOP and relevant regulations, and whether the trial data is recorded in a timely, truthful, accurate and complete manner. The audit should be performed by personnel not directly involved in this clinical trial.

The monitoring plan will provide detailed monitoring strategies (e.g., risk-based operational and quality management measures, such as risk management and mitigation strategies as well as analysis risk-based controls), methods, responsibilities and requirements, including handling of non-compliance issues and monitoring techniques (site-level, remote or on-site monitoring).

The sponsor or its designated personnel is responsible for the data management of this study, including data quality checks.

The sponsor is responsible for actions delegated to other individuals (e.g., contract research organization).

11.2. Source Data

The source documents provide evidence for the subjects' participation in the clinical study and substantiate the integrity of data collected. Source documents will be archived at the investigator's institution.

The data in the eCRF must be consistent with the source documents, or the discrepancy must be reasonably explained. Depending on the needs of the trial, the investigator may require previous medical records or transcribed records. In addition, current medical records must be provided.

The Sponsor's data monitoring plan defines the composition and source of source data.

The Sponsor's data management plan defines the usage instructions for the electronic information system.

The Investigator must ensure that the data entered in the eCRF can be traced back to the source records, and archive the original documents.

12. APPENDIX: ADVERSE EVENTS AND SERIOUS ADVERSE EVENTS - DEFINITIONS, SEVERITY, AND CAUSALITY

12.1. Definition and Supplement of AE

12.1.1. Drug Overdose

Drug overdose is defined as an intentional or unintentional dose of HRS-7535/placebo received by the subject, either actively or passively, at a dosage greater than 180 mg/day.

In this study, when a drug overdose event occurs, the Investigator shall complete the "SAE/AESI/Drug Overdose Report Form," sign and date it, and notify the Sponsor within 5 calendar days of becoming aware of the event.

If the event of drug overdose meets the SAE criteria, it must be reported according to the SAE reporting procedure.

12.2. Description and Supplement of SAE Definition

12.2.1. Hospitalization

Hospitalization is defined as any first admission (even if less than 24 hours) or prolongation of hospitalization. Hospitalization does not include the following:

- Rehabilitation facility;
- Sanatorium;
- Routine admission to the emergency department (less than 24 h);
- Same-day surgery (such as outpatient/same-day/ambulatory surgery).

Hospitalization or prolonged hospitalization unrelated to AE is not considered an SAE. For example, the following are not considered SAEs:

- Admission for treatment of a preexisting condition not associated with the development of a new AE or with a worsening of the preexisting condition (e.g., for workup of a persistent pretreatment laboratory abnormality);
- Hospitalization for administrative reasons (e.g., annual physical examination);
- Protocol-specified admission during the clinical trial (e.g., for a procedure required by the study protocol);
- Elective hospitalization unrelated to AE (e.g., elective cosmetic surgery);
- Scheduled treatment or surgical procedure (should be documented throughout the entire study protocol and/or in subjects' individual baseline information);
- Admission exclusively for the administration of blood products.

Diagnostic or therapeutic invasive (such as surgery) and non-invasive procedures should not be reported as AEs, but it should be reported when the disease condition leading to this procedure meets the definition of AE. Acute appendicitis that develops during the AE reporting period and meets the AE requirements should be reported as an AE, and the resulting appendectomy should be recorded as treatment of the AE.

12.3. Severity

When filling out the AE form in the eCRF, the investigator will use mild, moderate, and severe to describe the severity of AEs. To standardize the criteria, the severity of AEs is graded as follows:

- **Mild:** usually transient, generally does not affect the subject's activities of daily living, and may only require minimal treatment;
- **Moderate:** the subject experiences discomfort and disruption of activities of daily living that usually require treatment to alleviate, but do not pose a risk of significant or permanent harm to the subject;
- **Severe:** significantly impacts the subject's daily activities or severely affects the clinical condition, requiring intensive treatment and intervention.

Pay attention to distinguish between the severity and seriousness of AEs. The mild, moderate, and severe grades above are only used to describe the severity of AEs. For example, headache might be manifested as severe in severity, but it should not be listed as an SAE unless it meets the SAE criteria.

12.4. Causality

The causality assessment between an AE and the Investigational Product refers to the comprehensive evaluation to determine whether there is a reasonable possibility that the Investigational Product caused or contributed to the AE. The factors for judgment include whether the occurrence of the AE has a reasonable temporal relationship with the administration of the Investigational Product, the characteristics of the Investigational Product, the toxicological and pharmacological effects of the Investigational Product, the use of concomitant medications, the subject's underlying disease, medical history, family history, dechallenge and rechallenge reactions, etc. Facts (evidence) or arguments demonstrating the causality should usually be provided.

The investigator should assess the causality between AEs and the Investigational Product as "definitely related", "possibly related", "unlikely related", "not related", and "unassessable".

13. APPENDIX: DEFINITIONS AND SUPPORTING OPERATIONAL DETAILS

13.1. Contraception and Pregnancy Test

13.1.1. Definitions related to fertility

Female subjects are not required to use contraception if they are considered infertile in any of the following circumstances:

- Postmenopausal status is defined as women aged over 45 with natural menopause for at least 12 months without any other physiological or medical cause. Follicle Stimulating Hormone (FSH) levels in the postmenopausal range in women without hormonal contraception or hormonal replacement therapy may be used to confirm postmenopausal status. However, if menopause is less than 12 months, FSH alone at postmenopausal levels is not sufficient to confirm postmenopausal status.
- Hysterectomy and/or bilateral oophorectomy, bilateral salpingectomy;
- Infertility caused by congenital or acquired conditions (e.g., uterine aplasia, Turner syndrome, ovarian failure, etc. as confirmed by a gynecologist).

13.1.2. Contraception

Male subjects with azoospermia (caused by vasectomy or other potential diseases) are required to use condoms and their partners do not need to take contraceptive measures.

Female subjects are not required to use contraception if they are not of childbearing potential (see Section 13.1.1).13.1.1

Female subjects of childbearing potential and male subjects whose partners are women of childbearing potential must agree to use contraception, refrain from sperm/egg donation from the signing of the ICF to 2 weeks after the last dose of the Investigational Product, and comply with one of the following requirements:

- Abstinence (avoid heterosexual intercourse).*

or

- During heterosexual intercourse, female subjects (or partners of male subjects) must use a highly effective method of contraception, while male subjects must use a condom concurrently.

The reliability of sexual abstinence needs to be assessed based on the duration of the clinical trial and the subject's personal preference and lifestyle. For those who choose to completely not have a sexual life, abstinence (avoiding heterosexual intercourse) may be used alone for contraception. Periodic abstinence (e.g., based on ovulation, symptomatic body temperature, etc.), use of spermicides only, lactational menopause, and withdrawal are not acceptable methods of contraception.

Highly effective contraceptive methods are those that result in a failure rate of less than 1% per year when used consistently and correctly and include the following:

- Contraceptive pills (containing estrogen/progestin or progestogen) proven to be effective, administered orally, by injection, intravaginally, implantably, or transdermally;
- Properly placed intrauterine device (IUD) or effective intrauterine hormone-releasing system (IUS);
- Medically confirmed vasectomy of a female subject's male partner. For female subjects, the male partner should be their only sexual partner.
- Bilateral tubal ligation or bilateral tubal occlusion (the occlusion surgery has been proven effective by related instruments).

In addition, subjects need to know: the investigator needs to be notified as soon as the chosen contraceptive method is discontinued, or as soon as the subject or the subject's partner suspects or confirms a pregnancy.

13.1.3. Pregnancy test and FSH test

In this study, the pregnancy test and FSH testing rules are as follows:

- Female subjects aged 45 years or younger must complete pregnancy tests as specified in the protocol, with 2 serum pregnancy tests performed during the screening phase (V1) and before randomization (V3). After a negative pregnancy test result during the screening phase (V1), appropriate contraceptive measures should be initiated, and the run-in medication may be administered. A serum pregnancy test will be repeated before randomization (V3), and the Investigational Product will be administered only after confirmation of a negative result. Pregnancy testing will also be routinely conducted during the treatment period or upon early withdrawal, or additionally performed when a menstrual cycle is missed or when signs of pregnancy are suspected. Additional pregnancy testing will be performed when required by the Institutional Review Board or local regulations. If a positive pregnancy result is confirmed, the female subject will discontinue the Investigational Product but may remain in the study.

- For postmenopausal female subjects aged > 45 years and ≤65 years, pregnancy test and FSH test are required for confirmation. If the FSH results indicate postmenopausal level (FSH> 40 mIU/mL), these female subjects may be considered of non-childbearing potential, and no pregnancy test is required in subsequent follow-up; if the FSH results indicate non-postmenopausal level (FSH≤40 mIU/mL), pregnancy test is still required during the trial.
- Menopausal women over 65 years old, deemed infertile by the investigator, are not required to undergo pregnancy tests and FSH testing.

13.2. Laboratory Tests

HbA1c, fasting FPG, fasting serum insulin, fasting serum C-peptide, postprandial plasma glucose, postprandial serum insulin, postprandial serum C-peptide, UACR, and PK blood sampling need to be sent to the central laboratory for testing according to the Visit schedule.

Lipase and calcitonin are sent to the central laboratory for testing according to the local laboratory's selection.

13.3. Country-Specific Variations (not Applicable)

Not applicable.

13.4. Protocol Amendment History

This study protocol is version 2.0. The revision information of the current version is summarized in "Protocol Revision History and Current Version Revision"

13.5. CKD-EPI Formula

CKD-EPI formula estimation:

$$\text{eGFR}=a \times \left(\frac{\text{Blood creatinine(mg/dL)}}{b}\right)^{c} \times 0.993^{\text{Age}};$$

Gender	a	b	c	
Female	144	0.7	-0.329 (creatinine ≤0.7 mg/dL)	-1.209 (serum creatinine > 0.7 mg/dL)
male	141	0.9	-0.411 (creatinine ≤0.9 mg/dL)	-1.209 (serum creatinine > 0.9 mg/dL)

Blood creatinine: 1 mg/dL = 88.4 μmol/L

Note: eGFR results are rounded down to the nearest whole number.

References:

Levey AS, Stevens LA, Schmid CH, Zhang Y, Castro III AF, Feldman HI, Kusek JW, Eggers P, Van Lente F, Greene T, Coresh J. A new equation to estimate glomerular filtration rate. Annals of internal medicine. 2009 May 5;150(9):604-12.

14. APPENDIX: ABBREVIATIONS

ABBREVIATION	Full English Name
ADA	American Diabetes Association
AE	Adverse Event
AESI	Adverse Event of Special Interest
ALP	Alkaline Phosphatase
ALT	Alanine Aminotransferase
ANCOVA	Analysis of Covariance
AST	Aspartate Aminotransferase
AUC	Area Under the Plasma Concentration-Time Curve
BMI	Body Mass Index
CI	Confidence Interval
CKD-EPI	Chronic Kidney Disease Epidemiology Collaboration
Cmax	Maximum Plasma Concentration
CONSORT	Consolidated Standards of Reporting Trials
CRA	Clinical Research Associate
CS	Compound Symmetry
DMC	Data Monitoring Committee
DPP-4	Dipeptidyl Peptidase-4
DTSQ	Diabetes Treatment Satisfaction Questionnaire
eCRF	Electronic Case Report Form
eGFR	Estimated Glomerular Filtration Rate
FPG	Fasting Plasma Glucose
FSFV	First Subject First Visit
FSH	Follicle-Stimulating Hormone
GCP	Good Clinical Practice
GEE	Generalized Estimating Equation
GLP-1	Glucagon-Like Peptide-1
HbA1c	Glycated Hemoglobin
HBsAg	Hepatitis B Surface Antigen
HBV	Hepatitis B Virus
HDL-C	High Density Lipoprotein - Cholesterol
HOMA-β	Homeostasis Model Assessment of β-cell Function
HOMA-IR	Homeostasis Model Assessment of Insulin Resistance
IB	Investigator's Brochure
ICF	Informed Consent Form
ITT	Intention-to-treat
IUD	Intrauterine Device
IUS	Intrauterine System
IWRS	Interactive Web Response System
LDH	Lactate Dehydrogenase
LDL-C	Low-Density Lipoprotein - Cholesterol
LOCF	Last Observation Carried Forward
LSLV	Last Subject Last Visit
LYMP	Lymphocyte Count
MedDRA	Medical Dictionary for Regulatory Activities
MEN2	Multiple Endocrine Neoplasia Type 2
MMRM	Mixed Model of Repeated Measurement
MRHD	Maximum Recommended Human Dose
MTC	Medullary Thyroid Cancer
MTD	Maximum Tolerated Dose
NOAEL	No Observed Adverse Effect Level
NYHA	New York Heart Association
PopPK	Population Pharmacokinetics

ABBREVIATION	Full English Name
PK	Pharmacokinetics
PKCS	Pharmacokinetics Concentration Set
QD	Quaque Die (Once Daily)
REML	Restricted Maximum Likelihood
RET	Reticulocyte Count
SAE	Serious Adverse Event
SAP	Statistical Analysis Plan
SD	Standard Deviation
SGLT2	Sodium-Glucose Transport Protein 2
SMBG	Self-Monitoring of Blood Glucose
SMC	Safety Monitoring Committee
SOP	Standard Operation Procedure
SS	Safety Set
TBIL	Total Bilirubin
TC	Total Cholesterol
TEAE	Treatment-Emergent Adverse Event
TG	Triglyceride
UACR	Urine Albumin-to-Creatinine Ratio
ULN	Upper Limit of Normal
UN	Unstructured
WBC	White Blood Cell Count

15. APPENDIX: REFERENCES

1 Sun H, Saeedi P, Karuranga S, Pinkepank M, Ogurtsova K, Duncan BB, Stein C, Basit A, Chan JC, Mbanya JC, Pavkov ME. IDF Diabetes Atlas: Global, regional and country-level diabetes prevalence estimates for 2021 and projections for 2045. Diabetes research and clinical practice. 2022 Jan 1;183:109119.

2 Magliano DJ, Boyko EJ. IDF Diabetes Atlas 10th edition scientific committee. IDF DIABETES ATLAS [Internet]. 10th ed. Brussels: International Diabetes Federation. 2021.

3 Chinese Diabetes Society. Guideline for the prevention and treatment of type 2 diabetes mellitus in China (2020 edition) [J]. Chinese Journal of Diabetes Mellitus, 2021, 13(4): 315-409. [in Chinese]

4 ElSayed NA, Aleppo G, Aroda VR, Bannuru RR, Brown FM, Bruemmer D, Collins BS, Hilliard ME, Isaacs D, Johnson EL, Kahan S. 9. Pharmacologic approaches to glycemic treatment: standards of care in diabetes—2023. Diabetes Care. 2023 Jan 1;46(Supplement_1):S140-57.

5 DeFronzo RA, Ferrannini E, Groop L, Henry RR, Herman WH, Holst JJ, Hu FB, Kahn CR, Raz I, Shulman GI, Simonson DC. Type 2 diabetes mellitus. Nature reviews Disease primers. 2015 Jul 23;1(1):1-22.

6 ElSayed NA, Aleppo G, Aroda VR, Bannuru RR, Brown FM, Bruemmer D, Collins BS, Hilliard ME, Isaacs D, Johnson EL, Kahan S. 6. Glycemic targets: standards of care in diabetes—2023. Diabetes care. 2023 Jan 1;46(Supplement_1):S97-110.

A MULTICENTER, RANDOMIZED, DOUBLE-BLIND, PLACEBO-CONTROLLED, PARALLEL-GROUP PHASE III STUDY TO EVALUATE THE EFFICACY AND SAFETY OF HRS-7535 IN ADULT SUBJECTS WITH TYPE 2 DIABETES MELLITUS WITH INADEQUATE GLYCEMIC CONTROL BY DIET AND EXERCISE ALONE

Statistical Analysis Plan

Version No.:	1.0
Version Date	2026-04-23
Author:	MA Yanlin
Company	Jiangsu Hengrui Pharmaceuticals Co., Ltd.

Table of Contents

Table of Contents 2
List of Tables 3
List of Figures 3
Abbreviations 4
1. Version and Amendments 6
2. Introduction 6
  2.1. Trial Design 6
  2.2. Trial Objectives and Endpoints 7
  2.3. Estimand 9
    2.3.1. Primary Estimand 9
    2.3.2. Supplementary Estimand 9
    2.3.3. Secondary Estimand 10
  2.4. Sample size 11
  2.5. Randomization and Blinding 12
    2.5.1. Randomization 12
    2.5.2. Blinding 12
3. Statistical Hypothesis and Multiplicity 12
4. Statistical Analysis 13
  4.1. General Considerations 13
    4.1.1. Analysis Sets 13
    4.1.2. General Rules 13
    4.1.3. Baseline 15
    4.1.4. Missing data 15
    4.1.5. Rules for Intercurrent Events 15
    4.1.6. Stratification and Subgroup 16
    4.1.7. Analysis Window 16
    4.1.8. Analysis Period 18
  4.2. Characteristics of Participants 18
    4.2.1. Participant Distribution 18
    4.2.2. Protocol Deviations 18
    4.2.3. Summary of Analysis Sets 18
    4.2.4. Demographics and Baseline Characteristics 19
    4.2.5. Prior Medical History 19
    4.2.6. Prior and Concomitant Medication 19
  4.3. Efficacy Analysis 20
    4.3.1. Primary Endpoints Analysis 20
    4.3.2. Analysis of Key Secondary Endpoints 21
    4.3.3. Secondary Endpoints Analysis 22
    4.3.4. Exploratory Endpoint Analysis 24
    4.3.5. Subgroup Analysis 25
    4.3.6. Other Analysis 26
  4.4. Safety Analysis 26
    4.4.1. Adverse Events 26
    4.4.2. Laboratory Test 27
    4.4.3. Vital Signs 28
    4.4.4. Electrocardiogram 28
    4.4.5. Special Safety Analysis 28
    4.4.6. Other Safety Analysis 29
  4.5. Drug Exposure and Compliance 30
  4.6. Pharmacokinetic (PK) Analysis 30
    4.6.1. PK Concentration Analysis 30
  4.7. PD analysis: 31
  4.8. Immunogenicity (ADA) Analysis 31
  4.9. Other Analysis 31
  4.10. Interim Analysis 31
5. Changes to Protocol-planned Analyses 31
6. References 31
7. Appendices 31

List of Tables

Table 1. Five Attributes of the Primary Estimand 9
Table 2. Five Attributes of Supplementary Estimand 1 10
Table 3. Estimand Setting for Secondary Endpoints 10
Table 4. Analysis Window Setting..17
Table 5. Analysis Summary of Key Secondary Endpoints 22
Table 6. Summary Table of Secondary Endpoint Analysis 22
Table 7. Calculation Table of Single-Eye Diabetic Retinopathy Score 29
Table 8. Primary Endpoint and Dosage Judgment Criteria at Study End 30

List of Figures

Figure 1 Study Schematic Diagram 7

Abbreviations

Abbreviation	Full Name in English
AESI	Adverse Event of Special Interest
ANCOVA	Analysis of Covariance
ATC	Anatomic Therapeutic Chemical
BLQ	Below Limit of Quantitation
BMI	Body Mass Index
bpm	Beats per Minute
CI	Confidence Interval
CR	Copy Reference
CRA	Clinical Research Associate
CRF	Case Report Form
CS	Compound Symmetry
DTSQ	Diabetes Treatment Satisfaction Questionnaire
EDC	Electronic Data Capture
eGFR	Estimated Glomerular Filtration Rate
FPG	Fasting Plasma Glucose
GEE	Generalized Estimating Equation
HbA1c	Glycated Hemoglobin
HDL-C	High-Density Lipoprotein Cholesterol
HOMA-β	Homeostasis Model Assessment of β-cell Function
HOMA-IR	Homeostasis Model Assessment of Insulin Resistance
ITT	Intention to Treat
LDL-C	Low-Density Lipoprotein – Cholesterol
LLQ	Lower Limit of Quantitation
LOCF	Last Observation Carried Forward
MAR	Missing at Random
Max	Maximum
MedDRA	Medical Dictionary for Regulatory Activities
MI	Multiple Imputation
Min	Minimum
MMRM	Mixed-Effect Model Repeated Measures
PK	Pharmacokinetics
PKCS	Pharmacokinetics Concentration Set
PT	Preferred Term
QD	Quaque Die (Once Daily)
RDO	Retrieved Dropout
REML	Restricted Maximum Likelihood
SAE	Serious Adverse Event

Abbreviation	Full Name in English
SAP	Statistical Analysis Plan
S.D.	Standard Deviation
SMBG	Self-Monitoring of Blood Glucose
SOC	System Organ Classification
SS	Safety Set
TC	Total Cholesterol
TEAE	Treatment Emergent Adverse Event
TG	Triglyceride
TRAE	Treatment Related Adverse Event
UACR	Urine Albumin-to-Creatinine Ratio
UN	Unstructured
WHODrug	World Health Organization Drug Dictionary

1. Version and Amendments

Version 1.0 of the Statistical Analysis Plan (SAP) was prepared according to the latest version of the study protocol (version 2.0, 09 Sep 2024), describing the statistical analysis plan for efficacy, safety, and pharmacokinetics.

This SAP is the initial version and has no revision history.

SAP version (date)	Summary of revised content
SAP V1.0 (23 April 2026)	Not applicable

2. Introduction

2.1. Trial Design

This trial is a multicenter, randomized, double-blind, placebo-controlled, parallel-group Phase III clinical trial, including a screening period of up to 2 weeks, a 3-week run-in period, a 52-week double-blind treatment period, and a 2-week safety follow-up period. The trial population consists of adult subjects with type 2 diabetes inadequately controlled by diet and exercise alone, with a planned enrollment of 272 subjects.

Screening period (W-5 ~ W-3, up to 2 weeks):

After signing the Informed Consent Form, subjects will undergo screening assessments, and those who meet the inclusion/exclusion criteria will enter the run-in period.

Run-in period (W-3 ~ W0):

Subjects who successfully complete the screening period will begin oral administration of the single-blind run-in medication once daily for 3 weeks starting at V2, D-21.

The subject will undergo baseline examination at V3 (D-4), and the examination at V4 (D1) will be conducted to assess whether the subject meets the randomization criteria. Subjects who meet the randomization criteria will enter the 52-week double-blind treatment period.

Double-blind treatment period (W1 ~ W52):

Subjects will be randomized in a 1:1:1:1 ratio to the HRS-7535 30 mg group, 60 mg group, 90 mg group, and placebo group, with stratification by baseline HbA1c value (≤8.5%, > 8.5%) measured by the central laboratory and prior use of hypoglycemic drugs (yes, no). Subjects will start taking the double-blind treatment period drugs after randomization.

From Week 33 of the double-blind treatment period to the end of the double-blind treatment period, the placebo group will be switched to HRS-7535 30 mg treatment. All subjects will be required to undergo efficacy and safety assessments at the scheduled visits during the double-blind treatment period.

Safety follow-up period (W53 ~ W54):

After all subjects discontinue the double-blind treatment period medication, subjects will complete the safety follow-up via telephone visit at Week 54.

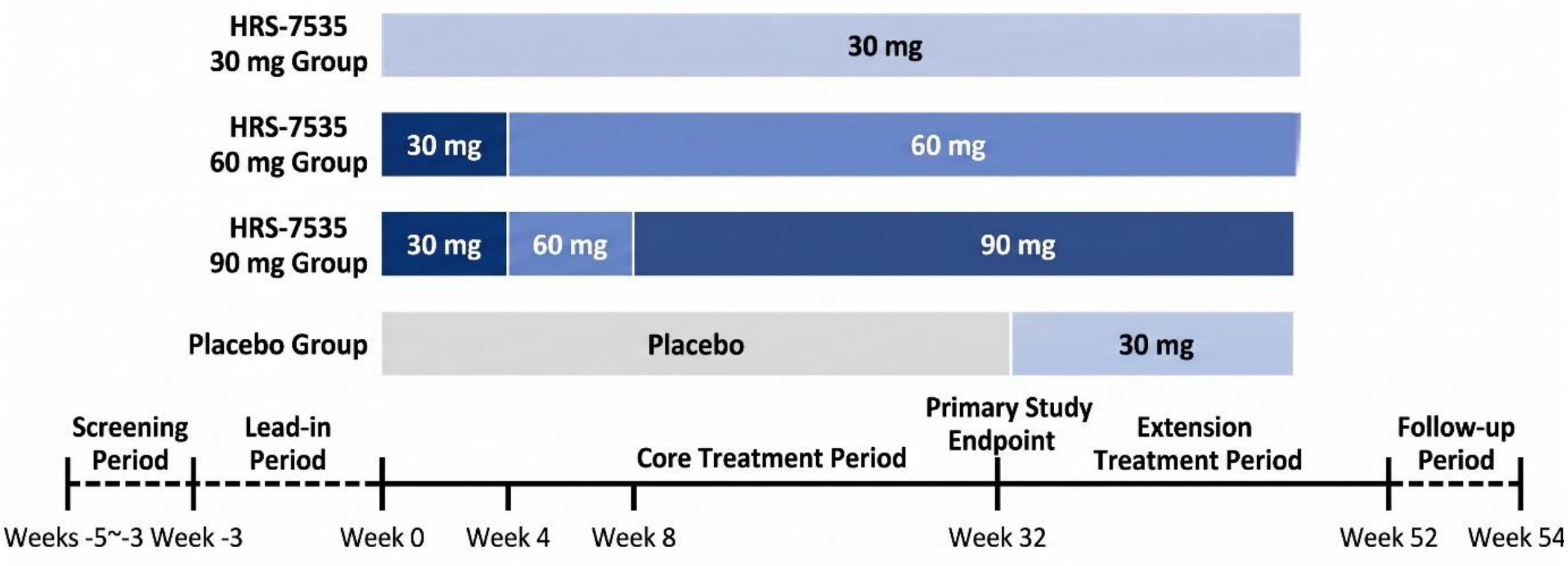

Figure 1 Study Schematic Diagram1

2.2. Trial Objectives and Endpoints

Primary Objective	Primary Endpoint
To confirm the efficacy of HRS-7535 in controlling blood glucose is superior to placebo in adult subjects with type 2 diabetes inadequately controlled by diet and exercise alone.	• The change in HbA1c from baseline at Week 32 in the HRS-7535 group compared with the placebo group
**Secondary Objectives**	**Secondary Endpoints**
In adult subjects with type 2 diabetes inadequately controlled by diet and exercise alone:<br><br>Evaluate the efficacy of HRS-7535 in other aspects of glycemic control,<br><br>Evaluate the efficacy of HRS-7535 in weight management.	Key secondary efficacy endpoints:<br><br>Compared with the placebo group, at Week 32 of treatment in the HRS-7535 group:<br>• Proportion of subjects reaching HbA1c < 7.0%<br>• Proportion of subjects reaching HbA1c ≤6.5%<br>• Change in FPG from baseline<br>• Percentage change in weight from baseline<br><br>Supplementary Secondary Efficacy Endpoint:<br><br>Compared with the placebo group, at Week 32 of treatment in the HRS-7535 group:<br>• Change in weight and waist circumference from baseline<br>• Proportion of subjects reaching HbA1c < 5.7%<br>• Changes from baseline in fasting serum insulin, fasting serum C-peptide, homeostasis model assessment of β-cell function (HOMA-β), and insulin resistance index (HOMA-IR)

	• Changes from baseline in 1-hour postprandial plasma glucose, 2-hour postprandial plasma glucose, 2-hour postprandial serum insulin, 2-hour postprandial serum C-peptide, and disposition index (DI)<br>• Change from baseline in 7-point SMBG profile<br>• Proportion of subjects requiring rescue treatment in each group<br>Compared with the placebo group, at Week 52 of treatment in the HRS-7535 group:<br>• Change in HbA1c from baseline<br>• Change in FPG from baseline<br>• Percentage change in weight from baseline<br>• Change in weight and waist circumference from baseline<br>• Proportion of subjects reaching HbA1c < 7.0%<br>• Proportion of subjects reaching HbA1c ≤6.5%<br>• Proportion of subjects reaching HbA1c < 5.7%<br>• Changes from baseline in fasting serum insulin, fasting serum C-peptide, homeostasis model assessment of β-cell function (HOMA-β), and insulin resistance index (HOMA-IR)<br>• Changes from baseline in 1-hour postprandial plasma glucose, 2-hour postprandial plasma glucose, 2-hour postprandial serum insulin, 2-hour postprandial serum C-peptide, and disposition index (DI)<br>• Change from baseline in 7-point SMBG profile<br>• Proportion of subjects requiring rescue treatment in each group
In adult subjects with type 2 diabetes inadequately controlled by diet and exercise alone:<br>To evaluate the safety and tolerability of HRS-7535.	Week 32 and Week 52 of Treatment<br>• Adverse events, hypoglycemic events, laboratory tests, vital signs, physical examination, 12-lead electrocardiogram, diabetic retinopathy screening and assessment
In adult subjects with type 2 diabetes inadequately controlled by diet and exercise alone:<br>To evaluate the pharmacokinetics (PK) of HRS-7535.	• Plasma HRS-7535 concentration
In adult subjects with type 2 diabetes inadequately controlled by diet and exercise alone:<br>To assess patient reported outcomes in each treatment group.	• Change from baseline to Week 32 and Week 52 in Diabetes Treatment Satisfaction Questionnaire status version (DTSQs)

Exploratory Objective	Exploratory endpoints
In adult subjects with type 2 diabetes inadequately controlled by diet and exercise alone:<br>Evaluate the efficacy of HRS-7535 in improving blood pressure, lipid metabolism, and renal function.	Compared with the placebo group, at Week 32 and Week 52 of treatment in the HRS-7535 group:<br>• Change from baseline in systolic blood pressure and diastolic blood pressure<br>• Percent change in TG, TC, LDL-C and HDL-C from baseline;<br>• Percentage change in UACR from baseline

Abbreviations: DTSQ = Diabetes Treatment Satisfaction Questionnaire; FPG = Fasting Plasma Glucose; HbA1c = Glycated hemoglobin; HDL-C = High-Density Lipoprotein Cholesterol; HOMA-IR = Homeostasis Model Assessment of Insulin Resistance; HOMA-β = Homeostasis Model Assessment of β-cell function; LDL-C = Low-Density Lipoprotein Cholesterol; PK = Pharmacokinetics; SMBG = Self-Monitoring of Blood Glucose; TC = Total Cholesterol; TG = Triglycerides; UACR = Urine Albumin/Creatinine Ratio.

2.3. Estimand

2.3.1. Primary Estimand

The primary efficacy endpoint will be analyzed under the primary estimand (treatment-policy estimand), with specific settings as follows:

Table 1. Five Attributes of the Primary Estimand1

Attribute	Definition
**Treatment**	Trial group: HRS-7535 30 mg, 60 mg, 90 mg, orally administered, QD<br>Control group: placebo, administered orally, QD
**Population**	Adult subjects with type 2 diabetes inadequately controlled by diet and exercise alone
**Variable (endpoint)**	Change from Baseline in HbA1c at Week 32
**Intercurrent events and strategies**	
Intercurrent event 1: premature discontinuation of study treatment	Treatment-policy strategy: if the subject prematurely discontinues study treatment before the primary endpoint at Week 32, the subsequently collected data will be included in the statistical analysis.
Intercurrent event 2: initiation of additional hypoglycemic drugs*	Treatment-policy strategy: if the subject starts using additional hypoglycemic drugs before the primary endpoint at Week 32, the subsequently collected data will be included in the statistical analysis.
**Population-level summary**	Difference in mean change from baseline in HbA1c at Week 32 between groups

Abbreviations: HbA1c = Glycated hemoglobin; QD=once daily.
*Additional hypoglycemic drugs are defined as any hypoglycemic drugs used cumulatively for more than 14 days.

2.3.2. Supplementary Estimand

The primary efficacy endpoint will be evaluated further under two supplementary estimands, with the specific settings as follows:

1. Supplementary estimand 1 (efficacy estimand):

Table 2. Five Attributes of Supplementary Estimand 1

Attribute	Definition
**Treatment**	Trial group: HRS-7535 30 mg, 60 mg, 90 mg, orally administered, QD<br>Control group: placebo, administered orally, QD
**Population**	Adult subjects with type 2 diabetes inadequately controlled by diet and exercise alone
**Variable (endpoint)**	Change from Baseline in HbA1c at Week 32
**Intercurrent events and strategies**	
Intercurrent event 1: premature discontinuation of study treatment	Hypothetical strategy: if the subject prematurely discontinues study treatment before the primary endpoint at Week 32, the subsequently collected data will not be included in the statistical analysis
Intercurrent event 2: initiation of additional hypoglycemic drugs*	Hypothetical strategy: if the subject starts using additional hypoglycemic drugs before the primary endpoint at Week 32, the subsequently collected data will not be included in the statistical analysis.
Intercurrent event 3: insufficient drug exposure before visits	Hypothetical strategy: if the subject had insufficient drug exposure within 90 days before a visit, the data collected at that visit point will not be included in the statistical analysis.
**Population-level summary**	Difference in mean change from baseline in HbA1c at Week 32 between groups

Abbreviations: HbA1c = Glycated hemoglobin; QD=once daily.
*Additional hypoglycemic drugs are defined as any hypoglycemic drugs used cumulatively for more than 14 days.
The definition of insufficient drug exposure is described in Section 4.1.5.

2. Supplementary estimand 2: the strategies for addressing intercurrent events 1 and 2 are hypothetical strategies, while the strategy for intercurrent event 3 is the treatment-policy strategy. All other attributes and intercurrent event settings are the same as those of supplementary estimand 1.

2.3.3. Secondary Estimand

The assessment of secondary endpoints will be performed with secondary estimands, and the strategies for addressing the intercurrent events are as follows:

Table 3. Estimand Setting for Secondary Endpoints

Endpoint	Treatment-Policy Estimand	Efficacy Estimand	No Estimand
**Key secondary efficacy endpoints:**<br>At Week 32 of treatment:<br>• Proportion of subjects reaching HbA1c < 7.0%<br>• Proportion of subjects reaching HbA1c ≤6.5%<br>• Change in FPG from baseline<br>• Percentage change in weight from baseline	√	√	
**Supplementary secondary efficacy endpoint:**<br>At Week 52 of treatment:<br>• Change in HbA1c from baseline<br>• Change in FPG from baseline	√	√	

• Percentage change in weight from baseline<br>• Change in weight and waist circumference from baseline			
**Supplementary secondary efficacy endpoint:**<br>At Week 32 of treatment:<br>• Change in weight and waist circumference from baseline<br>• Proportion of subjects reaching HbA1c < 5.7%<br>• Changes from baseline in fasting serum insulin, fasting serum C-peptide, HOMA-β, and HOMA-IR<br>• Changes from baseline in 1-hour postprandial plasma glucose, 2-hour postprandial plasma glucose, 2-hour postprandial serum insulin, 2-hour postprandial serum C-peptide, and DI<br>• Change from baseline in 7-point SMBG profile<br><br>**Exploratory endpoints:**<br>At Week 32 of treatment:<br>• Change from baseline in systolic blood pressure and diastolic blood pressure<br>• Percent change in TG, TC, LDL-C and HDL-C from baseline;<br>• Percentage change in UACR from baseline		√	
Other secondary and exploratory endpoints			√

For key secondary efficacy endpoints, the treatment-policy estimand is the primary estimand for confirmatory analyses, while the efficacy estimand serves as the supplementary estimand. The treatment-policy estimand in Table 3 is the same as the primary estimand in Section 2.3.1, with the variables (endpoints) changed to the corresponding endpoints and the population-level summaries changed to the between-group differences for the corresponding endpoints. The efficacy estimand is the same as supplementary estimand 1 in Section 2.3.2, with the variables (endpoints) changed to the corresponding endpoints and the population-level summaries changed to the between-group differences for the corresponding endpoints.

2.4. Sample size

The sample size determination of this study is based on the primary endpoint, i.e., the change in HbA1c from baseline at Week 32. Based on the results of Phase II study, assuming that the difference in HbA1c between each group of HRS-7535 and the placebo group is -0.85% for subjects without intercurrent events, and 0 for subjects with intercurrent events, the overall

difference would be -0.72% (considering an intercurrent event rate of 15%), with a standard deviation (SD) = 1.1%. A total of 272 subjects (68 per group) will be randomly assigned in a 1:1:1:1 ratio to the HRS-7535 30 mg group, 60 mg group, 90 mg group, and placebo group, providing an overall power of 90% to detect the superiority of all three groups of HRS-7535 over the placebo group at a one-sided significance level of 0.025.

2.5. Randomization and Blinding

2.5.1. Randomization

This study is a randomized controlled study design, using the stratified block randomization method. Screened subjects will be randomized to different groups at a 1:1:1:1 ratio. Randomization should be conducted as per the following stratification factors:

- Baseline HbA1c value measured by the central laboratory (≤8.5% vs. > 8.5%)
- Previous use of hypoglycemic drugs (yes vs. no).

2.5.2. Blinding

This study is a double-blind study. The labels of the investigational product and placebo are completely identical except for the kit number. Blinding must be maintained throughout subject enrollment and administration, documentation and evaluation of study results, inspection of the study process, and data management. The subjects, investigators, sponsor's researchers participating in clinical evaluation, and the clinical research associate (CRA) participating in this study will be unaware of which investigational product the subjects receive. The third-party laboratory testing personnel will be unblinded and will not perform PK testing for the placebo group. To control potential study bias, the blind codes will be properly kept as confidential information to prevent disclosure to the investigators and study staff in direct contact with the investigators until the study database lock is completed.

3. Statistical Hypothesis and Multiplicity

This study is designed as a superiority trial. During the testing process, each dose group of HRS-7535 will be compared with the placebo group regarding the primary efficacy endpoint and key secondary endpoints. Superiority tests will be conducted at a one-sided significance level of 0.025. The corresponding alternative hypotheses (the first subscript 1, 2, 3 in Hxx represents the HRS-7535 90 mg, 60 mg, and 30 mg groups, respectively; the second subscript 1-5 represents different endpoints) are as follows:

- H_{11}, H_{21}, H_{31}: superiority of HRS-7535 90 mg, 60 mg, 30 mg groups vs. placebo group in HbA1c change from baseline after 32 weeks of treatment
- H_{12}, H_{22}, H_{32}: superiority of HRS-7535 90 mg, 60 mg, 30 mg groups vs. placebo

group in proportion of subjects with HbA1c < 7.0% after 32 weeks of treatment

- H_{13}, H_{23}, H_{33}: superiority of HRS-7535 90 mg, 60 mg, 30 mg groups vs. placebo group in proportion of subjects with HbA1c ≤ 6.5% after 32 weeks of treatment
- H_{14}, H_{24}, H_{34}: superiority of HRS-7535 90 mg, 60 mg, 30 mg groups vs. placebo group in FPG change from baseline after 32 weeks of treatment
- H_{15}, H_{25}, H_{35}: superiority of HRS-7535 90 mg, 60 mg, 30 mg groups vs. placebo group in percentage change of weight from baseline after 32 weeks of treatment.

At the one-sided significance level of 0.025, a fixed-sequence testing procedure will be used to test the above hypotheses in the following order: H_{11}, H_{21}, H_{31}, H_{12}, H_{22}, H_{32}, H_{13}, H_{23}, H_{33}, H_{14}, H_{24}, H_{34}, H_{15}, H_{25}, and H_{35}. The testing procedure will be stopped if any one-sided p-value of the tests exceeds 0.025.

4. Statistical Analysis

4.1. General Considerations

4.1.1. Analysis Sets

Intention-to-treat (ITT) analysis set: All randomized subjects. Subjects will be analyzed according to the randomized treatment. Efficacy analyses will be based on the ITT set.
Safety set (SS): All subjects who receive at least one dose of the investigational product after randomization. Subjects will be analyzed according to the randomized treatment.
PK concentration analysis set (PKCS): All subjects who have been randomized to receive HRS-7535 treatment and have at least one evaluable post-dose PK concentration measurement.

4.1.2. General Rules

Study Day:

The date of the first dose of the investigational product during the double-blind treatment period is counted as the study start date (Day 1). If the subject does not receive the investigational product after randomization, the randomization date will be considered as Day 1. Using the study start date as a reference point, the study day corresponding to the examination dates (laboratory tests, 12-lead electrocardiogram, etc.) or event dates will be calculated using the following formula:

If the date of the examination/event is prior to the start of the study, the study day = date of the examination/event - study start date,
If the date of the examination/event is on or after the start of the study, the study day number = date of the examination/event - study start date + 1.

General Analysis

Descriptive statistics will be summarized as follows according to variable type:

- Continuous variables will be summarized using the number of subjects (n), mean, standard deviation (S.D.), median, minimum (min), and maximum (max).
- Categorical variables will be summarized using frequency and percentage.
- The PK concentration data will be summarized by number of subjects (n), n of BLQ, mean, geometric mean (GeoMean), standard deviation (S.D.), coefficient of variation (CV%), geometric coefficient of variation (GeoCV%), median, minimum (min), and maximum (max).

- For the following variables, geometric mean and geometric standard deviation will be included in descriptive statistics, and natural logarithm transformation will be performed in the analyses: lipase, amylase, fasting serum insulin, fasting serum C-peptide, 2-hour postprandial serum insulin, 2-hour postprandial serum C-peptide, TG, TC, LDL-C, HDL-C, ALT, AST, UACR, calcitonin, HOMA-β, HOMA-IR, and DI.
- In the analysis of DI, the total DI based on C-peptide (ISSI-2_cpep) will be used, as detailed in Appendix 7.2.

Decimal places

The decimal places in the analysis report will be rounded and presented according to the following rules:

- The decimal places of the minimum and maximum will be consistent with the decimal places of the raw data collected. The mean and median will have 1 more decimal place than the raw data, the standard deviation will have 2 more decimal places than the raw data, but a maximum of 2 decimal places will be retained.
- Percentages will be rounded to 1 decimal place. If the frequency is 0, the percentage will not be displayed.
- The p-value will be retained to four decimal places. If the p-value is < 0.0001, it will be reported as "< 0.0001"; if the p-value is > 0.9999, it will be reported as "> 0.9999".
- The Confidence Interval (CI) will have 1 more decimal place than the raw data, but no more than 2 decimal places.

Unscheduled visit

The analysis window for efficacy analyses is described in Section 4.1.7. For safety data summarized by descriptive statistics based on scheduled visits, only data from scheduled visit

time points will be included, excluding unscheduled visits. If the incidence of abnormalities or the most clinically significant post-baseline changes relative to baseline are summarized using shift tables, data from unscheduled visit time points will also be included. Unscheduled assessments will also be used to evaluate baseline. Listings will also present results from unscheduled visits.

Software

All statistical analyses will be performed in SAS® 9.4 or later versions.

4.1.3. Baseline

Baseline is defined as the last non-missing measurement/assessment before the first use of the investigational product (the first entry in V4 drug administration records), including the day of administration. If the subject does not use the investigational product after randomization, the baseline is defined as the last non-missing measurement/assessment before randomization (including the day of randomization). The determination of baseline includes test results from unscheduled visits. If a scheduled visit and an unscheduled visit occur on the same day and it is not possible to determine the chronological order, the test result from the scheduled visit is used, and the unscheduled visit on the same day is considered as a pre-baseline result.

4.1.4. Missing data

4.1.4.1. Date missing

Missing date-type data will be imputed according to Hengrui's Standards WI-BD-04-003 Date Type Missing Data Imputation in Clinical Trial V2.0.

4.1.4.2. PK Concentration Data

Below the Limit of Quantitation (BLQ)

For pre-dose PK concentrations below the lower limit of quantification (LLQ), 0 will be used in the summary analysis; for post-dose concentrations below LLQ, LLQ/2 will be used in the summary analysis. If more than half of the subjects at a specific time point have PK concentrations below LLQ, only the number of subjects, the number of observations below LLQ, and the maximum value will be summarized for concentration data at this time point. The LLQ value of HRS-7535 is 2 ng/mL.

4.1.4.3. Safety Data

Missing safety data will not be processed.

4.1.5. Rules for Intercurrent Events

Intercurrent event 1 - premature discontinuation of treatment: defined as any subject whose reason for treatment discontinuation is not 'completed'. The date of this intercurrent event is

the end date of the actual last dose of HRS-7535/placebo. For subjects without an end date of last dose, it is the start date of the actual last dose. For randomized subjects who do not receive investigational product, the date of this intercurrent event is the randomization date.

Intercurrent event 2 - initiation of additional hypoglycemic drugs: additional hypoglycemic drugs are defined as any hypoglycemic drugs used for more than 14 days cumulatively. The cumulative 14 days are defined as the total number of days of use of any additional hypoglycemic drug from randomization to the primary endpoint (regardless of the number of hypoglycemic drugs taken on the same day, it is counted as 1 day). The date of this intercurrent event is the date when the cumulative medication reaches 14 days.

Intercurrent event 3 - insufficient drug exposure before visits: for the 32-week core treatment period, insufficient drug exposure is defined as the actual total drug exposure within 90 days before a visit point (based on the previous two visits, a visit point defined as HbA1c test date) being less than 50% of the target total exposure, or the number of PK test values below the lower limit of quantification (BLQ) within 90 days exceeding 50% of the total number of PK tests (3 times) that should be performed within 90 days. The determination of insufficient exposure before visits applies starting from V10 (W20). For the extension period, insufficient drug exposure is defined as the actual total drug exposure within 90 days before the visit point (based on the previous two visits, a visit point defined as HbA1c test date) being less than 50% of the target total exposure. The placebo group only uses drug exposure to determine whether this intercurrent event occurs.

The date for intercurrent events is defined as the earliest date among all intercurrent events.

4.1.6. Stratification and Subgroup

The randomization stratification factors are as follows:

- Baseline HbA1c value measured by central laboratory (≤8.5% vs. > 8.5%);
- Previous use of hypoglycemic drugs (yes vs. no).

The subgroup variables include:

- Disease course of type 2 diabetes (> 2 years vs. ≤2 years)
- Gender (males vs. females)
- Baseline BMI (< 24 kg/m2 vs. 24-28 kg/m2 vs. > 28 kg/m2)
- Age (≥65 years vs. < 65 years).

4.1.7. Analysis Window

For the analyses of efficacy endpoints, the analysis window is defined as shown in Table 4 (calculated based on study days). If multiple assessment results (excluding the assessment

results of unscheduled visits) fall into the same analysis window, the assessment closest to the scheduled study day in the analysis window will be used for summary analysis (except baseline analysis; refer to the definition of baseline of each endpoint). If there are still multiple assessment results (one before and one after) that are equally close to the study date, the latest assessment result will be used for summary analysis. The analysis windows after baseline will not consider the assessment results of unscheduled visits.

Table 4. Analysis Window Setting

**Analysis Visit**	**Planned Study Day**	**HbA1c, FPG, Weight, Waist Circumference, Systolic Blood Pressure, Diastolic Blood Pressure Analysis Window (Day)**	**Fasting Serum Insulin, Fasting Serum C-Peptide, HOMA Analysis Window (Day)**	**1-Hour Postprandial Plasma Glucose, 2-Hour Postprandial Plasma Glucose, 2-Hour Postprandial Serum Insulin, 2-Hour Postprandial Serum C-Peptide, DI Analysis Window (Day)**	**TG, TC, LDL-C, HDL-C Analysis Window (Day)**	**UACR Analysis Window (Day)**
Baseline	D1	<=1	<=1	<=1	<=1	<=1
Week 4	D29	[2, 43]				[2, 43]
Week 8	D57	[44, 71]			[2, 99]	[44, 85]
Week 12	D85	[72, 99]				
Week 16	D113	[100, 127]	[2, 169]			[86, 148]
Week 20	D141	[128, 162]			[100, 183]	
Week 26	D183	[163, 204]				[149, 204]
Week 32	D225	[205, 246]	[170, 295]	[2, 295]	[184, 295]	[205, 295]
Week 38	D267	[247, 288]				
Week 44	D309	[289, 337]				
Week 52	D364	[338, 394]	[296, 394]	[296, 394]	[296, 394]	[296, 394]

For the derived endpoints HOMA and DI, the data of insulin, C-peptide, and glucose within the same planned visit will be used for derivation, and the earliest test date will be selected as the test date for HOMA and DI. Then, the analysis window for HOMA and DI will be determined based on this date.

No analysis window will be set up for endpoints (including safety endpoints) other than efficacy, and analyses will be performed at each visit based on the actually recorded visit points.

For endpoints such as HbA1c and plasma glucose that have both central and local laboratory data, only the central laboratory data will be used in efficacy analyses.

4.1.8. Analysis Period

The division between the run-in period and the core treatment period is based on the date of the first dose in the double-blind treatment period. The period before this date (exclusive) is the run-in period, and the period after this date is the core treatment period.

The division of the core treatment period and the extension period is based on the last drug administration date before V12 (W32). The period before this date (inclusive) is the core treatment period, and the period after this date (exclusive) is the extension period. For subjects who do not receive treatment in the extension period, all data will be included in the core period analysis.

For the screening and assessment of diabetic retinopathy, the analysis period will be divided based on the test date of the V12. The period including this date and before will be defined as the core treatment period, while the period after this date (exclusive) will be defined as the extension period. For subjects who do not receive treatment in the extension period, all data will be included in the core period analysis.

4.2. Characteristics of Participants

The definition of "participant" in this section is the same as "subject". The analysis of participant characteristics is based on ITT.

4.2.1. Participant Distribution

The participants will be summarized according to the following categories: number of screened participants, number and reasons for screen failures, number of participants entering the run-in period, number of run-in failures, number of randomized participants, number of randomized but not treated participants, number of treated participants, number of participants who discontinue treatment prematurely, number of participants who complete the study, and number of participants who withdraw from the study prematurely. Categorized summaries will also be provided for reasons of premature discontinuation of treatment and withdrawal from the study.

Listings will be provided for all participant distribution information.

4.2.2. Protocol Deviations

Prior to database lock, all participant data on the CRF will be inspected for major protocol deviations. All potential major protocol deviations will be reviewed and assessed by the investigator and sponsor.

All major protocol deviations will be summarized and presented in listings.

4.2.3. Summary of Analysis Sets

Descriptive statistics of the number of participants in each analysis set will be provided.

4.2.4. Demographics and Baseline Characteristics

Based on ITT, descriptive statistics will be performed on the following demographic and baseline characteristics of participants: age, sex, height, body weight, waist circumference, BMI and baseline characteristics (duration of type 2 diabetes, previous use of hypoglycemic drugs, HbA1c, fasting plasma glucose, fasting serum insulin, fasting serum C-peptide, HOMA-β, HOMA-IR, 1-hour postprandial plasma glucose, 2-hour postprandial plasma glucose, 2-hour postprandial serum insulin, 2-hour postprandial serum C-peptide, DI, TG, TC, HDL-c, Non-HDL-c, systolic blood pressure, diastolic pressure, heart rate).

Calculation of age in demographics and baseline characteristics:

Age (years) = (date of Informed Consent Form signing – date of birth + 1) / 365.25, rounded down.

BMI calculation:

BMI (kg/m^2) = weight (kg)/height $(m)^2$.

Calculation of disease duration:

Duration of type 2 diabetes (years) = (date of signing Informed Consent Form - date of diabetes diagnosis/onset + 1) / 365.25.

If the diagnosis/start date of diabetes is missing, the following rules will be applied for imputation: if only the year of the diagnosis/start date is recorded, the missing month and day will be imputed as the first day (01 Jan.). If only the year and month of the diagnosis/start date are recorded, the missing day will be imputed as the 1st, and the disease duration will be calculated accordingly.

Calculation of HOMA and DI: see Appendix 7.2 for details.

4.2.5. Prior Medical History

Medical history will be coded by system organ class (SOC) and preferred term (PT) using the Chinese version of Medical Dictionary for Regulatory Activities (MedDRA).

Based on the ITT, descriptive statistics will be performed on the medical history of the participants by treatment group. Listings will be provided for all the medical history.

4.2.6. Prior and Concomitant Medication

Prior medications and concomitant medications will be coded using the World Health Organization Drug Dictionary (WHO-Drug Dictionary), and the drugs will be categorized according to the ATC coding system to summarize the number of participants with prior medications and concomitant medications in each treatment group. The analyses of prior and concomitant medications will be based on the SS set, and all prior and concomitant medications will be listed. In the summary analyses, prior medications include drugs that

have been discontinued before the first administration of the study drug during the treatment period (starting from the double-blind period); concomitant medications include drugs that are administered after the first administration of the study drug during the treatment period (but no later than the date of the last administration of the study drug), or drugs that have been administered before the first administration of the study drug during the treatment period and are still in use.

4.3. Efficacy Analysis

4.3.1. Primary Endpoints Analysis

The definition of the primary estimand is provided in Section 2.3.1. The stratification factors involved in the primary endpoint analyses will be based on the randomization system.

4.3.1.1. Primary Analysis

For the primary estimand, the primary study endpoint will be analyzed based on the ITT set. The treatment-policy strategy will be applied for all intercurrent events, and data after intercurrent events will be included in the analysis.

Missing data due to intercurrent events will be imputed using the RDO (Retrieved Dropout) method. If the data do not support the RDO method, the CR (Copy Reference) method will be used. Missing data unrelated to intercurrent events are assumed to be missing at random (MAR). The analysis results of the imputed datasets will be combined using Rubin's rules[1]. The HbA1c measurements from Week 0 to Week 32 will be analyzed using an analysis of covariance (ANCOVA) model with baseline HbA1c value as a covariate, stratification factors [prior use of hypoglycemic drugs (yes, no)] and treatment group as fixed effects. The least squares means, standard errors, 95% confidence intervals (CIs) of the changes from baseline in HbA1c at Week 32 for each group and between groups, as well as the one-sided p-values for the difference between each dosage group of HRS-7535 and the placebo group will be estimated and listed. The trend of descriptive mean (based on observed values) of change from baseline in HbA1c over time for each treatment group will be presented.

4.3.1.2. Sensitivity Analysis

Sensitivity analysis 1: for the primary estimand, the primary study endpoint will be analyzed based on the ITT set. The treatment-policy strategy will be applied for all intercurrent events, and data after intercurrent events will be included in the analysis. For all missing HbA1c measurements from Week 0 to Week 32, the last observation carried forward (LOCF) method will be used for imputation. HbA1c measurements from Week 0 to Week 32 will be analyzed using analysis of covariance (ANCOVA) model with baseline HbA1c value as covariate,

stratification factors including previous use of hypoglycemic drugs (yes, no) and treatment group as fixed effects.

Sensitivity analysis 2: for the primary estimand, the primary study endpoint will be analyzed based on the ITT set. The treatment-policy strategy will be applied for all intercurrent events, and data after intercurrent events will be included in the analysis. For HbA1c measurements from 0-32 weeks, a tipping point analysis method will be used to impute all missing data. For the experimental group and control group data, a series of penalty coefficients (i.e., adding/subtracting a delta value to the imputed data of the experimental group and control group respectively) will be set to reduce the between-group differences, with the objective of finding the penalty coefficient (delta value) that makes the between-group differences not statistically significant. Under each pair of penalty coefficients, the MAR-based MI method will be used for multiple imputation, a delta value will be added/subtracted to the imputed data, and each imputed dataset will be analyzed using ANCOVA, consistent with the primary analysis. Using Rubin's rules[1], the analysis results of the imputed datasets will be combined.

4.3.1.3. Supplementary Analysis

1. For supplementary estimand 1, the primary study endpoint will be analyzed based on the ITT set. The hypothetical strategy will be applied for all intercurrent events, and data after intercurrent events will be considered as missing and not included in the analysis, and all missing data will be assumed to be missing at random. Changes from baseline in HbA1c measurements at Weeks 0-32 will be analyzed using a mixed-effect model for repeated measures (MMRM) with a restricted maximum likelihood (REML) method. The model includes stratification factors (previous use of hypoglycemic drugs [yes, no]), treatment, visit, and the interaction between treatment and visit as fixed effects, baseline HbA1c as the covariate, and within-subject variability with an unstructured (UN) or compound symmetry (CS) covariance matrix. The least squares means, standard errors, 95% CIs of the changes from baseline in HbA1c at Week 32 for each group and between groups, as well as the one-sided p-values for the between-group differences between each dosage group of HRS-7535 and the placebo group, will be estimated and listed.
2. For supplementary estimand 2, the analysis of the primary endpoint will be based on the ITT set, with the same analysis method as described in supplementary analysis 1.

4.3.2. Analysis of Key Secondary Endpoints

Based on the ITT, the analyses of key secondary endpoints adopt both the treatment-policy estimand and the efficacy estimand, with specific analysis methods shown in Table 5.

Table 5. Analysis Summary of Key Secondary Endpoints

Endpoint	Analytical Method	Notes
• Proportion of subjects reaching HbA1c < 7.0% at Week 32 of treatment<br>• Proportion of subjects reaching HbA1c ≤6.5% at Week 32	**Treatment-policy estimand:**<br>The generalized estimating equation (GEE) will be used to analyze the measurements from weeks 0 to 32. The model includes stratification factors (prior use of hypoglycemic drugs [yes, no]), treatment, visit, and the interaction between treatment and visit as fixed effects, baseline HbA1c as the covariate, and subject as the random effect. The within-subject variation is modeled using an unstructured (UN) or compound symmetry (CS) covariance matrix. The odds ratios between the two groups, along with its 95% CIs and one-sided p-values, are output. After imputing missing HbA1c values according to the imputation rules in the primary analysis method for the primary estimand, the proportion of subjects with HbA1c < 7.0%/≤6.5% are determined.<br>**Efficacy estimand:**<br>The analysis model is the same as the one for treatment-policy estimand, and GEE is used to analyze the measurements from Week 0 to 32. Missing data will be handled based on GEE itself.	
• Change in FPG from baseline at Week 32 of treatment<br>• Percentage change in weight from baseline at Week 32 of treatment	**Treatment-policy estimand:**<br>The measurements from Week 0 to 32 are analyzed, with the analysis method and imputation rules consistent with the primary analysis of the primary estimand, i.e., ANCOVA is used.<br>**Efficacy estimand:**<br>It is analyzed using a mixed-effect model repeated measures (MMRM) based on restricted maximum likelihood (REML). The model includes stratification factors (previous use of hypoglycemic drugs [yes, no] and baseline HbA1c value [≤8.5%, > 8.5%] measured by central laboratory), treatment, visit, and treatment-by-visit interaction as fixed effects, with the corresponding baseline of each endpoint as the covariate, and the within-subject variability is modeled using an unstructured (UN) or compound symmetry (CS) covariance matrix.<br>Output the least square mean differences between the two groups, along with its 95% CIs and one-sided p-values. Missing data will be handled based on MMRM itself.	

4.3.3. Secondary Endpoints Analysis

Based on the ITT, the secondary endpoints will be analyzed, and the estimand framework is detailed in Section 2.3.3, with specific analysis methods shown in Table 6.

Table 6. Summary Table of Secondary Endpoint Analysis

Endpoint	Analytical Method	Notes
At Week 52 of treatment:<br>• Change in HbA1c from baseline<br>• Change in FPG from baseline<br>• Percentage change in weight from baseline<br>• Change in weight and waist circumference from baseline	**Treatment-policy estimand:**<br>The measurements from weeks 0-52 are analyzed using the same analysis method and imputation rules as the primary analysis for the primary estimand, namely ANCOVA analysis.<br><br>**Efficacy estimand:**<br>The measurements from Week 0 to 52 are analyzed, with the analysis method and imputation rules being consistent with those for supplementary estimand 1, i.e., MMRM analysis is employed.	
At Week 32 of treatment<br>• Change in weight and waist circumference from baseline<br>• Proportion of subjects reaching HbA1c < 5.7%<br>• Changes from baseline in fasting serum insulin, fasting serum C-peptide, HOMA-β and HOMA-IR<br>• Changes from baseline in 1-hour postprandial plasma glucose, 2-hour postprandial plasma glucose, 2-hour postprandial serum insulin, 2-hour postprandial serum C-peptide, DI<br>• Change from baseline in 7-point SMBG profile	**Efficacy estimand:**<br>• Binary endpoints: the measurements from Week 0 to Week 32 are analyzed, and the analysis method and imputation rules are consistent with those for the binary variables in the key secondary endpoints, i.e., GEE analysis is used.<br>• Continuous endpoints with multiple post-baseline measurements: the measurements from Week 0 to 32 are analyzed, with the analysis method and imputation rules consistent with those for supplementary estimand 1, i.e., MMRM analysis is used.<br>• For continuous variables with only one post-baseline measurement, analysis will be performed using ANCOVA model. For subjects experiencing any intercurrent events, data subsequent to the earliest occurrence date will be set as missing. The treatment group will serve as the independent variable, with corresponding baseline values as covariates. The least squares mean (LSmean) changes from baseline, standard errors, and 95% CIs for each treatment group, as well as point estimates of differences between each treatment group and the control group, standard errors, and 95% CIs will be calculated and listed. Only subjects with non-missing baseline data will be included in the model, and all post-baseline missing data (including data set as missing due to intercurrent events) will be imputed using MI.	If the endpoint requires natural log transformation (see 4.1.2 for details), the log-transformed baseline value is used as a covariate in the model. The least squares means and 95% CIs for each group from the model output will be back-transformed (anti-log) to obtain the geometric mean ratio relative to baseline and its 95% CI for each treatment group. The difference in least squares means and its 95% CIs between treatment groups from the model output will be back-transformed (anti-log) to obtain the ratio of geometric mean ratios relative to baseline between groups (treatment vs. placebo) and its 95% CI.
Other Endpoints	Not based on the estimand framework, descriptive summaries will be performed.	

The 7-point SMBG profile is defined as the area under the curve of the 24-hour glucose profile, specifically calculated as the sum of seven trapezoidal areas divided by 24 hours, unit: mmol/L.

The area of a single trapezoid = sum of the blood glucose measurements at two consecutive points * time difference between scheduled measurements / 2.

Scheduled measurement times are at 7:00 (before breakfast), 9:00 (after breakfast), 12:00 (before lunch), 14:00 (after lunch), 18:00 (before dinner), 20:00 (after dinner), and 22:00 (before sleep). When calculating the last trapezoid area, the last point value is considered the same as the first point, with the next scheduled sampling time defined as the next day at 7:00.

For each visit, the 7-point SMBG measurements at any scheduled sampling time point can be used as the value for that point. If multiple measurements are taken, the mean value of that point is calculated. If data for that observation point are all missing, the mean of the remaining non-missing data points is used for imputation. If ≥ 3 measurement values are missing at the scheduled measurement times for a visit, the 7-point SMBG data for that visit is considered missing.

The 7-point SMBG analysis is not based on analysis windows and it directly uses the visit information entered in EDC.

The 7-point SMBG at each time point of every visit will be described by group and plotted accordingly.

4.3.4. Exploratory Endpoint Analysis

Exploratory endpoint analyses will be based on ITT, and all collected data will be included in the analyses.

For the changes in systolic blood pressure and diastolic blood pressure from baseline at Week 32 of treatment, the analyses will be performed using a mixed-effect model repeated measures (MMRM) based on restricted maximum likelihood (REML). The model includes measurements from Weeks 0-32, with stratification factors (prior use of hypoglycemic drugs [yes, no] and baseline HbA1c value [≤8.5%, > 8.5%] measured by the central laboratory), treatment, visit, and the interaction between treatment and visit as fixed effects, the corresponding baseline of each endpoint as the covariate, and the within-subject variation using an unstructured (UN) or compound symmetry (CS) covariance matrix.

For the percentage changes from baseline in Triglycerides (TG), Total Cholesterol (TC), Low-Density Lipoprotein Cholesterol (LDL-C), High-Density Lipoprotein Cholesterol (HDL-C), and UACR at Week 32, the same MMRM model described above will be applied

after log transformation. The covariate "baseline value of each endpoint as a covariate" in the model will be correspondingly changed to log-baseline value of each endpoint. The least squares means and 95% CIs for each treatment group output by the model will be exponentiated to obtain the percentage changes from baseline in geometric means and their 95% CIs for each treatment group. The calculation method: $(\exp(\text{LSMean}) - 1) * 100\%$, $(\exp(95\%\ \text{CI boundaries}) - 1) * 100\%$. The least squares mean differences and 95% CIs between treatment groups output by the model will be exponentiated to obtain the percentage differences in changes from baseline in geometric means between groups (experimental group vs placebo). The calculation method: $(\exp(\text{LSMean Difference}) - 1) * 100\%$, $(\exp(95\%\ \text{CI boundaries}) - 1) * 100\%$. The p-values will also be output.
Descriptive summary will be provided for the exploratory endpoints at Week 52 of treatment.
UACR will be calculated using the central laboratory test results, and the specific formula is as follows:
UACR (mg/g) = urine albumin (mg/L)/ [urine creatinine (μmol/L)/1,000] * 8.84.

4.3.5. Subgroup Analysis

The following subgroup analyses will be performed for the primary efficacy endpoint.

- Previous use of hypoglycemic drugs (yes vs. no) (based on EDC records)
- Baseline HbA1c (> 8.5% vs. ≤8.5%)
- Baseline HbA1c (> 8% vs. ≤8%)
- Duration of type 2 diabetes (> 2 years vs. ≤2 years)
- Gender (males vs. females)
- Baseline BMI (< 24 kg/m2 vs. 24-28 kg/m2 vs. > 28 kg/m2)
- Age (≥65 years vs. < 65 years).

Subgroup analyses will be consistent with the primary analysis of the primary endpoint, using the ANCOVA model with baseline HbA1c value as covariate, stratification factors [prior use of hypoglycemic drugs (yes, no)], subgroup variables, treatment group, and the interaction term between subgroup variables and treatment group as fixed effects. The least squares mean differences, standard errors, 95% confidence intervals (CIs) of the change from baseline in HbA1c at Week 32 for each subgroup, as well as the p-values of the interaction term between subgroup variables and treatment group, will be estimated and plotted in the forest plot. For the subgroup of prior use of hypoglycemic drugs (yes vs no), the stratification factor [prior use of hypoglycemic drugs (yes, no)] will not be included in the fixed effects.

Based on the efficacy estimand, subgroup analyses will be performed on the key secondary efficacy endpoint of the percent change in body weight from baseline at Week 32 of treatment, with the same analysis method as that for this key secondary endpoint under the efficacy estimand.

- Gender (males vs. females);
- Baseline BMI (< 24 kg/m2 vs. 24-28 kg/m2 vs. > 28 kg/m2)
- Age (≥65 years vs. < 65 years).

Based on the efficacy estimand, the analysis of the percentage change from baseline in UACR at Week 32 of treatment for the secondary efficacy endpoint will be performed for the following subgroups, with the same analysis method as that for this secondary endpoint under the efficacy estimand.

- Baseline UACR (≤30 mg/g vs. > 30 mg/g)

4.3.6. Other Analysis

Descriptive statistical analysis will be performed for the occurrence of intercurrent events, with the assessment window for 32-week/52-week intercurrent events defined as the period from baseline to the 32-week/52-week HbA1c assessment date (or planned HbA1c assessment date if missing). All intercurrent events will be listed.

4.4. Safety Analysis

Safety analyses will be performed by treatment group for the core treatment period and the overall treatment period (core treatment period + extension period). The definitions of each treatment period are detailed in Section 4.1.8. All safety analyses will be based on SS.

4.4.1. Adverse Events

Treatment-emergent adverse event (TEAE) is defined as any adverse event that occurs after the date of first dose of the investigational product during the double-blind treatment period. Treatment-related adverse event (TRAE) is defined as a TEAE with a causal relationship to the investigational product assessed as definitely related, possibly related, or unassessable. If the relationship between adverse event and investigational product is missing, the adverse event will be considered as a treatment related adverse event.

AESIs in this study include abnormal liver function, level 3 hypoglycemia events, abnormal calcitonin, and acute pancreatitis.

All adverse events will be coded using MedDRA V28.0, and graded as mild, moderate, and severe. If the event occurs multiple times under the same system organ class (SOC) and/or preferred term (PT), the subject will be counted only once during statistical analysis. When

the same adverse event is reported multiple times in the same subject, but the severity is different, the highest grade will be counted.

4.4.1.1. Summary of TEAE

The number and percentage of subjects experiencing the following TEAEs will be summarized:

Number of subjects with TEAEs, number of subjects with TRAEs, number of subjects with SAEs, number of subjects with treatment-related SAEs, number of subjects with AESIs, number of subjects with treatment-related AESIs, number of subjects with premature discontinuation of treatment due to AEs, number of subjects with premature discontinuation of treatment due to TRAEs, number of subjects with dose reduced or temporarily discontinued due to AEs, number of subjects with dose reduced or temporarily discontinued due to TRAEs, as well as the classification of TEAE and TRAE severity, SAE, and treatment-related SAE.

4.4.1.2. TEAE by SOC and PT

- TEAE (by SOC and PT, most severe grade by SOC and PT)
- TRAE (by SOC and PT, most severe grade by SOC and PT)
- TEAEs with incidence ≥5%
- TRAEs with incidence ≥5%

4.4.1.3. SAEs classified by SOC and PT (TEAE Classified by SOC and PT)

- SAE
- Treatment-related SAEs

4.4.1.4. AESI classified by SOC and PT (TEAE Classified by SOC and PT)

- AESI
- Treatment-related AESIs

4.4.1.5. Other AEs classified by SOC and PT (TEAE Classified by SOC and PT)

- Premature discontinuation of treatment due to TEAE
- Premature discontinuation of treatment due to TRAE
- Dose reduction or temporary discontinuation due to TEAE
- Dose reduction or temporary discontinuation due to TRAE

4.4.2. Laboratory Test

Descriptive statistical summaries will be performed for laboratory tests (hematology, blood chemistry, calcitonin, lipase, amylase, etc.), including the following analyses:

- Descriptive analysis of laboratory test data (including baseline) for each visit (excluding unscheduled visits);
- Descriptive analysis for the changes from baseline at each post-baseline visit (excluding unscheduled visits).

The calculation of eGFR is detailed in Appendix 7.1, with individual values retained to two decimal places. The age used in the formula corresponds to the age at each test date, and the specific calculation rule is as follows:
Age (years) = (laboratory test date – date of birth + 1) / 365.25, rounded down.

4.4.3. Vital Signs

Vital signs (including baseline) at each visit point and their changes from baseline at each post-baseline visit point will be analyzed descriptively, excluding unscheduled visits.
Descriptive statistics will be provided for subjects with any pulse > 130 bpm, any change > 20 bpm from baseline, and any pulse > 100 bpm with a change > 15 bpm from baseline, by treatment group, including unscheduled visits.
All test results will be listed.

4.4.4. Electrocardiogram

Descriptive analysis of the electrocardiogram data (including baseline) of each visit point and the changes in electrocardiogram data from baseline at each time point post-baseline will be performed, excluding unscheduled visits.
The number and proportion of subjects with maximum heart rate > 100, > 110, > 120, and > 130 beats/min at any post-baseline assessment in each treatment group will be counted, and this analysis includes unscheduled visits.
Descriptive analysis of the most severe clinically significant changes from baseline in ECG by treatment group will be performed, with the statistical method for clinical significance being the same as that for laboratory tests, and this analysis includes unscheduled visits.
Formula of QTcF:

$$\mathrm{QTcF(msec)} = \mathrm{QT}/(RR^{0.33})$$

$$\mathrm{RR} = 60/\mathrm{Heart\ rate\ (beats/min)}$$

All test results will be listed.

4.4.5. Special Safety Analysis

Descriptive summaries will be provided for gastrointestinal adverse reactions (including TEAEs by PTs: nausea, vomiting, diarrhoea, abdominal distension, constipation, and

decreased appetite), with summaries by PT and most severe grade. The incidence and prevalence rates of gastrointestinal adverse reactions (nausea, vomiting, diarrhoea, and abdominal distension) will be calculated based on the days of first occurrence or continuous occurrence, and their changes over days will be plotted. Cumulative incidence curves over time will be drawn for gastrointestinal adverse reactions (nausea, vomiting, diarrhoea, and abdominal distension) by treatment group.

Hypoglycemic events will be summarized descriptively by level, cause of hypoglycemia, whether the event is possibly symptomatic hypoglycemia, relative hypoglycemia, presence of hypoglycemic symptoms, and whether it is an SAE, with all hypoglycemic events listed.

4.4.6. Other Safety Analysis

Descriptive summary of screening and evaluation results for diabetic retinopathy will be provided, including the following contents:

- The number of subjects with diabetic retinopathy and diabetic macular oedema in the left and right eyes, as well as the corresponding grades, will be summarized according to the planned visit points
- The number of subjects with diabetic retinopathy in both eyes will be summarized as: grade worsening (upgrade) from baseline, no change from baseline, grade improvement (downgrade) from baseline, or unassessable. This analysis includes scheduled visits and unscheduled visits. The judgment rules for grade changes are as follows:

1) The diabetic retinopathy grades of the left and right eyes at each visit will be converted into scores (conversion method is shown in Table 7);

2) The scores of the left and right eyes will be summed to generate the total score for both eyes. If the grades of lesions in both the left and right eyes are undetermined or missing, the total score for both eyes will be missing;

3) Select the highest total score of both eyes after baseline and compare it with the total score of both eyes at baseline. If the total score increases, the grade is upgraded; if the total score remains unchanged, the grade remains the same; if the total score decreases, the grade is downgraded. If the total score of both eyes is missing at baseline or any visit thereafter, the grade change is unassessable.

Table 7. Calculation Table of Single-Eye Diabetic Retinopathy Score

	No lesion	Grade I	Grade II	Grade III	Grade I-III	Grade IV	Grade V	Grade VI	Grade IV-VI	Unassessable
Score	0	1	2	3	2	4	5	6	5	0

* If the grades of lesions in both the left and right eyes are undeterminable or missing, the total score for both eyes is missing.

List all screening and assessment results for diabetic retinopathy.

Physical examination data will be listed.

4.5. Drug Exposure and Compliance

Based on the SS set, the following analyses will be performed on drug exposure and compliance:

1. The total exposure time of the investigational product, the planned dose (mg) and the actual dose (mg) during the overall treatment period will be summarized descriptively by treatment group.

   Total duration of drug exposure during the treatment period: date of last dose - date of first dose + 1;

   Planned dose (mg): cumulative total of each planned administration (as judged by the investigator);

   Actual administered dose (mg): the cumulative sum of each actual dose administered.

2. Descriptive statistical summaries of compliance with all investigational products during the run-in period and overall treatment period will be performed by treatment group. Subject compliance (%) = actual dose taken (tablet)/planned dose (tablet) × 100%.

3. Descriptive summary of the dosage achieved by each treatment group at each visit will be performed, with the specific rules as follows:

Table 8. Primary Endpoint and Dosage Judgment Criteria at Study End

**Dosage achieved at each visit (mg)**	**Mean daily dosage (mg) per visit**
0	[0,7.5]
15	[7.5,22.5)
30	[22.5,37.5)
45	[37.5,52.5)
60	[52.5,75)
90	≥75

4. All drug administration records will be listed by treatment group, subject number, treatment period, date of administration, actual administered dose, etc.

4.6. Pharmacokinetic (PK) Analysis

4.6.1. PK Concentration Analysis

Based on the PKCS, descriptive statistics will be performed for the plasma concentration of HRS-7535 across all groups, including the number of subjects, the number of observations

above the lower limit of quantification, mean, standard deviation, median, minimum, maximum, geometric mean and coefficient of variation (based on geometric mean).

4.7. PD analysis:

Not applicable.

4.8. Immunogenicity (ADA) Analysis

Not applicable.

4.9. Other Analysis

Not applicable.

4.10. Interim Analysis

Not applicable.

5. Changes to Protocol-planned Analyses

Compared with the analyses in Protocol V2.0, the following contents have been modified or clarified in this SAP:

1) The intercurrent event of "insufficient drug exposure prior to visits" has been added to the original supplementary estimand and is handled using the hypothetical strategy; meanwhile, an additional supplementary estimand has been included, applying the hypothetical strategy only to "premature discontinuation of study treatment" and "initiation of additional hypoglycemic medications", while adopting the treatment-policy strategy for "insufficient drug exposure prior to visits"
2) The protocol planned to perform ANCOVA analysis on the change from baseline in DTSQs at Week 32, which has been modified to a descriptive analysis in the SAP
3) In the protocol, the subgroup analyses of baseline BMI (≥24 kg/m2 vs. < 24 kg/m2) and duration of type 2 diabetes (≥median [months] vs. <median [months]) has been refined in the SAP to baseline BMI (< 24 kg/m2 vs. 24-28 kg/m2 vs. > 28 kg/m2) and duration of type 2 diabetes (> 2 years vs. ≤2 years).

6. References

[1] Rubin, DB. Multiple Imputation for Nonresponse in Surveys. John Wiley & Sons Inc.; 1987.

7. Appendices

APPENDIX 7.1: eGFR Calculation Formula

$$\mathrm{eGFR} = a \times \left(\frac{\text{Blood creatinine(mg/dL)}}{b}\right)^{c} \times 0.993^{\mathrm{Age}}$$

Gender	a	b	c	
Female	144	0.7	-0.329 (creatinine ≤0.7 mg/dL)	-1.209 (serum creatinine > 0.7 mg/dL)
Male	141	0.9	-0.411 (creatinine ≤0.9 mg/dL)	-1.209 (serum creatinine > 0.9 mg/dL)

Blood creatinine: 1 mg/dL = 88.4 μmol/L

Appendix 7.2: Calculation Methods for HOMA and DI

HOMA-β (%) = [20 × Fasting Insulin (μU/mL)] / [Fasting Glucose (mmol/L) - 3.5]. If fasting glucose ≤ 3.5 mmol/L, HOMA-β is not calculable and set as missing value.

HOMA-IR = [Fasting Insulin (μU/mL) × Fasting Glucose (mmol/L)] / 22.5

If applicable, the HOMA2 formula will also be used for calculation.

DI Calculation

1. Total DI (ISSI-2_cpep) based on C-peptide = (AUC_Cpep / AUC_Glu) × ISI

ISI (Matsuda)= 10000 / sqrt[(Fasting Glucose (mg/dL) × Fasting Insulin (μU/mL)) × (Mean Mixed-meal Glucose (mg/dL) × Mean Mixed-meal Insulin (μU/mL))]

AUC_Cpep = 0.25 × Fasting C-peptide (pmol/L) + 0.5 × 0.5-hour Postprandial C-peptide (pmol/L) + 0.75 × 1-hour Postprandial C-peptide (pmol/L) + 0.5 × 2-hour Postprandial C-peptide (pmol/L)

AUC_Glu = 0.25 × Fasting Glucose (mmol/L) + 0.5 × 0.5-hour Postprandial Glucose (mmol/L) + 0.75 × 1-hour Postprandial Glucose (mmol/L) + 0.5 × 2-hour Postprandial Glucose (mmol/L)

Mean Mixed-meal Glucose (mg/dL) = (Fasting Glucose (mg/dL) + 0.5-hour Postprandial Glucose (mg/dL) + 1-hour Postprandial Glucose (mg/dL) + 2-hour Postprandial Glucose (mg/dL)) / 4

Mean Mixed-meal Insulin = (Fasting Insulin (μU/mL) + 0.5-hour Postprandial Insulin (μU/mL) + 1-hour Postprandial Insulin (μU/mL) + 2-hour Postprandial Insulin (μU/mL)) / 4

Conversion of glucose units: 1 mg/dL = 0.0555062 mmol/L.

Conversion of C-peptide units: 1 ng/ml = 331.094 pmol/L

2. If applicable, the following types of DI will also be calculated:

The early-phase disposition index based on insulin (DI_ins) = Insulin Sensitivity Index (ISI) × Insulinogenic Index (IGI)

Based on C-peptide early-phase DI (DI_cpep) = Insulin Sensitivity Index (ISI) × C-peptide Generation Index (CGI)

Total DI based on insulin (ISSI-2_ins)= (AUC_Ins / AUC_Glu) × ISI

IGI($\Delta I_{30}/\Delta G_{30}$)=(0.5-hour Postprandial Insulin (μU/mL) – Fasting Insulin (μU/mL)) / (0.5-hour Postprandial Glucose (mmol/L) – Fasting Glucose (mmol/L))

CGI($\Delta C_{30}/\Delta G_{30}$)=(0.5-hour Postprandial C-peptide (pmol/L) - Fasting C-peptide (pmol/L)) / (0.5-hour Postprandial Glucose (mmol/L) - Fasting Glucose (mmol/L))

AUC_Ins = 0.25 × Fasting Insulin (μU/mL) + 0.5 × 0.5-hour Postprandial Insulin (μU/mL) + 0.75 × 1-hour Postprandial Insulin (μU/mL) + 0.5 × 2-hour Postprandial Insulin (μU/mL)

If the 0.5-hour postprandial glucose ≤ fasting glucose, or 0.5-hour postprandial insulin ≤ fasting insulin under fasting conditions, or 0.5-hour postprandial C-peptide ≤ fasting C-peptide, then IGI, CGI and their corresponding DI are not calculable and set as missing values.

APPENDIX 7.3: SAS CODE EXAMPLE

(1) Example code for the ANCOVA Model:

```
Proc mixed data=data;
Class trt strata;
Model y = base trt strata/solution;
Lsmeans trt/diff cl;
Run;
```

(2) Example code for multiple imputation based on MAR:

```
Proc mi data=data seed=7535301 nimpute=30 out=data_aftermar;
class trt;
fcs regpmm(aval4= base trt);
fcs regpmm(aval8= base aval4 trt );
fcs regpmm(aval12= base aval4 aval8 trt );
fcs regpmm(aval16= base aval4 aval8 aval12 trt);
var base aval4 aval8 aval12 aval16 trt;
run;
```

proc mi statement: seed is the number of seeds used to ensure reproducibility of the imputation results, nimpute specifies the number of imputations, and minimum sets the minimum value after imputation to 0.
class statement: Specifies the categorical variables in the var statement.
fcs reg statement: variables like aval4 represent the visit variables converted from longitudinal data in ADaM format to cross-sectional format.
var statement: Includes all variables that are part of the imputation model.

(3) Example code for combining analysis results based on Rubin's Rules:

```
/*Output analysis results first*/
proc mianalyze data= CommonPdiffTests;
by trt;
modeleffects RiskDifference;
stderr stdErr;run;
```

proc mianalyze statement: parms specifies the dataset containing the analysis results that need to be combined.
modeleffects statement: list the factors in the dataset that need to be analyzed.
Lists the standard errors for the factors being analyzed.

(4) Example code for the MMRM Model:

```
proc mixed data=dataset;
        class treat time subj;
        model y=base treat time treat*time/solution ddfm=kr;
        repeated time/ subject=subj type=un;
        lsmeans treat*time / diff cl;
run;
```

class statement: Includes variables used in the analysis, such as treatment group (treat), visit time points (time), and subjects (subj).
model statement:
(a) y is the response variable, i.e., the measured value (the percentage of HbA1c change from baseline in the primary efficacy analysis in this study). The model includes base, treat, time, and their interaction treat*time;
(b) The solution option displays estimates of fixed effects;

(c) The ddfm option specifies the method for calculating denominator degrees of freedom. As the repeated statement specifies type=un, ddfm=kr is specified.

repeated statement:

(a) time is the repeated effect;

(b) The subject option specifies subjects (in this study) in the mixed model, here as subject=subj;

(c) The type option specifies the covariance structure, which in this MMRM analysis is Unstructured (type=un).

lsmeans statement:

(a) lsmeans treat*time tests differences in Least Squares Means between treatment groups across different time points;

(b) The diff option displays differences in Least Squares Means;

(c) The cl option calculates confidence intervals for each Least Squares Means, defaulting to 95% CI.

(5) Example code of GEE model:

```
proc gee data=test;
class trt visit strata2;
model Responder = base trt visit strata2 trt*visit / dist=bin link=logit;
repeated subject= subjid / type=UN covb corrw;
lsmeans trt*visit/diff cl exp;
run;
```

When the data volume is small, it may not converge and have errors. The TYPE could be changed to CS, AR, etc.

Publication Statistical Analysis Plan (pSAP)

HRS-7535 Phase III Clinical Studies

**Author**	Tingyan Zhong
**Company**	Jiangsu Hengrui Pharmaceuticals Co., Ltd.
**Version**	V1.0
**Date**	23 Apr., 2026

Table of Contents

1. **Version Revision** 3
2. **Background** 3
3. **Study Information** 3
4. **Statistical Methods** 5
   - **4.1. General Considerations** 5
   - **4.2. Analysis Sets** 5
   - **4.3. Missing Data** 5
   - **4.4. Participant Description** 5
   - **4.5. Demographics** 5
   - **4.6. Exploratory Analysis** 5
5. **References** 6
6. **Appendices** 7

1. Version Revision

Initial version, not applicable.

2. Background

This publication statistical analysis plan (pSAP, V1.0) describes the statistical methods for exploratory analyses of HRS-7535 Phase III clinical data, conducted by Jiangsu Hengrui Pharmaceuticals Co., Ltd. This pSAP was finalized before unblinding of any HRS-7535 Phase III study and serves as a supplement to each study's SAP. The results will support manuscript publication.

3. Study Information

This pSAP (V1.0) covers two HRS-7535 Phase III studies in T2DM, namely HRS-7535-301 and HRS-7535-302 (hereinafter referred to as "the two Phase III studies"). The overview of each study is shown in Table 1 below:

Table 1 Overview of HRS-7535 Phase III Studies in T2DM

Protocol No./Version No.:	Study Design	Investigational Product and Dosage	Study Population	Primary Endpoint	Study Duration	Study Status
HRS-7535-301/V2.0	randomized, double-blind, placebo-controlled, parallel-group Phase III clinical trial	QD dosing, 32-week core treatment period:<br>HRS-7535 30mg/<br>60mg (30mg[4w]-60mg[28w])/<br>90mg (30mg[4w]-60mg[4w]-90mg[24w])/<br>Placebo<br>20-week extension treatment period:<br>From week 33 of the double-blind treatment period to the end of the double-blind treatment period, the placebo group will be switched to HRS-7535 30 mg treatment. The other treatment groups will maintain the original dosage.	Adult subjects with type 2 diabetes with poor glycemic control by diet and exercise alone	The change in HbA1c from baseline at week 32 in the HRS-7535 group compared with the placebo group	Core treatment period 32 weeks + extended treatment period 20 weeks + follow-up period 2 weeks	Ongoing, database lock is expected on 24 Apr., 2026
HRS-7535-302/V1.3	Randomized, double-blind, positive-controlled, parallel-designed Phase III clinical trial	QD dosing, 32-week core treatment period:<br>HRS-7535 30mg/60mg (30mg[4w]-60mg[48w])/<br>90mg(30mg[4w]-60mg[4w]-90mg[44w])/<br>Dapagliflozin 10 mg	T2DM patients who have poor glycemic control despite being treated with metformin	Changes in HbA1c from baseline at week 32 in the HRS-7535 group compared with the dapagliflozin group	Core treatment period 32 weeks + extension treatment period 20 weeks + follow-up period 2 weeks.	Ongoing, expected database lock on 30 May., 2026

4. Statistical Methods

4.1. General Considerations

The definition of the analysis window for efficacy data is the same as that in the two Phase III studies [1,2]. Unless otherwise specified, all efficacy analyses are calculated using the central laboratory sampling results.

In general, continuous variables (such as age) will be statistically described using the number of observations, mean, median, standard deviation (SD), and minimum and maximum values. The decimal places of the minimum and maximum values are consistent with those recorded in the CRF (but only two decimal places will be retained for raw data with more than two decimal places). The mean and median will have one more decimal place than the raw data, and the SD will have two more decimal places than the raw data. Categorical variables will be summarized using descriptive statistics as frequencies and percentages of each category, with percentages maintained to one decimal place.

4.2. Analysis Sets

The definitions of the ITT analysis set and safety analysis set are the same as those in the two Phase III studies.

4.3. Missing Data

Unless otherwise specified, all imputations are the same as those in the two Phase III studies.

4.4. Participant Description

The statistical method for participant distribution is the same as that in the two Phase III studies.

4.5. Demographics

The statistical methods for baseline demographics are the same as those in the two Phase III studies.

4.6. Exploratory Analysis

If data conditions permit, exploratory mediation analysis will be conducted to evaluate the indirect effects of HRS-7535 on β-cell function through changes in glucose

metabolism (HbA1c, FPG) or percentage change in body weight, as well as the direct effects on β-cell function indicators. β-cell function indicators include but are not limited to:

- ISSI-2 (insulin Secretion-Sensitivity Index-2)
- DI (disposition index, insulinogenic index × ISI)
- HOMA-β
- HOMA-IR

Specifically, the analysis models include but are not limited to the regression-based mediation effect model [3]. This model takes treatment as the independent variable, baseline values of the dependent variable as covariates, change from baseline in HbA1c, FPG, or percentage change in body weight as mediating variables, and β-cell function indicators as dependent variables, providing point estimates and 95% CIs (calculated based on the Bootstrap method) for direct and indirect effects.

If data conditions permit, a supplementary analysis of the 52-week glycemic endpoint will be conducted. The intercurrent event of insufficient drug exposure is defined as the actual total drug exposure within 90 days prior to the visit point (HbA1c measurement date) (based on dosing at the previous two visits) being less than 50% of the target total exposure, or a 52-week PK measurement value below BLQ. A hypothetical strategy is applied to the three intercurrent events (premature discontinuation of study treatment, initiation of additional glucose-lowering drugs, and insufficient drug exposure prior to visit), with data after intercurrent events excluded from the analysis. The analysis is performed using a mixed-effects model for repeated measures (MMRM) based on restricted maximum likelihood (REML). This model includes stratification factors (prior use of glucose-lowering drugs [yes, no]), treatment, visit, and the interaction between treatment and visit as fixed effects, with baseline HbA1c as the covariate. The within-subject variability is modeled using an unstructured (UN) or compound symmetry (CS) covariance matrix. The least squares mean, standard error, 95% CI of the change from baseline in HbA1c at Week 52, and the nominal p-value for the difference between each HRS-7535 dosage group and the placebo group are estimated and presented.

5. References

[1] HRS-7535-301 Statistical Analysis Plan V1.0 2026.04.23;

[2] HRS-7535-302 Statistical Analysis Plan V1.0 is being drafted, with the estimated finalization date in May 2026;
[3] MacKinnon DP. Introduction to Statistical Mediation Analysis. New York, NY: Routledge; 2008.

6. Appendices

None.
