# Supplementary material for: Oral small-molecule GLP-1 receptor agonist safiglipron in early type 2 diabetes: a randomized, double-blind, placebo-controlled trial
Source: Nat Med. 2026 Sep 29;32(11):4330–9. doi: 10.1038/s41591-026-04651-9 (PMC13768188; doi:10.1038/s41591-026-04651-9)
Supplement: Supplementary file 2 [file 41591_2026_4651_MOESM2_ESM.pdf]

nature portfolio

Corresponding author(s): Xinhua Xiao, Zi Ye

Last updated by author(s): Aug 12, 2026

Reporting Summary

Nature Portfolio wishes to improve the reproducibility of the work that we publish. This form provides structure for consistency and transparency in reporting. For further information on Nature Portfolio policies, see our Editorial Policies and the Editorial Policy Checklist.

Statistics

For all statistical analyses, confirm that the following items are present in the figure legend, table legend, main text, or Methods section.

n/a	Confirmed	
☐	☒	The exact sample size (*n*) for each experimental group/condition, given as a discrete number and unit of measurement
☐	☒	A statement on whether measurements were taken from distinct samples or whether the same sample was measured repeatedly
☐	☒	The statistical test(s) used AND whether they are one- or two-sided<br>*Only common tests should be described solely by name; describe more complex techniques in the Methods section.*
☐	☒	A description of all covariates tested
☐	☒	A description of any assumptions or corrections, such as tests of normality and adjustment for multiple comparisons
☐	☒	A full description of the statistical parameters including central tendency (e.g. means) or other basic estimates (e.g. regression coefficient) AND variation (e.g. standard deviation) or associated estimates of uncertainty (e.g. confidence intervals)
☐	☒	For null hypothesis testing, the test statistic (e.g. *F*, *t*, *r*) with confidence intervals, effect sizes, degrees of freedom and *P* value noted<br>*Give P values as exact values whenever suitable.*
☒	☐	For Bayesian analysis, information on the choice of priors and Markov chain Monte Carlo settings
☐	☒	For hierarchical and complex designs, identification of the appropriate level for tests and full reporting of outcomes
☒	☐	Estimates of effect sizes (e.g. Cohen's *d*, Pearson's *r*), indicating how they were calculated

Our web collection on statistics for biologists contains articles on many of the points above.

Software and code

Policy information about availability of computer code

Data collection	Data were collected using the HRTAU EDC (version 4.1.20).
Data analysis	Statistical analyses were performed using SAS (version 9.4). Sample-size calculations were performed using East (version 6.5.4). The prespecified exploratory mediation analyses were performed using Python (version 3.12).

For manuscripts utilizing custom algorithms or software that are central to the research but not yet described in published literature, software must be made available to editors and reviewers. We strongly encourage code deposition in a community repository (e.g. GitHub). See the Nature Portfolio guidelines for submitting code & software for further information.

Data

Policy information about availability of data

All manuscripts must include a data availability statement. This statement should provide the following information, where applicable:

- Accession codes, unique identifiers, or web links for publicly available datasets
- A description of any restrictions on data availability
- For clinical datasets or third party data, please ensure that the statement adheres to our policy

Qualified researchers may request access to anonymized individual participant data that underlie the results reported in this article. Data will be made available beginning 24 months after primary publication of the trial results. Access will be provided to researchers who provide a methodologically sound proposal for a specific purpose. Proposals should be directed to the corresponding authors: Xinhua Xiao (xiaoxh2014@vip.163.com) or Zi Ye (zi.ye@hengrui.com). Requests will be

evaluated by the corresponding authors and sponsor on the basis of scientific merit, methodological feasibility, consistency with participants' informed consent and protection of participant confidentiality. Applicants will normally be informed of the decision within 8 weeks. Approved access will be subject to execution of a data-use agreement, and data will be shared through a secure data-transfer process. Source data are provided with this paper.

Research involving human participants, their data, or biological material

Policy information about studies with human participants or human data. See also policy information about sex, gender (identity/presentation), and sexual orientation and race, ethnicity and racism.

Reporting on sex and gender	Sex was recorded from the national identification document used for trial registration; gender identity was not assessed separately. Participants were enrolled regardless of sex. Sex was a prespecified subgroup variable for the primary endpoint, although the trial was not powered for sex-specific treatment comparisons.
Reporting on race, ethnicity, or other socially relevant groupings	All participants were categorized as Asian; ethnicity and other socially relevant groupings were not collected.
Population characteristics	Participants were adults aged 18–75 years with type 2 diabetes inadequately controlled with diet and exercise alone. Demographic and baseline clinical characteristics were generally balanced across treatment groups and are presented in Table 1.
Recruitment	Potential participants were recruited by investigators at 46 participating hospitals in China and underwent protocol-defined screening after providing written informed consent. Screening began on 25 September 2024, and randomization occurred from 21 October 2024 to 31 March 2025. Of 475 individuals screened, 375 entered the 3-week single-blind placebo run-in and 284 were randomized. Voluntary, hospital-based participation, the eligibility criteria and the run-in may have selected participants with relatively early type 2 diabetes who were more motivated and adherent than the broader clinical population. Enrollment exclusively in China may further limit generalizability. However, these selection processes occurred before randomization; randomized allocation, double-blinding and balanced baseline characteristics minimized their potential influence on between-group treatment comparisons.
Ethics oversight	The protocol, amendment and informed consent materials were approved by the Ethics Committee of Peking Union Medical College Hospital, the coordinating center, and by the institutional review board or independent ethics committee at each of the 46 participating hospitals named in Supplementary Table 1. The trial was conducted in accordance with the Declaration of Helsinki and Good Clinical Practice guidelines. All participants personally provided written informed consent before undergoing any study-specific procedures. Participants received transportation reimbursement and compensation for blood collection, as specified in the ethics committee-approved informed consent materials.

Note that full information on the approval of the study protocol must also be provided in the manuscript.

Field-specific reporting

Please select the one below that is the best fit for your research. If you are not sure, read the appropriate sections before making your selection.

☒ Life sciences ☐ Behavioural & social sciences ☐ Ecological, evolutionary & environmental sciences

For a reference copy of the document with all sections, see nature.com/documents/nr-reporting-summary-flat.pdf

Life sciences study design

All studies must disclose on these points even when the disclosure is negative.

Sample size	The planned sample size was 272 participants, with 68 participants per group. Assuming an overall treatment difference of −0.72 percentage points in HbA1c change from baseline to week 32 between each safaglipron dose and placebo, an SD of 1.1 percentage points, and a one-sided α of 0.025, this sample size provided 90% overall power to show superiority of all three safigliptron doses versus placebo for the primary endpoint.
Data exclusions	All 284 randomized participants were included in the intention-to-treat population for efficacy analyses. The safety population included all participants who received at least one dose of study treatment. No participants were otherwise excluded from the prespecified analysis populations.
Replication	Experimental replication was not applicable to this randomized clinical trial. Reproducibility was supported by the prespecified protocol and SAP and by standardized procedures implemented across 46 sites.
Randomization	Participants were randomly assigned (1:1:1:1) to safigliptron 30 mg, 60 mg, or 90 mg, or placebo using a centralized interactive web response system. Randomization used a block design and was stratified by baseline HbA1c (≤8.5% vs >8.5%) and previous use of glucose-lowering medication (yes vs no).
Blinding	Safigliptron and matching placebo tablets were identical in appearance. Participants, investigators, site staff, outcome assessors, and sponsor personnel involved in trial conduct remained masked to treatment assignment throughout the 32-week placebo-controlled period. During the extension period, participants originally assigned to placebo switched to safigliptron 30 mg at week 33 while masking was maintained. Data analysts remained masked until database lock.

Reporting for specific materials, systems and methods

We require information from authors about some types of materials, experimental systems and methods used in many studies. Here, indicate whether each material, system or method listed is relevant to your study. If you are not sure if a list item applies to your research, read the appropriate section before selecting a response.

Materials & experimental systems

n/a	Involved in the study
☒	☐ Antibodies
☒	☐ Eukaryotic cell lines
☒	☐ Palaeontology and archaeology
☒	☐ Animals and other organisms
☐	☒ Clinical data
☒	☐ Dual use research of concern
☒	☐ Plants

Methods

n/a	Involved in the study
☒	☐ ChIP-seq
☒	☐ Flow cytometry
☒	☐ MRI-based neuroimaging

Clinical data

Policy information about clinical studies

All manuscripts should comply with the ICMJE guidelines for publication of clinical research and a completed CONSORT checklist must be included with all submissions.

Clinical trial registration	The trial was prospectively registered with Chinadrugtrials.org.cn (CTR20243559), in accordance with Chinese regulatory requirements, before enrollment of the first participant. Registration with ClinicalTrials.gov (NCT06672172) was subsequently completed after enrollment had begun to improve the international accessibility of the trial record.
Study protocol	The full trial protocol and statistical analysis plan are provided in the Supplementary Information.
Data collection	The trial was conducted at 46 hospitals in China. Screening began on 25 September 2024, participants were randomized from 21 October 2024 to 31 March 2025, and the last participant completed trial follow-up on 17 April 2026. Data were collected during screening, the 3-week single-blind placebo run-in, the 52-week treatment period and the 2-week safety follow-up. Scheduled visits occurred at baseline and weeks 2, 4, 8, 12, 16, 20, 26, 32, 42 and 52. At scheduled visits, clinical measurements were obtained, blood and urine samples were collected, and adverse events, concomitant medications and treatment adherence were recorded using the HRTAU EDC (version 4.1.20).
Outcomes	The primary endpoint was change from baseline in HbA1c at week 32. The confirmatory secondary endpoints, all assessed at week 32, were the proportions of participants achieving HbA1c <7.0% and ≤6.5%, change from baseline in fasting plasma glucose and percentage change from baseline in bodyweight. HbA1c and fasting plasma glucose were measured in venous blood samples, and bodyweight was measured at scheduled study visits.<br>Prespecified supportive secondary endpoints assessed at weeks 32 and 52 included bodyweight, waist circumference, HbA1c target achievement, rescue therapy use, seven-point self-monitored blood glucose profiles, fasting and mixed-meal tolerance test-derived glucose, insulin and C-peptide measures, HOMA-β, HOMA-IR and disposition index. During the mixed-meal tolerance test, venous blood samples were collected after an overnight fast and at 30, 60 and 120 min after the standardized meal. Treatment satisfaction was assessed using the Diabetes Treatment Satisfaction Questionnaire status version.Prespecified exploratory endpoints assessed at weeks 32 and 52 included blood pressure, lipid parameters and urinary albumin-to-creatinine ratio. Safety assessments included adverse events, hypoglycemic episodes, clinical laboratory tests, vital signs, 12-lead electrocardiograms and diabetic retinopathy assessment by fundus photography.

Plants

Seed stocks	Not applicable. This study did not use any plant materials or seed stocks.
Novel plant genotypes	Not applicable. No novel plant genotypes (transgenic, gene-edited, mutagenized, or hybridized lines) were generated or used.
Authentication	Not applicable. Plant authentication procedures were not relevant to this study.
